# Alkali-ion-modified zeolitic imidazolate framework glasses

**DOI:** 10.1038/s41557-026-02115-8

**Published:** 2026-05-04

**Authors:** Pascal Kolodzeiski, Benjamin M. Gallant, Lennard Richter, Mario Antonio T. Ongkiko, Carlo Franke, Aleksander Kostka, Wen-Long Xue, Chinmoy Das, Jan-Benedikt Weiß, Elena Kolodzeiski, Thomas Kress, Gregor Kieslich, Tong Li, Andrew J. Morris, Dominik Kubicki, Sebastian Henke

**Affiliations:** 1 Fakultät für Chemie und Chemische Biologie, Technische Universität Dortmund, Dortmund, Germany; 2 School of Chemistry, University of Birmingham, Birmingham, UK; 3 School of Metallurgy and Materials, University of Birmingham, Birmingham, UK; 4 Faculty of Mechanical Engineering, Atomic-scale Characterisation, Ruhr-Universität Bochum, Bochum, Germany; 5 Center for Interface-Dominated High Performance Materials (ZGH), Ruhr-Universität Bochum, Bochum, Germany; 6 Department of Chemistry, SRM University-AP, Amaravati, India; 7 Department of Chemistry, TUM School of Natural Sciences, Technical University of Munich, Garching, Germany; 8 Yusuf Hamied Department of Chemistry, University of Cambridge, Cambridge, UK

**Keywords:** Metal-organic frameworks, Solid-state chemistry, Materials chemistry

## Abstract

Modifying glass compositions is key to creating silicate-based glasses for technologies including optical fibres, catalytic supports, protective coatings and separation membranes. Here we extend this concept to metal–organic framework (MOF) glasses by modifying the MOF glass former ZIF-62 with Li(bim) and Na(bim) as compatible glass modifiers (benzimidazolate, bim^−^). Melt-quenching of physical mixtures with increasing Na(bim) content yields modified MOF glasses that exhibit a systematic decrease in the glass transition temperature (*T*_g_), accompanied by increased liquid fragility, configurational heat capacity at *T*_g_ and density: paralleling silicate glass chemistry through partial network depolymerization. Structural and spectroscopic analysis, coupled with density-functional theory calculations, confirm that Na(bim) is incorporated homogeneously into the MOF glass framework rather than the pores and reveal the presence of undercoordinated sodium ion environments. Finally, extraction of the modifier by water treatment increases glass porosity, akin to established borosilicate glass processes. This work introduces a transferable approach for tailoring the structure and properties of MOF glasses.

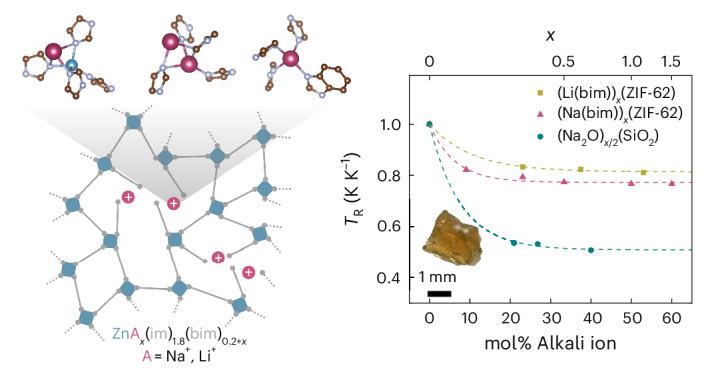

## Abstract (web summary)

The properties of metal–organic framework glasses can be modulated by additives, but understanding how they modify the glass network is challenging. Now, alkali-modifier sites in MOF glasses have been identified, and the impact of both modifier content and identity on the processing temperatures and hierarchical porosity has been examined.

## Main

**Fig. 1:**
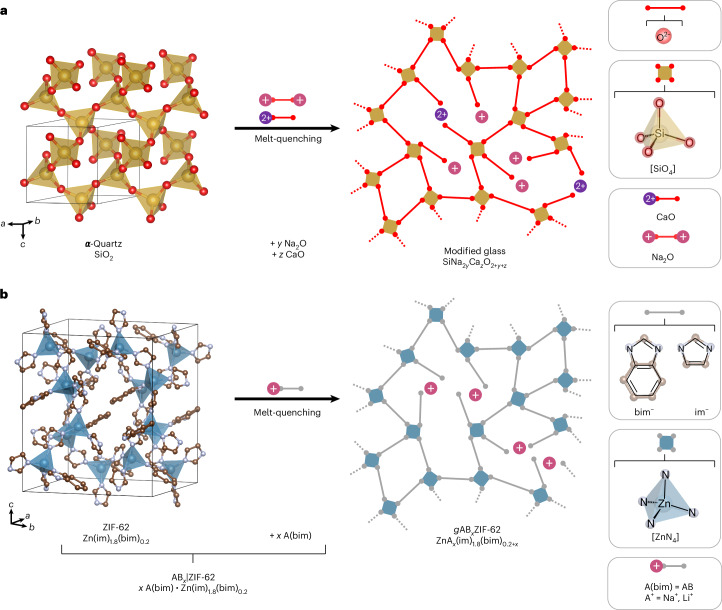
Conceptual scheme of glass modification in silicate and MOF glasses. **a**, Traditional modification of SiO_2_ glass with Na_2_O and CaO as network modifiers. **b**, Extension of this concept to the MOF glass former ZIF-62 using A(bim) (A = Na, Li) as modifiers. The schematics illustrate the crystal structures of SiO_2_ and ZIF-62, alongside a 2D depiction of the modified glass networks, highlighting the effect of modifiers on SiO_2_ or Zn(im/bim)_2_ glass network connectivity. In the MOF case, physical mixtures are labelled AB_*x*_|ZIF-62, with the corresponding glasses denoted *g*AB_*x*_ZIF-62, where *x* represents the molar amount of A(bim) added per mole ZIF-62. Hydrogens were omitted for clarity.

Glass manufacturing dates back to ancient civilizations, with early glass fragments from Egypt and Mesopotamia demonstrating an early understanding of how additives influence silicate glass properties^1^. The addition of fluxes, such as metal oxides and carbonates, to SiO_2_ lowers processing temperatures and facilitates glass formation by disrupting the network of corner-sharing [SiO_4_] tetrahedra^2^ (Fig. 1a). Modifiers such as Na_2_O reduce the number of bridging oxygens, creating non-bridging oxygens and depolymerizing the framework, thereby altering the glass’s thermal, optical and mechanical properties^2,3^. This compositional tunability underpins the versatility of silicate glasses, from everyday products to advanced aerospace and pharmaceutical applications^4,5^, and continues to drive innovation across diverse industries^3^.

Recently, metal–organic framework (MOF) glasses emerged as a new class of glass formers offering unique structural and functional properties^6,7^. Among them, zeolitic imidazolate frameworks (ZIFs) have garnered much attention due to their structural similarities to aluminosilicate zeolites, including tetrahedral coordination of the metal ions and similar metal–ligand–metal bond angles^8,9^. ZIF-62 (Zn(im)_1.8_(bim)_0.2_, where im^−^ is imidazolate and bim^−^ is benzimidazolate) is one of the first ZIFs shown to exhibit melting on heating and glass formation on subsequent cooling of the melt (that is, melt-quenching)^10^. Its high glass-forming ability, partial retention of microporosity, absence of grain boundaries and melt-processability make ZIF-62 glass attractive for separation and membrane applications^11–15^. However, the number of MOFs capable of forming glasses is strongly limited, and the factors governing their glass formation remain a topic of vast research interest^16–20^.

To broaden the structural and compositional space of ZIF glasses, previous studies have (1) modified the crystalline glass former via linker and/or node substitution or guest impregnation to shift melting and glass formation^11,17,18,21–27^, and (2) mixed the glass former with additional phases, yielding heterogeneous composites^28,29^ or, in some cases, flux-melted and/or blended glasses^30–32^. Although effective, these approaches are either synthesis-intensive and system-specific or lack a unifying framework for how added components modify the glass network. The deliberate use of a ‘modifier’ in the sense established for silicate glasses remains largely unexplored for MOF glasses.

Inspired by the traditional principles of silicate glass modification, we show the modification of ZIF-62 glass using sodium benzimidazolate Na(bim) and lithium benzimidazolate Li(bim)^33^ as modifiers (Fig. 1b). We comprehensively examine the structural, thermal and chemical properties of the modified glass as a function of modifier concentration, demonstrating reduced glass transition temperatures (*T*_g_) and increased liquid fragilities, aiding processing and shaping. Moreover, we demonstrate that extraction of the incorporated modifier via water leaching increases glass porosity. This behaviour is analogous to the Vycor process in silicate glass manufacturing^34,35^ and highlights how concepts from traditional glass chemistry can be transferred to MOF-based glasses.

## Results and discussion

### ZIF glass modification with Na(bim)

**Fig. 2:**
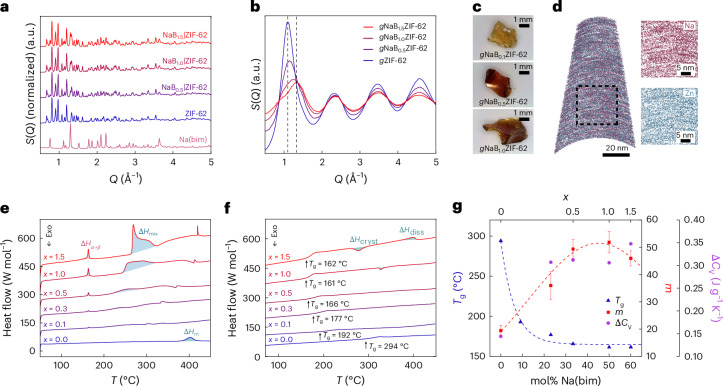
X-ray total scattering, thermal analysis, and microstructural and microscopic characterization of Na(bim)-modified ZIF glasses. **a**,**b**, X-ray total scattering functions *S*(*Q*) of the crystalline compounds and selected physical mixtures (**a**), and the corresponding glasses (**b**). Dashed lines mark the FSDP positions for non-modified and heavily modified glasses. **c**, Optical microscopy images of glass shards from *g*NaB_*x*_ZIF-62 preparations using the autoclave approach. **d**, Slice of the reconstructed APT data for *g*NaB_0.3_ZIF-62, displaying the Na and Zn ion distributions. The right insets detail the Na (top) and Zn (bottom) ion distributions in the highlighted region. **e**, DSC thermograms of NaB_*x*_|ZIF-62 mixtures across different *x* values recorded at 10 °C min^−1^. Endothermic events correspond to ZIF-62 melting (Δ*H*_m_), the *α* → *β* phase transition of Na(bim) (Δ*H*_*α*→*β*_), and reaction and intermixing processes Δ*H*_mix_. **f**, DSC thermograms of *g*NaB_*x*_ZIF-62 materials with varying *x* recorded at 10 °C min^−1^, highlighting the glass transition onset (*T*_g_, arrow). Endothermic and exothermic (Exo) events are attributed to crystallization (Δ*H*_cryst_) and dissolution (Δ*H*_diss_) of the dense ZIF-7-III phase. Scans are offset by 80 W mol^−1^ (molar unit: ZnNa_*x*_(im)_1.8_(bim)_0.2+*x*_). **g**, Trends in *T*_g_, the liquid fragility index (*m*) and heat capacity change (Δ*C*_V_) at *T*_g_ for *g*NaB_*x*_ZIF-62. Error bars for *m* represent the standard deviation (1*σ*) derived from linear fits to DSC data. The dashed lines are visual guides.

Physical mixtures of the glass former ZIF-62 and the modifier Na(bim) in varying ratios were prepared by grinding of the crystalline starting materials. All handling was done under inert atmosphere due to the hygroscopic nature of Na(bim). The derived physical mixtures are denoted as NaB_*x*_|ZIF-62 and feature variable composition *x*(Na(bim))·Zn(im)_1.8_(bim)_0.2_, with *x* = 0.1, 0.3, 0.5, 1.0 and 1.5. Heating these mixtures to 450 °C in a differential scanning calorimetry (DSC) setup (small scale, roughly 10 mg) or in a custom-made hermetic steel crucible with a tube furnace (autoclave approach, roughly 250 mg; Supplementary Fig. 9), followed by cooling to room temperature yielded amorphous materials as confirmed by powder X-ray diffraction (PXRD) (Fig. 2a,b). The obtained materials exhibit clear signs of macroscopic material flow and glass shard formation (Fig. 2c and Supplementary Fig. 6). At low Na(bim) concentrations (*x* = 0.1), the melt-quenched glasses (denoted as *g*NaB_*x*_ZIF-62) contained crystalline residues of ZIF-zni (Zn(im)_2_)^36^ when held at 450 °C for only 30 min (Supplementary Fig. 18). Extending the isothermal hold to 1 h resulted in fully amorphous materials, even for *x* = 0.1 (Supplementary Fig. 19). This tempering step was essential for melt homogenization (Methods).

Solution-phase ^1^H nuclear magnetic resonance (NMR) spectroscopy of digested glass samples verified the absence of organic decomposition products following glass preparation (Supplementary Figs. 22–30). Simultaneous thermal analysis, combining thermogravimetry with differential thermal analysis, revealed a reduced decomposition temperature (*T*_d_) of ~480–540 °C for the modified glasses, compared with *g*ZIF-62 (*T*_d_ = 603 °C; Supplementary Figs. 11–14). Elemental mapping via scanning transmission electron microscopy (TEM) with energy dispersive X-ray spectroscopy (STEM-EDX) confirmed a homogeneous distribution of Zn^2+^ and Na^+^ ions in the *g*NaB_*x*_ZIF-62 materials with both low (*x* = 0.3) and high (*x* = 1.0) modifier contents (Supplementary Figs. 84 and 86). Unfortunately, quantitative STEM-EDX analysis of Zn and Na was limited by overlapping emission lines. Further insights into the three-dimensional distribution of the Zn-containing and Na-containing (complex) ions in the modified glasses were obtained by atom probe tomography (APT) on *g*NaB_0.3_ZIF-62 (Fig. 2d). APT maps the three-dimensional spatial distribution of individual elements with subnanometre resolution^37,38^. The 3D-APT reconstruction reveals a homogeneous distribution of Zn-containing and Na-containing species at the nanometre scale, which was confirmed by a statistical analysis comparing the experimental with computed randomized data (Supplementary Fig. 83).

### Thermal behaviour

**Extended Data Fig. 1:**
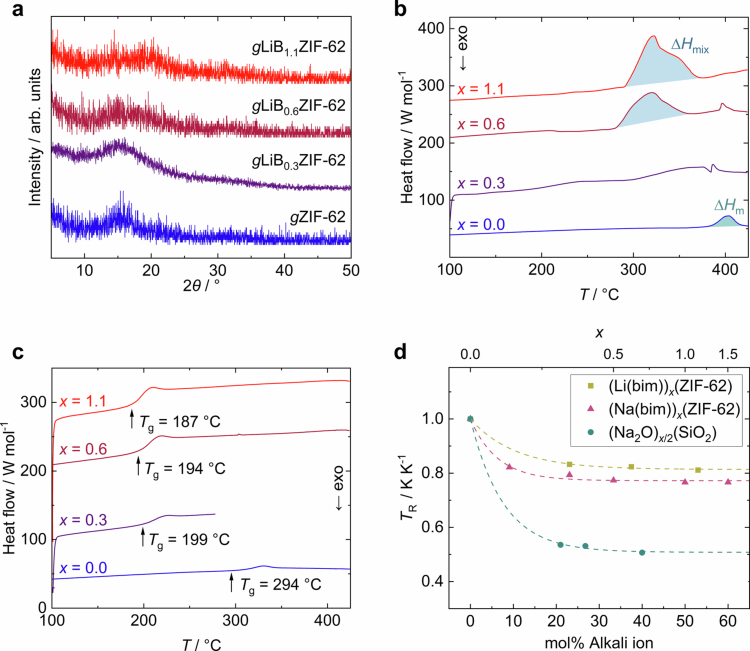
Structural and thermal analysis of Li(bim)-modified ZIF glasses. **a**, PXRD pattern of *g*LiB_*x*_ZIF-62 glasses collected using CuKα radiation. **b**, DSC thermograms of LiB_*x*_|ZIF-62 mixtures across different *x* values recorded at 10 °C min^−1^. Endothermic events correspond to ZIF-62 melting (Δ*H*_m_), and reaction and intermixing processes Δ*H*_mix_. **c**, DSC thermograms of *g*LiB_*x*_ZIF-62 materials with varying modifier contents (*x*) recorded at 10 °C min^−1^, highlighting the glass transition onset (*T*_g_, arrow). Scans are offset by 80 W mol^−1^ (molar unit: ZnLi_*x*_(im)_1.8_(bim)_0.2+*x*_), respectively. **d**, Reduced glass transition temperature *T*_R_ = *T*_g_(modified glass) / *T*_g_(glass former) for Na(bim) and Li(bim)-modified ZIF-62 glasses and sodium silicate glasses^63^ as a function of the alkali modifier content. Lines are exponential fits to the data and serve as a guide to the eye.

Cyclic DSC and in situ variable-temperature (VT)-PXRD provide comprehensive insights into the thermal behaviour of the NaB_*x*_|ZIF-62 physical mixtures and the resulting glasses. In the first DSC heating scans, weak endothermic signals at 166 °C correspond to the solid-state *α* → *β* phase transition of Na(bim), consistent with previous findings^33^ (Fig. 2e). This is followed by overlapping endothermic events that scale with Na(bim) concentration, indicating simultaneous melting, material intermixing and reaction processes during melt homogenization up to 450 °C (Supplementary Figs. 40–43). The flux-melting process is corroborated by VT-PXRD data, which show the complete disappearance of Bragg reflections for Na(bim) and ZIF-62 around 350 °C, followed by internal crystallization of either the dense ZIF-zni (Zn(im)_2_)^36^ at low modifier concentrations (*x* = 0.1, 0.3) or ZIF-7-III (Zn(bim)_2_)^39^ at higher concentrations (*x* = 1.0, 1.5) (Supplementary Figs. 32–39). The absence of the Na(bim) *α* → *β* phase transition and the stable *T*_g_ observed in subsequent heating scans confirm the complete incorporation of the modifier in the glass matrix (Fig. 2f). Increasing Na(bim) content leads to a reduction in *T*_g_, from 294 °C for *g*ZIF-62 down to 161 °C for *g*NaB_1.0_ZIF-62, suggesting progressive depolymerization of the glass network as *x* increases (Fig. 2g). This trend aligns with observations in traditional sodium silicate glasses, where alkali ion incorporation reduces the SiO_2_ glass network connectivity, leading to a decreased *T*_g_ and liquid viscosity^3^. As in sodium silicate systems, *T*_g_ decreases steeply at low modifier concentrations and asymptotically approaches a limiting minimum at higher concentrations (Extended Data Fig. 1d). At the highest Na(bim) contents (*g*NaB_1.5_ZIF-62), the glass transition signal is followed by an exothermic peak at 293 °C, succeeded by an endothermic event of similar magnitude around 407 °C, suggesting intermediate crystallization and dissolution of a transient phase of ZIF-7-III, as supported by VT-PXRD (Supplementary Figs. 37–39).

Further DSC experiments determined the calorimetric fragility indices (*m*) of the modified glasses to evaluate changes in the activation energy of viscous flow at *T*_g_^40,41^ (Supplementary Figs. 46–50). The fragility index differentiates strong glass formers, such as SiO_2_ (*m* = 20), which features a network structure of strongly associated SiO_4_ units, from fragile ones (*m* ≫ 20), such as molecular (for example organic) glass-forming compounds^42^. For *g*ZIF-62, we determined a fragility index of 19 ± 2, classifying it as a strong liquid and aligning with previous reports (*m* ≈ 23)^13,17^. However, as *x* increases, *m* reaches roughly 51 for *g*NaB_1.0_ZIF-62, approaching values typical of molecular glass formers such as glycerol (*m* = 53)^43^. This increase indicates an increasingly complex energy landscape with a greater diversity of configurational states and higher activation energies for viscous flow^42^ (Supplementary Table 6). The associated steeper decline in viscosity with temperature is advantageous for melt processing. In addition, the heat capacity change (Δ*C*_V_) at *T*_g_ increases with higher Na(bim) content, again reflecting a successively larger number of accessible configurational states beyond *T*_g_^42,44^ (Fig. 2g and Supplementary Fig. 52).

The reduced glass stability, as indicated by crystallization events observed during the heating of *g*NaB_*x*_ZIF-62 glasses with *x* ≤ 0.3 and *x* ≥ 1.0, is attributed to reduced Zn–im/bim network connectivity^45^, lower melt viscosity and local excesses of im^−^ and bim^−^ linkers, which encourage partial crystallization from the super-cooled liquid state^46^. Consequently, phase separation, structural rearrangement and nucleation at these modifier concentrations promote the intermediate crystallization of dense, thermodynamically favourable phases—ZIF-zni for *x* ≤ 0.3 and ZIF-7-III for *x* ≥ 1.0—at elevated temperature.

### Local structure analysis

Local structural changes induced by Na(bim)-modification of ZIF-62 glasses were investigated through infrared (IR) spectroscopy, X-ray total scattering combined with pair distribution function (PDF) analysis and solid-state ^23^Na and ^13^C NMR spectroscopy, revealing insights into bond modifications and medium-range order.

**Fig. 3:**
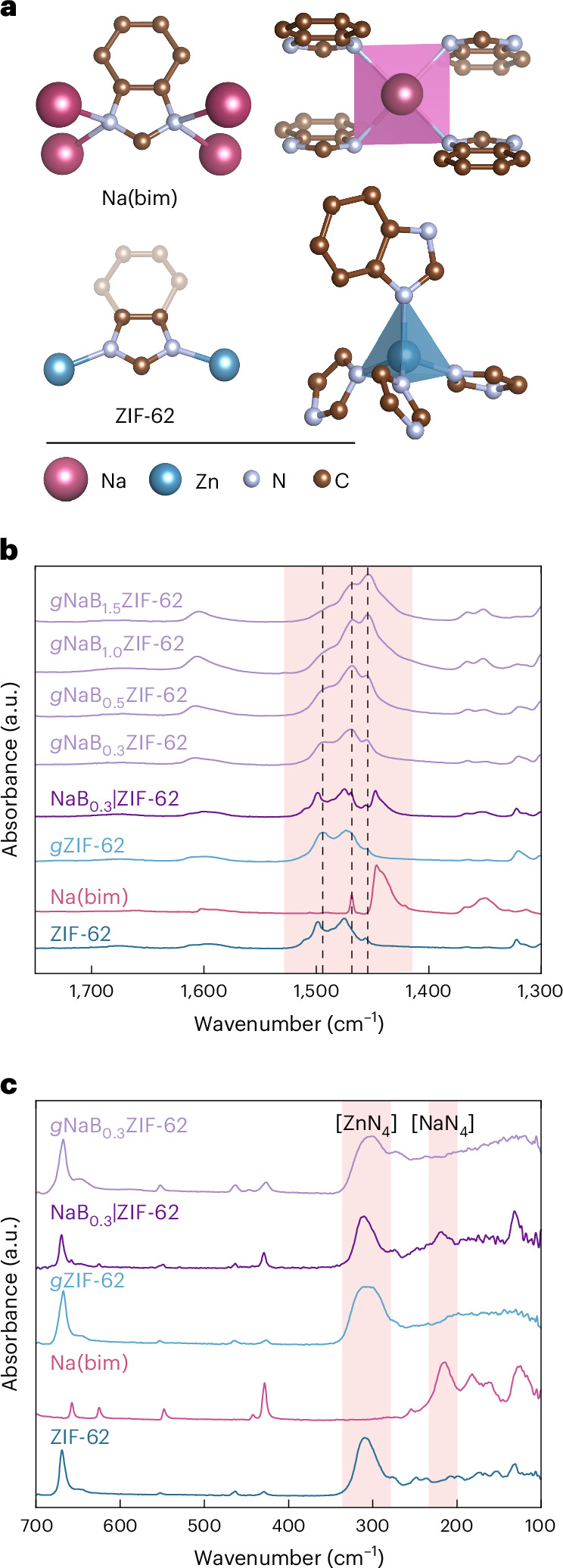
Vibrational spectroscopic analysis of Na(bim) incorporation in ZIF-62 glasses. **a**, Ball-and-stick representation of the local environment surrounding bim^−^/im^−^ linkers and the Na^+^/Zn^2+^ ions in α-Na(bim) (top) and ZIF-62 (bottom). Hydrogens are omitted for clarity. **b**, Excerpt of the mid-IR spectra of ZIF-62, Na(bim), the physical mixture NaB_0.3_|ZIF-62 and *g*NaB_*x*_ZIF-62 glasses with varying *x*. The red-highlighted region corresponds to the bim^−^/im^−^ ring stretching bands, with dashed lines indicating the peak positions in the modified glasses. **c**, Far-IR spectra focusing on the asymmetric [ZnN_4_] stretching band (~309 cm^−1^) and the [NaN_4_] stretching band (~214 cm^−1^).

In the mid-IR spectra (Fig. 3a,b), the distinct vibrational bands of Na(bim) at 1,468 cm^−1^ and 1,445 cm^−1^, identified via density-functional theory (DFT)-based vibrational analysis, correspond to bim^−^ ring stretching modes (Supplementary Fig. 5). For ZIF-62, analogous im^−^/bim^−^ ring stretching modes appear at 1,499 cm^−1^, 1,475 cm^−1^ and 1,456 cm^−1^, which broaden and shift to 1,494 cm^−1^, 1,474 cm^−1^ and 1,455 cm^−1^ in *g*ZIF-62^47^. In the physical mixtures, IR bands from Na(bim) and ZIF-62 are well separated. On vitrification, they merge into three broader bands located at 1,494 cm^−1^, 1,468 cm^−1^ and 1,455 cm^−1^. The intensity ratio of these three peaks varies as *x* increases, with a substantial increase in the band at 1,455 cm^−1^ with concurrent loss of the strong band at 1,494 cm^−1^. These changes are consistent with an increased density of Na^+^-coordinated im^−^/bim^−^, resulting in increased disorder and reduced bond stiffness, leading to band broadening and redshifts.

The far-IR spectra provide further information on metal coordination (Fig. 3a,c). The asymmetric [ZnN_4_] stretching band^48^ at 309 cm^−1^ in crystalline ZIF-62 broadens and shifts to lower wavenumbers in *g*ZIF-62, reflecting distorted Zn^2+^ coordination geometries. The asymmetric [NaN_4_] stretching band of Na(bim) at 214 cm^−1^ is detectable in the spectrum of the physical mixtures but blends into the background after glass formation, confirming the loss of the distinct coordination environment of Na^+^ ions in crystalline Na(bim). On Na(bim) incorporation in the ZIF glass, the [ZnN_4_] stretching band shifts towards even lower wavenumbers, indicating that the modifier weakens the Zn–N bond strength and further disturbs the Zn^2+^ coordination environment.

Complementary insights are obtained from X-ray total scattering experiments. In the scattering function *S*(*Q*), the first sharp diffraction peak (FSDP) shifts from 1.10 Å^−1^ to 1.33 Å^−1^ as *x* increases, suggesting progressive densification of the glass^49,50^ (Fig. 2b). This shift is accompanied by a decrease in scattering intensity and broadening of the FSDP, reflecting reduced electron density contrast and a shorter correlation length on modifier incorporation^49^.

**Fig. 4:**
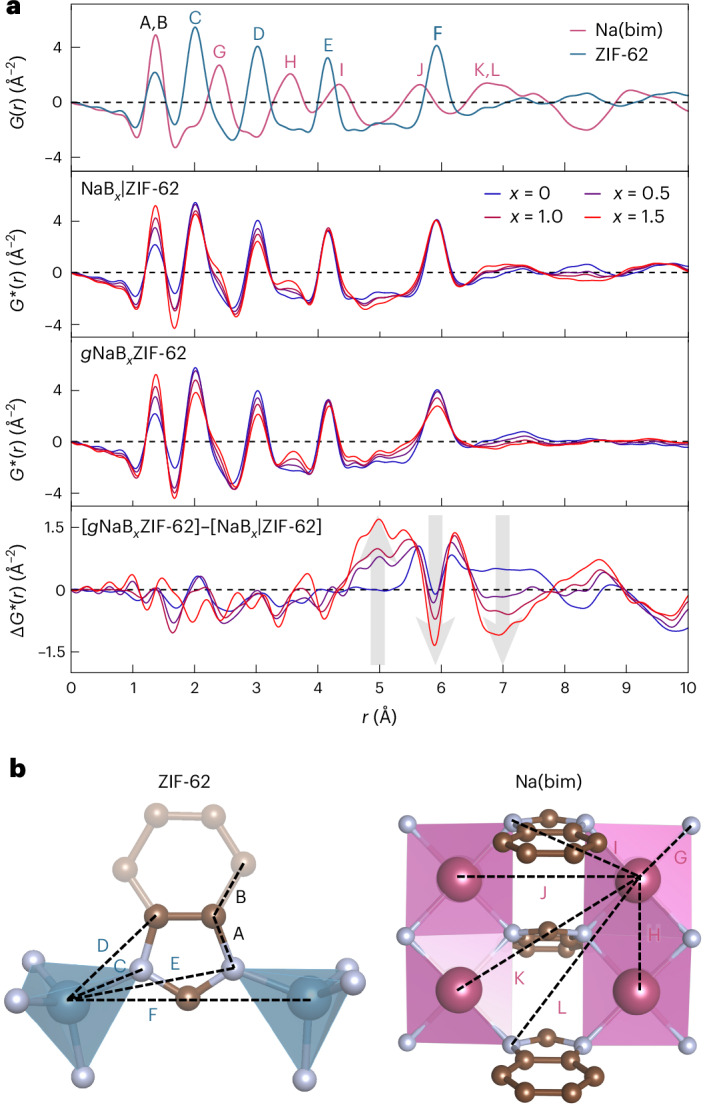
PDF analysis of structural changes during vitrification. **a**, PDFs in the form of *G*(*r*) (scaled by number density) or G*(*r*) (rescaled to the molar unit ZnNa_*x*_(im)_1.8_(bim)_0.2+*x*_) of crystalline Na(bim) and ZIF-62, the crystalline physical mixtures NaB_*x*_|ZIF-62, and the corresponding glasses *g*NaB_*x*_ZIF-62. The differential PDFs (ΔG*(*r*)) illustrate the structural changes between the rescaled PDFs of the physical mixtures and their glasses, highlighting modifications induced by vitrification. **b**, Representation of short-range connectivity of ZIF-62 (left) and α-Na(bim) (right), with key interatomic distances (A–L) highlighted.

To further investigate these structural changes, PDFs, represented as *G*(*r*), were extracted from the X-ray total scattering data for both the NaB_*x*_|ZIF-62 physical mixtures and the corresponding *g*NaB_*x*_ZIF-62 glasses and compared with ZIF-62 and *g*ZIF-62 (Fig. 4a). Owing to the high scattering cross-section of Zn^2+^, Zn-based interatomic distances dominate the PDFs at *r* ≥ 2 Å. The data reveal that, while short-range order, reflecting the immediate environment of Zn^2+^ and the organic linkers, is maintained during melting and glass formation, long-range order (*r* > 10 Å) is lost, consistent with the amorphization process (Supplementary Fig. 65).

To quantify the structural deviations of the Na(bim)-modified glasses from pristine *g*ZIF-62, the *G*(*r*) functions were rescaled to a common molar unit, ZnNa_*x*_(im)_1.8_(bim)_0.2+*x*_ (Fig. 4a and Supporting Information). The rescaled PDFs, G*(*r*), of the ZIF glasses show substantial broadening and reduced intensity of the Zn···Zn next-neighbour peak at 5.9 Å as *x* increases. This observation supports the formation of non-Zn^2+^-bridging im^−^/bim^−^ linkers in Na(bim)-modified glasses, where linkers are attached to one Zn^2+^ and one Na^+^ rather than bridging two Zn^2+^ ions, thereby reducing connectivity in the network.

Differential PDF analysis^51,52^ was performed to isolate changes in interatomic distances on glass modification (Fig. 4a,b). By subtracting the G*(*r*) of physical mixtures from that of their glasses, difference functions ΔG*(*r*) = G*_i_(*r*) − G*_ii_(*r*) were obtained, with *i* = *g*NaB_*x*_ZIF-62 and *ii* = NaB_*x*_|ZIF-62. These difference functions reveal a progressing reduction in the population of ideal Zn···Zn distances (*r* = 5.9 Å) as Na(bim) content increases, along with a gradual shift towards slightly longer Zn···Zn distances. This shift indicates modifier-induced perturbations of Zn^2+^ sites, resulting in a broader distribution of Zn···Zn distances, consistent with the redshift and broadening of the [ZnN_4_] vibrational band (Fig. 3c). In addition, the loss of pair correlations around 7 Å is largely attributed to the loss of Na···Na distances specific to crystalline Na(bim), while new Na···Zn distances may be evident in the range from 4.5 Å to 5.5 Å, as this region is increasingly populated in the *G*(*r*) of the glasses with higher *x*.

Further extended X-ray absorption fine structure measurements on the Zn *K*-edge of ZIF-62, *g*ZIF-62 and *g*NaB_*x*_ZIF-62 materials provide a coordination number of 4 across all samples, confirming that tetrahedral Zn^2+^ network nodes are largely preserved during glass formation and modification (Supplementary Table 11).

**Fig. 5:**
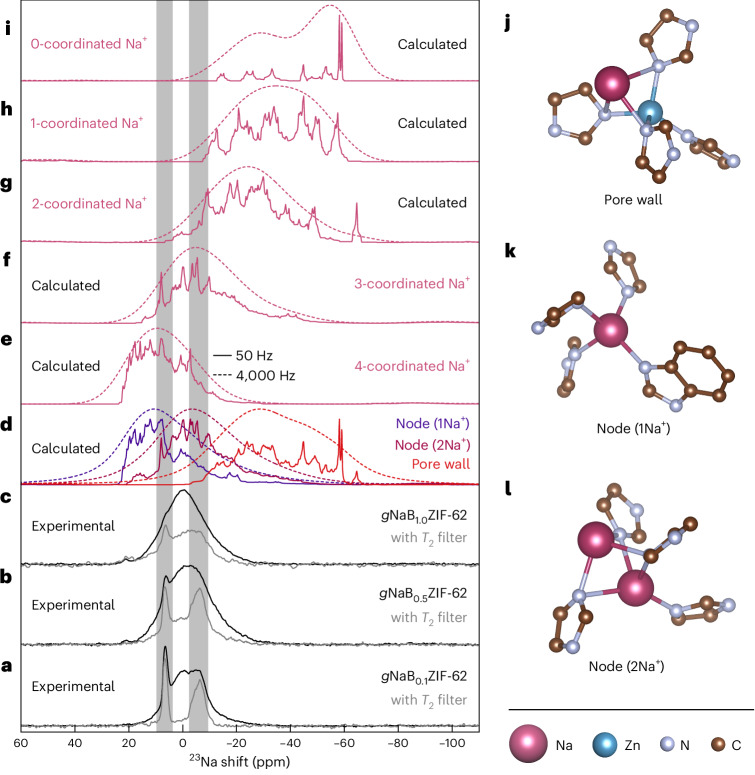
NMR and DFT analysis of structural changes during vitrification. **a**–**c**, Experimental ^23^Na NMR (20.0 T, 20 kHz MAS) spectra of *g*NaB_*x*_ZIF-62 with *x* = 0.1 (**a**), 0.5 (**b**) and 1.0 (**c**). Spectra acquired with both a short (0.1 ms, black) and long (25.6 ms, grey) echo delay are shown, with the latter effectively filtering components in the spectra with short spin–spin relaxation times (*T*_2_). **d**–**i**, Predicted ^23^Na NMR spectra of Na^+^ sites in DFT-simulated *g*NaB_*x*_ZIF-62 structures, categorized by Na^+^ occupation in the *g*NaB_*x*_ZIF-62 structure (1Na^+^ and 2Na^+^ correspond to singly and doubly occupied node sites, respectively) (**d**) and N–Na^+^ coordination number (**e**–**i**). **j**–**l**, Examples of local structures surrounding Na^+^ in DFT-simulated *g*NaB_*x*_ZIF-62: a MOF pore-wall site (**j**), a singly occupied node site (**k**) and a doubly occupied node site (**l**). Gaussian line broadening of 50 Hz (solid) and 4,000 Hz (dashed) is applied to calculated spectra to simulate the effect of static disorder.

To further investigate the local structure of *g*NaB_*x*_ZIF-62 materials, we used a combined theoretical and experimental approach using solid-state ^23^Na magic-angle spinning (MAS) NMR spectroscopy: a powerful technique for probing atomic-level structures, even in highly disordered solids. Figure 5a–c shows ^23^Na MAS NMR spectra of *g*NaB_*x*_ZIF-62 with *x* = 0.1, 0.5 and 1.0 at 20.0 T (MAS = 20 kHz).

At *x* = 0.1, three signals are observed: two narrow peaks at 6.8 ppm and −6.3 ppm, and a broad peak centred around 0 ppm. The large full-width at half-maximum (13.9 ppm) of this signal indicates pronounced static disorder in the Na^+^ local environment. As the Na(bim) content increases, the intensity of the broad component grows, dominating other spectral features. To further interrogate these overlapping components, we used a longer echo delay during acquisition of ^23^Na spectra, effectively suppressing signals with short spin–spin relaxation times (*T*_2_)^53^. This filtering reveals the same two narrow components in *g*NaB_0.5_ZIF-62 and *g*NaB_1.0_ZIF-62 as in *g*NaB_0.1_ZIF-62 that are obscured by the broad signal in the unfiltered spectra (Fig. 5a–c and Supplementary Fig. 94). This suggests that, at low *x*, Na^+^ predominantly occupies two well-defined coordination sites corresponding to the narrow peaks. However, the presence of the broad signal even at *x* = 0.1, and the disappearance of narrow peaks at higher *x*, indicate that these preferred coordination sites support limited Na^+^ occupancy (*x* < 0.1), while excess Na^+^ populates a diverse distribution of local coordination environments.

To structurally identify the Na^+^ coordination modes observed experimentally, we generated possible Na^+^ environments using ab initio random structure searching^54^. To simulate a wide range of Na^+^ coordination environments, we geometry optimized supercells of (Na_2*y*_Zn_16-*y*_)((im)_1.75_(bim)_0.25_)_16_ for integer *y* = 1–4, where *y* = 2 corresponds to a Na^+^:Zn^2+^ ratio consistent with *x* = 0.29. Structures were relaxed to minima in the potential energy surface by minimizing DFT forces. For each *y*, ab initio random structure searching yielded 10 distinct, local energy-minimized structures, resulting in 200 Na^+^ environments (Supplementary Fig. 88). The corresponding solid-state ^23^Na NMR spectra (20.0 T, 20 kHz) were simulated using calculated chemical shielding and electric-field gradient tensors (Supplementary Fig. 95).

As expected, DFT-relaxed structures reveal diverse Na^+^ environments, ranging from Na^+^ incorporation at node sites (that is, sites similar to Zn^2+^ sites in ZIF-62) to Na^+^ attachment on the framework’s pore walls. The degree of Na^+^ coordination by im^−^/bim^−^ nitrogen lone pairs spans coordination numbers from 0 to 4 (Supplementary Fig. 89), with broad distributions in Na–N distances and N–Na–N angles (Supplementary Fig. 90). To investigate the correlation between ^23^Na NMR shift and both Na^+^ positioning and N–Na^+^ coordination, all 200 calculated Na^+^ sites were classified by their coordination number and grouped to generate cumulative predicted ^23^Na spectra (Fig. 5d–i, 50 Hz and 4,000 Hz Gaussian broadening was applied to simulate broadening due to static disorder).

As a result, three distinct Na^+^ site categories emerge: (1) Na^+^ attached to the pore walls (Fig. 5j), (2) Na^+^ replacing a tetrahedral Zn^2+^ node (Fig. 5k) and (3) two Na^+^ jointly replacing a tetrahedral Zn^2+^ node (Fig. 5l). Grouping predicted ^23^Na spectra corresponding to Na^+^ sites in these three categories (Fig. 5d) reveals minimal overlap between experimental data and shifts predicted for pore-wall sites, indicating that Na^+^ preferentially adopts coordination sites similar to those of tetrahedral Zn^2+^ nodes, with doubly occupied sites constituting the dominant population and singly occupied sites representing a smaller population. This preference is probably caused by short, energetically unfavourable Na^+^···Zn^2+^ distances associated with *π*-coordination at pore walls (mean 3.49 Å across calculated pore-wall sites).

Further grouping of the simulated spectra by N–Na^+^ coordination number (Fig. 5d–i and Supplementary Table 14) reveals a strong correlation between ^23^Na chemical shift and coordination, with minimal overlap between predicted ^23^Na shifts for 2-coordinated, 1-coordinated and 0-coordinated Na^+^ and the experimental spectral region. The strongest correlation is seen with 3-coordinated Na^+^, confirming that Na^+^ substitution for Zn^2+^ distorts the network and reduces connectivity. Supporting this, we find a close correlation between the Na^+^ coordination number and Na^+^ site assignment (Supplementary Fig. 89), with mean coordination numbers of 1.35 (pore wall), 3.17 (doubly occupied nodes) and 3.60 (singly occupied nodes).

Singly occupied Na^+^ node sites carry a formal −1 framework charge, whereas doubly occupied sites are charge neutral. Because ^23^Na MAS NMR indicates only a minor population of pore-wall Na^+^ sites, charge compensation for singly occupied node sites must predominantly arise from local framework defects, most plausibly missing-linker defects. This interpretation is consistent with the DFT-derived diversity of Na^+^ environments and with the broad distribution of N–Na^+^ bond lengths and angles (Supplementary Fig. 90).

While overlapping predicted ^23^Na line shapes prevent definitive assignment of the narrow, low-intensity experimental signals at 6.8 ppm and −6.3 ppm, simulated spectra suggest these signals arise from singly or doubly occupied node sites (Supplementary Fig. 91). In addition, the cumulative predicted spectrum of doubly occupied node sites centres around the broad experimental peak at ~0 ppm (Fig. 5d). Collectively, the simulated spectra suggest that at low *x*, Na^+^ resides in coordination environments similar to those of distorted Zn^2+^ sites; as *x* increases, doubly occupied Na^+^ node sites become more populated, which gradually increases the intensity of the broad spectral component centred at ~0 ppm, reflecting growing structural disorder.

These structural insights reveal a fundamental contrast between Na-modified ZIF glasses and sodium silicate glasses. In silicates, Na^+^ is typically coordinated by 5–6 oxide anions and is seen to form percolation channels through the glass network^55,56^. In Na-modified ZIF glasses, however, the coordination number is only 3–4, with Na^+^ coordinated by im^−^/bim^−^ nitrogen lone pairs. Although Na^+^ may singly or doubly occupy tetrahedral-like positions reminiscent of Zn^2+^, the Na–N interaction is far weaker than the Zn–N bonds defining the ZIF network. Consequently, Zn^2+^ substitution by Na^+^ weakens the coordination network by creating non-Zn^2+^-bridging imidazolate-type linkers. The distinct geometric constraints of the im^−^ and bim^−^ linkers further reinforce this effect: unlike compact, spherical oxide anions in silicate glasses, the extended organic imidazolate linkers introduce steric hindrance, limiting the number of nitrogen atoms that can coordinate to Na^+^. Furthermore, the directional character of the nitrogen lone pairs favours fewer and more specific Na^+^···N contacts compared with the largely non-directional ionic Na^+^···O interactions in silicates. This distinction highlights the unique structural role of Na^+^ in ZIF glasses and underscores the potential of adapting glass modification principles to MOF-based systems to achieve structural and functional outcomes fundamentally different from those of inorganic glasses.

### Modifier extraction and gas sorption

**Fig. 6:**
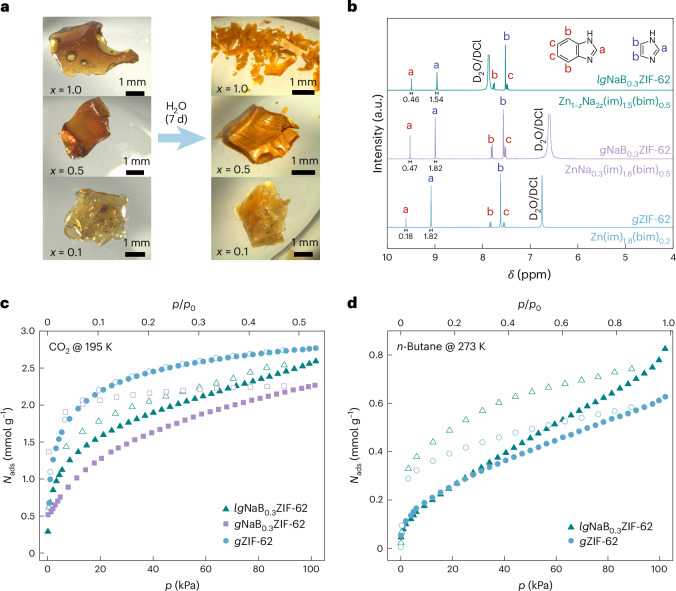
The influence of water leaching on the material’s porosity. **a**, Optical microscopy images of *g*NaB_*x*_ZIF-62 glasses before and after suspension in water for 7 days. **b**, ^1^H solution NMR spectra of the modified glass before and after water leaching, digested in D_2_O/DCl and DMSO-*d*_6_. The chemical shift of the protons of the solvent D_2_O/DCl is strongly pH dependent, leading to variations in their positions across the spectra. ^1^H NMR signals (**a**–**c**) are assigned to protons in Him (blue letters) and Hbim (red letters), respectively. **c**, Sorption isotherms for CO_2_ collected at 195 K. **d**, *n*-Butane sorption isotherms collected at 273 K. Points of the adsorption and desorption branches are shown as solid and open symbols, respectively.

The porosity of ZIF glasses is typically much lower than that of their crystalline parent frameworks, prompting recent efforts to enhance and control the glasses’ porosity for applications in hydrocarbon separation and carbon capture^12,18,57^. Here Na(bim)-modified ZIF glasses were subjected to a water-extraction process to investigate porosity evolution through selective modifier leaching. On submersion in water, the Na–N bonds undergo hydrolysis, releasing NaOH and imH/bimH from *g*NaB_*x*_ZIF-62, generating a strongly basic solution (Supplementary Fig. 100), while largely preserving the Zn–im/bim network. Optical microscopy reveals progressive changes in the glass shards over several days (Fig. 6a and Supplementary Fig. 99). The initially transparent glass pieces become opaque and swell once in contact with water. After 7 days, samples with low Na(bim) content (*x* = 0.1) largely retain their structure, whereas higher modifier contents lead to lamellar cracking (*x* = 0.5) or fragmentation (*x* = 1.0). This increasing water sensitivity with *x* reflects the growing fraction of hydrophilic modifier relative to the water-stable ZIF glass former^14^.

Quantitative ^1^H solution NMR spectroscopy of acid-digested, water-leached *g*NaB_0.3_ZIF-62 (denoted *lg*NaB_*x*_ZIF-62, *lg* = leached glass) demonstrates preferential removal of Na(im) over Na(bim), ascribed to the greater steric bulk of bim^−^ relative to im^−^ and indirectly supporting the presence of Na–im bonds in the modified glasses (Fig. 6b and Supplementary Fig. 103). The washing solution contains about 95% imidazole and 5% benzimidazole (Supplementary Fig. 104). Despite leaching, all samples remain amorphous, as confirmed by PXRD (Supplementary Figs. 101 and 102). Complementary ^23^Na spin-counting experiments show that roughly one-third of the initial Na^+^ content is removed during leaching (Supplementary Fig. 111), consistent with many Na^+^ environments of differing hydrolytic stability. Far-IR spectroscopy shows a slight blueshift of the [ZnN_4_] stretching band in *lg*NaB_0.3_ZIF-62, indicating strengthened Zn–N interactions postleaching (Supplementary Fig. 106). DSC of *lg*NaB_0.3_ZIF-62 shows several broad endothermic events on first heating (Supplementary Fig. 110); after annealing at 450 °C for 30 min, *T*_g_ increases from 117 °C to 275 °C, suggesting partial network repolymerization on annealing.

This extraction process parallels the well-established Vycor process, developed in the 1930s to produce porous silicate glasses for applications in catalysis, adsorption and membrane technologies^34,35^. In the Vycor process, selective acid leaching of phase-separated alkali borosilicate glasses generates controlled porosity^58^. Analogously, modifier removal from *g*NaB_*x*_ZIF-62 provides a pathway for creating more porosity in MOF-derived glasses.

Porosity changes were quantified by CO_2_ sorption at 195 K for *g*ZIF-62, *g*NaB_*x*_ZIF-62 and *lg*NaB_*x*_ZIF-62 (Fig. 6c). CO_2_ is an established probe for quantifying the microporosity of ZIF glasses, as N_2_ access is typically hindered at 77 K. At 195 K, the CO_2_ sorption isotherm reaches complete micropore filling close to 100 kPa^59^. The parent glass *g*ZIF-62 shows a Type I isotherm with a maximum CO_2_ uptake of 2.77 mmol g^−1^, consistent with literature^12,17^. By contrast, *g*NaB_0.3_ZIF-62 shows a lower uptake (2.27 mmol g^−1^) and pronounced hysteresis, aligning with glass densification inferred from the FSDP analysis (Fig. 2b) and reduced accessible microporosity due to modifier incorporation.

After modifier extraction, the resulting *lg*NaB_*x*_ZIF-62 exhibits an increased CO_2_ uptake of 2.59 mmol g^−1^, approaching that of *g*ZIF-62, along with substantially reduced hysteresis, indicating improved pore accessibility. The slightly lower uptake and residual hysteresis of *lg*NaB_0.3_ZIF-62 compared with *g*ZIF-62 are attributed to the higher fraction of the bulky bim^−^ linkers in the leached glass. Given the maximum relative pressure (*p*/*p*_0_) for CO_2_ sorption at 195 K is about 0.55 (refs. ^60–62^), these measurements are limited to evaluating microporosity (Supplementary Fig. 115) without access to mesopore or macropore volume. However, fitting the CO_2_ sorption data to a dual-site Langmuir model, followed by extrapolation to a hypothetical CO_2_ saturation pressure, suggests that *lg*NaB_0.3_ZIF-62 possesses a total pore volume ~26% greater than that of *g*ZIF-62 (Supplementary Fig. 112 and Supplementary Table 15). This finding is corroborated by *n*-butane sorption at 273 K, which shows a 26% higher *n*-butane capacity of *lg*NaB_0.3_ZIF-62 compared with *g*ZIF-62 at saturation, evidencing further mesoporosity and macroporosity generated on leaching (Fig. 6d).

Together, these results demonstrate that selective modifier extraction enables recovery of microporosity and generation of mesoporosity or macroporosity, providing a versatile strategy for tuning MOF glass porosity.

### ZIF glass modification with Li(bim)

Finally, to probe the generality of alkali-benzimidazolate glass modification, we investigated Li(bim) as an alternative modifier^33^. Melt-quenching of LiB_*x*_|ZIF-62 likewise yields homogeneous glasses exhibiting a modifier-dependent decrease in *T*_g_ and signatures of network depolymerization, closely paralleling the behaviour observed for Na(bim)-modified systems (Extended Data Fig. 1). Notably, the *x*-dependent relative decrease in *T*_g_ of the alkali-modified ZIF glasses remains substantially smaller than that observed for Na_2_O-modified silicate glasses^63^, underscoring the fundamentally different structural roles of alkali oxides and alkali benzimidazolates as glass modifiers. The slightly higher *T*_g_ values of Li(bim)-modified glasses compared with their sodium analogues are rationalized by the smaller ionic radius of Li^+^ and correspondingly stronger Li–N interactions^33^. Full experimental and structural characterization of Li(bim)-modified ZIF-62 glasses is provided in the Extended Data and Supplementary Information. Together, these results demonstrate that the modifier concept is governed by network chemistry rather than a specific alkali species.

## Conclusion

The vitrification of the MOF glass former ZIF-62 with Na(bim) by means of a flux-melting approach has successfully yielded modified ZIF glasses, conceptually analogous to traditional (silicate) glass modification systems. Key properties, including a modifier-induced decrease in *T*_g_, increased hydrophilicity and enhanced liquid fragility, were shown to be tunable in a controlled, continuous manner. These trends correspond to progressive network depolymerization and increased structural disorder, mirroring mechanisms observed in inorganic glasses. Combined ^23^Na MAS NMR spectroscopy and DFT calculations provide key insights into the local Na^+^ environments. Unlike in silicate glasses, Na^+^ primarily integrates into the ZIF glass network by replacing Zn^2+^ nodes, forming highly disordered and undercoordinated Na^+^ environments. This substitution reduces Zn–im/bim–Zn network connectivity and partially collapses the pore structure compared with *g*ZIF-62, underscoring the profound impact of modifiers on the structural and functional properties of MOF glasses.

Beyond these valuable insights, the modified ZIF-62 glasses retain a substantial fraction of their intrinsic microporosity, in contrast to earlier and most recent approaches to ZIF glass modification, where glass modification effectively eliminates residual microporosity^23,32^. In addition, the postsynthetic removal of the Na-based modifier via water extraction highlights the adaptability of leaching processes to MOF glasses. This enables recovery and further tuning of porosity, expanding the functional capabilities of MOF glasses for gas separation, storage and catalysis. Extension of the strategy to Li(bim) demonstrates its broad applicability and robustness, with thermal and structural trends closely paralleling those observed for the sodium analogues.

More broadly, this work establishes a transferable framework for non-stoichiometric modification of MOF glasses, expanding their chemical and functional diversity beyond the constraints of glass formation alone. While the number of meltable MOFs remains limited, the modifier framework demonstrated here provides a scalable strategy for diversifying the properties of existing MOF glass formers. The structure–property relationships identified here translate into practical design rules, linking modifier content and identity to *T*_g_, viscosity, crystallization tendency and hierarchical porosity, advancing MOF glass chemistry from empirical discovery towards rational design and positioning MOF glasses as a versatile platform for next-generation porous and functional materials. Although the adjustable *T*_g_ and improved flowability resulting from reduced viscosity may facilitate processing at lower temperatures, practical manufacturing can be constrained by the requirement of autogenous pressure during melt-quenching and the strong adhesion of the modified MOF glasses to crucible surfaces, which currently hinder the production of large glass monoliths. Nevertheless, the demonstrated flux modification strategy may enable the transformation of previously non-meltable ZIFs into processable glasses, expanding the landscape of accessible MOF glass materials. Looking forward, this framework motivates targeted optimization of stability and processing (for example, suppressing crystallization through balanced modifier content and cation choice), and extension to property spaces not addressed here, including mechanical performance, transport (ionic or electronic) and adsorption selectivity, where modifier chemistry and leaching protocols provide clear experimental levers.

## Methods

All chemicals and solvents used in the synthesis were purchased from commercial suppliers and used without further purification if not stated otherwise.

### Synthesis of ZIF-62 (Zn(im)_1.8_(bim)_0.2_)

Here 2.4 g of Zn(NO_3_)_2_·6 H_2_O (8.1 mmol, 8.1 eq.), 122 mg benzimidazole (1.0 mmol, 1.0 eq.) and 1.727 g imidazole (25.4 mmol, 25.4 eq.) were dissolved in 14 ml of *N*,*N*-dimethylformamide in a Teflon lined autoclave (25 ml). The autoclave was sealed tightly, transferred to a preheated oven at 130 °C and heated for 45 h. After cooling to room temperature, the colourless crystals were washed with iso-propanol (3 × 10 ml) and ground thoroughly. The powder was thermally activated under a dynamic vacuum (10^−3^ mbar) at 280 °C for 4 h in a tube furnace. Zn(im)_1.8_(bim)_0.2_ was obtained as a colourless powder (yield 511 mg, 1.8 mmol, 36%).

### Synthesis of *α*-Na(bim)

Na(bim) was synthesized according to a synthetic protocol reported in the literature^33^.

Before the synthesis, benzimidazole (bimH) was dried by resublimation at 100 °C under a dynamic vacuum (10^−3^ mbar) overnight. In a purged Schlenk flask (250 ml), 1.5 g (12.7 mmol, 1.1 eq.) of dry bimH was dissolved in 30 ml of dry tetrahydrofuran under constant argon flow. Then 6 ml (12.0 mmol, 1.0 eq.) of sodium hexamethyldisilazane (2 M in tetrahydrofuran) was added while the solution was cooled with an ice bath. Within 1 h, the reaction mixture was allowed to warm up to room temperature under constant stirring. Next, the yellow reaction mixture was filtered through a syringe filter (polytetrafluoroethylene, 0.2 μm) and transferred into another purged Schlenk flask (250 ml). After that, 40 ml of dry *n*-hexane was added to initiate the formation of a white precipitate. The reaction mixture was stored in a refrigerator (8 °C) overnight. The next day, the mixture was decanted and the obtained white powder was washed with dry *n*-hexane (2 × 20 ml). The residual solvent was removed under dynamic vacuum (10^−3^ mbar) at 100 °C for 2 h and the solid material was then heated at 280 °C in a tube furnace for 3 h under a dynamic vacuum (10^−3^ mbar) to remove any excess bimH. *α*-Na(bim) was obtained as a light-yellow powder and stored in a glovebox under an inert N_2_ atmosphere (yield 1.43 g, 10.2 mmol, 85%).

### Preparation of *g*NaB_*x*_ZIF-62 glasses (*x* = 0.0, 0.1, 0.3, 0.5, 1.0, 1.5)

Na(bim) modified glasses were prepared by mixing the corresponding amount of the glass former ZIF-62 with the modifier Na(bim) in an N_2_-filled glovebox (Extended Data Table 1). The physical mixture was ground thoroughly for 5 min to ensure sufficient homogenization after being transferred to either a hermetically sealed DSC crucible (10 mg scale) or a custom-made hermetically sealed quartz glass lined stainless-steel crucible (250 mg scale). The small-scale glass preparation (*x* = 0.1, 0.3, 0.5, 1.0, 1.5) temperature program was applied in a DSC apparatus. It consisted of an initial heating step from room temperature to a maximum temperature (450 °C) followed by an isothermal segment of 30 min and a final cooling step back to room temperature. All heating and cooling rates were executed at a constant rate of ±10 °C min^−1^.

For glass preparation on a larger scale (*x* = 0.1, 0.5, 1.0), the stainless-steel crucible equipped with a quartz glass inlet was filled with 250 mg of the corresponding physical mixture, sealed tightly under an N_2_ atmosphere (glovebox) and transferred into a purged Schlenk tube that was introduced to a tube furnace (autoclave approach). For the autoclave approach, the isothermal segment in the aforementioned temperature program was extended to 1 h to allow for complete mixing in the liquid state. Finally, the Schlenk tube was removed from the furnace and cooled to room temperature within 40 min using pressurized airflow on the outside of the Schlenk tube as a cooling agent. Further handling of the obtained glasses was performed in a glovebox under an N_2_ atmosphere. In the case of *x* = 0 (preparation of *g*ZIF-62) the autoclave approach was chosen. In the modified temperature program, the maximum temperature was increased to 470 °C, followed by an isothermal segment (1 h) and final cooling back to room temperature according to the procedure mentioned above.

### Preparation of *lg*NaB_0.3_ZIF-62

The modified glass *g*NaB_0.3_ZIF-62 (250 mg) was prepared in an autoclave approach as described above. After vitrification, the material was ground thoroughly for 5 min and subsequently dispersed in distilled water (25 ml). The water was exchanged 5 times over the course of 24 h, and the suspension was stirred for the next 2 days. After that time, the mixture was decanted, and the material was washed with MeOH (3 × 25 ml). The remaining solid material was transferred to a Schlenk tube and heated at 100 °C under a dynamic vacuum overnight to remove any remaining solvent residues. The washing solutions were collected, and the bulk solvent was removed via rotary evaporation at 60 °C.

### PXRD

Room temperature PXRD measurements were conducted on a Siemens D5005 diffractometer in Bragg-Brentano geometry using CuKα radiation in the range from 5° to 50° 2*θ* with a step size of 0.01°. The sample material was ground using *n*-hexane and the resulting suspension was deposited on a single crystal zero background sample holder made from silicon (cut along the (610) plane).

VT-PXRD measurements were performed at Beamline BL9 at DELTA (Dortmunder Elektronenspeicherring Anlage) with a monochromatic X-ray beam (*λ* = 0.4568 Å) using a MAR345 plate detector in an angular range from roughly 2–12° 2*θ*. The thoroughly ground sample material was filled in a borosilicate capillary (1.0 mm diameter) under an inert N_2_ atmosphere (glovebox) and sealed using epoxy glue (UHU Schnellfest). An Anton Parr DHS1100 sample stage was used for the temperature adjustment. The temperature program consisted of a stepwise temperature increase from room temperature to the respective maximum temperature using a constant heating rate of roughly 30 °C min^−1^. The heating ramp was interrupted by isothermal segments at the respective temperature steps of 120 s for data collection. Data integration and processing were performed in DAWN Science version 2.30.0^64,65^. Structureless profile fits (Pawley method^66^) were performed using the routines provided by the TOPAS-academic v.6 software package^67^.

### Variable-temperature X-ray total scattering

In situ variable-temperature X-ray total scattering experiments were conducted at beamline I15-1 at Diamond Light Source (DLS) or at Beamline P02.1 at Deutsches Elektronen Synchrotron (DESY). At DLS a monochromatic X-ray beam (*λ* = 0.1617 Å) was used for data collection in combination with a Perkin Elmer XRD 4343CT 2D plate detector. Under an inert N_2_ atmosphere (glovebox), the finely ground sample material was filled in a borosilicate glass capillary (1.0 mm outer diameter) and sealed using epoxy glue (UHU Schnellfest). A hot air blower was used to adjust the sample temperature stepwise up to 450 °C with a constant heating rate of 20 °C min^−1^ followed by an isothermal segment of 5 min for data collection and subsequent cooling back to room temperature. PDFgetX3 version 2.2.0^68^ was used for further processing (calculation of *I*(*Q*), *S*(*Q*) data) and the calculation of PDF data in the form of *G*(*r*). For the differential PDF approach, the total scattering data were evaluated using Gudrun (version 2017). The subsequent data manipulation (LC-fitting approach) was performed with algorithms provided by the NumPy (version 1.21.5) and SciPy (version 1.9.1) libraries in Python v.3.9.13. For NaB_0.3_|ZIF-62 in situ variable-temperature X-ray total scattering measurements were performed at DESY with a wavelength of 0.2071 Å in combination with a Varex XRD 4343CT plate detector and an Oxford Instruments hot air blower for temperature adjustment applying the same temperature program as at DLS. The measurements were collected using borosilicate capillaries (1 mm outer diameter), which were filled with the finely ground sample material and sealed under an inert N_2_ atmosphere (glovebox). In addition, a room temperature measurement of ex situ prepared finely ground *g*NaB_0.3_ZIF-62 material was conducted. Sample preparation, filling of the capillaries and sealing with glue (UHU Schnellfest) was performed under an inert N_2_ atmosphere (glovebox). Data integration and processing were performed in DAWN Science version 2.30.0 and PDFgetX3^64,65^.

### Simultaneous thermogravimetric and differential thermal analysis

Simultaneous thermal analysis measurements were performed on a Discovery SDT 650 instrument from TA Instruments under a constant nitrogen flow of 100 ml min^−1^. For the measurement, a small amount (~10 mg) of the powdered sample was placed in an open alumina crucible under an inert (N_2_) atmosphere. The sample crucible was removed from the inert atmosphere and quickly transferred into the simultaneous thermal analysis instrument, where it was heated with a constant heating rate of 10 °C min^−1^ in a range from 50 °C to ~600 °C under constant N_2_ flow. Data evaluation was carried out in TA Instruments TRIOS version 5.1.1.46572. Decomposition temperatures *T*_d_ were determined as the onset of mass loss in the thermogravimetric curve.

### DSC

DSC measurements were performed on a DSC25 from TA Instruments. The samples were ground thoroughly and placed in a hermetically sealed aluminium crucible under an inert N_2_ atmosphere. All experiments were conducted under constant N_2_ flow (50 ml min^−1^) in a temperature range from 50 °C to roughly 500 °C. Unless stated differently, a constant heating and cooling rate of ±10 °C min^−1^ was applied. Temperature and enthalpy calibration was performed with different metal standards (indium, zinc, lead) using the same temperature program. Data evaluation was carried out in TA Instruments TRIOS version 5.1.1.46572. The integrals of the heat flow curves are given as enthalpies Δ*H* even though the measurements were performed under hermetic conditions (*V* = constant) since it is generally assumed that enthalpies Δ*H* and internal energies Δ*U* are similar for processes involving only condensed phases^69^. Modulated DSC measurements for Δ*C*_*V*_ calculation were carried out with a heating rate of 2 °C min^−1^, a modulation period of 120 s and an amplitude of ±1 °C min^−1^.

### Solution ^1^H NMR spectroscopy

Solution ^1^H NMR were performed on a Bruker DPX-300, DPX 500 or Agilent DD2 500 spectrometer. The crystalline material was dissolved in dimethylsulfoxide (DMSO)-*d*_6_ (0.5 ml) and DCl/D_2_O (35 wt%, 1 drop, <0.1 ml). Data processing was performed in MestReNove (v.14.2.0). The data were referenced to the residual proton signals of DMSO, and chemical shifts were given relative to tetramethylsilane.

### Fourier transform IR spectroscopy

Far-IR and mid-IR spectra were collected on a Spectrum 3 Fourier transform IR spectrometer in a range from 4,000 cm^−1^ to 400 cm^−1^ (mid IR) or 30 cm^−1^ to 700 cm^−1^ (far IR), respectively. A Gladi ATR-300 unit from Pike Technologies was used as an attenuated total reflectance unit. During the measurement, all samples were compressed with a stamp securing the sample on the ATR diamond and an inert-gas (N_2_) flow was put in place to minimize the sample’s reaction with atmospheric water.

### Isothermal gas physisorption

Gas sorption isotherms were collected in a Quantachrome iQ MP porosimeter. The ground sample material (50–90 mg) was degassed at 100 °C for roughly 2 h under dynamic vacuum (*p* ≈ 10^−5^ kPa) before the measurement. Isothermal physisorption measurements were conducted by using N_2_ (77 K, purity >99.999%), CO_2_ (195 purity >99.995%) and *n*-butane (273 K, purity >99.9%) as gas probes. The temperature was adjusted using a 3P Instrument CryoTune filled with liquid nitrogen. Between two measurements, the samples were degassed under dynamic vacuum (*p* ≈ 10^−5^ kPa) at ambient temperature for roughly 2 h. After the sorption measurements with *n*-butane, the sample was also degassed under a dynamic vacuum at 100 °C for 30 min.

### Electron microscopy

The STEM/EDS investigations were performed on an aberration-corrected JEOL JEM-ARM200F NEOARM. Scanning electron microscopy (SEM) images were taken with the FEI G4 CX focused ion beam (FIB)/SEM. For the preparation of SEM and TEM samples, a small amount of the particles was dispersed in hexane by ultrasonication and then dropped onto Cu TEM grids. To prevent the particles from reacting with humidity, this process was carried out in a Sylatech GB1500-E glovebox. The individual TEM grids were then sealed airtight in plastic bags in the glovebox to protect them from exposure to humidity during transportation to the TEM.

### APT

To prepare the APT samples, the particles were placed on aluminium sample holders in a Sylatech GB1500-E glovebox at a humidity of <1 ppm. Individual particles were then picked up with a micromanipulator in a FEI G4 CX focused FIB/SEM, welded to a microtip coupon and subsequently shaped into a cone-shaped specimen (size 50 × 50 × 100 nm) using a standard annular milling procedure. To prevent the hygroscopic particles from reacting with the air humidity between the preparation steps, a Ferrovac Ultra-High Vacuum Cryo Transfer Suitcase was used to transfer the samples between the glovebox, FIB/SEM and APT in an ultra-high vacuum. The APT measurements were performed in a Cameca LEAP 5000 XR operating in laser mode at a temperature of 60 K, a pulse frequency of 125 kHz, a laser pulse energy of 20 pJ and a detection rate of 0.4%. The APT data were reconstructed and analysed with the commercial software AP Suite v.6.3.0.90.

### Solid-state NMR spectroscopy

The powdered *g*NaB_*x*_ZIF-62 samples were packed into 3.2-mm zirconia rotors (room temperature ^23^Na measurements) or zirconia crucible inserts then placed inside 7-mm zirconia rotors (variable-temperature ^23^Na measurements). Solid-state MAS NMR spectra of ^23^Na (224.8 MHz) were recorded on a Bruker Avance Neo 20.0 T spectrometer equipped with a 3.2-mm MAS probe using 62.5 kHz radio frequency field. A MAS rate of 20 kHz was used for all such samples. Variable-temperature ^23^Na MAS spectra were recorded on the same spectrometer but equipped with a 7-mm laser-heated MAS probe and using 250-kHz radio frequency field. The temperature of samples in this probe was calibrated using the ^79^Br shift variation of KBr^70^. A MAS rate of 4 kHz was used for all such samples. ^23^Na chemical shifts were referenced to NaCl (7.71 ppm). Quantitative ^23^Na spectra were recorded using a recycle delay of 5× measured *T*_*1*_ component for each sample. All rotors were spun using dry nitrogen.

### DFT calculations

The magnetic shielding and electric-field gradient tensors were calculated using CASTEP (version 23.1)^71^ sing density-functional perturbation theory and the gauge-including projector augmented wave method^72–75^. The Perdew–Burke–Ernzerhof functional and on-the-fly ultrasoft pseudopotentials were used^76^. A cut-off energy of 900 eV was used for the plane-wave basis set. The convergence criteria for electronic self-consistency was 10^−10^ eV. A Monkhorst–Pack grid with *k*-point spacing of 0.03 Å^−1^ was used for Brillouin-zone integration^77^. Structures were relaxed to a force threshold of 0.01 eV/Å before the calculation of NMR tensors. The van der Waals forces in the system were described using the Grimme D3 dispersion correction^78,79^.

Configurations of (Na_2*y*_Zn_*16-y*_)((im)_1.75_(bim)_0.25_)_16_ were constructed using the Atomic Simulation Environment Python package. The DFT-relaxed structure of ZIF-62 (Zn_*16*_)((im)_1.75_(bim)_0.25_)_16_ was used as a base structure. Na^+^ ions were introduced by randomly inserting Na atoms into the base cell and removing a corresponding number of Zn^2+^ ions for charge balancing. The number of Na^+^ ions *N* = 2, 4, 6 and 8 corresponded to *x* = 0.13, 0.29, 0.46 and 0.67, respectively, in NaB_x_ZIF-62. Ten configurations for each of the specified Na-loading were created and geometry optimized.

We used the Python packages Soprano and mrsimulator^80^ to transform CASTEP NMR outputs into finite speed MAS solid-state NMR spectra. Soprano was used to scrape CASTEP.magres files for their chemical shielding and electric-field gradient tensors. Mrsimulator combined the tensor information with experimental parameters to simulate finite speed MAS ssNMR. The experimental parameters included a rotor frequency of 20,000 Hz and an external magnetic field of 20 T. Isotropic chemical shielding of ^23^Na was referenced against calculated and experimental NaCl at 551.59 ppm and 7.21 ppm, respectively.

## Online content

Any methods, additional references, Nature Portfolio reporting summaries, source data, extended data, supplementary information, acknowledgements, peer review information; details of author contributions and competing interests; and statements of data and code availability are available at 10.1038/s41557-026-02115-8.

## Supplementary information

Supplementary Information Supplementary Figs. 1–131, Discussion and Tables 1–20.

## Data Availability

The authors declare that all data supporting the findings of this study are available within the article and its Supplementary Information. The underlying datasets are available via Zenodo at 10.5281/zenodo.18495471 (ref. ^81^).

## Extended data

**Extended Data Table 1:** Composition of NaB_*x*_|ZIF-62 materials

*x*	*m*_ZIF-62_ / mg	*n*_ZIF-62_ / mmol	*m*_Na(bim)_ / mg	*n*_Na(bim)_ / mmol
0	250.0	1.19	0.0	0.00
0.1	234.3	1.12	15.7	0.11
0.3	208.2	0.99	41.8	0.30
0.5	187.4	0.89	62.6	0.45
1.0	149.8	0.71	100.2	0.71
1.5	124.8	0.60	125.2	0.89

The physical mixtures comprise various amounts of glass former ZIF-62 and glass modifier Na(bim).

## References

[1] Faber, K. T., Casadio, F., Masic, A., Robbiola, L. & Walton, M. Looking back, looking forward: materials science in art, archaeology, and art conservation. *Annu. Rev. Mater. Res.* **51**, 460 (2021).

[2] Goltsman, B. M. & Yatsenko, E. A. Modern fluxing materials and analysis of their impact on silicate structures: a review. *Open Ceram.* **17**, 100540 (2024).

[3] Musgraves, J. D., Hu, J. & Calvez, L. (eds) *Springer Handbook of Glass* (Springer, 2019).

[4] Ramakanth, D., Singh, S., Maji, P. K., Lee, Y. S. & Gaikwad, K. K. Advanced packaging for distribution and storage of COVID-19 vaccines: a review. *Environ. Chem. Lett.* **19**, 3597–3608 (2021). 34104127 10.1007/s10311-021-01256-1 PMC8173863

[5] Yang, Y. et al. The modification and strengthening dual functionality of rare-earth oxide in La_2_O_3_-BaO-SiO_2_ glass for high temperature sealing application. *J. Alloy. Compd.* **950**, 169892 (2023).

[6] Bennett, T. D. et al. Hybrid glasses from strong and fragile metal-organic framework liquids. *Nat. Commun.* **6**, 8079 (2015). 26314784 10.1038/ncomms9079 PMC4560802

[7] Ma, N. & Horike, S. Metal–organic network-forming glasses. *Chem. Rev.* **122**, 4163–4203 (2022). 35044749 10.1021/acs.chemrev.1c00826

[8] Banerjee, R. et al. High-throughput synthesis of zeolitic imidazolate frameworks and application to CO_2_ capture. *Science* **319**, 939–943 (2008). 18276887 10.1126/science.1152516

[9] Park, K. S. et al. Exceptional chemical and thermal stability of zeolitic imidazolate frameworks. *Proc. Natl Acad. Sci. USA* **103**, 10186–10191 (2006). 16798880 10.1073/pnas.0602439103 PMC1502432

[10] Bennett, T. D. et al. Melt-quenched glasses of metal-organic frameworks. *J. Am. Chem. Soc.* **138**, 3484–3492 (2016). 26885940 10.1021/jacs.5b13220

[11] Frentzel-Beyme, L., Kloß, M., Kolodzeiski, P., Pallach, R. & Henke, S. Meltable mixed-linker zeolitic imidazolate frameworks and their microporous glasses: from melting point engineering to selective hydrocarbon sorption. *J. Am. Chem. Soc.* **141**, 12362–12371 (2019). 31288513 10.1021/jacs.9b05558

[12] Frentzel-Beyme, L., Kolodzeiski, P., Weiß, J.-B., Schneemann, A. & Henke, S. Quantification of gas-accessible microporosity in metal-organic framework glasses. *Nat. Commun.* **13**, 7750 (2022). 36517486 10.1038/s41467-022-35372-5 PMC9751146

[13] Qiao, A. et al. A metal-organic framework with ultrahigh glass-forming ability. *Sci. Adv.* **4**, eaao6827 (2018). 29536040 10.1126/sciadv.aao6827 PMC5844704

[14] Wang, Y. et al. A MOF glass membrane for gas separation. *Angew. Chem. Int. Ed.* **59**, 4365–4369 (2020). 10.1002/anie.201915807 31893511

[15] Zhang, Y. et al. A hybrid ZIF-8/ZIF-62 glass membrane for gas separation. *Chem. Commun.* **58**, 9548–9551 (2022). 10.1039/d2cc03179e 35929541

[16] Xue, W., Das, C., Weiß, J. & Henke, S. Insights into the mechanochemical glass formation of zeolitic imidazolate frameworks. *Angew. Chem. Int. Ed.* **63**, e202405307 (2024). 10.1002/anie.202405307 38874082

[17] Song, J. et al. Modulating liquid–liquid transitions and glass formation in zeolitic imidazolate frameworks by decoration with electron-withdrawing cyano groups. *J. Am. Chem. Soc.* **145**, 9273–9284 (2023). 37070213 10.1021/jacs.3c01933

[18] Xue, W.-L. et al. Highly porous metal-organic framework liquids and glasses via a solvent-assisted linker exchange strategy of ZIF-8. *Nat. Commun.* **15**, 4420 (2024). 38789474 10.1038/s41467-024-48703-5 PMC11126584

[19] Bumstead, A. M. et al. Investigating the melting behaviour of polymorphic zeolitic imidazolate frameworks. *Cryst. Eng. Comm.* **22**, 3627–3637 (2020).

[20] Madsen, R. S. K., Sarkar, S., Iversen, B. B. & Yue, Y. Sensitivity of the glass transition and melting in a metal–organic framework to ligand chemistry. *Chem. Commun.* **58**, 823–826 (2021). 10.1039/d1cc03541j 34929725

[21] Thorne, M., Gomez, M. L. R., Bumstead, A., Li, S. & Bennett, T. Mechanochemical synthesis of mixed metal, mixed linker glass-forming metal–organic frameworks. *Green Chem.* **22**, 2505–2512 (2020).

[22] Frentzel-Beyme, L. et al. Porous purple glass—a cobalt imidazolate glass with accessible porosity from a meltable cobalt imidazolate framework. *J. Mater. Chem. A* **7**, 985–990 (2019).

[23] Nozari, V. et al. Ionic liquid facilitated melting of the metal-organic framework ZIF-8. *Nat. Commun.* **12**, 5703 (2021). 34588462 10.1038/s41467-021-25970-0 PMC8481281

[24] Sørensen, S. S. et al. Water promotes melting of a metal–organic framework. *Chem. Mater.* **36**, 2756–2766 (2024). 38558915 10.1021/acs.chemmater.3c02873 PMC10976635

[25] Hou, J. et al. Halogenated metal–organic framework glasses and liquids. *J. Am. Chem. Soc.* **142**, 3880–3890 (2020). 31978302 10.1021/jacs.9b11639

[26] Nozari, V. et al. Low-temperature melting and glass formation of the zeolitic imidazolate frameworks ZIF-62 and ZIF-76 through ionic liquid incorporation. *Adv. Mater. Technol.* **7**, 2200343 (2022).

[27] Bumstead, A. M. et al. Formation of a meltable purinate metal–organic framework and its glass analogue. *Chem. Commun.* **59**, 732–735 (2022). 10.1039/d2cc05314d 36541403

[28] Ashling, C. W. et al. Synthesis and properties of a compositional series of MIL-53(Al) metal–organic framework crystal-glass composites. *J. Am. Chem. Soc.* **141**, 15641–15648 (2019). 31491080 10.1021/jacs.9b07557 PMC7007233

[29] Li, S. et al. A new route to porous metal–organic framework crystal–glass composites. *Chem. Sci.* **11**, 9910–9918 (2020).

[30] Longley, L. et al. Liquid phase blending of metal-organic frameworks. *Nat. Commun.* **9**, 2135 (2018). 29907760 10.1038/s41467-018-04553-6 PMC6004012

[31] Longley, L. et al. Flux melting of metal–organic frameworks. *Chem. Sci.* **10**, 3592–3601 (2019). 30996951 10.1039/c8sc04044c PMC6430010

[32] Cao, F. et al. Continuous structure modification of metal-organic framework glasses via halide salts. *Nat. Commun.* **16**, 7001 (2025). 40739141 10.1038/s41467-025-62143-9 PMC12311036

[33] Kolodzeiski, P. et al. Lithium and sodium benzimidazolate coordination networks: syntheses, structures, and thermal properties. *Cryst. Growth Des.* **24**, 7278–7286 (2024).

[34] Hood, H. P. & Nordberg, M. E. Treated borosilicate glass. US patent 2,106,744 (1938).

[35] Inayat, A., Reinhardt, B., Uhlig, H., Einicke, W.-D. & Enke, D. Silica monoliths with hierarchical porosity obtained from porous glasses. *Chem. Soc. Rev.* **42**, 3753–3764 (2012). 23081802 10.1039/c2cs35304k

[36] Wharmby, M. T. et al. Extreme flexibility in a zeolitic imidazolate framework: porous to dense phase transition in desolvated ZIF-4. *Angew. Chem. Int. Ed.* **54**, 6447–6451 (2015). 10.1002/anie.201410167 25873105

[37] Gault, B. et al. Atom probe tomography. *Nat. Rev. Methods Prim.* **1**, 51 (2021). 10.1038/s43586-021-00047-w PMC10502706 37719173

[38] Li, T., Devaraj, A. & Kruse, N. Atomic-scale characterization of (electro-)catalysts and battery materials by atom probe tomography. *Cell Rep. Phys. Sci.* **3**, 101188 (2022).

[39] Zhao, P. et al. Phase transitions in zeolitic imidazolate framework 7: the importance of framework flexibility and guest-induced instability. *Chem. Mater.* **26**, 1767–1769 (2014). 24634567 10.1021/cm500407f PMC3953891

[40] Zheng, Q., Zheng, J., Solvang, M., Yue, Y. & Mauro, J. C. Determining the liquidus viscosity of glass-forming liquids through differential scanning calorimetry. *J. Am. Ceram. Soc.* **103**, 6070–6074 (2020).

[41] Zheng, Q., Mauro, J. C. & Yue, Y. Reconciling calorimetric and kinetic fragilities of glass-forming liquids. *J. Non-Cryst. Solids* **456**, 95–100 (2017).

[42] Angell, C. A. Formation of glasses from liquids and biopolymers. *Science* **267**, 1924–1935 (1995). 17770101 10.1126/science.267.5206.1924

[43] Chen, Z. et al. Calorimetric determination of fragility in glass forming liquids: T_f_ vs. T_g_-onset methods. *Eur. Phys. J. E* **37**, 52 (2014). 10.1140/epje/i2014-14052-y 24965151

[44] Liu, H. et al. A medium range order structural connection to the configurational heat capacity of borate–silicate mixed glasses. *Phys. Chem. Chem. Phys.* **18**, 10887–10895 (2016). 27040155 10.1039/c6cp00749j

[45] Ogawa, T. et al. Network size control in coordination polymer glasses and its impact on viscosity and H^+^ conductivity. *Chem. Mater.* **34**, 5832–5841 (2022).

[46] Jiusti, J. et al. Effect of network formers and modifiers on the crystallization resistance of oxide glasses. *J. Non-Cryst. Solids* **550**, 120359 (2020).

[47] Hadjiivanov, K. I. et al. Power of infrared and Raman spectroscopies to characterize metal-organic frameworks and investigate their interaction with guest molecules. *Chem. Rev.* **121**, 1286–1424 (2021). 33315388 10.1021/acs.chemrev.0c00487

[48] Ryder, M. R. et al. Identifying the role of terahertz vibrations in metal-organic frameworks: from gate-opening phenomenon to shear-driven structural destabilization. *Phys. Rev. Lett.* **113**, 215502 (2014). 25479503 10.1103/PhysRevLett.113.215502

[49] Elliott, S. R. Extended-range order, interstitial voids and the first sharp diffraction peak of network glasses. *J. Non-Cryst. Solids* **182**, 40–48 (1995).

[50] Crupi, C., Carini, G., Ruello, G. & D’Angelo, G. Intermediate range order in alkaline borate glasses. *Philos. Mag.* **96**, 788–799 (2016).

[51] Chapman, K. W. & Chupas, P. J. in *In-situ Characterization of Heterogeneous Catalysts* (eds Rodriguez, J. A. et al.) 147–168 (John Wiley & Sons, 2013).

[52] Castillo-Blas, C., Romero-Muñiz, I., Mavrandonakis, A., Simonelli, L. & Platero-Prats, A. E. Unravelling the local structure of catalytic Fe-oxo clusters stabilized on the MOF-808 metal organic-framework. *Chem. Commun.* **56**, 15615–15618 (2020). 10.1039/d0cc06134d 33290455

[53] Zhou, S. et al. Reactive passivation of wide-bandgap organic–inorganic perovskites with benzylamine. *J. Am. Chem. Soc.* **146**, 27405–27416 (2024). 39348291 10.1021/jacs.4c06659 PMC11467896

[54] Pickard, C. J. & Needs, R. J. Ab initio random structure searching. *J. Phys. Condens. Matter* **23**, 053201 (2011). 21406903 10.1088/0953-8984/23/5/053201

[55] Greaves, G. N. EXAFS and the structure of glass. *J. Non-Cryst. Solids* **71**, 203–217 (1985).

[56] Nhan, N. T., Lien, P. T., Kien, P. H., San, L. T. & Hung, P. K. Study of diffusion in sodium silicate glass using molecular dynamics simulation. *Silicon* **16**, 5571–5581 (2024).

[57] Smirnova, O. et al. Precise control over gas-transporting channels in zeolitic imidazolate framework glasses. *Nat. Mater.* **23**, 262–270 (2024). 38123813 10.1038/s41563-023-01738-3 PMC10837076

[58] Enke, D., Janowski, F. & Schwieger, W. Porous glasses in the 21st century––a short review. *Microporous Mesoporous Mater.* **60**, 19–30 (2003).

[59] Dantas, S., Struckhoff, K. C., Thommes, M. & Neimark, A. V. Phase behavior and capillary condensation hysteresis of carbon dioxide in mesopores. *Langmuir* **35**, 11291–11298 (2019). 31380648 10.1021/acs.langmuir.9b01748

[60] Samios, S., Stubos, A. K., Papadopoulos, G. K., Kanellopoulos, N. K. & Rigas, F. The structure of adsorbed CO_2_ in slitlike micropores at low and high temperature and the resulting micropore size distribution based on GCMC simulations. *J. Colloid Interface Sci.* **224**, 272–290 (2000). 10727338 10.1006/jcis.1999.6683

[61] Branton, P. J., Hall, P. G., Treguer, M. & Sing, K. S. W. Adsorption of carbon dioxide, sulfur dioxide and water vapour by MCM-41, a model mesoporous adsorbent. *J. Chem. Soc. Faraday Trans.* **91**, 2041–2043 (1995).

[62] Vishnyakov, A., Ravikovitch, P. I. & Neimark, A. V. Molecular level models for CO_2_ sorption in nanopores. *Langmuir* **15**, 8736–8742 (1999).

[63] Deubener, J., Müller, R., Behrens, H. & Heide, G. Water and the glass transition temperature of silicate melts. *J. Non-Cryst. Solids* **330**, 268–273 (2003).

[64] Basham, M. et al. Data Analysis WorkbeNch (DAWN). *J. Synchrotron. Rad.* **22**, 853–858 (2015). 10.1107/S1600577515002283 PMC4416692 25931106

[65] Filik, J. et al. Processing two-dimensional X-ray diffraction and small-angle scattering data in DAWN 2. *J. Appl. Cryst.* **50**, 959–966 (2017). 28656043 10.1107/S1600576717004708 PMC5458597

[66] Pawley, G. S. Unit-cell refinement from powder diffraction scans. *J. Appl. Cryst.* **14**, 357–361 (1981).

[67] Coelho, A. A. TOPAS and TOPAS-Academic: an optimization program integrating computer algebra and crystallographic objects written in C++. *J. Appl. Cryst.* **51**, 210–218 (2018).

[68] Juhás, P., Davis, T., Farrow, C. L. & Billinge, S. J. L. PDFgetX3: a rapid and highly automatable program for processing powder diffraction data into total scattering pair distribution functions. *J. Appl. Cryst.* **46**, 560–566 (2013).

[69] Simões, J. A. M. & da Piedade, M. M. *Molecular Energetics: Condensed-Phase Thermochemical Techniques* (Oxford Univ. Press, 2008).

[70] Thurber, K. R. & Tycko, R. Measurement of sample temperatures under magic-angle spinning from the chemical shift and spin-lattice relaxation rate of 79Br in KBr powder. *J. Magn. Reson.* **196**, 84–87 (2009). 18930418 10.1016/j.jmr.2008.09.019 PMC2632797

[71] Clark, S. J. et al. First principles methods using CASTEP. *Z. Kristallogr. Cryst. Mater.* **220**, 567–570 (2005).

[72] Bonhomme, C. et al. First-principles calculation of NMR parameters using the gauge including projector augmented wave method: a chemist’s point of view. *Chem. Rev.* **112**, 5733–5779 (2012). 23113537 10.1021/cr300108a

[73] Yates, J. R., Pickard, C. J. & Mauri, F. Calculation of NMR chemical shifts for extended systems using ultrasoft pseudopotentials. *Phys. Rev. B* **76**, 024401 (2007).

[74] Pickard, C. J. & Mauri, F. All-electron magnetic response with pseudopotentials: NMR chemical shifts. *Phys. Rev. B* **63**, 245101 (2001).

[75] Profeta, M., Mauri, F. & Pickard, C. J. Accurate first principles prediction of 17 O NMR parameters in SiO_2_: assignment of the zeolite ferrierite spectrum. *J. Am. Chem. Soc.* **125**, 541–548 (2003). 12517169 10.1021/ja027124r

[76] Perdew, J. P., Burke, K. & Ernzerhof, M. Generalized gradient approximation made simple. *Phys. Rev. Lett.* **77**, 3865–3868 (1996). 10062328 10.1103/PhysRevLett.77.3865

[77] Monkhorst, H. J. & Pack, J. D. Special points for Brillouin-zone integrations. *Phys. Rev. B* **13**, 5188–5192 (1976).

[78] McNellis, E. R., Meyer, J. & Reuter, K. Azobenzene at coinage metal surfaces: role of dispersive van der Waals interactions. *Phys. Rev. B* **80**, 205414 (2009).

[79] Grimme, S., Antony, J., Ehrlich, S. & Krieg, H. A consistent and accurate ab initio parametrization of density functional dispersion correction (DFT-D) for the 94 elements H-Pu. *J. Chem. Phys.* **132**, 154104 (2010). 20423165 10.1063/1.3382344

[80] Srivastava, D. J., Giammar, M., Venetos, M. C., McCarthy-Carney, L. & Grandinetti, P. J. MRSimulator: a cross-platform, object-oriented software package for rapid solid-state NMR spectral simulation and analysis. *J. Chem. Phys.* **161**, 212501 (2024). 39620417 10.1063/5.0237608

[81] Kolodzeiski, P. et al. Alkali-ion-modified zeolitic imidazolate framework glasses. *Zenodo*10.5281/zenodo.18495471 (2026). 10.1038/s41557-026-02115-8 PMC13423876 42082788

