# Supplementary material for: Alkali-ion-modified zeolitic imidazolate framework glasses
Source: Nat Chem. 2026 May 4;18(8):1383–92. doi: 10.1038/s41557-026-02115-8 (PMC13423876; doi:10.1038/s41557-026-02115-8)
Supplement: Supplementary file 1 — Supplementary Figs. 1–131, Discussion and Tables 1–20. [file 41557_2026_2115_MOESM1_ESM.pdf]

nature chemistry

Supplementary information https://doi.org/10.1038/s41557-026-02115-8

# Alkali-ion-modified zeolitic imidazolate framework glasses

In the format provided by the authors and unedited

# Content

S1. Infrared Spectroscopy ....................................................................................3
S2. Density Functional Theory Calculation and Vibrational Analysis of Na(bim).....5
S3. Optical Microscopy Images ....................................................................................8
S4. Large-Scale Vitrification Set-up (Autoclave approach)....................................10
S5. Simultaneous Thermal Analysis ..........................................................................11
S6. X-Ray Powder Diffraction ....................................................................................13
S7. Solution NMR Spectroscopy.................................................................................16
S8. Variable Temperature Powder X-Ray Diffraction ..............................................23
S9. Differential Scanning Calorimetry .......................................................................27
S9.1. Calorimetric Fragility Measurements..............................................................31
S9.2. Heat Capacity Measurements ........................................................................36
S10. X-Ray Pair Distribution Function Analysis ........................................................38
S10.1. Experimental Details ....................................................................................38
S10.2. Variable Temperature *I*(*Q*) Data .......................................................................39
S10.3. Variable Temperature *S*(*Q*) Data .......................................................................41
S10.4. Variable Temperature *G*(*r*) Data .......................................................................43
S10.5. First Sharp Diffraction Peak Analysis...........................................................46
S10.6. Differential PDF Analysis ..............................................................................48
S10.7. Discussion of $\Delta G^*(r)$ approach ................................................................52
S11. Extended X-ray Absorption Fine Structure Analysis..........................................54
S11.1. Experimental Details ....................................................................................54
S12. Atom Probe Tomography ....................................................................................60
S13. Electron Microscopy and Energy-Dispersive X-Ray Spectroscopy .................62
S14. Density Functional Theory Calculations ..........................................................65
S15. Solid-State Nuclear Magnetic Resonance Spectroscopy ................................72
S16. Water Leaching Experiments ..............................................................................80
S16.1. Optical Microscopy Images ...........................................................................80
S16.2. X-Ray Diffraction Measurements ..................................................................81
S16.3. Solution $^1$H NMR Spectroscopy ..................................................................82
S16.4. SEM Imaging..................................................................................................84
S16.5. Infrared Spectroscopy ...................................................................................85
S16.6. X-ray Pair Distribution Function Analysis .....................................................86
S16.7. Differential Scanning Calorimetry..................................................................87

S16.8. Quantification of $Na^+$ Content by $^{23}Na$ MAS NMR....................................88
S16.9. Gas Sorption Experiments ....................................................................89
S17. Atmospheric Stability ...............................................................................95
S18. Lithium Benzimidazolate Modification ........................................................96
S18.1. Experimental Details ..............................................................................97
S18.2. Infrared Spectroscopy ............................................................................98
S18.3. Microscopy Images ................................................................................98
S18.4. Powder X-ray Diffraction ........................................................................99
S18.5. Solution $^1H$ NMR Spectroscopy ...........................................................101
S18.6. Differential Scanning Calorimetry..........................................................104
S18.7. Variable Temperature Powder X-ray Diffraction ....................................106
S18.8. X-ray Pair Distribution Function Analysis ..............................................107
S19. Bibliography.............................................................................................108

# S1. Infrared Spectroscopy

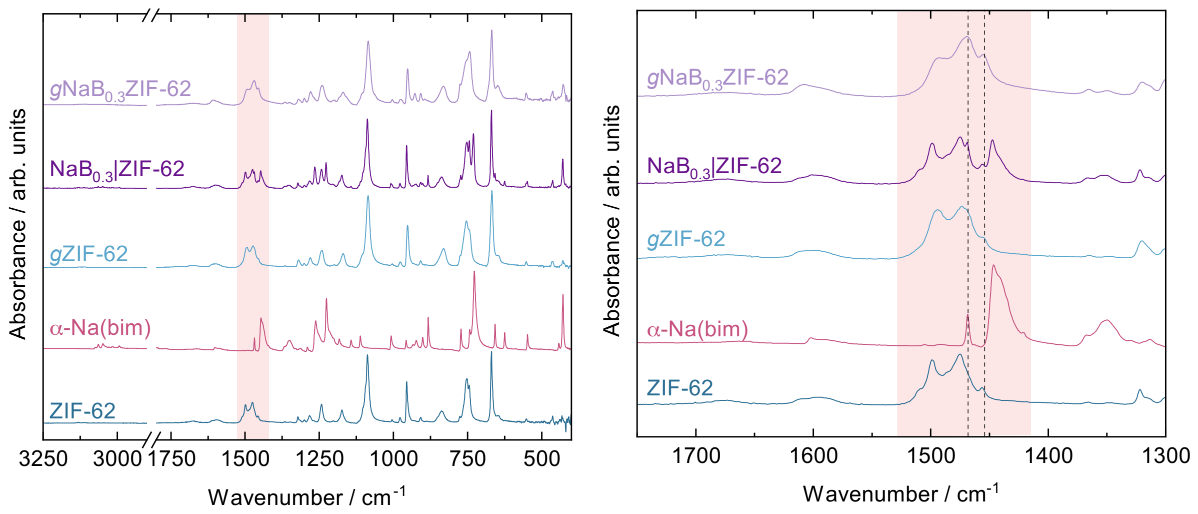


**Supplementary Figure 1.** Mid-infrared spectra of ZIF-62, the corresponding glass *g*ZIF-62, the modifier α-Na(bim), the physical mixture of modifier and glass former $NaB_{0.3}$|ZIF-62 and the corresponding glass of the physical mixture *g*$NaB_{0.3}$ZIF-62 with a zoom into the highlighted wavenumber range (right).

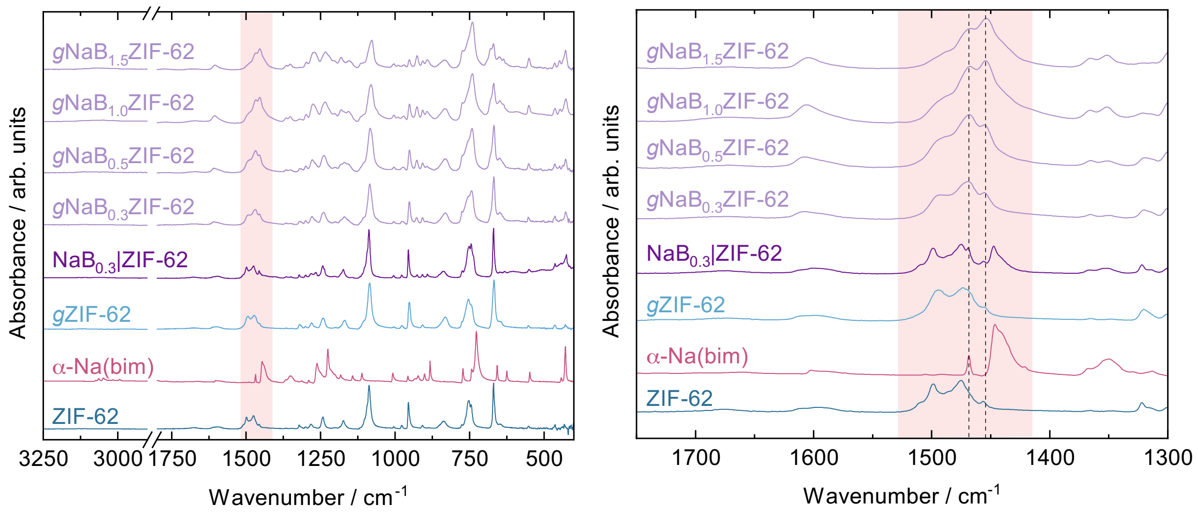


**Supplementary Figure 2.** Mid-infrared spectra of ZIF-62, the corresponding glass *g*ZIF-62, the modifier α-Na(bim), and Na(bim)-modified ZIF-62 glasses *g*$NaB_x$ZIF-62 with different amounts of modifier incorporated *x*. The right figure shows a zoom into the region from 1300 – 1750 $cm^{-1}$.

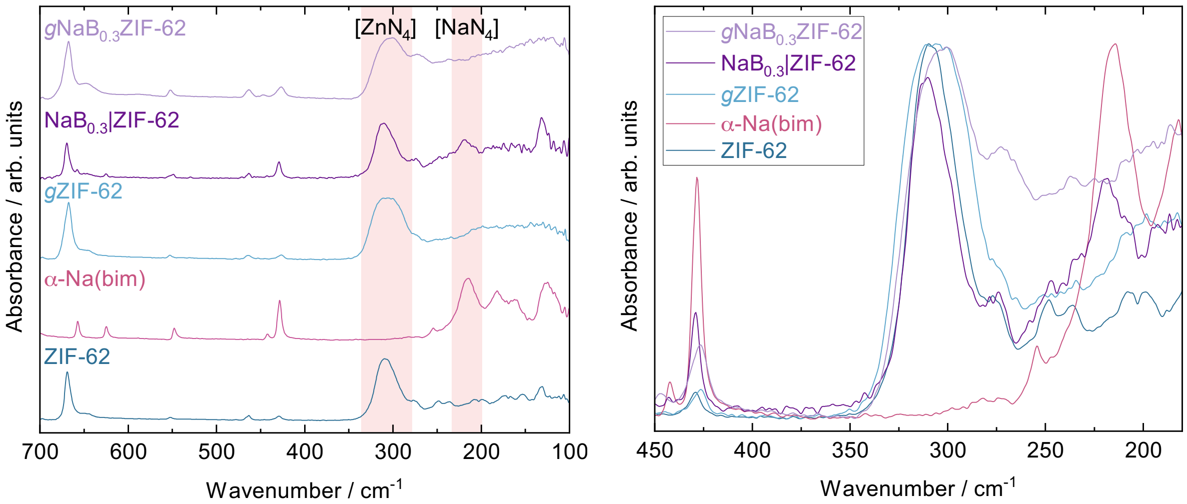


**Supplementary Figure 3.** Far-infrared spectra of ZIF-62, the corresponding glass *g*ZIF-62, the modifier α-Na(bim), the physical mixture of modifier and glass former $NaB_{0.3}$|ZIF-62 and the corresponding glass of the physical mixture *g*$NaB_{0.3}$ZIF-62 with a zoom into the highlighted wavenumber range (right).

## S2. Density Functional Theory Calculation and Vibrational Analysis of Na(bim)

The IR spectra of α-Na(bim) were calculated using the Vienna Ab Initio Simulation Package (VASP, version 5.4)[1,2], using periodic density functional (DFT) with the optPBE-vdw functional and projected augmented wave (PAW) pseudo-potentials. The IR calculations are based on a geometry-optimized structure, which included both the relaxation of the cell parameters as well as the relaxation of the nuclei position. The cut-off energy of the plane wave basis set is 400 eV. For describing the electronic states, the Methfessel-Paxton smearing method was employed with a smearing width of 0.2 eV. The convergence criteria for the forces on the nuclei was 0.02 eV/Å and the criterion for the electronic relaxation is set to $10^{-7}$ eV. The Brillouin zone integration is based on a 6x6x1 k-point grid.

The optimised lattice constants of the used unit cell are $a$ = 8.2672 Å, $b$ = 7.0732 Å, and $c$ = 11.3000 Å (see Supplementary Figure 4). The IR calculations were carried out using the density functional perturbation theory (DFPT) vibrational analysis approach.[3–5]

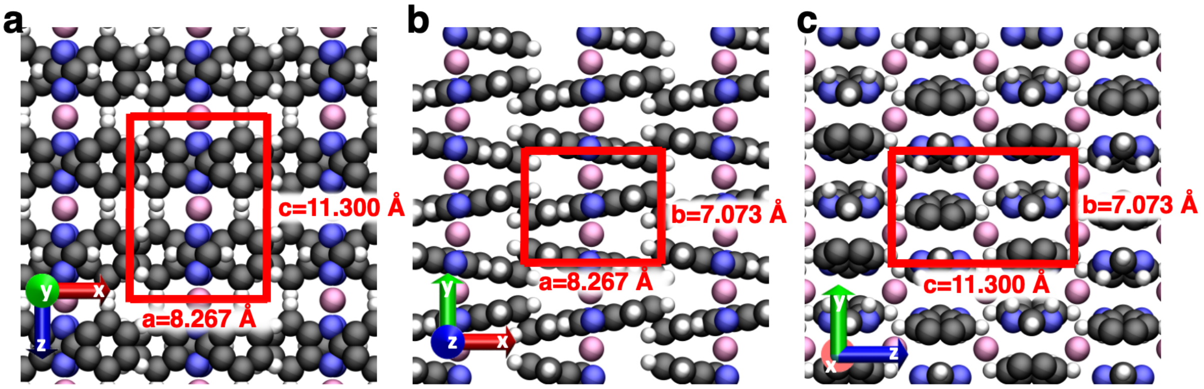


**Supplementary Figure 4.** Visualization of the geometry-optimized molecular structure of α-Na(bim). The C-atoms are coloured in black, the N atoms in blue, H in white, and Na in light purple. a), b), and c) show the structure, including the unit cell (red rectangle), along three different orientations. The respective orientation is highlighted by the axes at the bottom left corner of each panel.

**Supplementary Table 1.** Wavenumbers of prominent vibrational bands found in the experimental far-IR spectra ($\tilde{\nu}$exp), the corresponding vibrational band obtained by DFT ($\tilde{\nu}$calc) and a description of the vibrational mode. Animations of the corresponding molecular vibrations are available at http://doi.org/10.6084/m9.figshare.28264145.

| $\tilde{\nu}_{exp}$ / $cm^{-1}$ | $\tilde{\nu}_{calc}$ / $cm^{-1}$ | Description of vibrational mode |
|---|---|---|
| 657 | 650.0 | Symmetric $N_4$ in-plane vibration, Na-N stretching contribution |
| 624 | 626.8 | Aromatic in-plane deformation, Na-N stretching contribution |
| 547 | 555.4 | Aromatic in-plane deformation vibration |
| 442 | 435.4 | Benzimidazolate in-plane vibration, Na-N stretching vibration contribution |
| 428 | 429.8 | Phenyl out-of-plane vibration |
| 253 | 269.2 | Benzimidazolate out-of-plane vibration |
| 214 | 211.4 | $NaN_4$ in-plane vibration |
| 182 | 194.6 | $NaN_4$ in-plane vibration |

**Supplementary Table 2.** Wavenumbers of prominent vibrational bands found in the experimental mid-IR spectra ($\tilde{\nu}$exp), the corresponding vibrational band obtained by DFT ($\tilde{\nu}$calc) and a description of the vibrational mode. Animations of the corresponding molecular vibrations are available at http://doi.org/10.6084/m9.figshare.28264145.

| $\tilde{\nu}_{exp}$ / $cm^{-1}$ | $\tilde{\nu}_{calc}$ / $cm^{-1}$ | Description of vibrational mode |
|---|---|---|
| 3048 - 3075 | 3084.0 | C-H stretching vibration |
| 1601 | 1575.0 | In-plane-ring stretching vibration |
| 1468 | 1462.0 | In-plane ring-stretching vibration |
| 1446 | 1437.0 | In-plane ring-stretching vibration |
| 1446 | 1424.0 | In-plane C-N stretching vibration (asymmetric) |
| 1261 | 1249.0 | In-plane C-N stretching vibration (symmetric) |
| 1225 | 1218.0 | In-plane C-N stretching vibration (asymmetric) |
| 882 | 874.2 | In-plane ring stretching vibration |
| 882 | 873.4 | C-N out-of-plane vibration |
| 882 | 872.8 | C-N out-of-plane vibration |
| 727 | 724.4 | C-N out-of-plane vibration |

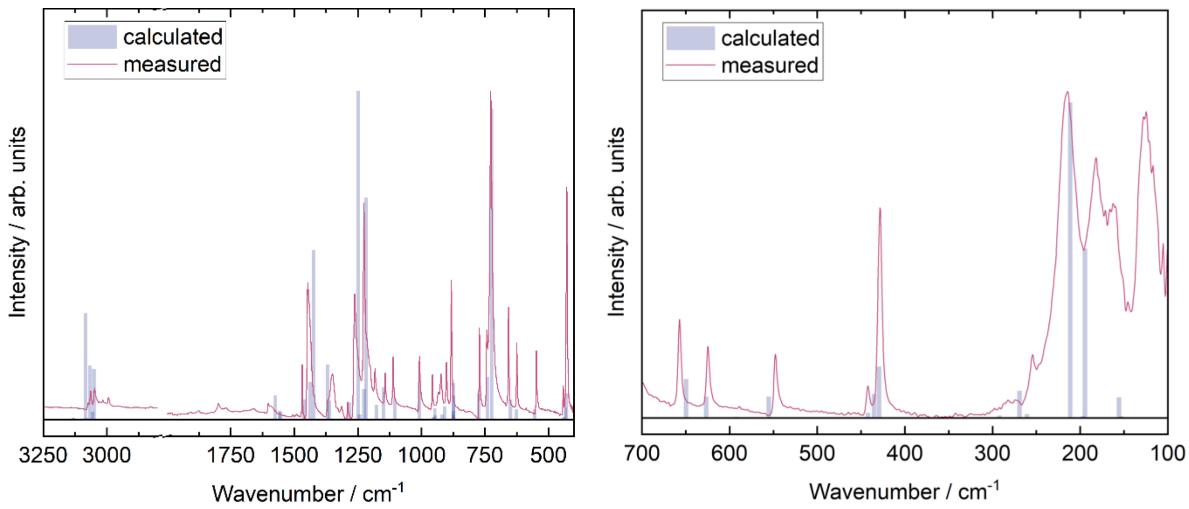


**Supplementary Figure 5.** MIR (left) and FIR (right) spectra of α-Na(bim) shown together with the intensity distribution of vibrational bands obtained by DFT calculations.

## S3. Optical Microscopy Images

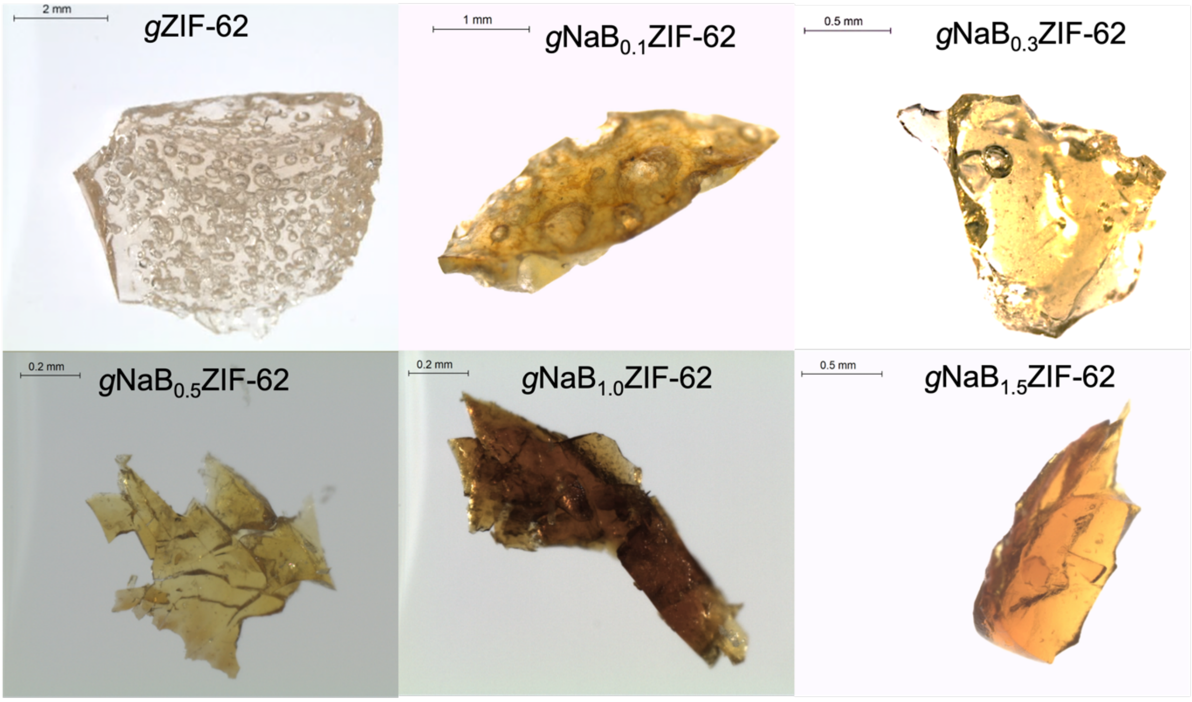


**Supplementary Figure 6.** Optical microscopy images of shards of *g*ZIF-62 (autoclave approach) and *g*NaB$_x$ZIF-62 materials with different Na(bim) contents *x* prepared on a small scale in a DSC apparatus.

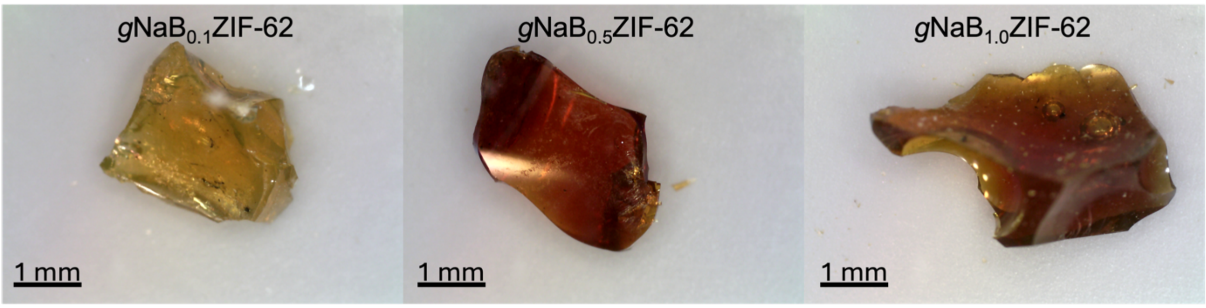


**Supplementary Figure 7.** Optical microscopy images of shards of *g*NaB$_x$ZIF-62 materials with different Na(bim) contents *x*. The glasses were prepared using the autoclave approach.

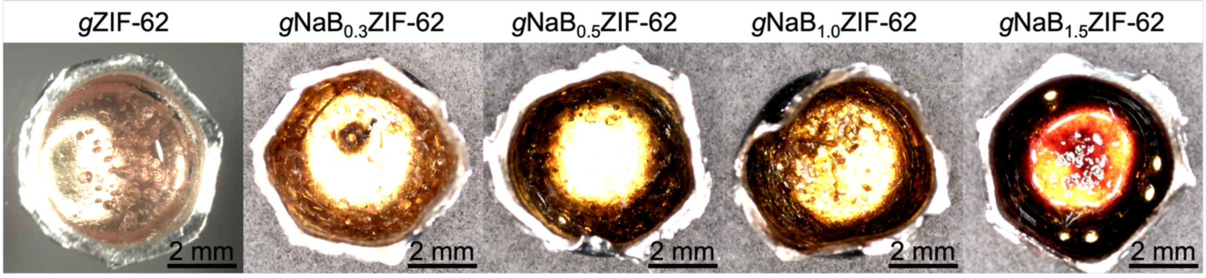


**Supplementary Figure 8.** Optical microscopy images of *g*ZIF-62 and *g*NaB$_x$ZIF-62 materials in DSC pans with different Na(bim) contents *x*. The glasses were prepared in a DSC apparatus (~10 mg scale) in a sealed DSC pan, which was opened after the heating-cooling cycle for melting and glass formation. The images illustrate the pronounced wetting behaviour of the modified glasses compared to pure *g*ZIF-62 and the formation of a concave meniscus on the surface of the modified glasses compared to a convex meniscus for *g*ZIF-62. This behaviour is in accordance with the lower viscosity of the modified glasses, as the concave meniscus formation requires sufficient fluidity in the liquid state.

## S4. Large-Scale Vitrification Set-up (Autoclave approach)

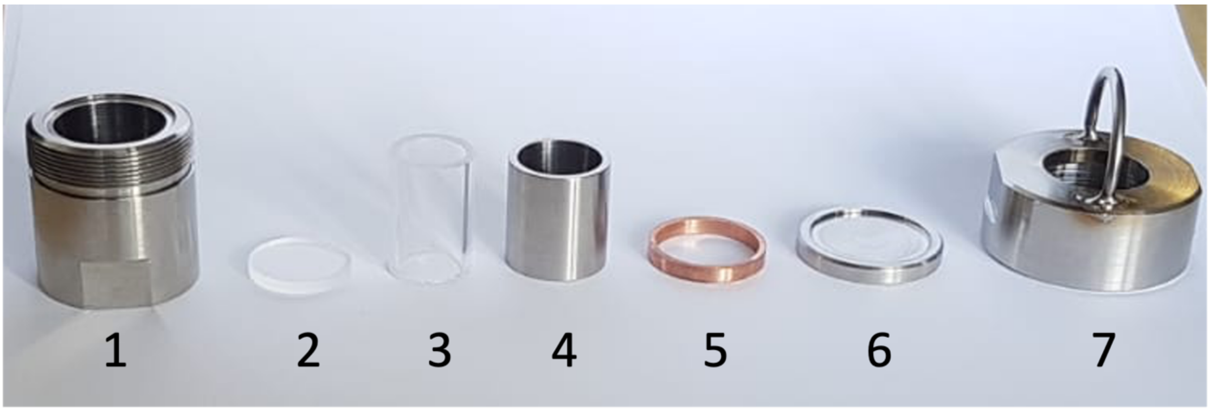


**Supplementary Figure 9.** Photograph of the disassembled custom-made hermetic crucible for the vitrification on a larger scale consisting of a stainless-steel mantle (1), the top plate (6), the screw cap (7) and a copper sealing ring (5) sitting between the mantle and the top plate. The sample material itself is placed in a quartz-glass inlet consisting of the bottom plate (2) and the quartz-glass mantle (3), which is stabilised against tilting by the inner stainless-steel mantle (4).

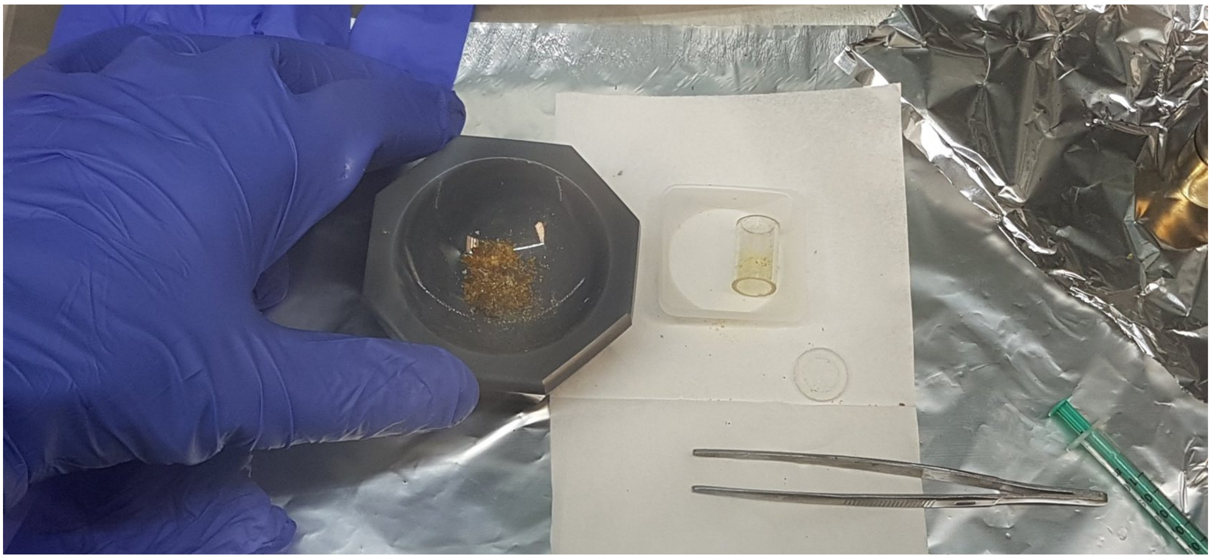

**Supplementary Figure 10.** Photograph taken during the removal of melt-quenched *g*$NaB_{0.5}$ZIF-62 from the custom-made hermetic crucible (autoclave approach). The strong adhesion of the modified glass to the quartz glass inlet after melt-quenching required breaking the monolith.

## S5. Simultaneous Thermal Analysis

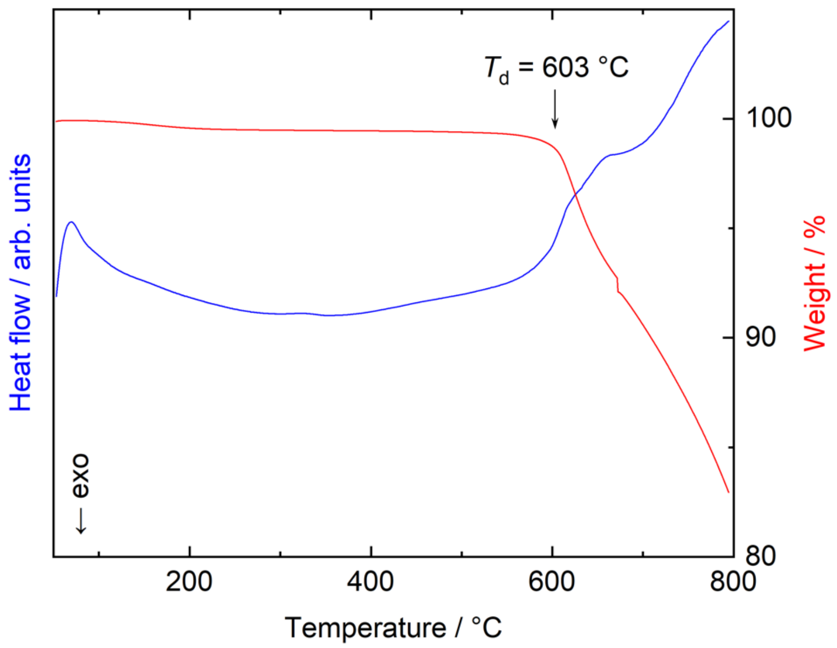


**Supplementary Figure 11.** STA thermogram of *g*ZIF-62 recorded with a constant heating rate of 10 °C min$^{-1}$ under constant $N_2$ flow. The arrow indicates the decomposition temperature $T_d$.

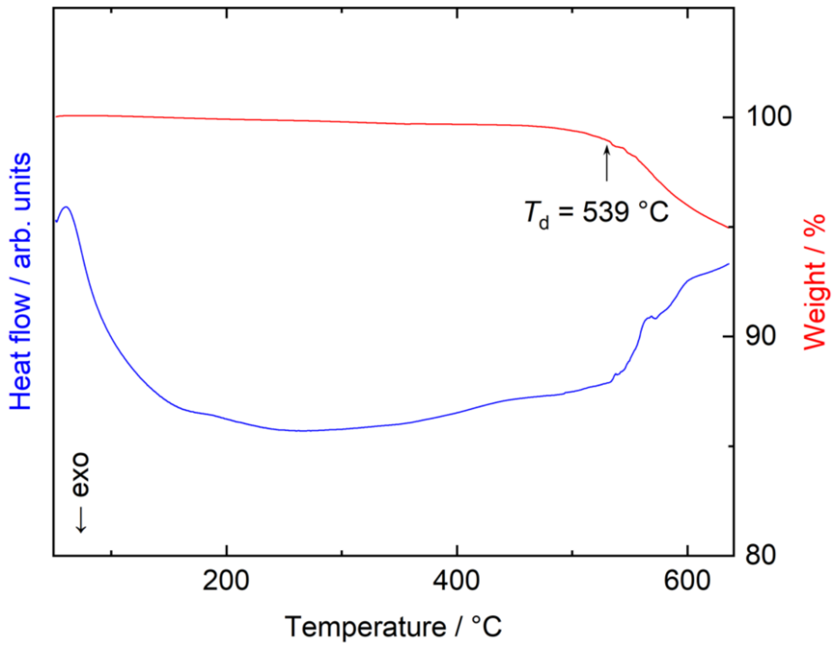


**Supplementary Figure 12.** STA thermogram of *g*$NaB_{0.5}$ZIF-62 recorded with a constant heating rate of 10 °C min$^{-1}$ under constant $N_2$ flow. The arrow indicates the decomposition temperature $T_d$.

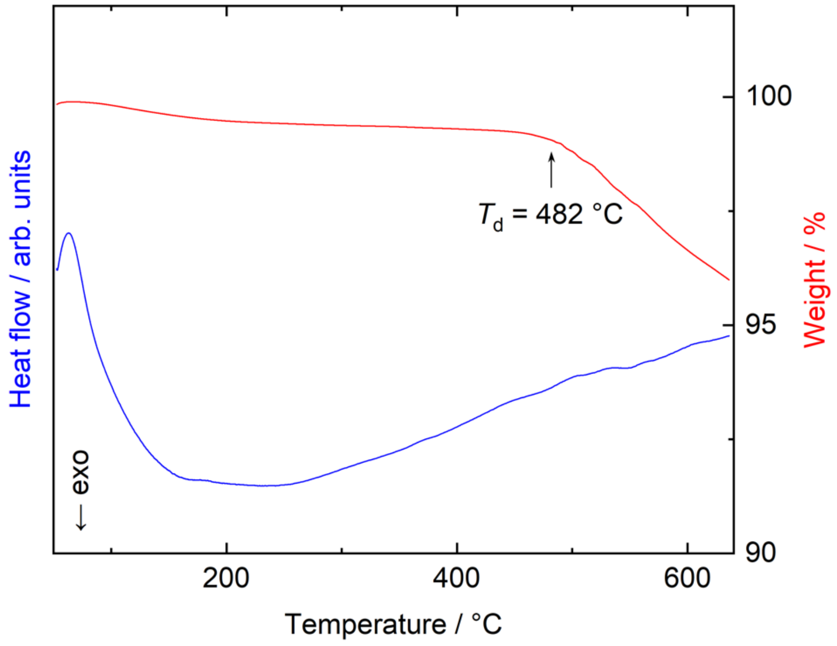


**Supplementary Figure 13.** STA thermogram of *g*NaB$_{1.0}$ZIF-62 recorded with a constant heating rate of 10 °C min$^{-1}$ under constant $N_2$ flow. The arrow indicates the decomposition temperature $T_d$.

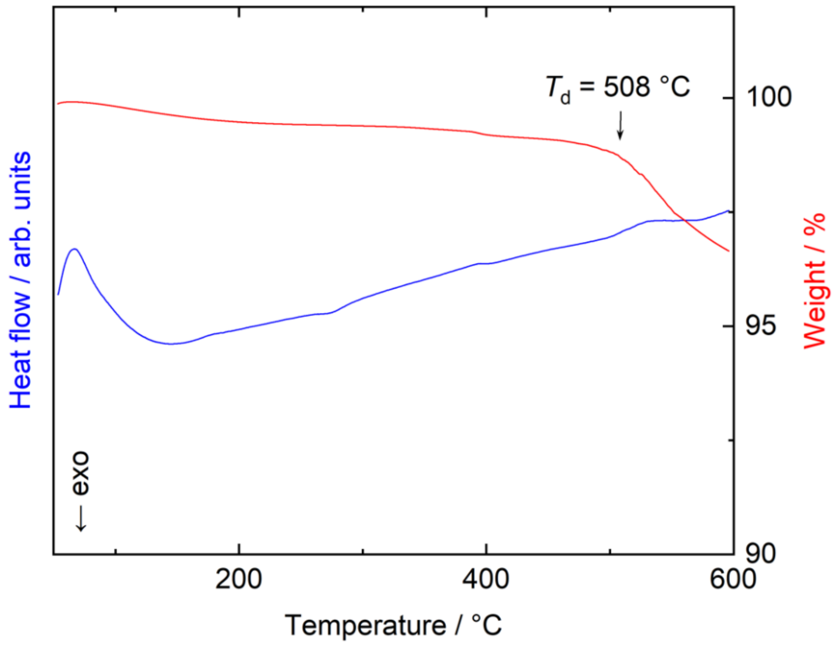


**Supplementary Figure 14.** STA thermogram of *g*NaB$_{1.5}$ZIF-62 recorded with a constant heating rate of 10 °C min$^{-1}$ under constant $N_2$ flow. The arrow indicates the decomposition temperature $T_d$.

# S6. X-Ray Powder Diffraction

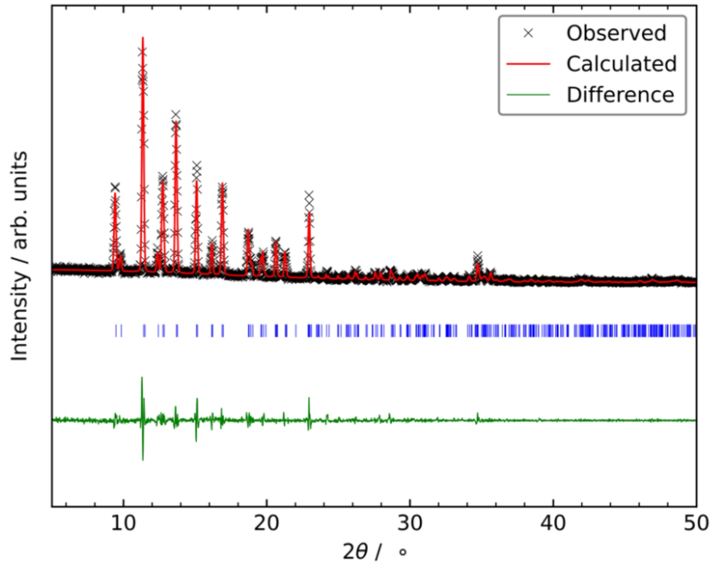


**Supplementary Figure 15.** Structureless profile fit (Pawley method[6]) performed on the PXRD measurement of ZIF-62 recorded with CuK$_{\alpha}$ radiation. The blue tick marks indicate allowed Bragg peak positions. Refined unit cell parameters are shown in Supplementary Table 3.

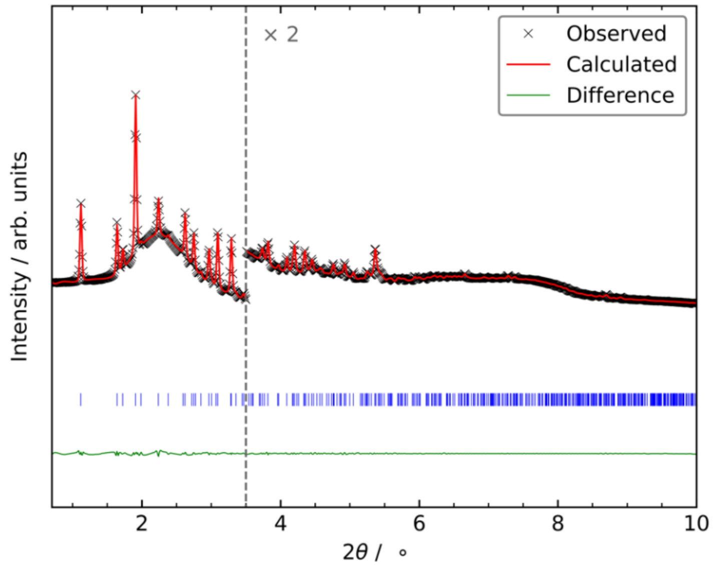


**Supplementary Figure 16.** Structureless profile fit (Pawley method[6]) performed on the PXRD measurement of α-Na(bim) recorded at room temperature with a wavelength of 0.1617 Å at DLS. Blue tick marks indicate the position of allowed Bragg peaks. Refined unit cell parameters are shown in Supplementary Table 3.

**Supplementary Table 3.** Refined parameters obtained by profile fitting (Pawley method[6]) performed on the PXRD data of ZIF-62 and α-Na(bim).

| Material | ZIF-62 | α-Na(bim) |
|---|---|---|
| crystal system | orthorhombic | orthorhombic |
| space group | *Pbca* | *Pbcm* |
| $a$ / Å | 15.371(4) | 8.282(2) |
| $b$ / Å | 15.494(3) | 7.0677(9) |
| $c$ / Å | 17.940(4) | 11.292(2) |
| $\alpha$ / ° | 90 | 90 |
| $\beta$ / ° | 90 | 90 |
| $\gamma$ / ° | 90 | 90 |
| $V$ / Å$^3$ | 4272(2) | 661.0(2) |
| $R_{wp}$ / % | 13.85 | 0.81 |
| $R_{exp}$ / % | 9.33 | 1.16 |
| $\chi$ | 1.49 | 0.70 |

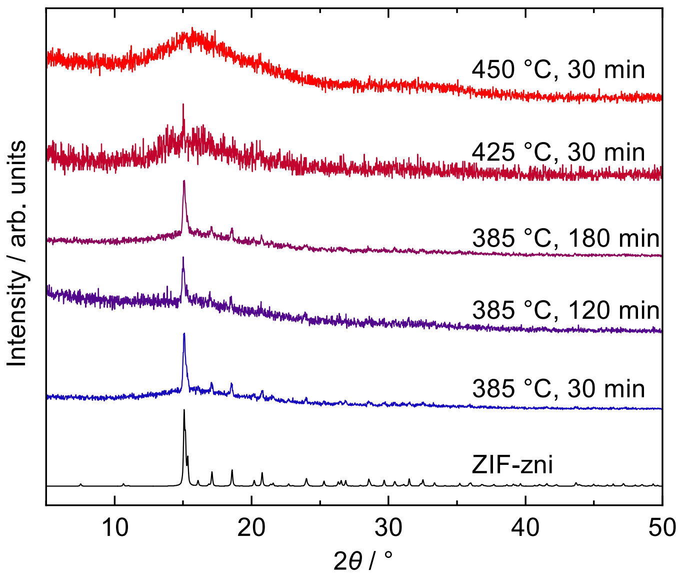


**Supplementary Figure 17.** PXRD patterns of $NaB_{0.3}$|ZIF-62 after different temperature treatments consisting of a heating segment from room temperature up to a maximum temperature, followed by an isothermal segment of several minutes and subsequent cooling back to room temperature. The heating and cooling were performed at a constant rate of 10 °C min$^{-1}$. The maximum temperature and time of the isothermal segment are given next to the corresponding PXRD patterns of the materials after the temperature treatment. The PXRD patterns were recorded with CuK$_{\alpha}$ radiation under air. The black pattern (bottom) corresponds to a simulated diffraction pattern of ZIF-zni (CCDC: IMIDZB).

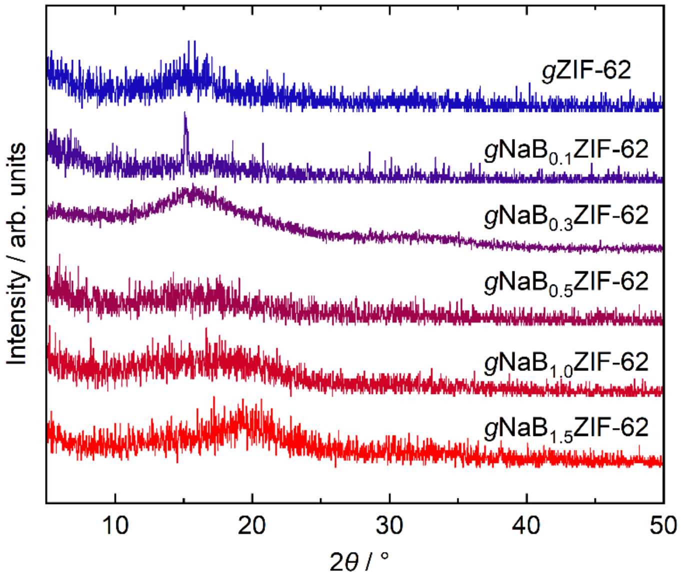


**Supplementary Figure 18.** PXRD patterns of $g$NaB$_x$ZIF-62 materials after the melt-quenching procedure performed in a DSC apparatus (~10 mg scale) consisting of a heating segment from room temperature up to 450 °C, followed by an isothermal segment of 30 mins and subsequent cooling back to room temperature. The heating and cooling were performed at a constant rate of 10 °C min$^{-1}$. Diffraction patterns were recorded with CuK$_\alpha$ radiation under air.

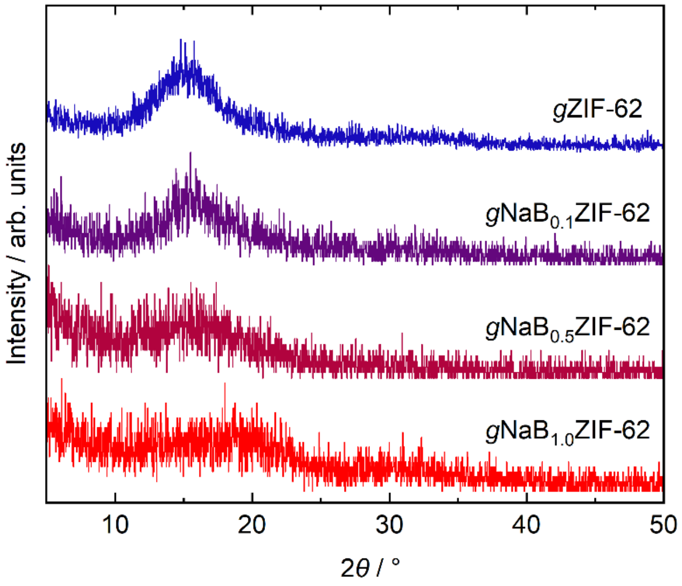


**Supplementary Figure 19.** PXRD patterns of $g$NaB$_x$-ZIF-62 materials after the melt-quenching procedure in the autoclave approach (~250 mg scale) consisting of a heating segment from room temperature up to 450 °C, followed by an isothermal segment of 30 mins and subsequent cooling back to room temperature. The heating was performed at a constant rate of 10 °C min$^{-1}$ while cooling was performed under external airflow (cooling rate ca. -10 °C min$^{-1}$). Diffraction patterns were recorded with CuK$_\alpha$ radiation under air.

## S7. Solution NMR Spectroscopy

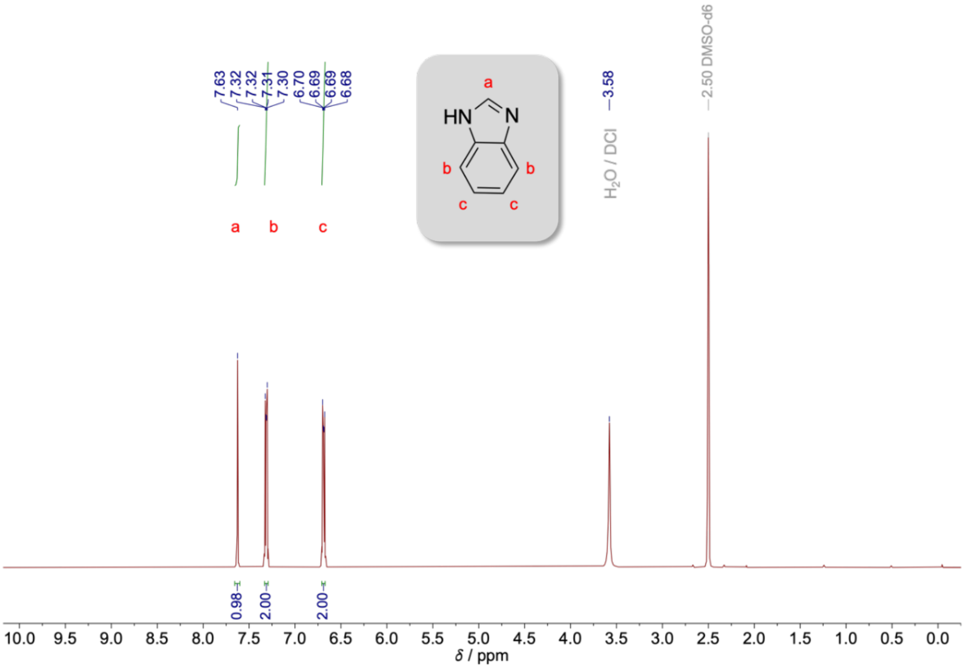


**Supplementary Figure 20.** $^{1}$H NMR spectrum of Na(bim) dissolved in DMSO-$d_6$ and DCl/$D_2O$ (35 wt%, one drop, <0.1 mL).

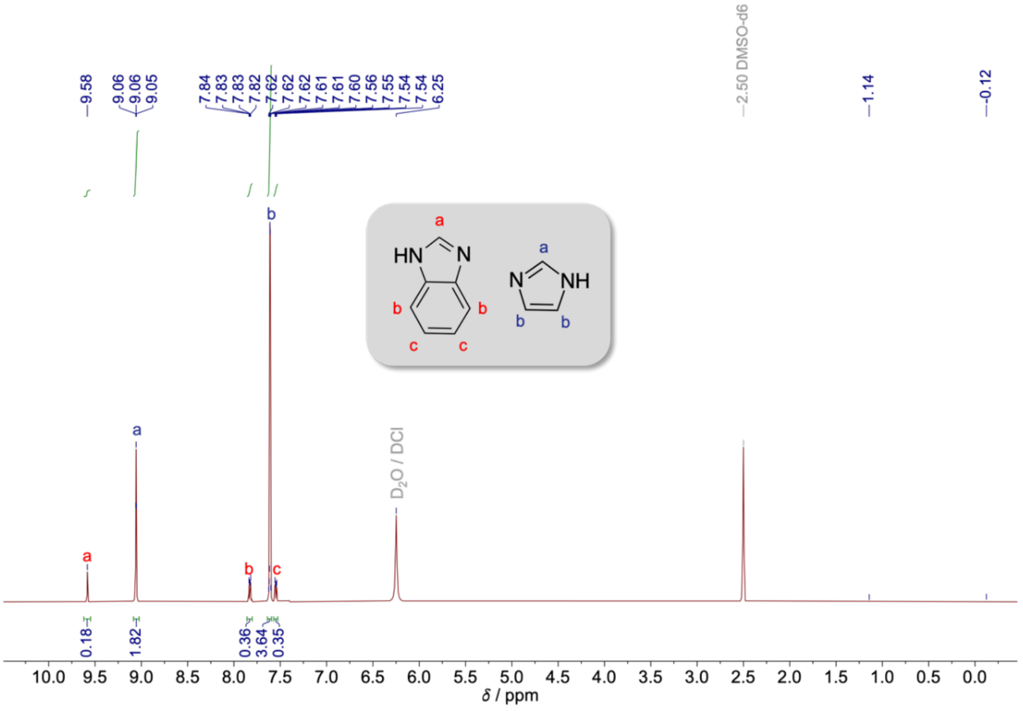


**Supplementary Figure 21.** $^{1}$H NMR spectrum of ZIF-62 dissolved in DMSO-$d_6$ and DCl/$D_2O$ (35 wt%, one drop, <0.1 mL).

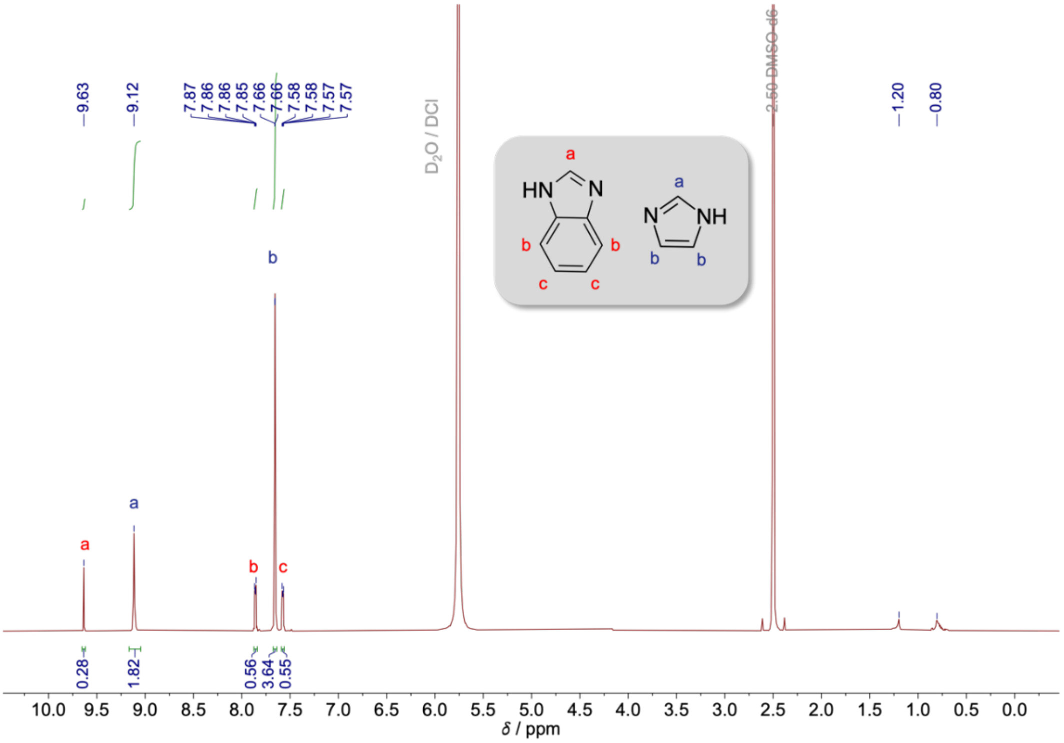


**Supplementary Figure 22.** $^{1}$H NMR spectrum of *g*NaB$_{0.1}$ZIF-62 (prepared in ~10 mg scale) dissolved in DMSO-$d_6$ and DCl/$D_2O$ (35 wt%, one drop, <0.1 mL). Signals at around 0.8 and 1.2 ppm are assigned to residues of *n*-hexane, which was added during the grinding of the obtained glass monolith.

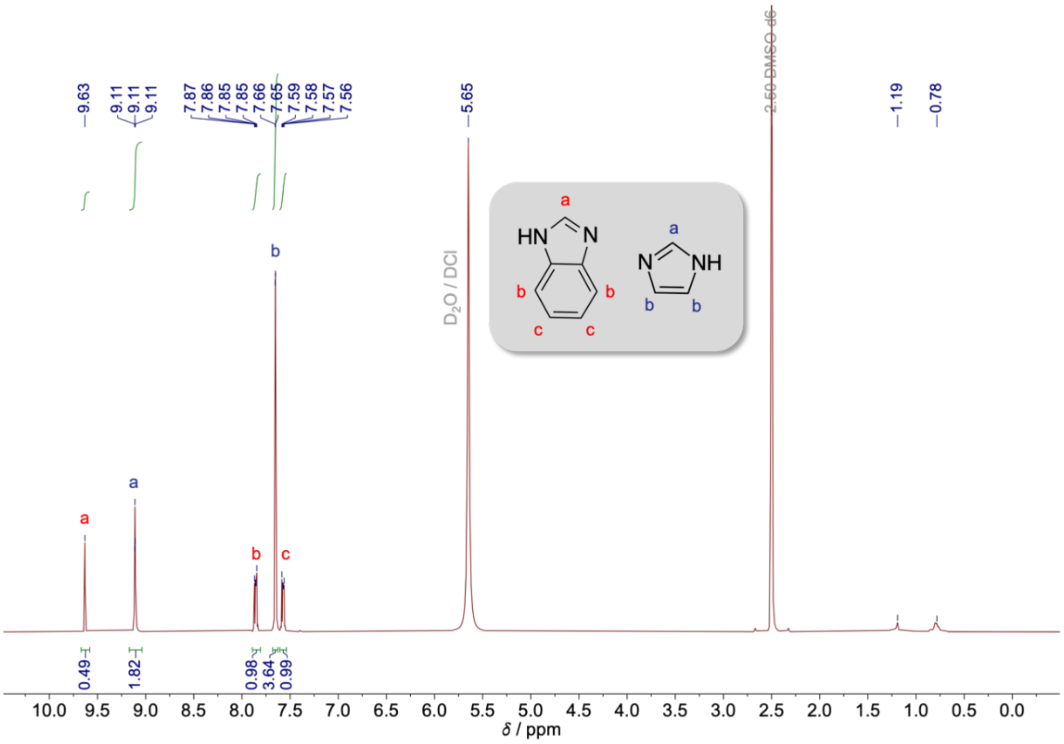


**Supplementary Figure 23.** $^{1}$H NMR spectrum of *g*NaB$_{0.3}$ZIF-62 (prepared in ~10 mg scale) dissolved in DMSO-$d_6$ and DCl/$D_2O$ (35 wt%, one drop, <0.1 mL). Signals at around 0.8 and 1.2 ppm are assigned to residues of *n*-hexane, which was added during the grinding of the obtained glass monolith.

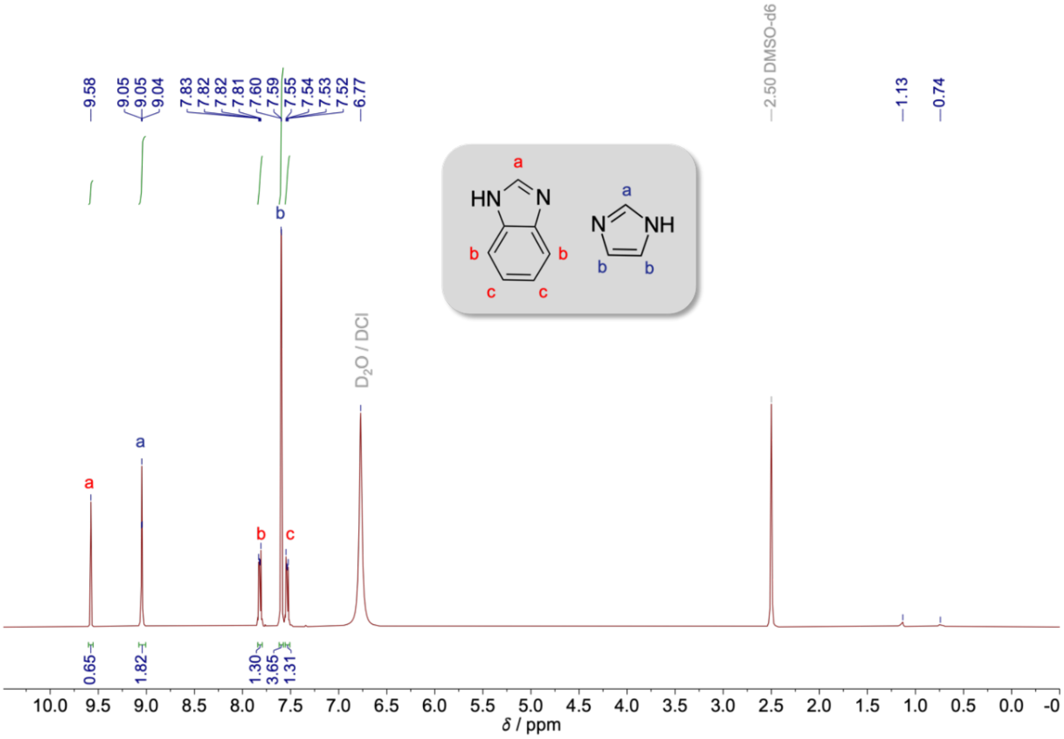


**Supplementary Figure 24.** $^{1}H$ NMR spectrum of *g*NaB$_{0.5}$ZIF-62 (prepared in ~10 mg scale) dissolved in DMSO-$d_6$ and DCl/$D_2O$ (35 wt%, one drop, <0.1 mL). Signals at around 0.8 and 1.2 ppm are assigned to residues of *n*-hexane, which was added during the grinding of the obtained glass monolith.

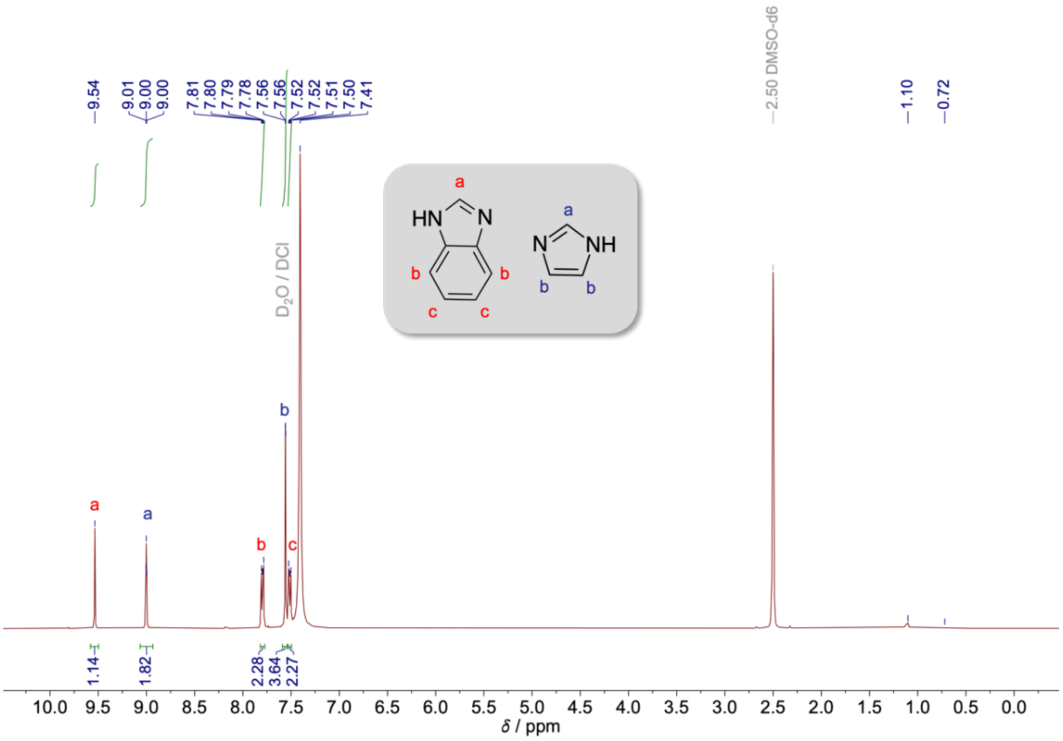


**Supplementary Figure 25.** $^{1}H$ NMR spectrum of *g*NaB$_{1.0}$ZIF-62 (prepared in ~10 mg scale) dissolved in DMSO-$d_6$ and DCl/$D_2O$ (35 wt%, one drop, <0.1 mL). Signals at around 0.8 and 1.2 ppm are assigned to residues of *n*-hexane, which was added during the grinding of the obtained glass monolith.

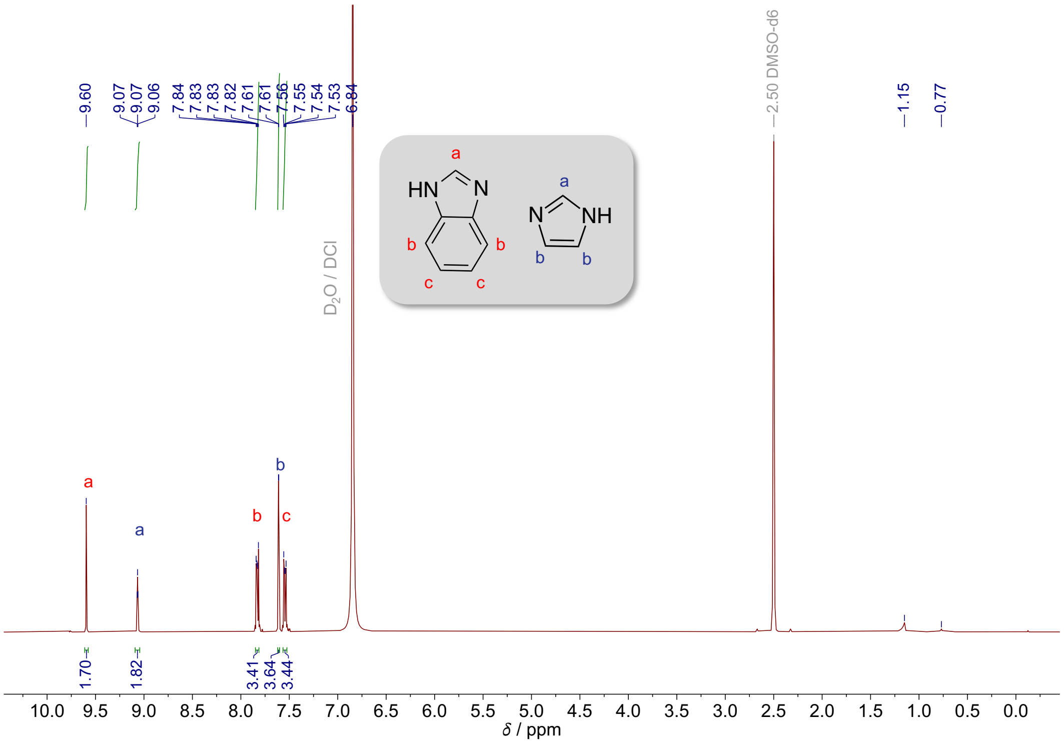

**Supplementary Figure 26.** $^1$H NMR spectrum of *g*NaB$_{1.5}$ZIF-62 (prepared in ~10 mg scale) dissolved in DMSO-$d_6$ and DCl/$D_2O$ (35 wt%, one drop, <0.1 mL). Signals at around 0.8 and 1.2 ppm are assigned to residues of *n*-hexane, which was added during the grinding of the obtained glass monolith.

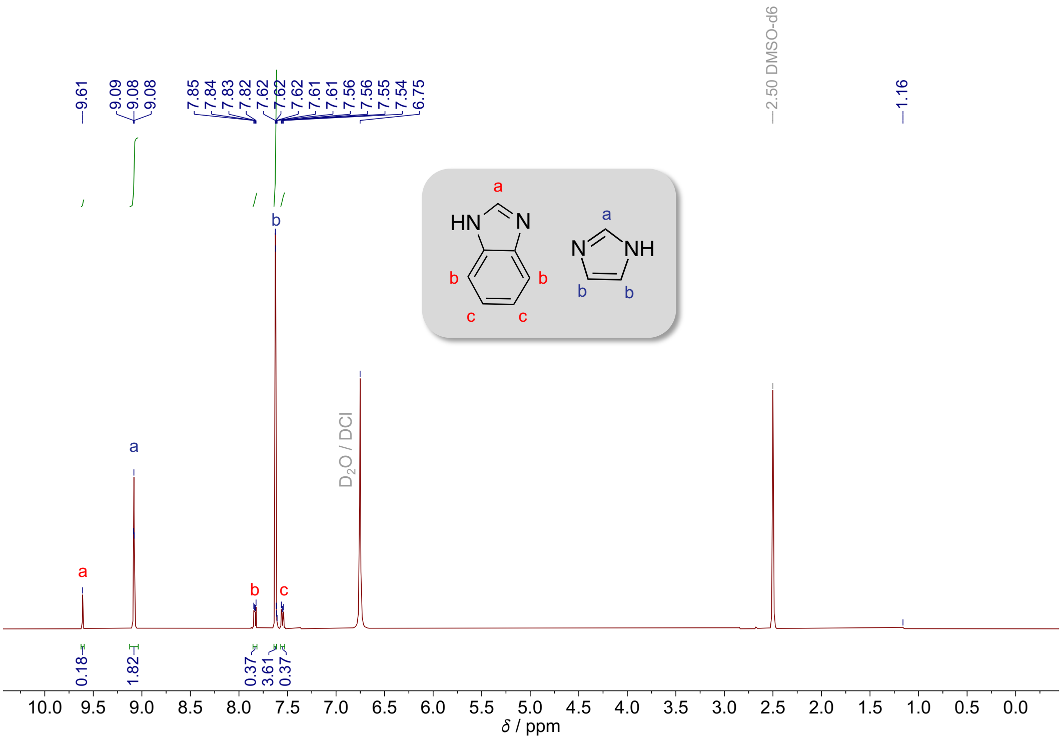

**Supplementary Figure 27.** $^1$H NMR spectrum of *g*ZIF-62 (prepared in ~250 mg scale) dissolved in DMSO-$d_6$ and DCl/$D_2O$ (35 wt%, one drop, <0.1 mL). Signals at around 0.8 and 1.2 ppm are assigned to residues of *n*-hexane, which was added during the grinding of the obtained glass monolith.

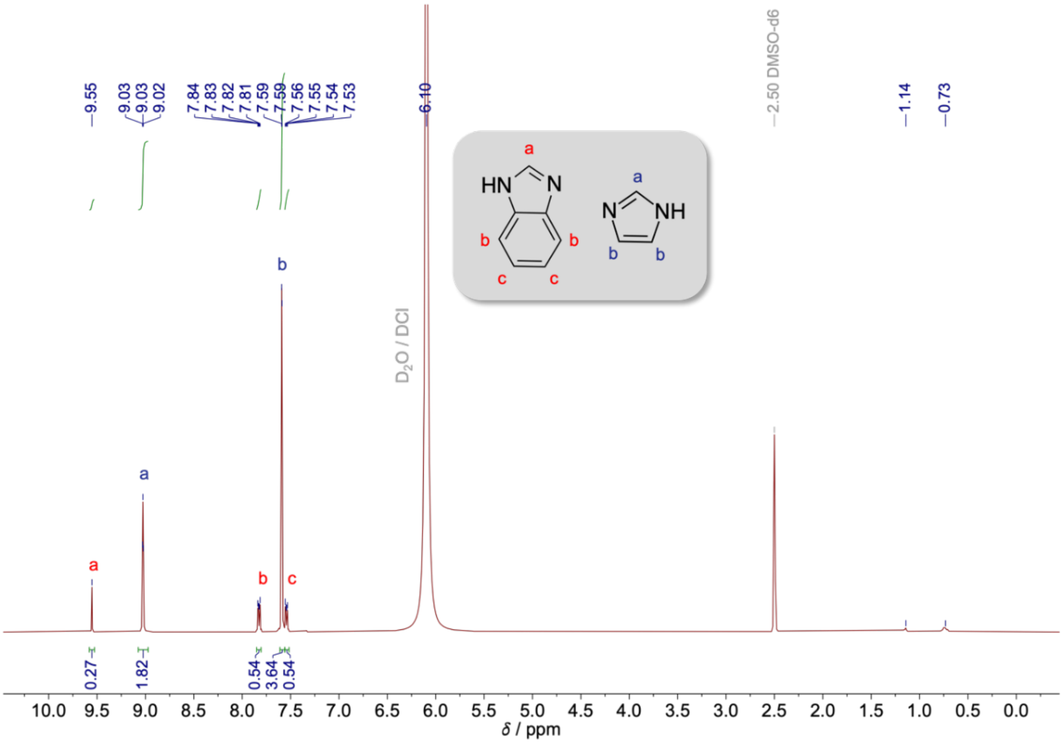


**Supplementary Figure 28.** $^{1}H$ NMR spectrum of *g*$NaB_{0.1}$ZIF-62 (prepared in ~250 mg scale) dissolved in DMSO-$d_6$ and $DCl/D_2O$ (35 wt%, one drop, <0.1 mL). Signals at around 0.8 and 1.2 ppm are assigned to residues of *n*-hexane, which was added during the grinding of the obtained glass monolith.

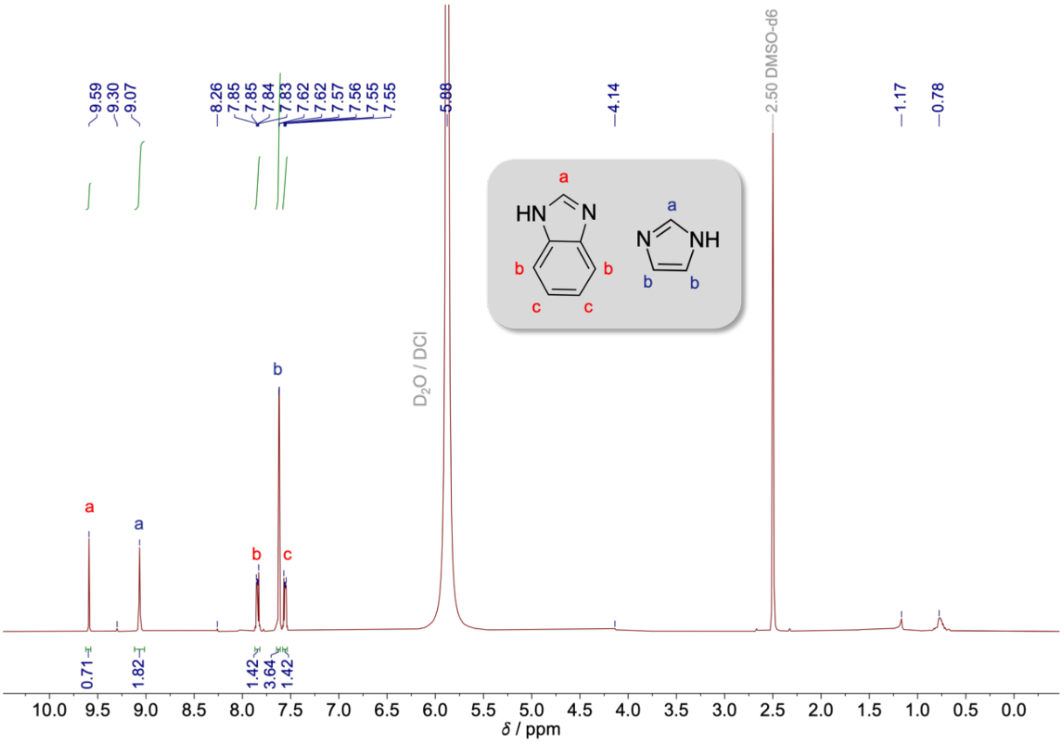


**Supplementary Figure 29.** $^{1}H$ NMR spectrum of *g*$NaB_{0.5}$ZIF-62 (prepared in ~250 mg scale) dissolved in DMSO-$d_6$ and $DCl/D_2O$ (35 wt%, one drop, <0.1 mL). Signals at around 0.8 and 1.2 ppm are assigned to residues of *n*-hexane, which was added during the grinding of the obtained glass monolith.

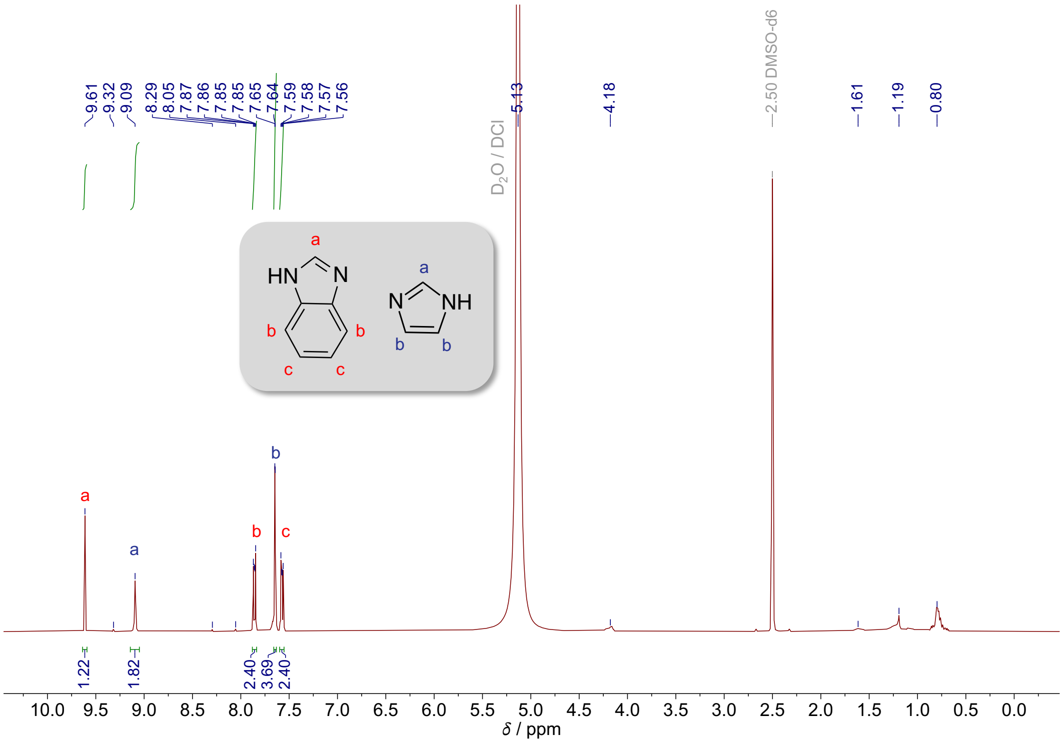


**Supplementary Figure 30.** $^1$H NMR spectrum of *g*NaB$_{1.0}$ZIF-62 (prepared in ~250 mg scale) dissolved in DMSO-$d_6$ and DCl/D$_2$O (35 wt%, one drop, <0.1 mL). Signals at around 0.8 and 1.2 ppm are assigned to residues of *n*-hexane, which was added during the grinding of the obtained glass monolith.

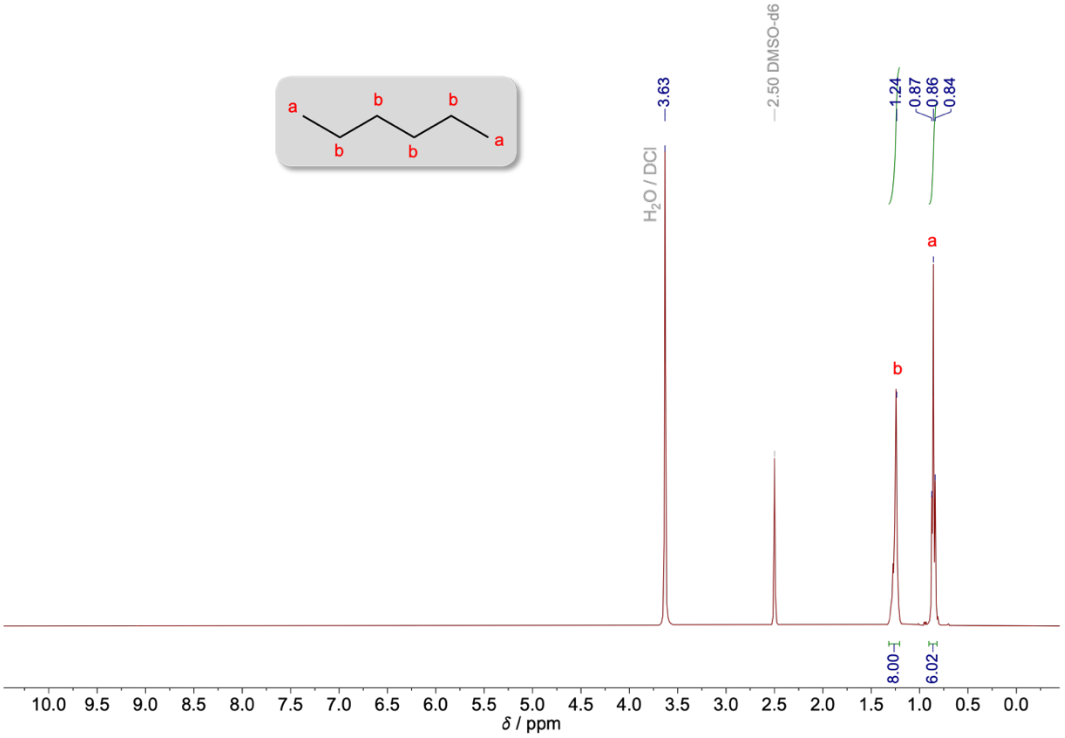


**Supplementary Figure 31.** $^1$H NMR spectrum of *n*-hexane dissolved in DMSO-$d_6$ and DCl/D$_2$O (35 wt%, one drop, <0.1 mL).

**Supplementary Table 4.** Name and chemical composition of prepared Na(bim)-modified ZIF-62 glasses *g*NaB$_x$ZIF-62 for different Na(bim) contents *x* and ZIF-62. Materials were obtained via two different approaches (i.e. small and large scale) and their chemical composition was calculated according to the bim-to-im ratio obtained from solution $^1$H NMR spectroscopic measurements of the glass samples digested in DMSO-$d_6$ and DCl/$D_2O$ (35 wt%, one drop, <0.1 mL). The thus calculated Na(bim) content *x*($^1$H NMR) does not necessarily coincide with the *x* used in the nomenclature (*g*NaB$_x$ZIF-62) due to rounding. It is assumed that the *x* values in the nomenclature are in accordance with the solution $^1$H NMR results with regard to the error margin of the NMR measurement. The chemical composition of the corresponding physical mixtures NaB$_x$|ZIF-62 is expected to be identical to those of the respective glasses. Deviations between the intended chemical composition and the composition found in $^1$H NMR spectroscopy may be attributed to weighting errors during the preparation of the physical mixtures and measurement inaccuracies of $^1$H NMR spectroscopy.

| Approach | Name | bim content | im content | Chemical composition | *x*($^1$H NMR) |
|---|---|---|---|---|---|
| Small Scale (~ 10 mg) | ZIF-62 | 0.18 | 1.82 | $Zn(im)_{1.82}(bim)_{0.18}$ | - |
| | *g*NaB$_{0.1}$ZIF-62 | 0.28 | | $Na_{0.10}Zn(im)_{1.82}(bim)_{0.28}$ | 0.10 |
| | *g*NaB$_{0.3}$ZIF-62 | 0.49 | | $Na_{0.31}Zn(im)_{1.82}(bim)_{0.49}$ | 0.31 |
| | *g*NaB$_{0.5}$ZIF-62 | 0.65 | | $Na_{0.47}Zn(im)_{1.82}(bim)_{0.65}$ | 0.47 |
| | *g*NaB$_{1.0}$ZIF-62 | 1.14 | | $Na_{0.96}Zn(im)_{1.82}(bim)_{1.14}$ | 0.96 |
| | *g*NaB$_{1.5}$ZIF-62 | 1.70 | | $Na_{1.52}Zn(im)_{1.82}(bim)_{1.70}$ | 1.52 |
| Large Scale (~ 250 mg) | *g*ZIF-62 | 0.18 | 1.82 | $Zn(im)_{1.82}(bim)_{0.18}$ | - |
| | *g*NaB$_{0.1}$ZIF-62 | 0.27 | | $Na_{0.09}Zn(im)_{1.82}(bim)_{0.27}$ | 0.09 |
| | *g*NaB$_{0.5}$ZIF-62 | 0.71 | | $Na_{0.53}Zn(im)_{1.82}(bim)_{0.71}$ | 0.53 |
| | *g*NaB$_{1.0}$ZIF-62 | 1.22 | | $Na_{1.04}Zn(im)_{1.82}(bim)_{1.22}$ | 1.04 |

# S8. Variable Temperature Powder X-Ray Diffraction

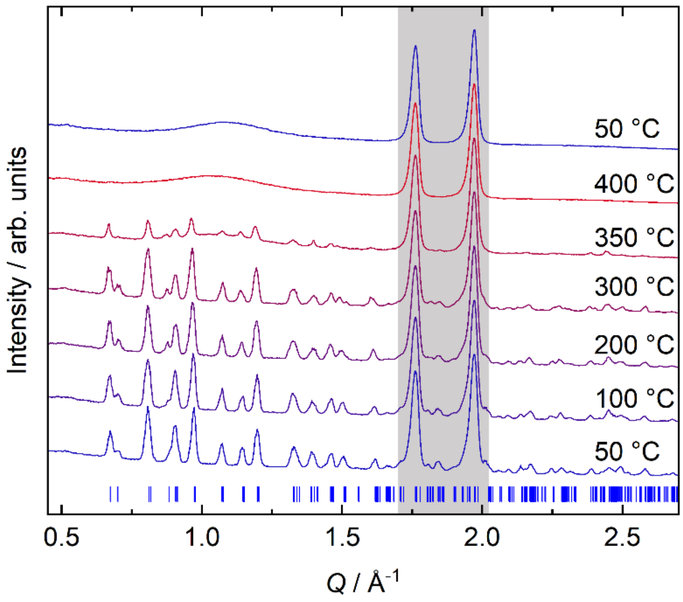


**Supplementary Figure 32.** VT-PXRD patterns of a heating-cooling-cycle of ZIF-62 recorded with λ = 0.4568 Å at DELTA starting with the heating from 50 °C (blue, bottom) up to 400 °C (red) and subsequent cooling back to 50 °C (blue, top). The greyed-out reflections are related to the used experimental set up and do not originate from the sample material. The blue tick marks correspond to the allowed Bragg peak positions of ZIF-62 (CCDC: GIZJOP).

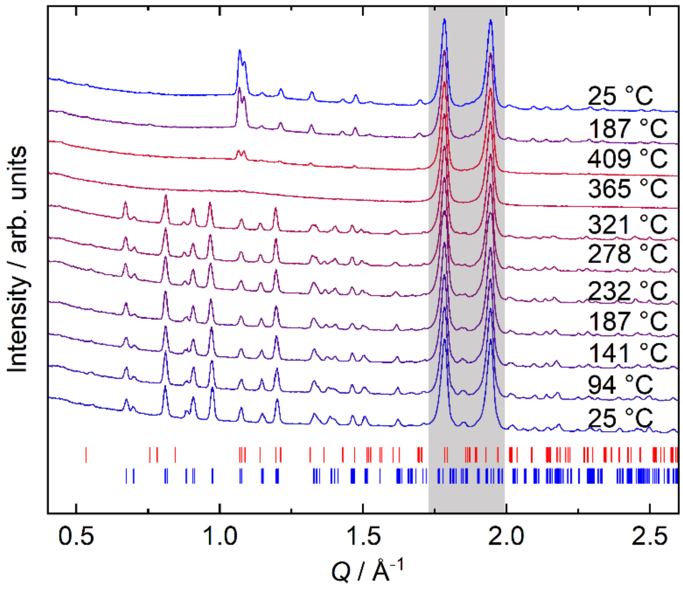


**Supplementary Figure 33.** VT-PXRD patterns of a heating-cooling-cycle of $NaB_{0.1}$|ZIF-62 recorded with λ = 0.4616 Å at DELTA starting with the heating from 25 °C (blue, bottom) up to 409 °C (red) and subsequent cooling back to 25 °C (blue, top). The tick marks correspond to the allowed Bragg peak positions of ZIF-62 (blue) and ZIF-zni (red) (CCDC: GIZJOP, IMIDZB). The greyed-out reflections are related to the used experimental set up and do not originate from the sample material.

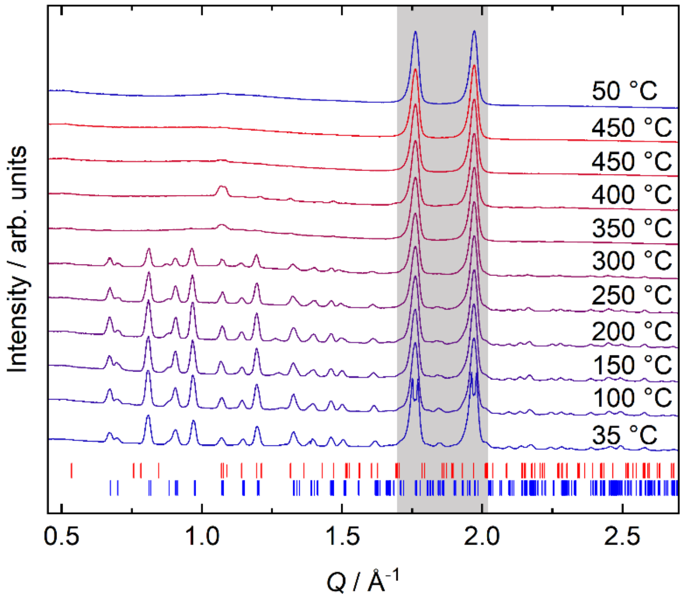


**Supplementary Figure 34.** VT-PXRD patterns of a heating-cooling-cycle of $NaB_{0.3}$|ZIF-62 recorded with λ = 0.4568 Å at DELTA starting with the heating from 35 °C (blue, bottom) up to 450 °C (red) followed by an isothermal segment (2nd 450 °C measurement) and subsequent cooling back to 50 °C (blue, top). The tick marks correspond to the allowed Bragg peak positions of ZIF-62 (blue) and ZIF-zni (red). The latter crystallises at 350 °C and dissolves again above 400 °C (CCDC: GIZJOP, IMIDZB). The greyed-out reflections are related to the used experimental set up and do not originate from the sample material.

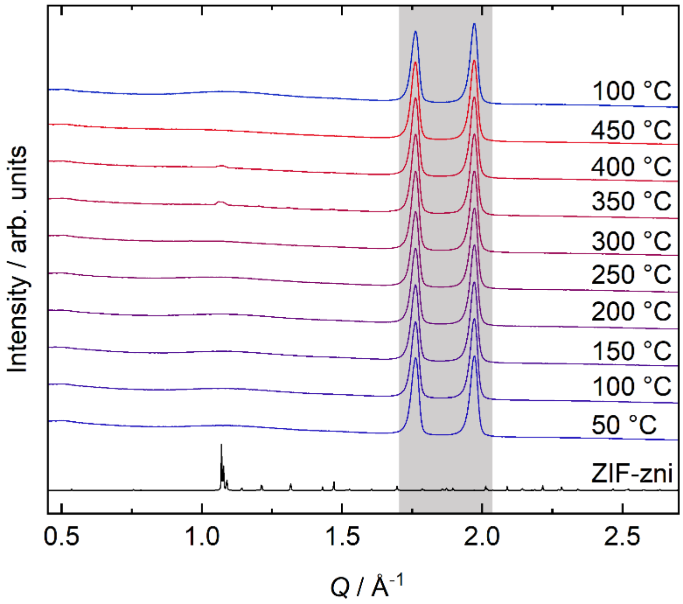


**Supplementary Figure 35.** VT-PXRD patterns of a heating-cooling-cycle of *g*$NaB_{0.3}$ZIF-62 recorded with λ = 0.4568 Å at DELTA starting with the heating from 50 °C (blue, bottom) up to 450 °C (red) and subsequent cooling back to 100 °C (blue, top). The black line corresponds to a simulated diffraction pattern of ZIF-zni (bottom) (CCDC: IMIDZB). The greyed-out reflections are related to the used experimental set-up and do not originate from the sample material. The depicted VT-PXRD data show the intermediate crystallisation of ZIF-zni during the heating of *g*$NaB_{0.3}$ZIF-62 starting at 350 °C and the subsequent dissolution of the crystalline phase at even higher temperatures.

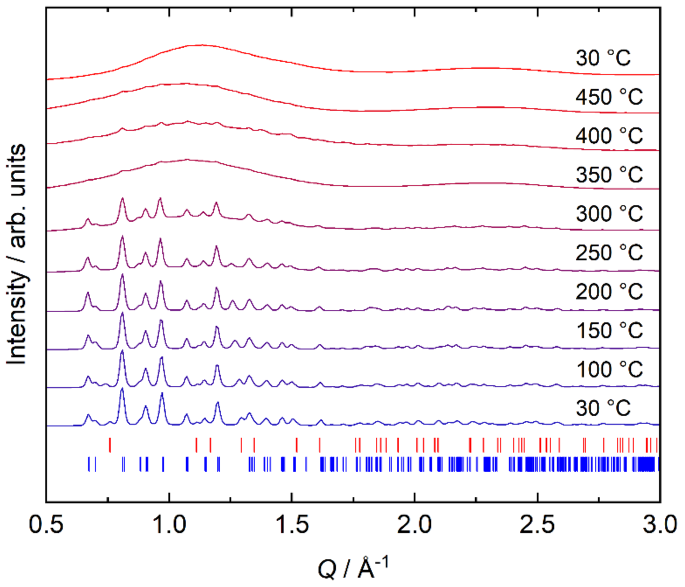


**Supplementary Figure 36.** VT-PXRD patterns of a heating-cooling-cycle of $NaB_{0.5}$|ZIF-62 recorded with λ = 0.1616 Å at DLS starting with the heating from 30 °C (blue, bottom) up to 450 °C (red) and subsequent cooling back to 30 °C (blue, top). The tick marks correspond to the allowed Bragg peak positions of ZIF-62 (blue) and α-Na(bim) (red) (CCDC: GIZJOP, QOVDAK). The intermediate appearance of reflections in the pattern collected at 400 °C is likely caused by residual microcrystalline ZIF-62 from the unheated part of the capillary moving into the beam path via the flow of the (amorphous) liquid.

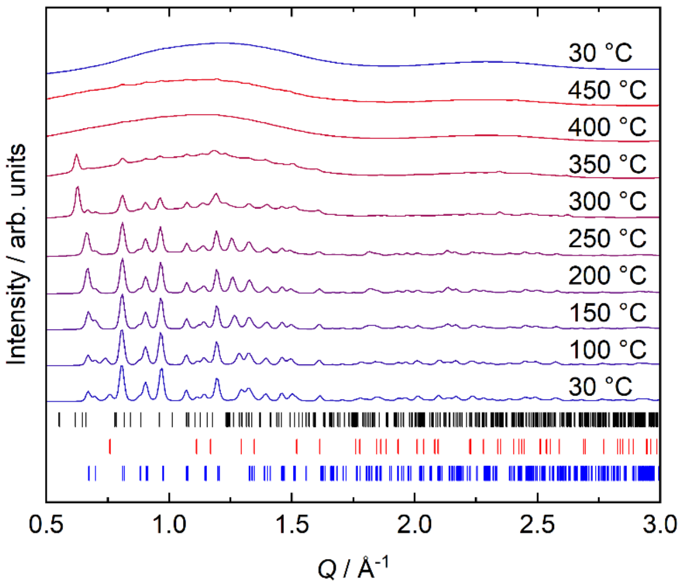


**Supplementary Figure 37.** VT-PXRD patterns of a heating-cooling-cycle of $NaB_{1.0}$|ZIF-62 recorded with λ = 0.1616 Å at DLS starting with the heating from 30 °C (blue, bottom) up to 450 °C (red) and subsequent cooling back to 30 °C (blue, top). The tick marks indicate the allowed Bragg peak positions of ZIF-62 (blue), α-Na(bim) (red) as well as ZIF-7-III (black) after crystallisation at 300 °C (CCDC: GIZJOP, QOVDAK, KOLYAM).

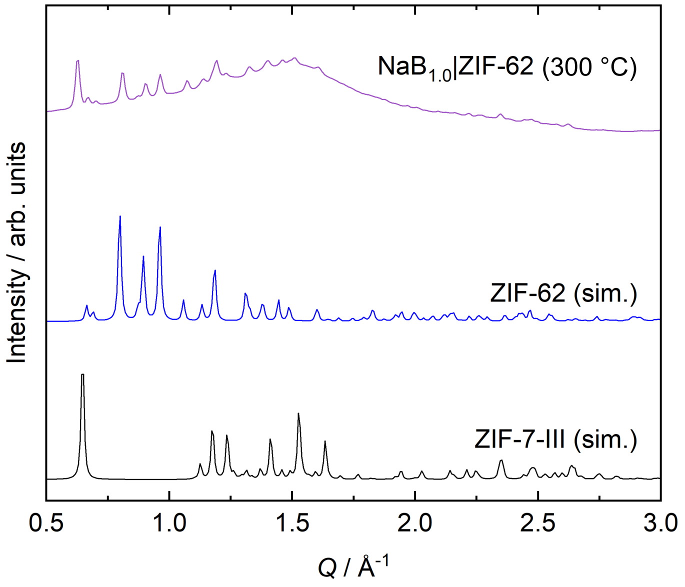


**Supplementary Figure 38.** VT-PXRD patterns of $NaB_{1.0}$|ZIF-62 recorded at 300 °C with λ = 0.1616 Å at DLS, together with simulated diffraction patterns of ZIF-62 (blue) and ZIF-7-III (black) demonstrating the presence of residual ZIF-62 and crystallised ZIF-7-III in the physical mixture during the thermal upscan shown in Supplementary Figure 37 (CCDC: GIZJOP, KOLYAM). The first reflection of ZIF-7-III (*hkl* = 002) is slightly shifted compared to the first reflection observed in the pattern of $NaB_{1.0}$|ZIF-62 due to the thermal expansion of the crystalline phase at elevated temperatures.

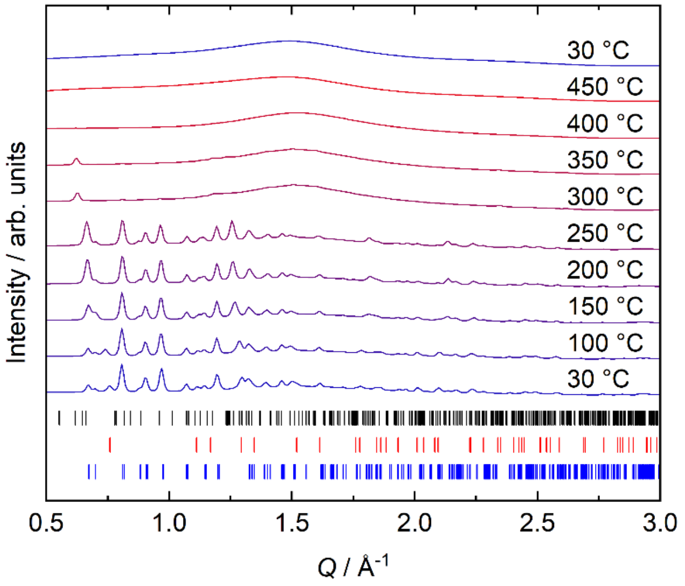


**Supplementary Figure 39.** VT-PXRD patterns of a heating-cooling-cycle of $NaB_{1.5}$|ZIF-62 recorded with λ = 0.1616 Å at DLS starting with the heating from 30 °C (blue, bottom) up to 450 °C (red) and subsequent cooling back to 30 °C (blue, top). The tick marks correspond to the allowed Bragg peak positions of ZIF-62 (blue) and α-Na(bim) (red) as well as ZIF-7-III (black) which crystallises at 300 °C followed by dissolution of the formed crystalline phase at 400 °C (CCDC: GIZJOP, QOVDAK, KOLYAM).

## S9. Differential Scanning Calorimetry

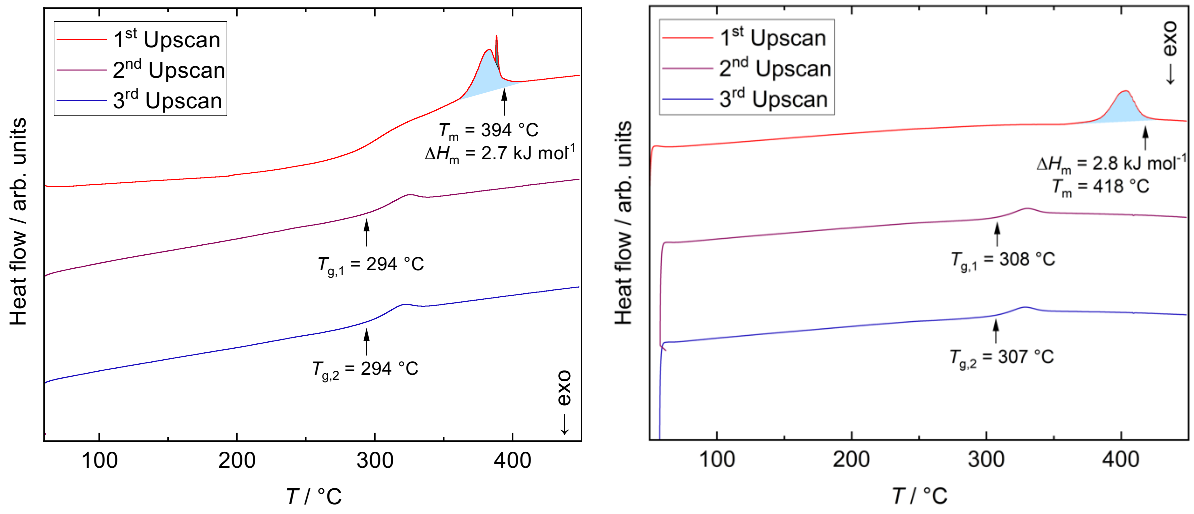


**Supplementary Figure 40.** Heat flow curves of consecutive thermal upscans of ZIF-62 measured under hermetic conditions in a sealed crucible (left) and in a perforated one (right). The autogenous pressure build-up leads to artefacts in the first thermal upscan, which are assigned to the deformation of the crucible (left). Further, the thermal events of melting ($T_m$) and the glass transition ($T_g$) appear at lower temperatures (left) compared to the measurement performed under atmosphere pressure (right). Heating and cooling were performed at a constant rate of ±10 °C min$^{-1}$.

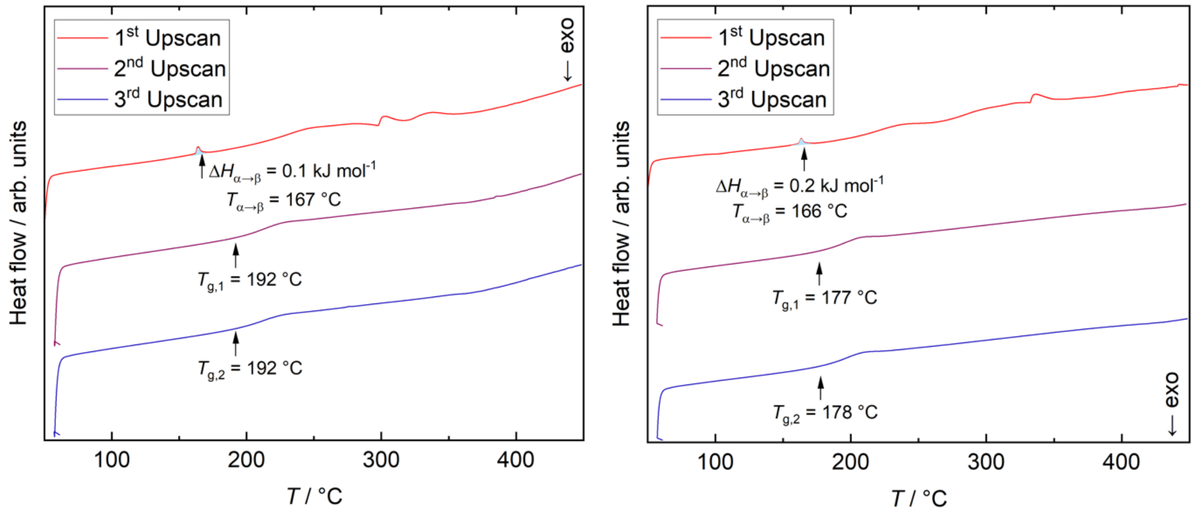


**Supplementary Figure 41.** Heat flow curves of consecutive thermal upscans of the physical mixture $NaB_{0.1}$|ZIF-62 (left) and $NaB_{0.3}$|ZIF-62 (right). An isothermal segment of 30 mins was applied after the first upscan to improve homogenization. Heating and cooling were performed at a constant rate of ±10 °C min$^{-1}$. Enthalpies $\Delta H$ were calculated based on the mean molar mass of the corresponding physical mixture. Sharp features in the first upscan beyond 350 °C are considered artefacts resulting from crucible deformation under the autogenous pressure build-up. The arrows indicate the temperature of the glass transition $T_g$, the temperature of the α→β phase transition of pure Na(bim) in the physical mixture $T_{\alpha\rightarrow\beta}$ and the temperature of the intermixing process $T_{mix}$ during the reaction of ZIF-62 with Na(bim)..

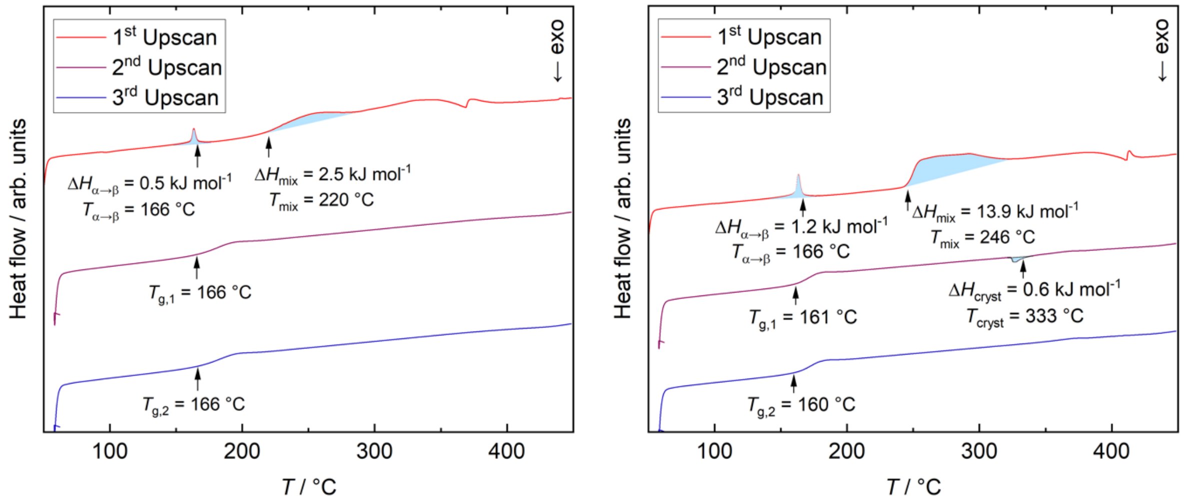


**Supplementary Figure 42.** Heat flow curves of consecutive thermal upscans of the physical mixture $NaB_{0.5}$|ZIF-62 (left) and $NaB_{1.0}$|ZIF-62 (right). An isothermal segment of 30 mins was applied after the first upscan to improve homogenization. Heating and cooling were performed at a constant rate of ±10 °C $min^{-1}$. Enthalpies $\Delta H$ were calculated based on the mean molar mass of the corresponding physical mixture. Sharp features in the first upscan beyond 350 °C are considered artefacts resulting from crucible deformation under the autogenous pressure build-up. The arrows indicate the temperature of the glass transition $T_g$, the temperature of the α→β phase transition of pure Na(bim) in the physical mixture $T_{\alpha\rightarrow\beta}$ and the temperature of the intermixing process $T_{mix}$ during the reaction of ZIF-62 with Na(bim).

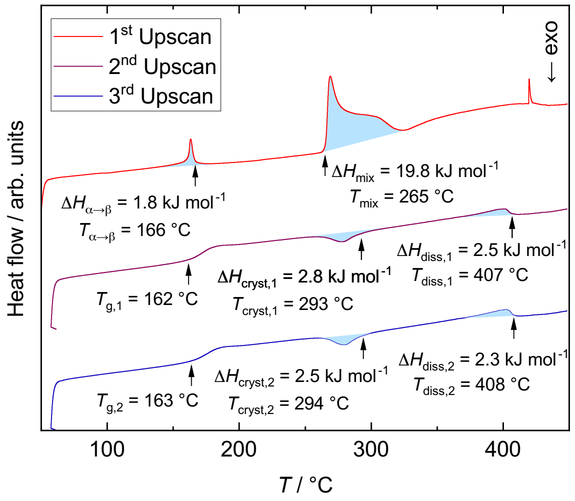


**Supplementary Figure 43.** Heat flow curves of consecutive thermal upscans of the physical mixture $NaB_{1.5}$|ZIF-62. An isothermal segment of 30 mins was applied after the first upscan to improve homogenization. Heating and cooling were performed at a constant rate of ±10 °C $min^{-1}$. Enthalpies $\Delta H$ were calculated based on the mean molar mass of the corresponding physical mixture. Endothermic features in the first upscan beyond 350 °C are considered artefacts resulting from crucible deformation under the autogenous pressure build-up. The arrows indicate the temperatures of the glass transition $T_g$, the temperature of the α→β phase transition of pure Na(bim) in the physical mixture $T_{\alpha\rightarrow\beta}$ or the temperature of the intermixing process $T_{mix}$ during the reaction of ZIF-62 with Na(bim) in the first thermal upscan. $T_{cryst}$ and $T_{diss}$ correspond to the crystallization and subsequent dissolution process of the intermediately formed crystalline ZIF-7-III phase in the second and third thermal upscan.

**Supplementary Table 5.** Enthalpies associated with the $\alpha \rightarrow \beta$ phase transition of Na(bim) ($\Delta H_{\alpha\rightarrow\beta}$) in the first thermal upscan of the physical mixtures with different Na(bim) contents x as well as the enthalpy which is assigned to the process of intermixing ($\Delta H_{mix}$) during the reaction of ZIF-62 and Na(bim) in the first thermal upcan in the corresponding DSC thermograms (see Supplementary Figure 40 – Supplementary Figure 43). The extracted molar enthalpies are calculated based on the molar unit $ZnNa_x(im)_{1.8}(bim)_{0.2+x}$. The obtained $\Delta H_{\alpha\rightarrow\beta}$ values ($\Delta H_{\alpha\rightarrow\beta}$(Na(bim)) = 1.3 kJ mol$^{-1}$)[7] are in rough accordance with the amount of Na(bim) in the physical mixtures as reflected by the solution $^1$H NMR spectroscopy results.

| $x$ | $\Delta H_{a\rightarrow b}$ / J g$^{-1}$ | $\Delta H_{\alpha\rightarrow\beta}$ / kJ mol$^{-1}$ | $\Delta H_{mix}$ / J g$^{-1}$ | $\Delta H_{mix}$ / kJ mol$^{-1}$ |
|---|---|---|---|---|
| 0.1 | 0.5 | 0.1 | - | - |
| 0.3 | 0.8 | 0.2 | - | - |
| 0.5 | 1.7 | 0.5 | 9.0 | 2.5 |
| 1.0 | 3.6 | 1.2 | 40.4 | 13.9 |
| 1.5 | 4.3 | 1.8 | 47.0 | 19.7 |

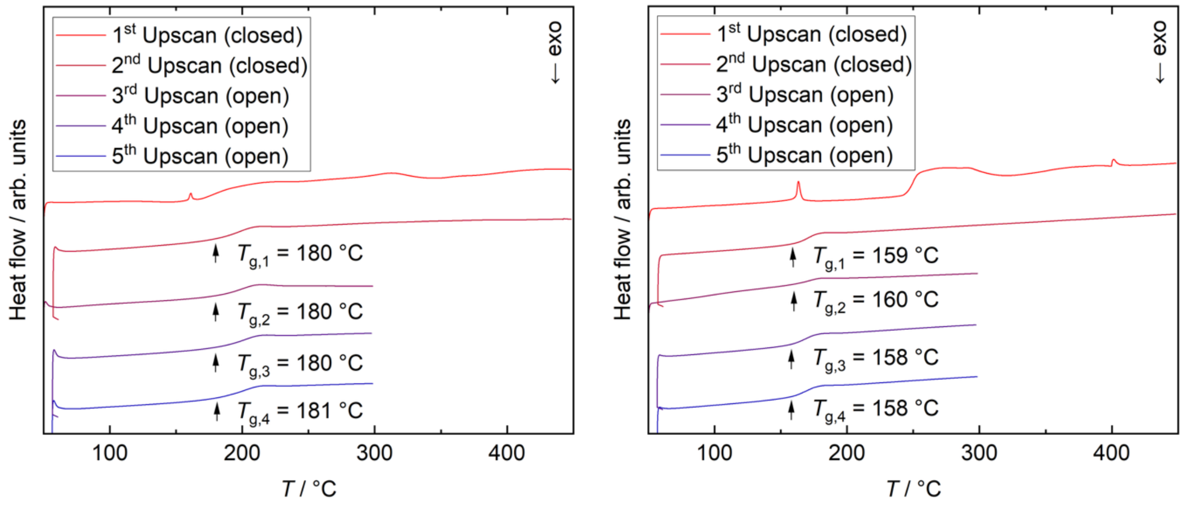


**Supplementary Figure 44.** Heat flow curves of consecutive thermal upscans of the physical mixture $NaB_{0.3}$|ZIF-62 (left) and $NaB_{1.0}$|ZIF-62 (right). An isothermal segment of 30 minutes was applied after the first upscan to improve homogenization of the melt. To investigate the influence of the autogenous pressure build-up in the hermetically sealed crucible, only the first two upscans were collected in a sealed crucible (closed) while the 3$^{rd}$ to 5$^{th}$ upscans were collected with a pierced crucible lid (open). Heating and cooling were performed at a constant rate of ±10 °C min$^{-1}$. Sharp features in the first upscan beyond 350 °C are considered artefacts resulting from crucible deformation under the autogenous pressure build-up. The arrows indicate the temperature of the glass transition $T_g$, and monitor only minor changes in the glass transition temperature between the first two and the following upscans at ambient pressure.

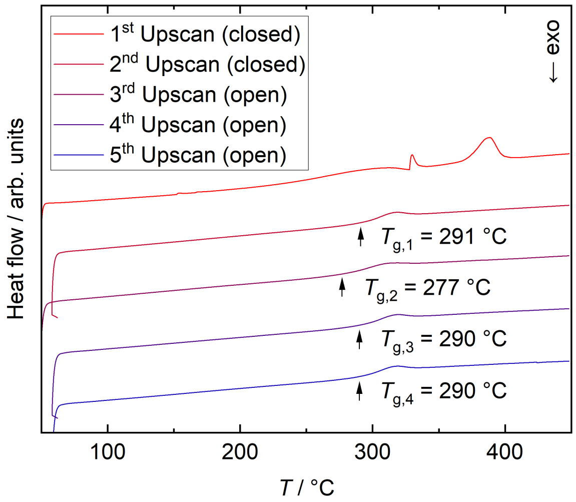


**Supplementary Figure 45.** Heat flow curves of consecutive thermal upscans of ZIF-62. An isothermal segment of 30 minutes was applied after the first upscan to improve homogenization of the melt. To investigate the influence of the autogenous pressure build-up in the hermetically sealed crucible, only the first two upscans were collected in a sealed crucible (closed) while the 3$^{rd}$ to 5$^{th}$ upscans were collected with a pierced crucible lid (open). Heating and cooling were performed at a constant rate of ±10 °C min$^{-1}$. Sharp features in the first upscan are considered artefacts resulting from crucible deformation under the autogenous pressure build-up. The arrows indicate the temperature of the glass transition $T_g$, and monitor a decrease in $T_g$ after opening of the crucible (3$^{rd}$ upscan). After the relaxation of the glass (i.e. after undergoing $T_{g,2}$), the glass transition temperature increases in upscans 4 and 5 back to the initial value of 290 °C.

## S9.1. Calorimetric Fragility Measurements

### Experimental Details

All DSC experiments were performed on the DSC25 from TA Instruments under a constant $N_2$ flow of 50 mL $min^{-1}$. Data processing was carried out in TA Instruments TRIOS version 5.1.1.46572.

Calorimetric fragility measurements were performed according to the experimental protocol first published by Zheng et al.[8] For the measurement, a powdered sample was filled in an aluminium crucible which was hermetically sealed under an inert atmosphere ($N_2$). The sample was subject to a temperature program consisting of an initial vitrification process (heating to 450 °C, followed by an isothermal segment of 30 min at the maximum temperature), followed by the actual fragility measurement procedure. For this, the sample was subject to several consecutive cooling and heating cycles each with a different but constant cooling/heating rate ($q_c$) of ± 7, 10, 13, 16, 19 K $min^{-1}$. The fictive temperature $T_f$, as well as the glass transition temperature $T_g$ was defined as the inflexion point of the heat flow curve during the glass transition. After the measurement, the obtained glass was subject to PXRD measurements to make sure that the sample was not altered during the temperature program applied for fragility determination (e.g. by partial crystallization of ZIF-zni or ZIF-7-III). The liquid fragility index $m$ was obtained as the slope of a linear fit to the data plotted according to its definition given in Equation (1). The following equation further shows the relation between $m$ and the activation energy of viscous flow $E_a$ at $T_g$ and the ideal gas constant $R$:

$$m = \frac{\partial \log\left(\frac{1}{q_c}\right)}{\partial\left(\frac{T_g}{T_f}\right)} = \frac{1}{2.303} \cdot \frac{\partial \ln\left(\frac{1}{q_c}\right)}{\partial\left(\frac{T_g}{T_f}\right)} = \frac{E_a}{2.303 \cdot R \cdot T_g} \quad (1)$$

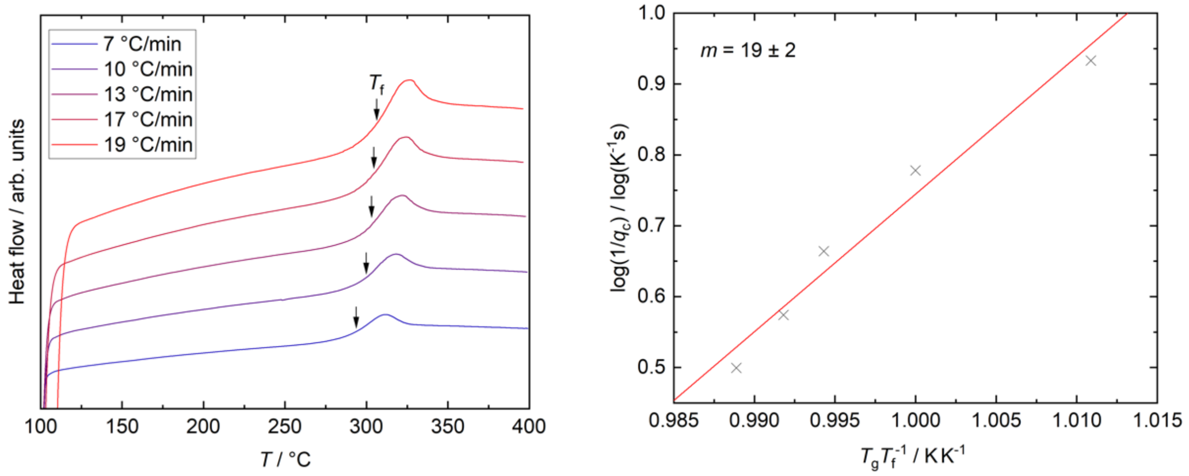


**Supplementary Figure 46.** Left: Consecutive heating branches with different heating rates in a cyclic DSC experiment of *g*ZIF-62. The black arrows indicate the determined fictive temperatures $T_f$ at the glass transition. Right: Linear regression performed on the data points extracted from the DSC experiments providing the calorimetric fragility index $m$ ($R^2$ = 0.97).

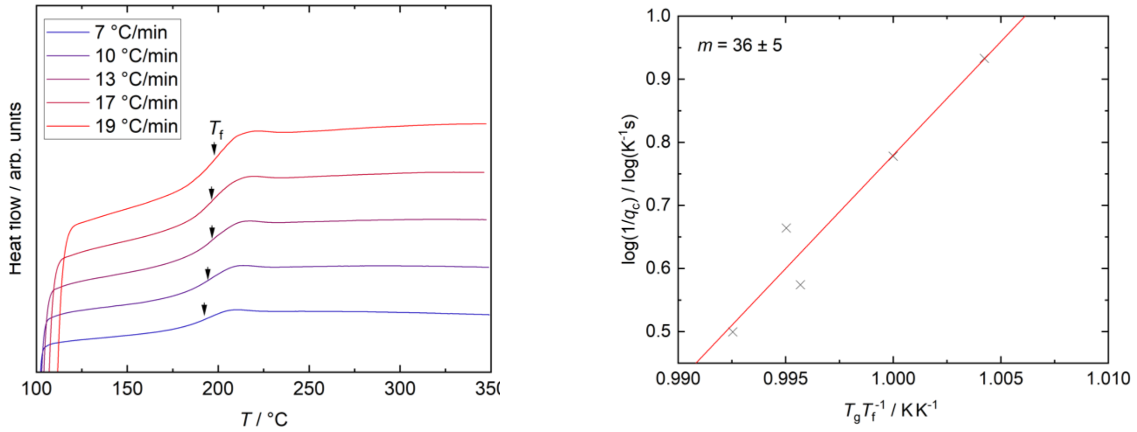


**Supplementary Figure 47.** Left: Consecutive heating branches with different heating rates in a cyclic DSC experiment of *g*NaB$_{0.3}$ZIF-62. The black arrows indicate the determined fictive temperatures $T_f$ at the glass transition. Right: Linear regression performed on the data points extracted from the DSC experiments providing the calorimetric fragility index $m$ ($R^2$ = 0.94).

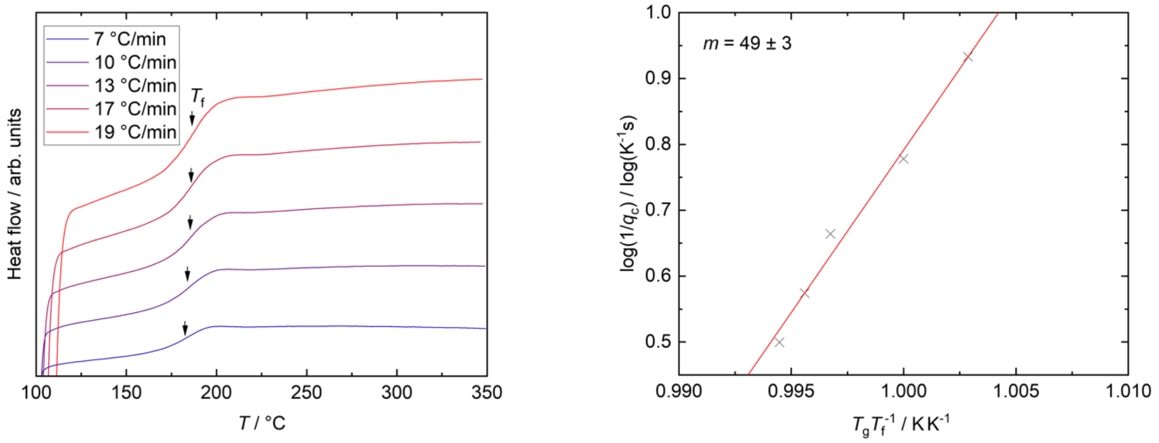


**Supplementary Figure 48.** Left: Consecutive heating branches with different heating rates in a cyclic DSC experiment of *g*$NaB_{0.5}$ZIF-62. The black arrows indicate the determined fictive temperatures $T_f$ at the glass transition. Right: Linear regression ($R^2$ = 0.96) performed on the data points extracted from the DSC experiments providing the calorimetric fragility index *m*.

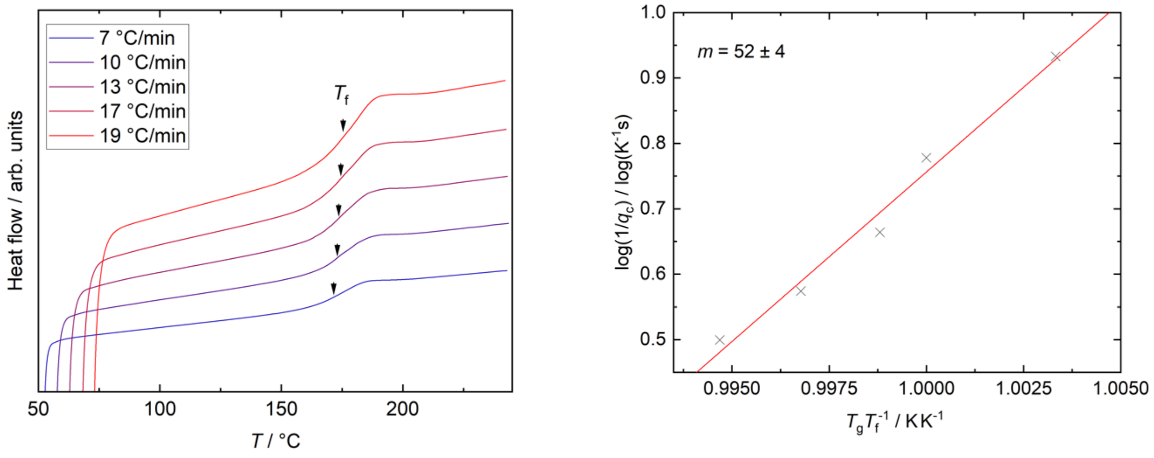


**Supplementary Figure 49.** Left: Consecutive heating branches with different heating rates in a cyclic DSC experiment of *g*$NaB_{1.0}$ZIF-62. The black arrows indicate the determined fictive temperatures $T_f$ at the glass transition. Right: Linear regression ($R^2$ = 0.98) performed on the data points extracted from the DSC experiments providing the calorimetric fragility index *m*.

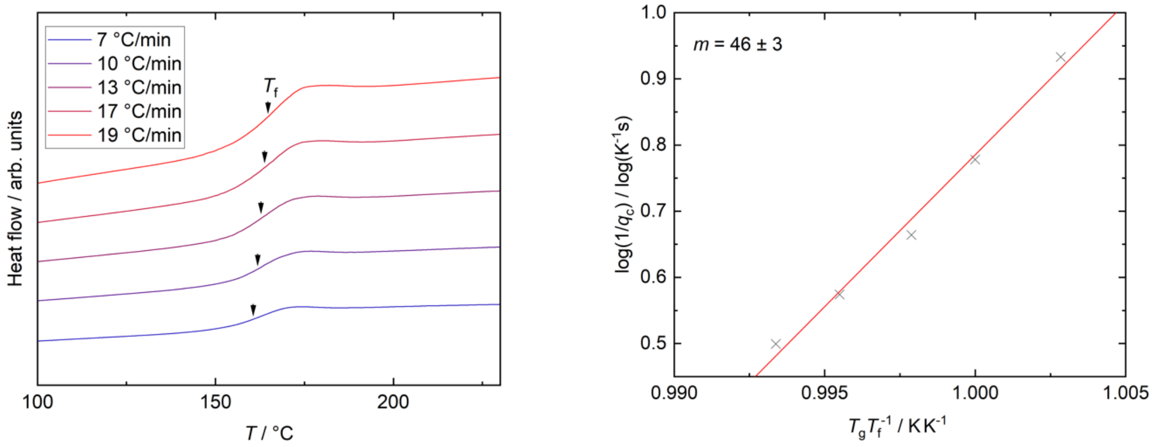


**Supplementary Figure 50.** Left: Consecutive heating branches with different heating rates in a cyclic DSC experiment of *g*NaB$_{1.5}$ZIF-62. The black arrows indicate the determined fictive temperatures $T_f$ at the glass transition. Right: Linear regression ($R^2$ = 0.99) performed on the data points extracted from the DSC experiments providing the calorimetric fragility index *m*.

.

**Supplementary Table 6.** Calorimetric fragility index *m* of Na(bim)-modified ZIF-62 glasses with the chemical formula ZnNa$_x$(im)$_{1.8}$(bim)$_{0.2+x}$, their glass transition temperature $T_g$ and the resulting activation energy of viscous flow $E_A$ at $T_g$ calculated according to Equation (1).

| *x* | m | $T_g$ (midpoint)/ °C | $E_A$ / kJ mol$^{-1}$ |
|---|---|---|---|
| 0 | 19 ± 2 | 299.9 | 213 ± 22 |
| 0.3 | 36 ± 5 | 194.3 | 322 ± 46 |
| 0.5 | 49 ± 3 | 183.9 | 432 ± 31 |
| 1 | 52 ± 4 | 173.0 | 443 ± 34 |
| 1.5 | 46 ± 3 | 161.9 | 382 ± 24 |

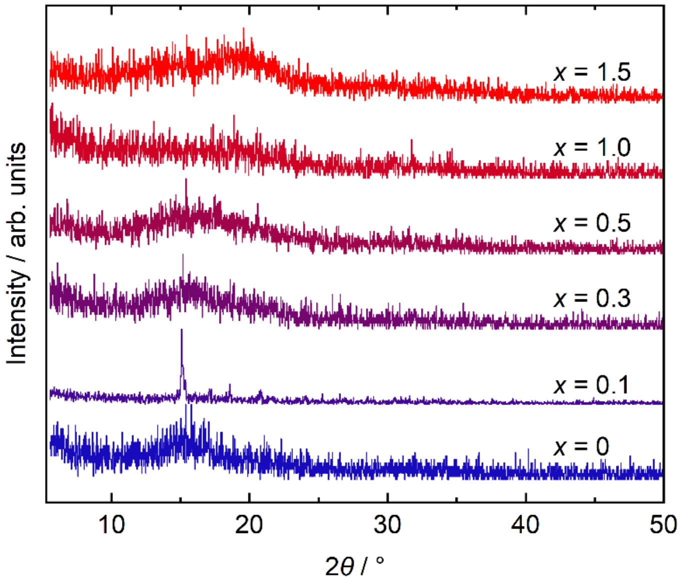


**Supplementary Figure 51.** PXRD pattern of *g*NaB$_x$ZIF-62 materials after undergoing the temperature program applied during the calorimetric fragility determination. The measurement was conducted using CuK$\alpha$ radiation and performed under air. The diffraction patterns show diffuse scattering for all glasses except for *g*NaB$_{0.1}$ZIF-62. Here, partial recrystallisation of ZIF-zni is observed. Thus, the calorimetric fragility index of *g*NaB$_{0.1}$ZIF-62 could not be determined, as the material experiences partial crystallization to ZIF-zni upon thermal cycling.

## S9.2. Heat Capacity Measurements

### Experimental Details

Reversing heat capacity measurements were performed on a DSC25 from TA instruments. The samples were ground thoroughly and placed in a hermetically sealed aluminium crucible under an inert $N_2$ atmosphere. All experiments were carried out under constant $N_2$ flow (50 mL min$^{-1}$) via modulated DSC (MDSC) with a mean heating rate of 2 °C min$^{-1}$, a modulated temperature amplitude of ±1 °C and a modulation period of 120 s. Baseline and heat capacity calibration was performed with a sapphire standard before the measurement. Data evaluation was carried out in TA instruments TRIOS version 5.1.1.46572.

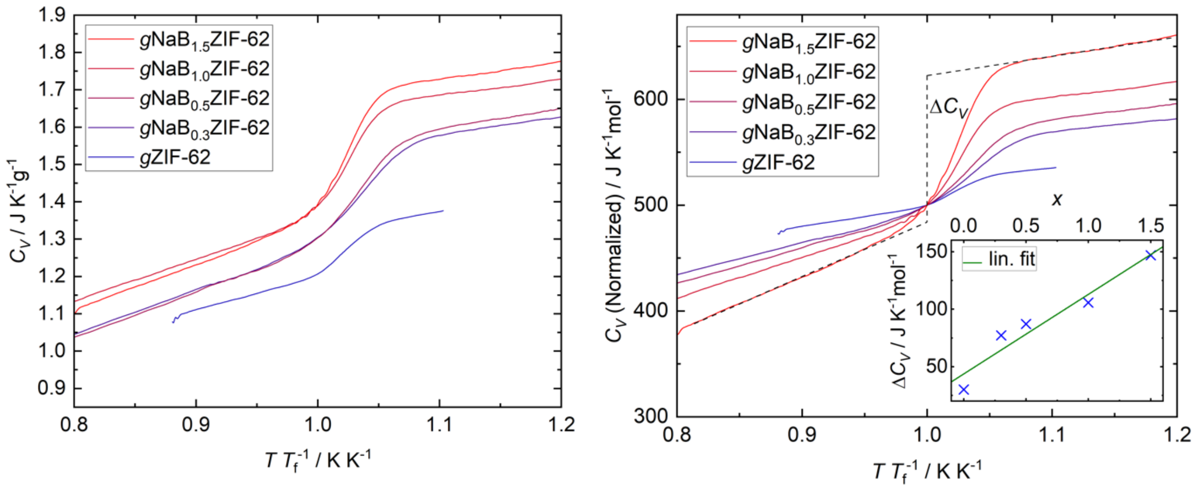


**Supplementary Figure 52.** Reversing heat capacities $C_V$ of *g*NaB$_x$ZIF-62 materials with various amounts of modifier *x* according to the general chemical formula ZnNa$_x$(im)$_{1.8}$(bim)$_{0.2+x}$ defined as molecular unit. Left: Absolut gravimetric reversing heat capacities of *g*NaB$_x$ZIF-62 materials in the unit J K$^{-1}$ g$^{-1}$. Right: Molar reversing heat capacities of *g*NaB$_x$ZIF-62 materials. The offset in molar heat capacity was chosen arbitrarily according to the intersection point ($T\ T_f^{-1} = 1$) and corrected accordingly. The inset shows the change of the heat capacity $\Delta C_V$ at the temperature of the glass transition recorded with a heating rate of 2 °C min$^{-1}$ ($T_f$) plotted against the modifier content *x*.

**Supplementary Table 7**. Reverse heat capacity $\Delta C_V$, at the temperature of the glass transition $T_f$ measured with a heating rate of 2 °C min$^{-1}$ for the modified ZIF-62 glasses with different Na(bim) contents *x*.

| $x$ | $\Delta C_V$ / J mol$^{-1}$K$^{-1}$ | $T_f$ / °C |
|---|---|---|
| 0 | 30 | 304.8 |
| 0.3 | 77 | 174.9 |
| 0.5 | 87 | 171.1 |
| 1.0 | 106 | 160.7 |
| 1.5 | 146 | 154.4 |

# S10. X-Ray Pair Distribution Function Analysis

## S10.1. Experimental Details

Variable temperature *in-situ* X-ray total scattering measurements were performed at Diamond Light Source (DLS), UK, and Deutsches Elektronen Synchrotron (DESY), Germany. At DLS three different physical mixtures with $x$ = 0.5, 1.0, and 1.5 of glass-former ZIF-62 with varying amounts of Na(bim) modifier were investigated under heating to 450 °C and after melt-quenching as well as the pure starting compounds Na(bim) and ZIF-62. For the measurements, the powdered sample material was filled in quartz capillaries (1 mm outer diameter) under an inert Ar atmosphere and sealed with epoxy glue (UHU-Schnellfest). X-rays with a wavelength of 0.1617 Å were used for data collection. Data processing was performed using PDFgetX3. For $NaB_{0.3}$|ZIF-62 *in-situ* variable temperature X-ray total scattering measurements were performed at DESY with a wavelength of 0.2071 Å starting at room temperature with a stepwise increase up to 450 °C as well as after returning to room temperature. The variable temperature measurements were collected using quartz capillaries (1 mm outer diameter). Additionally, a room temperature measurement of finely ground *g*$NaB_{0.3}$ZIF-62 material (prepared in a DSC apparatus via the small-scale approach, see Experimental Section) was conducted using a borosilicate capillary (1 mm outer diameter). Sample preparation, filling of the capillaries and sealing with UHU Schnellfest was performed under an inert atmosphere (Glovebox). Data processing was performed using DAWN Science Version 2.30.0 and PDFgetX3.

For the differential PDF analysis, only the data collected at DLS ($NaB_x$|ZIF-62 with $x$ = 0, 0.5, 1.0, 1.5) were included as these data were collected under identical measurement conditions regarding the *in-situ* glass preparation procedure (heating rates, temperature steps, data collection time) as well as the wavelength and the choice of capillaries. The total scattering data were reevaluated using Gudrun (Version 2017) which is expected to provide pair distribution functions with higher accuracy in the low $r$ region than PDFGetX3. In the latter case, the applied polynomial correction leads to artefacts in the extracted $G(r)$ functions at low $r$.[9] The densities of the physical mixtures and their glasses were calculated based on the respective ratio of the crystallographic densities of the isolated crystalline starting compounds Na(bim) and ZIF-62 (see Supplementary Table 8). The maximum $Q$-space used in the Fourier

transformation was set to $Q_{max}$ = 18.5 Å$^{-1}$. Gudrun provides PDF data denoted as differential correlation function "*D*(*r*)". These data, which were used for the evaluation presented here, are in accordance with the *G*(*r*) functions obtained from PDFgetX3 regarding their physical meaning as defined in Equation 2 with *S*(*Q*) as the scattering function with the respective limit of $S(Q \rightarrow \infty) = 1$.[10] For the sake of consistency, the *D*(*r*) functions are denoted as *G*(*r*) in the following evaluation.

$$G(r) = \frac{2}{\pi} \int_{Q_{min}}^{Q_{max}} Q\,[S(Q) - 1]\, sin(Qr)\, \mathrm{d}Q \qquad (2)$$

## S10.2. Variable Temperature *I*(*Q*) Data

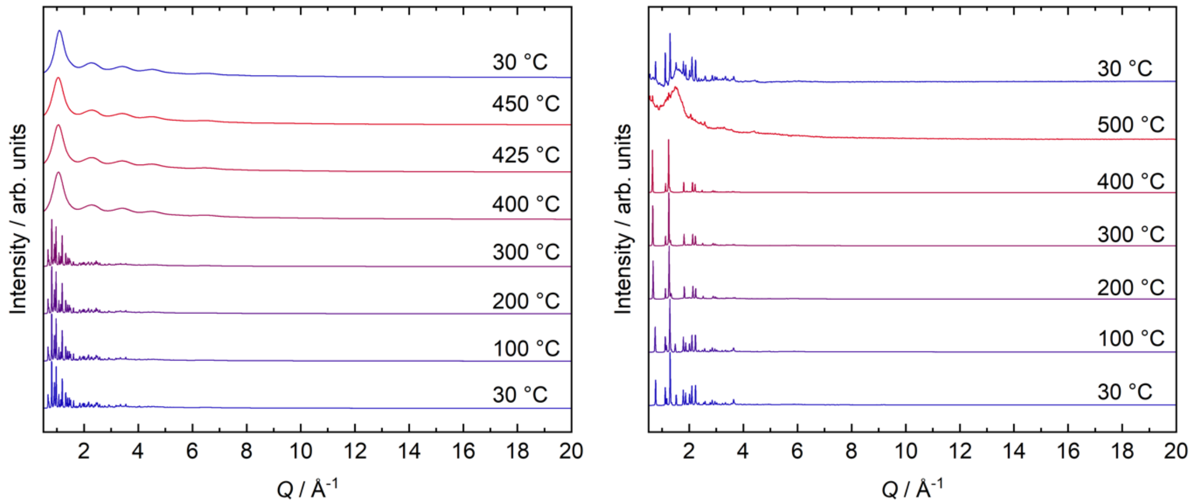


**Supplementary Figure 53.** Normalized, background-subtracted and scattering-corrected diffraction data collected for PDF analysis of the glass former ZIF-62 (left) and modifier Na(bim) (right) with increasing temperature and after temperature-quenching (top-pattern in blue). Data collection was performed at DLS with a wavelength of 0.1617 Å.

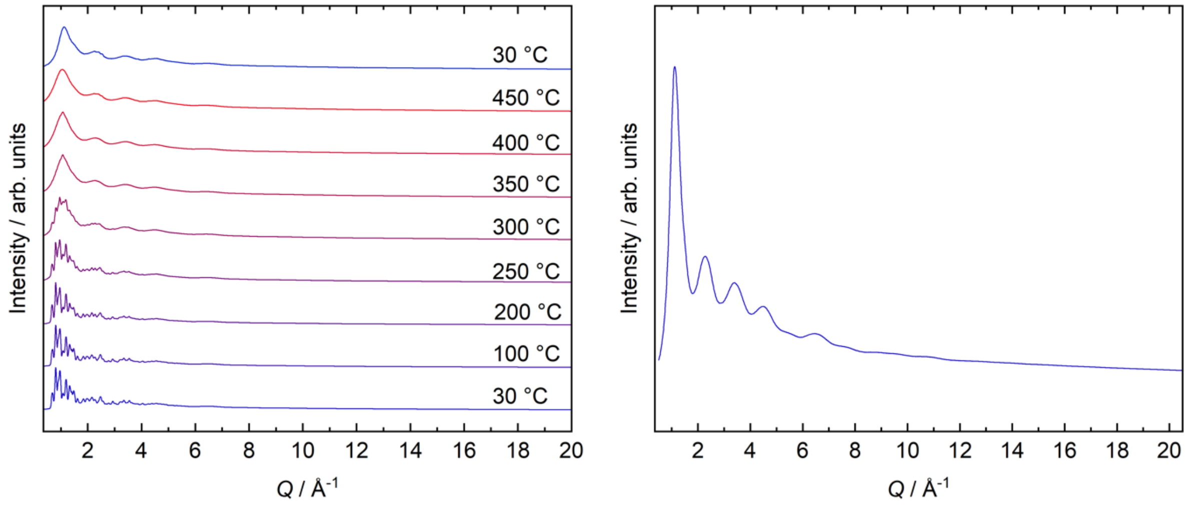


**Supplementary Figure 54.** Left: Normalized, background-subtracted, and scattering-corrected diffraction data collected for PDF analysis of the physical mixtures $NaB_{0.3}$|ZIF-62 with increasing temperature and after temperature-quenching (top-pattern in blue). Right: Normalized, background-subtracted, and scattering-corrected diffraction data collected for PDF analysis of *g*$NaB_{0.3}$ZIF-62 prepared *ex-situ*. Data collection was performed at DESY with a wavelength of 0.2077 Å.

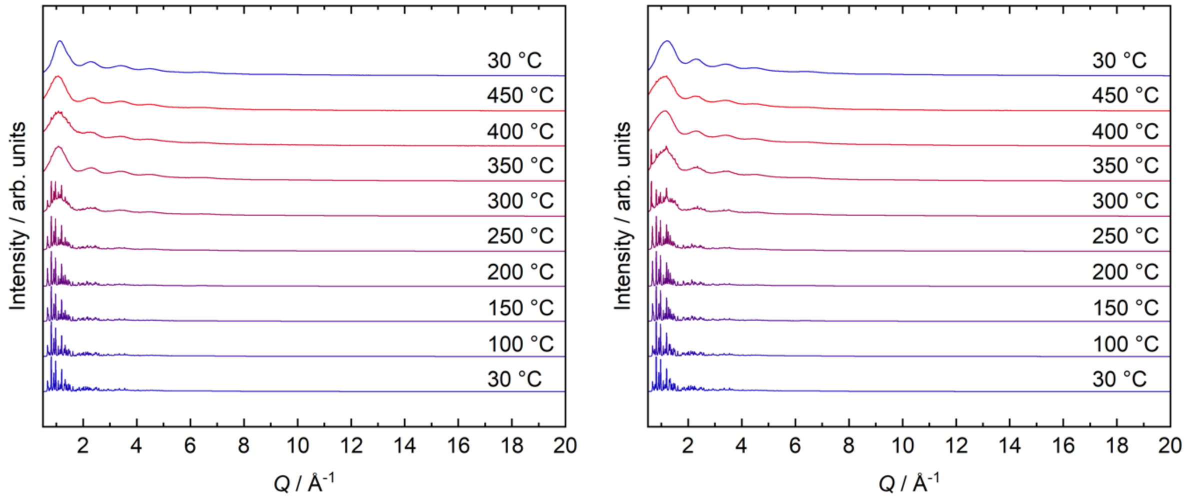


**Supplementary Figure 55.** Normalized, background-subtracted, and scattering-corrected diffraction data collected for PDF analysis of the physical mixtures $NaB_x$|ZIF-62 with $x$ = 0.5 (left), 1.0 (right) with increasing temperature and after temperature-quenching (top-pattern in blue). Data collection was performed at DLS with a wavelength of 0.1617 Å.

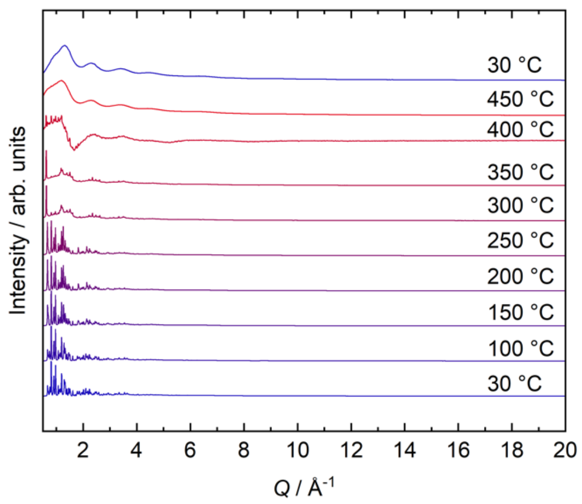


**Supplementary Figure 56**. Normalized, background-subtracted, and scattering-corrected diffraction data collected for PDF analysis of the physical mixtures NaB$_x$|ZIF-62 with $x$ = 1.5 with increasing temperature and after temperature-quenching (top-pattern in blue). Data collection was performed at DLS with a wavelength of 0.1617 Å. The overall low intensity in the pattern collected at 400 °C is the result of the liquid material moving out of the beam path.

## S10.3. Variable Temperature *S*(*Q*) Data

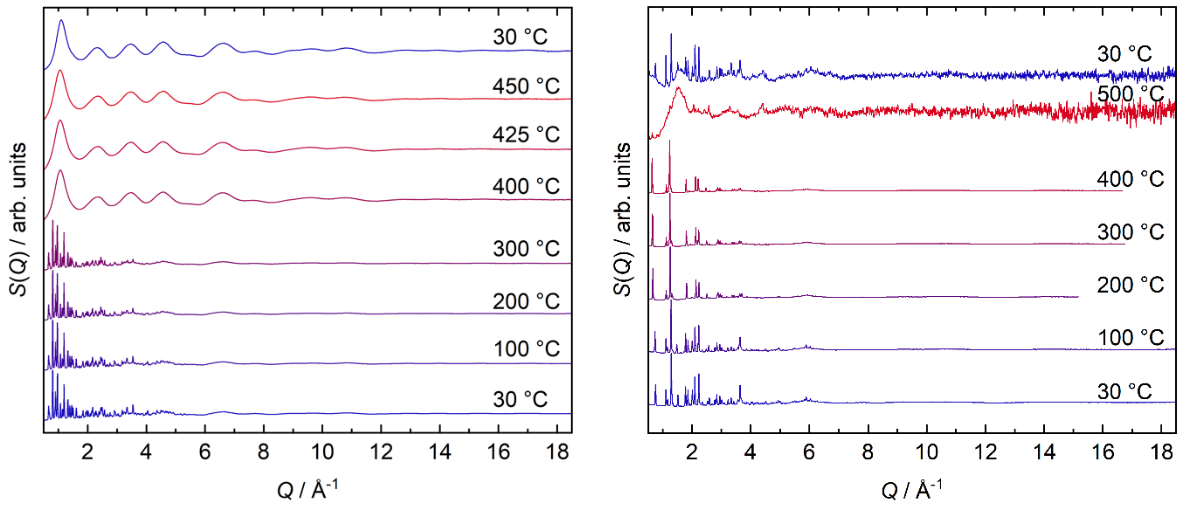


**Supplementary Figure 57**. Structure function *S*(*Q*) extracted from X-ray total scattering data for the glass former ZIF-62 (left) and Na(bim) (right) with increasing temperature and after temperature-quenching (top-pattern in blue). Data collection was performed at DLS with a wavelength of 0.1617 Å. The overall low intensity in the pattern of Na(bim) (right) collected at 500 °C is the result of the liquid material moving out of the beam path.

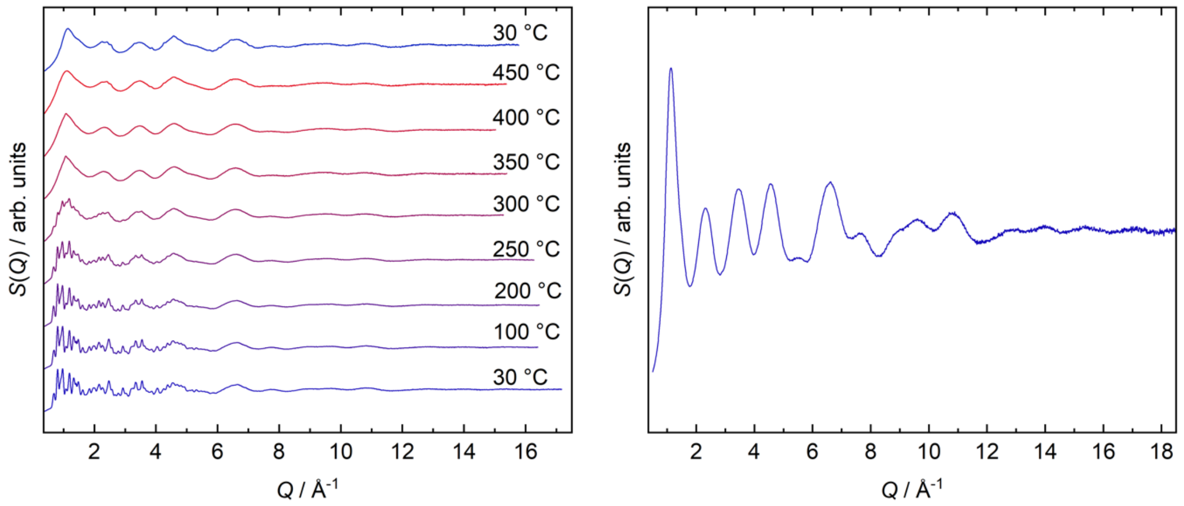


**Supplementary Figure 58**. Right: Structure function *S*(*Q*) extracted from X-ray total scattering data of $NaB_{0.3}$|ZIF-62 with increasing temperature and after temperature-quenching (top-pattern in blue). Left: Structure function *S*(*Q*) extracted from X-ray total scattering data of *g*$NaB_{0.3}$ZIF-62 prepared *ex-situ*. Data collection was performed at DESY with a wavelength of 0.2077 Å.

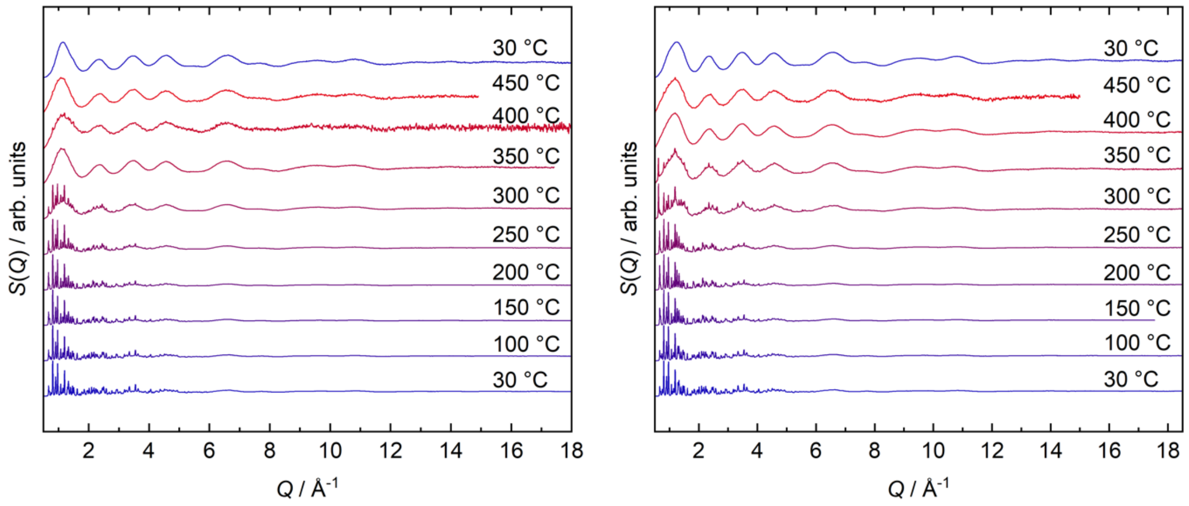


**Supplementary Figure 59.** Structure function *S*(*Q*) extracted from X-ray total scattering data for the physical mixtures $NaB_x$|ZIF-62 with $x$ = 0.5 (left), 1.0 (right) with increasing temperature and after temperature-quenching (top-pattern in blue). Data collection was performed at DLS with a wavelength of 0.1617 Å. The overall low intensity and high noise level in the pattern of $NaB_{0.5}$|ZIF-62 (left) collected at 400 °C is the result of the liquid material moving out of the beam path.

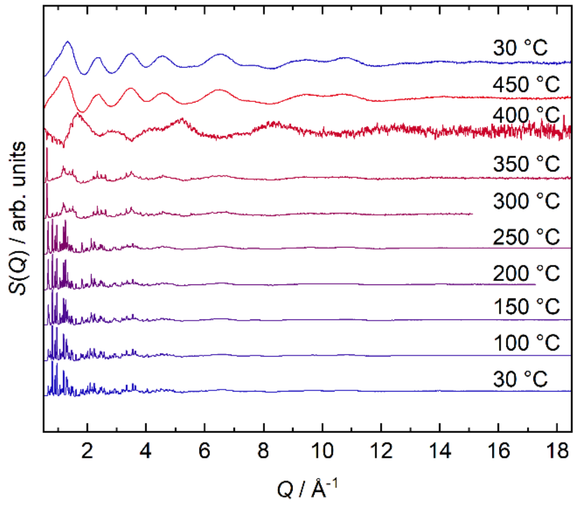


**Supplementary Figure 60**. Structure function $S(Q)$ extracted from X-ray total scattering data for the physical mixture $NaB_x$|ZIF-62 with $x$ = 1.5 with increasing temperature and after temperature-quenching (top-pattern in blue). Data collection was performed at DLS with a wavelength of 0.1617 Å. The overall low intensity and high noise level in the pattern collected at 400 °C is the result of the liquid material moving out of the beam path.

## S10.4. Variable Temperature *G*(*r*) Data

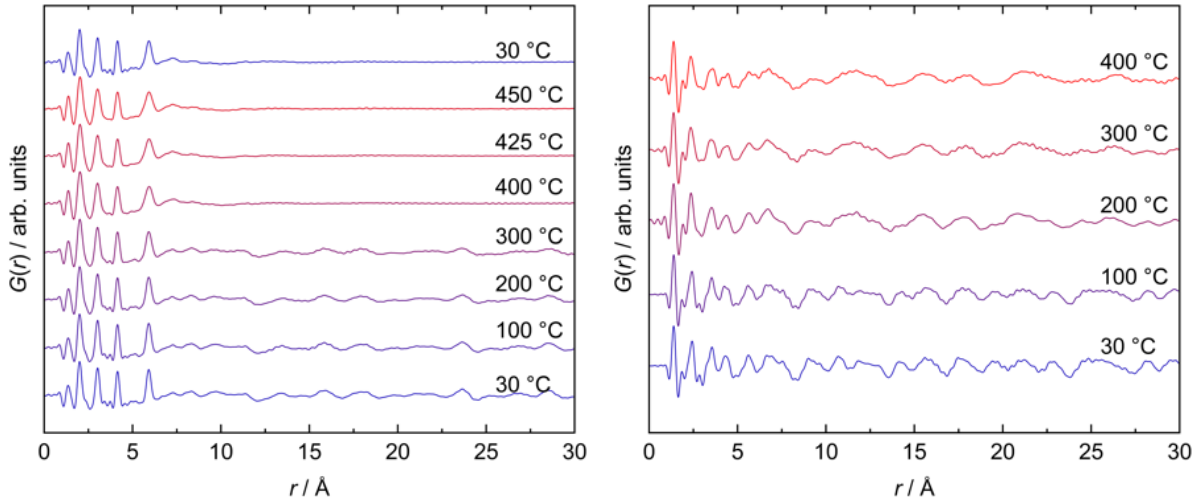


**Supplementary Figure 61.** *In-situ* PDF data in the form of $G(r)$ of the glass former ZIF-62 (left) and Na(bim) (right) with increasing temperature and after temperature-quenching (top-pattern in blue). Data collection was performed at DLS with a wavelength of 0.1617 Å.

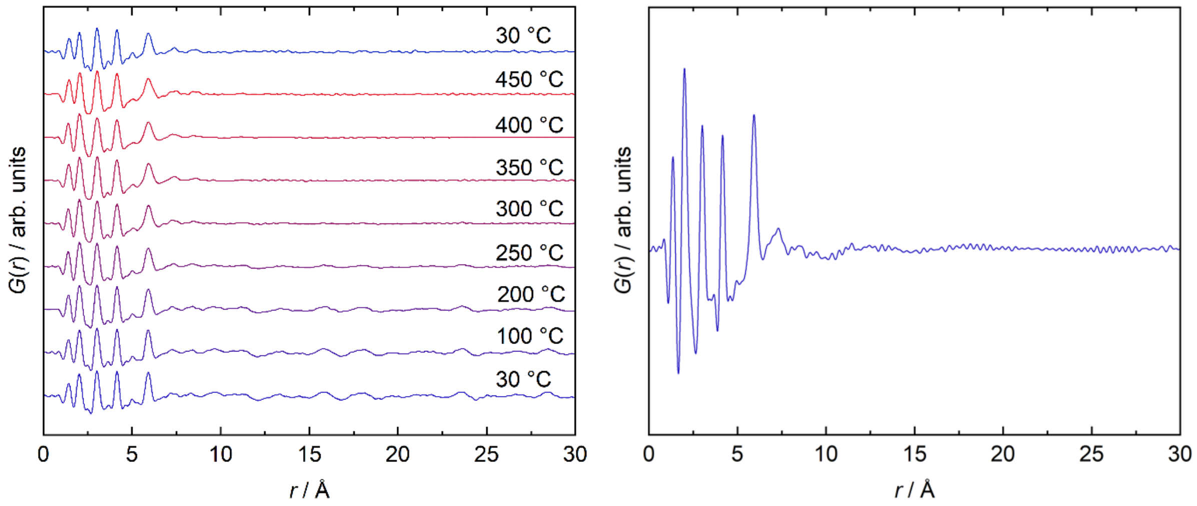


**Supplementary Figure 62.** Left: *In-situ* PDF data in the form of *G*(*r*) of the physical mixture $NaB_{0.3}$|ZIF-62 with increasing temperature and after temperature-quenching (top-pattern in blue). Right: PDF data in the form of *G*(*r*) of *g*$NaB_{0.3}$ZIF-62 prepared *ex-situ*. Data collection was performed at DESY with a wavelength of 0.2077 Å.

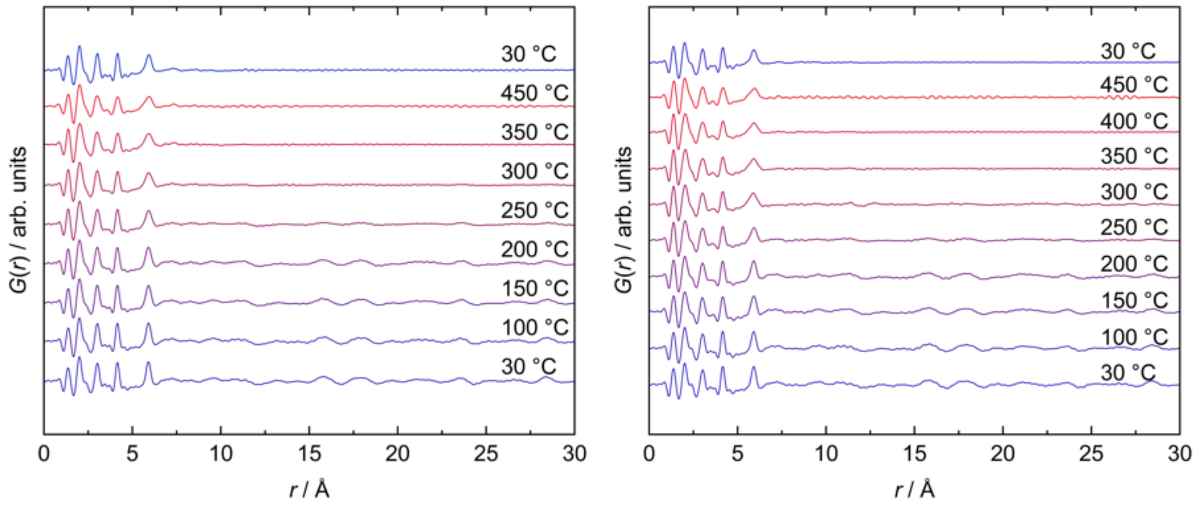


**Supplementary Figure 63.** *In-situ* PDF data in the form of *G*(*r*) of the physical mixtures $NaB_x$|ZIF-62 with $x$ = 0.5 (left), 1.0 (right) with increasing temperature and after temperature-quenching (top-pattern in blue). Data collection was performed at DLS with a wavelength of 0.1617 Å.

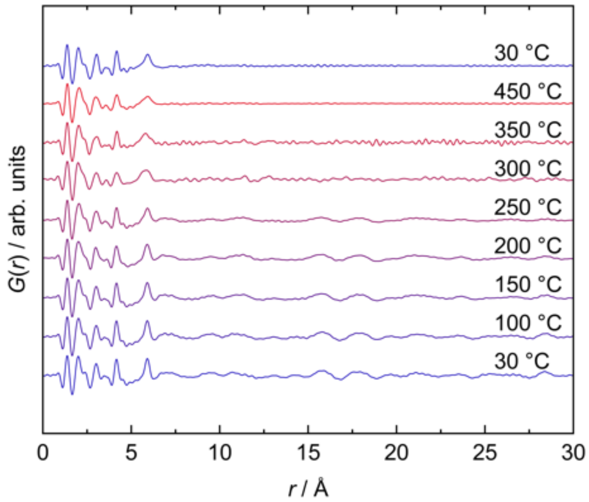


**Supplementary Figure 64.** *In-situ* PDF data in the form of $G(r)$ of the physical mixtures $NaB_x$|ZIF-62 with $x = 1.5$ with increasing temperature and after temperature-quenching (top-pattern in blue). Data collection was performed at DLS with a wavelength of 0.1617 Å.

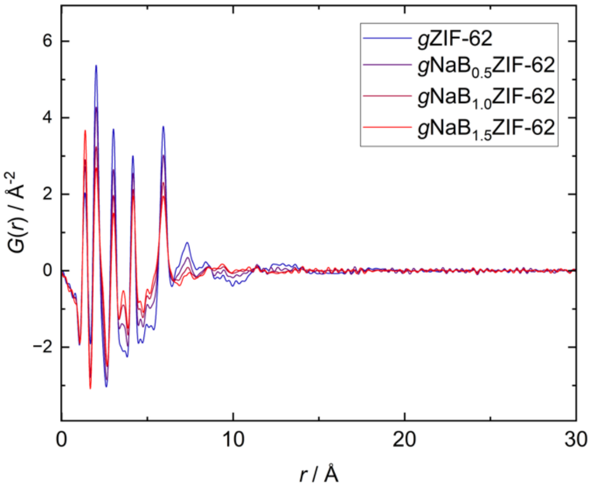


**Supplementary Figure 65.** PDF data in the form of $G(r)$ of the modified glasses $g$Na$B_x$ZIF-62 collected at room temperature proving the absence of long-range order for the modified glasses. Data collection was performed at DLS with a wavelength of 0.1617 Å.

## S10.5. First Sharp Diffraction Peak Analysis

A closer look at the first sharp diffraction peak (FSDP) provides more information about the medium range order (MRO) of the modified glass.[11–14] An accepted theory by Elliott describes the FSDP's origin as a consequence of the periodicity of boundaries of small voids which are homogenously distributed throughout the material.[15] As shown in Supplementary Figure 67 the intensity of the total scattering structure functions $S(Q)$ decreases with increasing amount of modifier incorporated. Within the void model, this intensity decrease can be explained by a loss of contrast in electron density as former voids are successively occupied by the modifier. A similar behaviour was observed for sodium-ion-modified silicate glasses investigated by neutron scattering.[15,16] The shift of the FSDP's maximum from 1.10 $Å^{-1}$ to 1.33 $A^{-1}$ with an increase in modifier content is associated with the densification of the material, while the broadening of the FSDP corresponds to a loss of real-space correlation length ($L = 2\pi/\Delta Q$). Hence, the incorporation of the modifier seems to reduce the MRO of the glass while increasing the density and reducing the porosity of the glassy material.

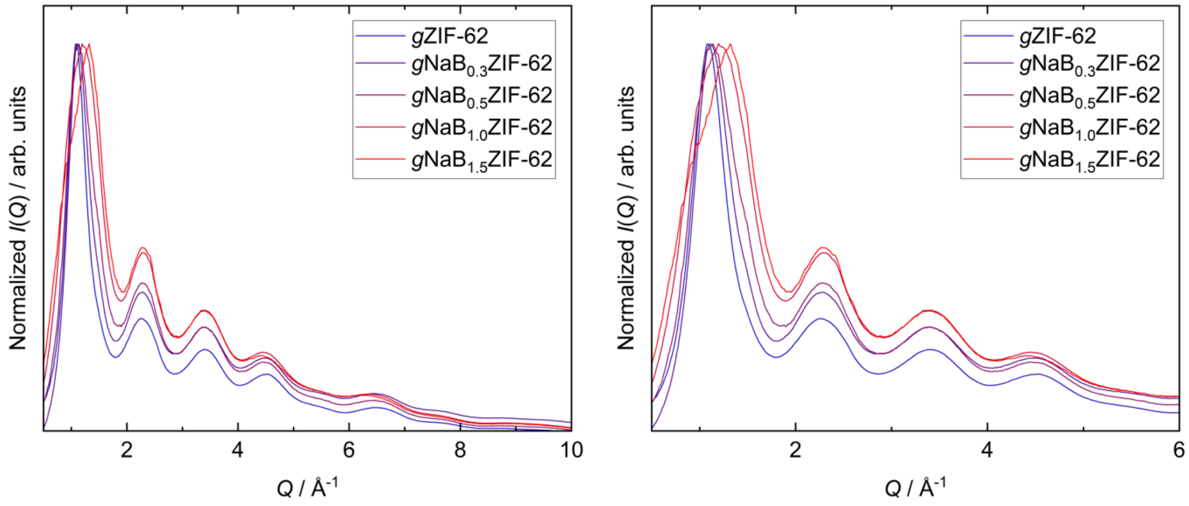


**Supplementary Figure 66.** Normalized, background-subtracted and scattering-corrected diffraction data collected for PDF analysis of *g*$NaB_xZIF$-62 materials for $x$ = 0 (*g*ZIF-62), 0.3, 0.5, 1.0 and 1.5 (left) and zoom into the low-$Q$ region displaying the systematic shift in the FSDP with increasing modifier content $x$ (right).

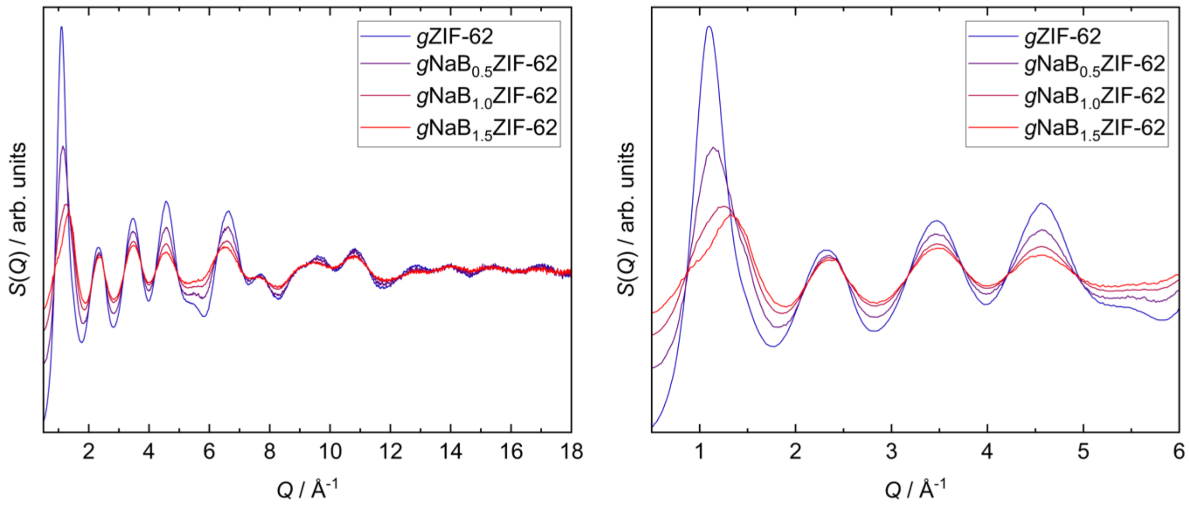


**Supplementary Figure 67.** Total scattering structure functions *S*(*Q*) of *g*NaB$_x$ZIF-62 materials for $x$ = 0 (*g*ZIF-62), 0.5, 1.0, 1.5 with a zoom into the low-*Q* region displaying the shift in the FSDP and the loss of its intensity with increasing modifier contents $x$.

## S10.6. Differential PDF Analysis

**Supplementary Table 8.** Mean crystallographic densities $\rho_m$ and number densities $\rho_0$ of NaB$_x$|ZIF-62 materials calculated from the densities of the crystalline compounds ZIF-62 and Na(bim). The calculated densities were used in the processing of the PDF data of NaB$_x$|ZIF-62 and *g*NaB$_x$ZIF-62 materials.

| Material | $\rho_m$ / g cm$^{-3}$ | $\rho_0$ / Å$^{-3}$ |
|---|---|---|
| ZIF-62 | 1.297 | 0.068 |
| NaB$_{0.5}$\|ZIF-62 | 1.322 | 0.073 |
| NaB$_{1.0}$\|ZIF-62 | 1.338 | 0.076 |
| NaB$_{1.5}$\|ZIF-62 | 1.350 | 0.079 |
| Na(bim) | 1.408 | 0.091 |

Firstly, the functions *G*(*r*) were transformed to the radial distribution functions (RDFs) *R*(*r*) via:

$$R(r) = r(G(r) + 4\pi r \rho_0) \tag{3}$$

The RDFs of the physical mixtures were fitted with a linear combination of the RDFs of the pure compounds Na(bim) and ZIF-62, assuming that the contribution of the RDF of the particle's interface in the physical mixtures can be neglected. This approach is similar to differential PDF analysis and closely related to principal component analysis (PCA) as described in the literature.[17,18]

$$\frac{1}{r} R_{\mathrm{LC}} = \frac{1}{r} \left(c_1 \cdot R_{\text{ZIF-62}}(r) + c_2 \cdot R_{\mathrm{Na(bim)}}(r)\right) \tag{4}$$

With $c_1$ and $c_2$ being the coefficients representing the intensity contribution of the RDFs of the pure compounds to the RDF of their physical mixtures.

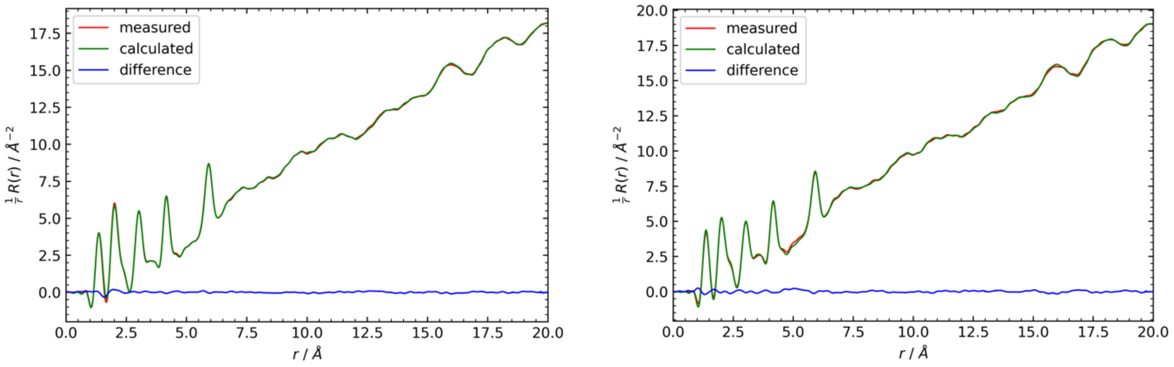


**Supplementary Figure 68.** RDFs of the physical mixtures NaB$_x$|ZIF-62 (measured) with $x$ = 0.5 (left) and 1.0 (right), fitted with the linear combination function $R_{LC}(r)/r$ (calculated) and the difference between both with $R^2$ = 0.999 (left) and $R^2$ = 0.999 (right). The refined linear combination parameters are shown in Supplementary Table 9.

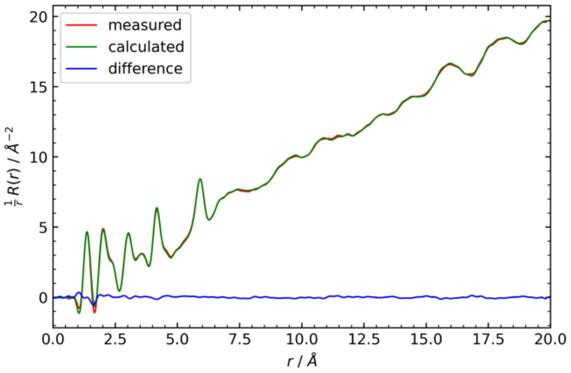


**Supplementary Figure 69.** RDFs of the physical mixtures NaB$_x$|ZIF-62 (measured) with $x$ = 1.5 fitted with the linear combination function $R_{LC}(r)/r$ (calculated) and the difference between both with $R^2$ = 0.999. The refined linear combination parameters are shown in Supplementary Table 9.

The difference curves for all linear combination fits (Supplementary Figure 68 and Supplementary Figure 69) appear featureless and show excellent agreement between the $R(r)$ of the physical mixtures and $R_{LC}(r)$. Therefore, it can be concluded that the contribution of the particle interfaces to the RDFs and PDFs can be neglected. The obtained linear combination coefficients $c_1$ and $c_2$ for the construction of the $R_{LC}(r)$ functions obtained as the result of these fits are summarized in Supplementary Table 9.

**Supplementary Table 9.** Linear combination coefficients $c_1$ and $c_2$ of the linear combination function $R_{LC}(r)$ for different physical mixtures with varying ratios of glass former (ZIF-62) and -modifier (Na(bim)) together with the coefficient of determination ($R^2$) provided by the fits shown in Supplementary Figure 68 and Supplementary Figure 69.

| Material | $c_1$ (ZIF-62) | $c_2$ (Na(bim)) | $R^2$ |
|---|---|---|---|
| $NaB_{0.5}$\|ZIF-62 | 0.798 ± 0.001 | 0.208 ± 0.001 | 0.999 |
| $NaB_{1.0}$\|ZIF-62 | 0.708 ± 0.001 | 0.312 ± 0.001 | 0.999 |
| $NaB_{1.5}$\|ZIF-62 | 0.634 ± 0.001 | 0.395 ± 0.001 | 0.999 |

A closer look at the $G(r)$ functions of the isolated starting compounds reveals that the signal associated with next-neighbour Zn···Zn correlations in ZIF-62 ($r$ = 5.9 Å) are located at the same distance where the PDF of Na(bim) has a value close to zero (see Supplementary Figure 70, left). Consequently, the PDF of Na(bim) does not contribute to the intensity of the physical mixture's $G(r)$ at $r$ = 5.9 Å. In the $G(r)$ functions of the physical mixtures, the same signal decreases in intensity upon the addition of Na(bim) (see Supplementary Figure 70, right). This is explained by the decreasing density of next-neighbour Zn···Zn correlations as a result of the successive dilution of ZIF-62 upon the addition of Na(bim).

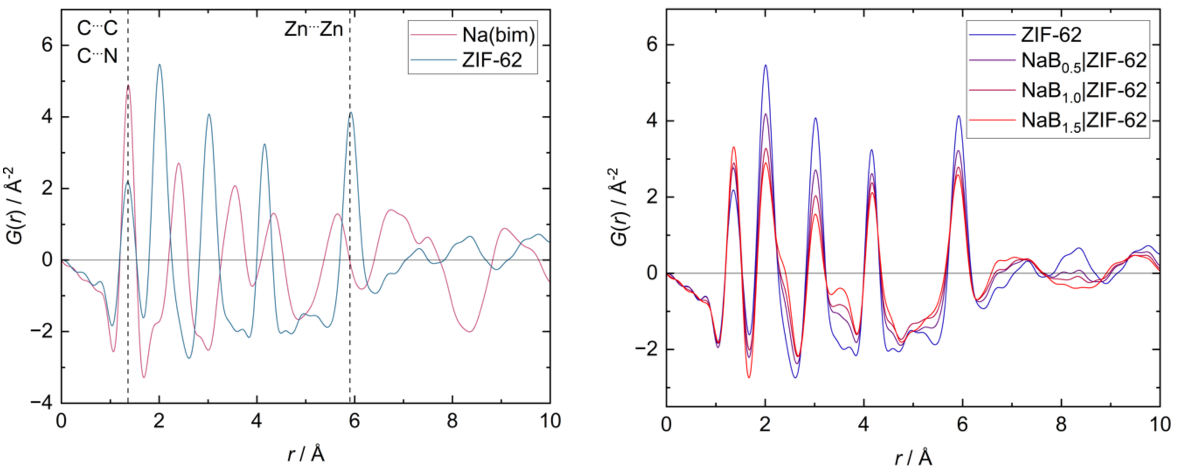


**Supplementary Figure 70.** PDFs in the form of $G(r)$ for Na(bim) and ZIF-62 (left) as well as for different physical mixtures (right). The dashed lines mark the allocation of next-neighbour C···C/C···N and Zn···Zn correlations.

Next, the signal at 5.9 Å was chosen for rescaling the $G$(r) functions of the physical mixtures to the intensity found for pure ZIF-62. This step is performed to achieve a renormalization of the physical mixture's $G(r)$ functions to the next-neighbour Zn···Zn

correlation density of ZIF-62. This normalization is essentially a normalization to a molar unit of $ZnNa_x(im)_{1.8}(bim)_{0.2+x}$. Hence, the scaling factors derived from the respective maxima of the physical mixture's *G*(*r*) functions (at 5.9 Å) are in good accordance with the scaling factors calculated from $c_1$ ($c_1^{-1}$) in the LC-fitting approach discussed above (see Supplementary Table 9 and Supplementary Table 10). In the following, all rescaled *G*(*r*) functions are denoted as *G**(*r*). In the *G**(*r*) functions of the physical mixtures, the intensity of the signal at 5.9 Å remains the same throughout all physical mixtures, whereas the intensity of the signal at 1.37 Å increases with *x* (see Figure 4 in the main manuscript*)*. This signal, corresponding to next-neighbour C···C and C···N correlations, increases with *x* due to the growing number of those correlations per molar unit ($ZnNa_x(im)_{1.8}(bim)_{0.2+x}$).

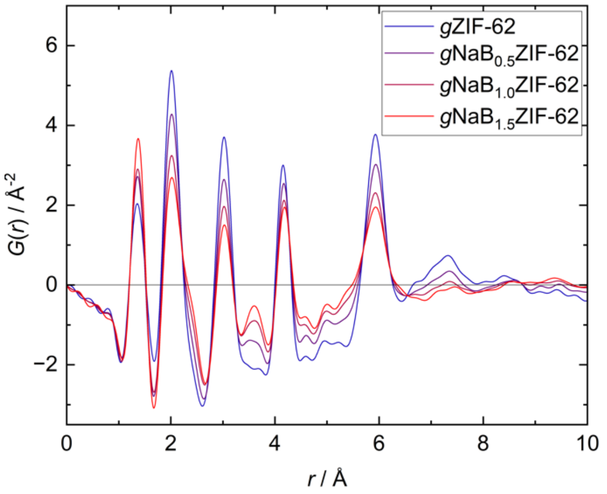


**Supplementary Figure 71.** *G*(*r*) functions of the modified glasses *g*NaB*x*|ZIF-62 showing the increase of C···C/C···N correlation density (signal at 1.4 Å) and decreasing Zn···Zn correlation density (signal at 5.9 Å) with growing amount of Na(bim) incorporation.

Upon glass formation, the organic (benz)imidazolate linkers remain intact as proven by solution $^1$H NMR spectroscopic measurements (see Section S7). Hence, the signal reflecting the number of C···C/C···N correlations should remain largely unaltered during vitrification. Rescaling the *G*(*r*) functions of the glasses according to the maximum intensity of this signal found in the physical mixture's *G**(*r*) function (first signal at ~ 1.37 Å) provides *G**(*r*) functions for the corresponding glasses (see Figure 4 in the main manuscript).

**Supplementary Table 10.** Scaling factors $c$ for different physical mixtures and their corresponding glasses. The scaling factors were obtained from the linear combination fitting approach ($c_{LC} = c_1^{-1}$) and by comparing the maximum intensity of the signal at 5.9 Å (Zn···Zn) in the $G(r)$ function of the physical mixtures with the intensity of ZIF-62 ($c_{Zn...Zn}$). $c_{C...C}$ refers to the scaling factor of a glass' to the respective physical mixture's $G(r)$ function and is based on the relative maximum intensities of the first signal at 1.4 Å (C···C/C···N). The far-right column contains the product of the two scaling factors applied to the glasses' $G(r)$ functions to obtain $G^*(r)$.

| $x$ | $c_{LC}$ | $c_{Zn...Zn}$ | $c_{C...C}$ | $c_{Zn...Zn} \cdot c_{C...C}$ |
|---|---|---|---|---|
| 0.0 | 1.000 | 1.000 | 1.073 | 1.073 |
| 0.5 | 1.253 | 1.268 | 1.017 | 1.290 |
| 1.0 | 1.412 | 1.471 | 1.004 | 1.478 |
| 1.5 | 1.577 | 1.573 | 0.905 | 1.424 |

To further evaluate the change in the abundance of correlations in the $G^*(r)$ functions during glass formation, the difference between the $G^*(r)$ functions of the physical mixtures and the vitreous counterpart was calculated for the varying chemical compositions. These $\Delta G^*(r)$ functions ($\Delta G^*(r) = G^*_{glass}(r) - G^*_{physical\ mixture}(r)$) monitor the change in the atomic pair distribution occurring upon the transformation of the crystalline physical mixture to the sodium-modified ZIF glass (see Figure 4 in the main manuscript).

## S10.7. Discussion of $\Delta G^*(r)$ approach

The $\Delta G^*(r)$ functions show a loss in signal intensity for the correlation at 5.9 Å assigned to an increasing loss of next-neighbour Zn···Zn pair correlations with increasing modifier content (see Figure 4 in the main manuscript). The $\Delta G^*(r)$ functions further display an increased population of distances around 4.5 – 5.5 Å and a further decrease in population in the region around 6.5 – 7.5 Å with the growing amount of modifier incorporated in the vitrification process.

Nonetheless, interpreting these changes is challenging due to the rather high noise level in the $\Delta G^*(r)$. Further, it must be noted that for the calculation of the $\Delta G^*(r)$ functions and the necessary scaling of the $G(r)$ functions, the approximation of a suitable baseline is crucial. The baseline $f(r)$ of the $G(r)$ function depends on the average number density $\rho_0$ of the materials (in the chosen normalisation) and can be described via the linear Equation 5:[19]

$$f(r) = -4\,\pi\,\rho_0\,r \quad (5)$$

For the PDF evaluation, the number density $\rho_0$ of the physical mixtures was calculated as the mean number density of ZIF-62 and Na(bim) weighted by their respective ratio (see Supplementary Table 8). The subsequent upscaling of the physical mixture's $G(r)$ functions to $G^*(r)$ (with scaling factors > 0, see Supplementary Table 10) formally increases the number density $\rho_0^*$ (with $f^*(r) = -4\,\pi\,\rho_0^*\,r$, for the baseline of $G^*(r)$) to a value where the physical mixture's number density reflects the same Zn···Zn pair correlation density as for pure ZIF-62. As a consequence, the signal reflecting Zn···Zn pair correlations (at 5.9 Å) remain of the same maximum intensity in the physical mixture's $G^*(r)$ functions.

For the evaluation of the glasses' PDFs, the same number densities $\rho_0$ were applied as for the physical mixtures. The scaling factors for the calculation of $G^*(r)$ for the modified glasses are very similar to the scaling factors used in the physical mixtures $G^*(r)$ (see Supplementary Table 10). The trend of an increasing density of the $g$NaB$_x$ZIF-62 materials with $x$, which is reflected in the calculated $\rho_0$ (and $\rho_0^*$) values, is also monitored by the growing shift of the FSDP in $S(Q)$ with $x$ (see Section 10.5). However, it is known that the density of $g$ZIF-62 is slightly higher than for the crystalline parent framework.[13]

This densification is not taken into account in the evaluation presented here due to the lack of exact density values for the $g$NaB$_x$ZIF-62 materials. Nonetheless, the densification during glass formation will lead to a slightly steeper falling baseline than anticipated, which will influence the signal intensities used for scaling and, therefore, the resulting $\Delta G^*(r)$ function. However, due to the anticipated higher densities of $g$NaB$_x$ZIF-62 materials compared to the corresponding physical mixtures, the observed trends in the $\Delta G^*(r)$ functions with varying $x$ are expected to remain unchanged or become even more pronounced even though the real densities of the glasses cannot be applied, as they are unknown.

## S11. Extended X-ray Absorption Fine Structure Analysis

### S11.1. Experimental Details

XAFS measurements were conducted at BL10 at Dortmund Electron Accelerator (DELTA) from 9.5 keV to 10.5 keV in transmission mode to cover the energy of the Zn K absorption edge. The material was mixed and thoroughly ground with various amounts of hexagonal boron nitride (BN) to reach a dilution of ca. 50 – 80 % (w/w). The prepared material was then filled in Kapton capillaries (1 mm inner diameter), and the capillaries were sealed using epoxy glue under an inert (Ar) atmosphere in a glovebox. The calibration of the energy at the Zn K edge was performed using Zn powder diluted to 10% using BN.

XAFS data analysis was performed in the ATHENA module of the Demeter software package.[20] The EXAFS spectra were obtained by subtracting the post-edge background from the overall absorption, followed by normalisation of the data with respect to the edge-jump step. Subsequently, the $\chi(k)$ data were Fourier transformed into real ($R$) space to separate the EXAFS contributions from different coordination shells. To obtain the quantitative structural parameters surrounding Zn atoms, least-squares curve fitting was performed using the ARTEMIS module of the Demeter software package.

The following EXAFS equation was used:

$$\chi(k) = \sum_j \frac{N_j S_0^2 F_j(k)}{k R_j^2} \cdot \exp\left[-2k^2\sigma_j^2\right] \cdot \exp\left[\frac{-2R_j}{\lambda(k)}\right] \cdot \sin\left[2kR_j + \phi_j(k)\right] \qquad (6)$$

where $S_0^2$ is the amplitude reduction factor, $F_j(k)$ is the effective curved-wave backscattering amplitude, $N_j$ is the number of neighbors in the $j$-th atomic shell, $R_j$ is the distance between the X-ray absorbing central atom and the atoms in the $j$-th atomic shell (back scatterer), $\lambda$ is the mean free path in Å, $\phi_j(k)$ is the phase shift (including the phase shift for each shell and the total central atom phase shift), $\sigma_j$ is the Debye-Waller parameter of the $j$-th atomic shell (variation of distances around the average $R_j$). The amplitude reduction factor $S_0^2$ was determined to be 0.91 by fitting the Zn–N coordination in crystalline ZIF-62, where the coordination number is known to be 4. This fixed value of $S_0^2$ was subsequently applied to all other samples to extract the Zn–N coordination number and real Zn–N bond length (see Supplementary Table 11).

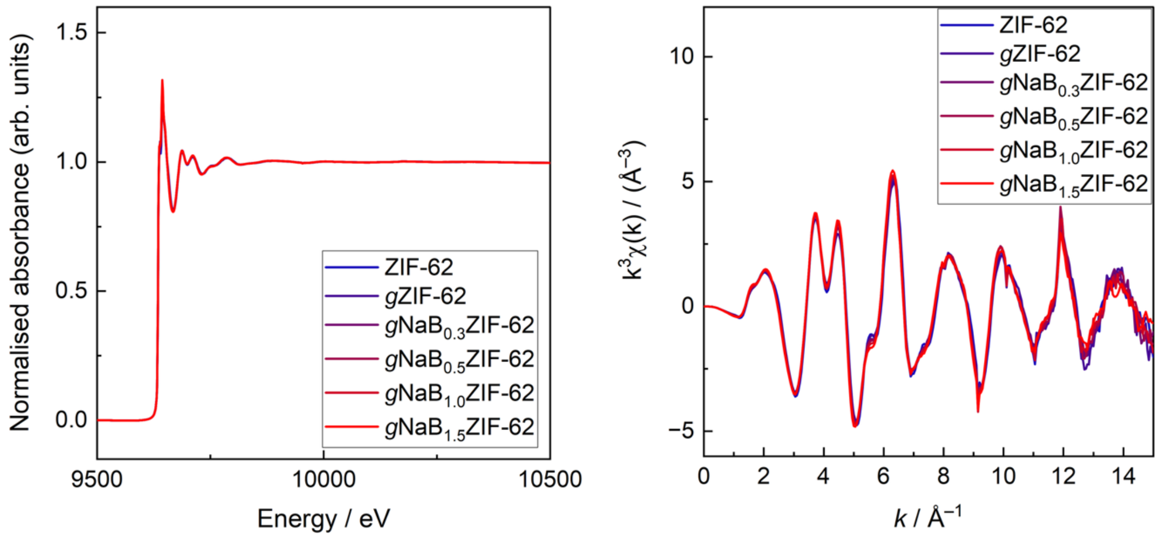


**Supplementary Figure 72.** Left: Normalized X-ray absorption spectra of ZIF-62, *g*ZIF-62 and the modified glasses *g*NaB$_x$ZIF-62. Right: EXAFS signal in the form of $k^3\chi(k)$ plotted against the wavenumber *k*.

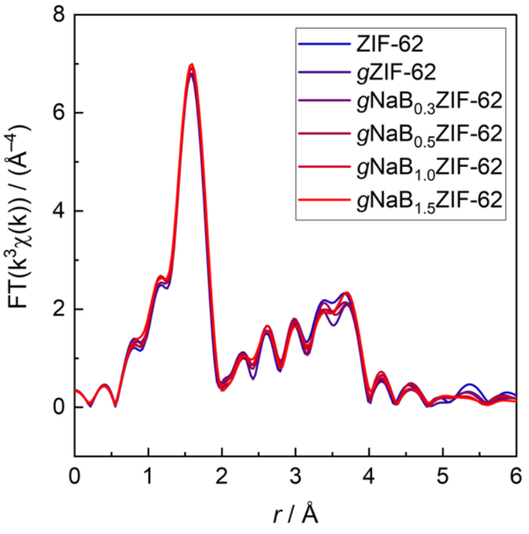


**Supplementary Figure 73.** Left: Fourier-transform of the EXAFS signal plotted against the real-space distance *r* for ZIF-62, *g*ZIF-62 and the modified glasses *g*NaB$_x$ZIF-62.

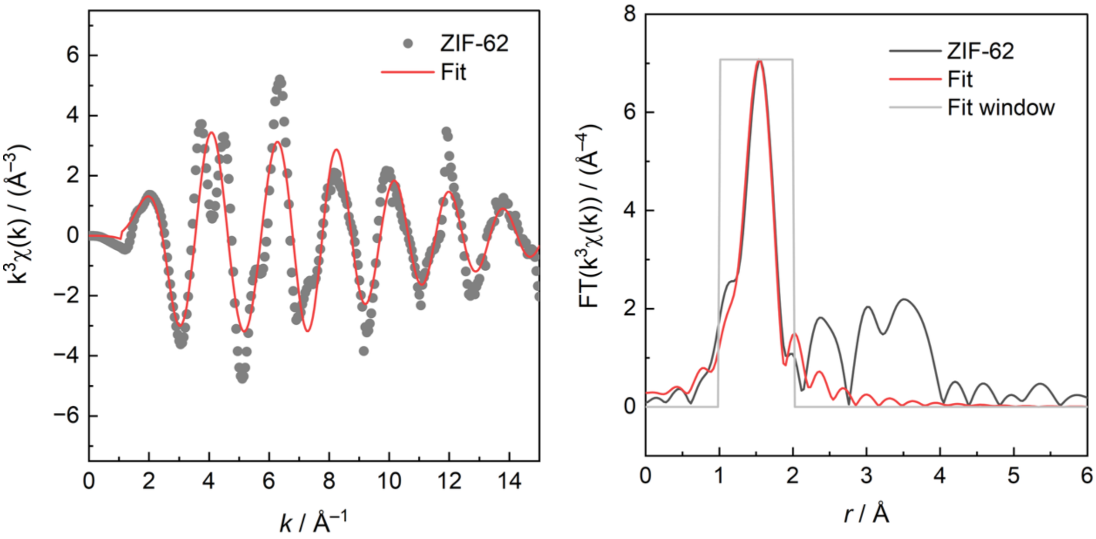


**Supplementary Figure 74.** Left: Fit to the EXAFS signal in the form of $k^3\chi(k)$ according to Equation 6 for ZIF-62. Right: Fourier transform of the EXAFS signal for ZIF-62 plotted against the real-space distance *r* with the corresponding Fourier transform of the fit shown left. The fitted range covers the interatomic distances of the first coordination shell of $Zn^{2+}$ (i.e. [$ZnN_4$] units) in the investigated material. The refined parameters of the fitted function are summarized in Supplementary Table 11.

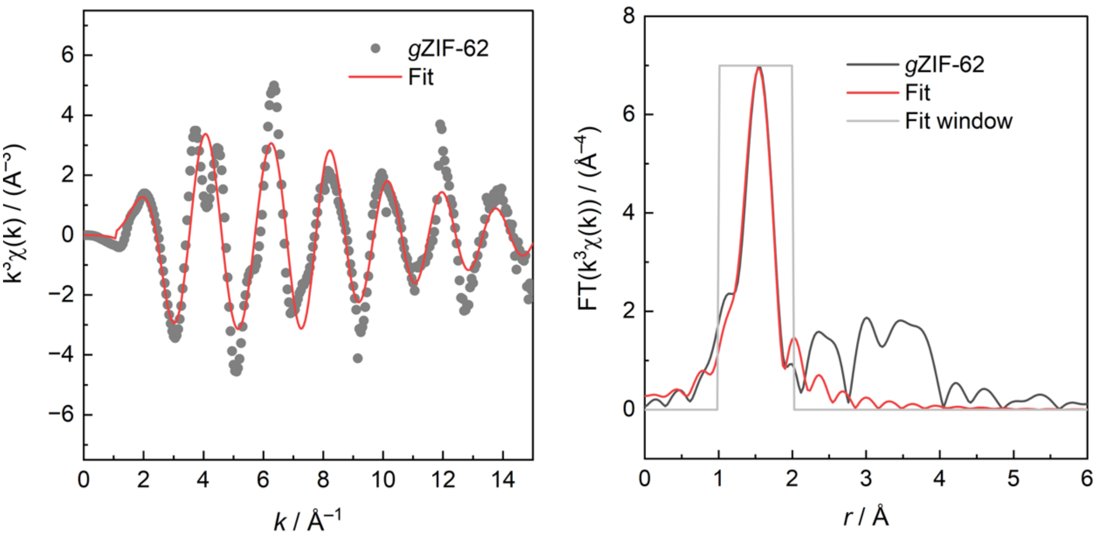


**Supplementary Figure 75.** Left: Fit to the EXAFS signal in the form of $k^3\chi(k)$ according to Equation 6 for *g*ZIF-62. Right: Fourier transform of the EXAFS signal for *g*ZIF-62 plotted against the real-space distance *r* with the corresponding Fourier transform of the fit shown left. The fitted range covers the interatomic distances of the first coordination shell of $Zn^{2+}$ (i.e. [$ZnN_4$] units) in the investigated material. The refined parameters of the fitted function are summarized in Supplementary Table 11.

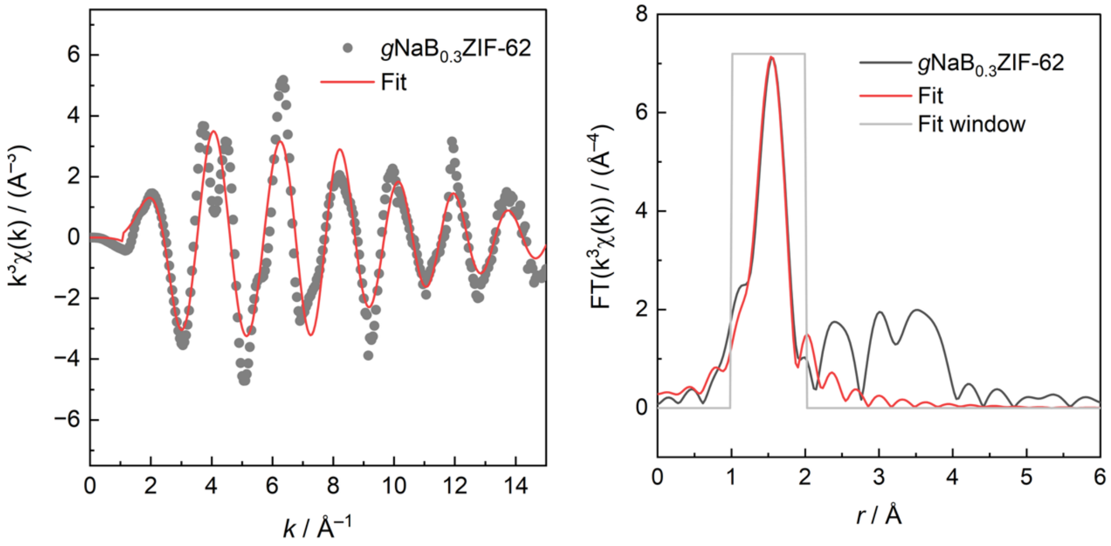


**Supplementary Figure 76.** Left: Fit to the EXAFS signal in the form of $k^3\chi(k)$ according to Equation 6 for *g*NaB$_{0.3}$ZIF-62. Right: Fourier transform of the EXAFS signal for *g*NaB$_{0.3}$ZIF-62 plotted against the real-space distance *r* with the corresponding Fourier transform of the fit shown left. The fitted range covers the interatomic distances of the first coordination shell of $Zn^{2+}$ (i.e. [$ZnN_4$] units) in the investigated material. The refined parameters of the fitted function are summarized in Supplementary Table 11.

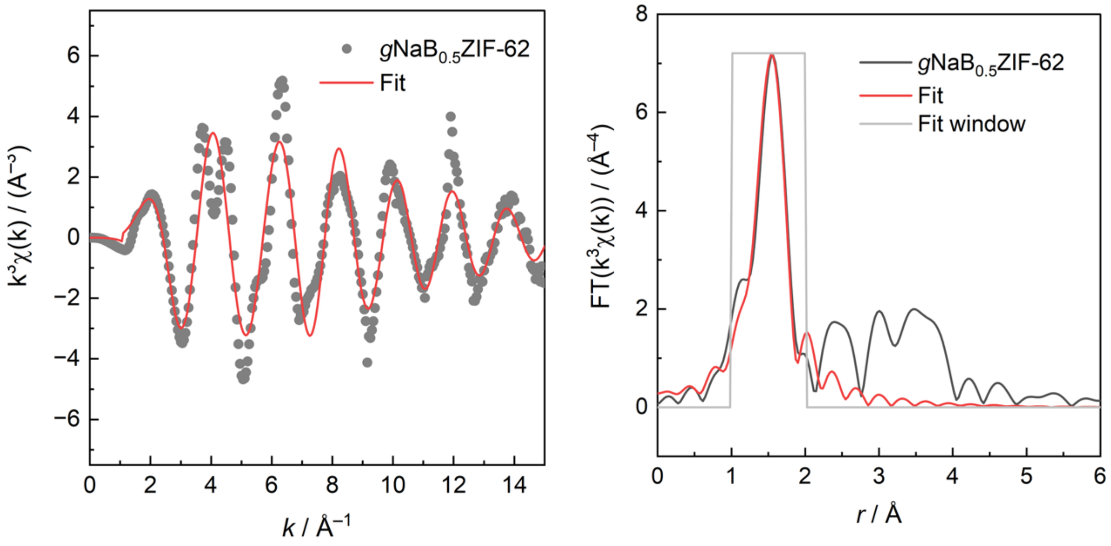


**Supplementary Figure 77.** Left: Fit to the EXAFS signal in the form of $k^3\chi(k)$ according to Equation 6 for *g*NaB$_{0.5}$ZIF-62. Right: Fourier transform of the EXAFS signal for *g*NaB$_{0.5}$ZIF-62 plotted against the real-space distance *r* with the corresponding Fourier transform of the fit shown left. The fitted range covers the interatomic distances of the first coordination shell of $Zn^{2+}$ (i.e. [$ZnN_4$] units) in the investigated material. The refined parameters of the fitted function are summarized in Supplementary Table 11.

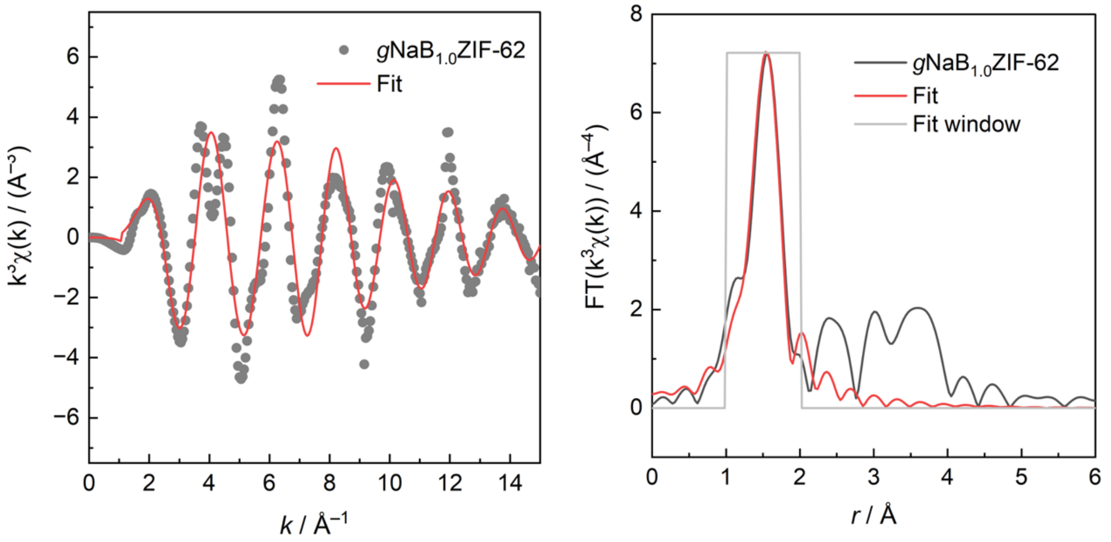


**Supplementary Figure 78.** Left: Fit to the EXAFS signal in the form of $k^3\chi(k)$ according to Equation 6 for *g*NaB$_{1.0}$ZIF-62. Right: Fourier transform of the EXAFS signal for *g*NaB$_{1.0}$ZIF-62 plotted against the real-space distance *r* with the corresponding Fourier transform of the fit shown left. The fitted range covers the interatomic distances of the first coordination shell of $Zn^{2+}$ (i.e. [$ZnN_4$] units) in the investigated material. The refined parameters of the fitted function are summarized in Supplementary Table 11.

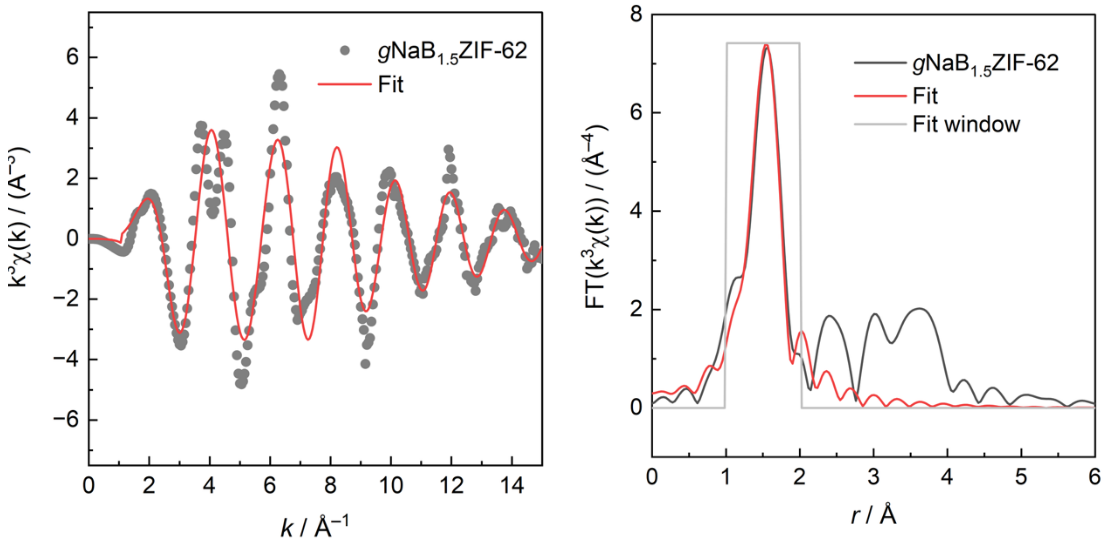


**Supplementary Figure 79.** Left: Fit to the EXAFS signal in the form of $k^3\chi(k)$ according to Equation 6 for *g*NaB$_{1.5}$ZIF-62. Right: Fourier transform of the EXAFS signal for *g*NaB$_{1.5}$ZIF-62 plotted against the real-space distance *r* with the corresponding Fourier transform of the fit shown left. The fitted range covers the interatomic distances of the first coordination shell of $Zn^{2+}$ (i.e. [$ZnN_4$] units) in the investigated material. The refined parameters of the fitted function are summarized in Supplementary Table 11.

**Supplementary Table 11.** Refined parameters obtained by fitting Equation 6 to the EXAFS signal of ZIF-62, *g*ZIF-62 and the modified glassed *g*NaB$_x$ZIF-62 shown in Supplementary Figure 74 - Supplementary Figure 79. The determined parameters correspond to the first coordination shell (i.e. [$ZnN_4$] coordination shell) and include the amplitude reduction factor $S_0$, the Zn coordination number (CN), the average Zn–N distance *r*, the determined Debye-Waller factor $\sigma^2$, the deviation of the Zn K absorption edge from the expected value for $Zn^{2+}$ $\Delta E_0$ and the *R* factor indicating the goodness if the fit.

| Material | Shell | $S_0^2$ | CN | *r* /Å | σ$^2$ | $\Delta E_0$ / eV | *R* factor |
|---|---|---|---|---|---|---|---|
| ZIF-62 | Zn-N | 0.907 ± 0.06 | 4 | 1.984 ± 0.001 | 0.004 | 4.5 ± 0.6 | 0.010 |
| *g*ZIF-62 | Zn-N | 0.91 | 3.95 ± 0.23 | 1.987 ± 0.004 | 0.004 | 4.4 ± 0.6 | 0.007 |
| *g*NaB$_{0.3}$ZIF-62 | Zn-N | 0.91 | 4.11 ± 0.28 | 1.989 ± 0.005 | 0.005 | 4.4 ± 0.6 | 0.009 |
| *g*NaB$_{0.5}$ZIF-62 | Zn-N | 0.91 | 4.03 ± 0.29 | 1.988 ± 0.005 | 0.004 | 4.3 ± 0.7 | 0.011 |
| *g*NaB$_{1.0}$ZIF-62 | Zn-N | 0.91 | 4.08 ± 0.31 | 1.989 ± 0.005 | 0.004 | 4.3 ± 0.7 | 0.013 |
| *g*NaB$_{1.5}$ZIF-62 | Zn-N | 0.91 | 4.23 ± 0.34 | 1.989 ± 0.005 | 0.004 | 4.2 ± 0.8 | 0.014 |

# S12. Atom Probe Tomography

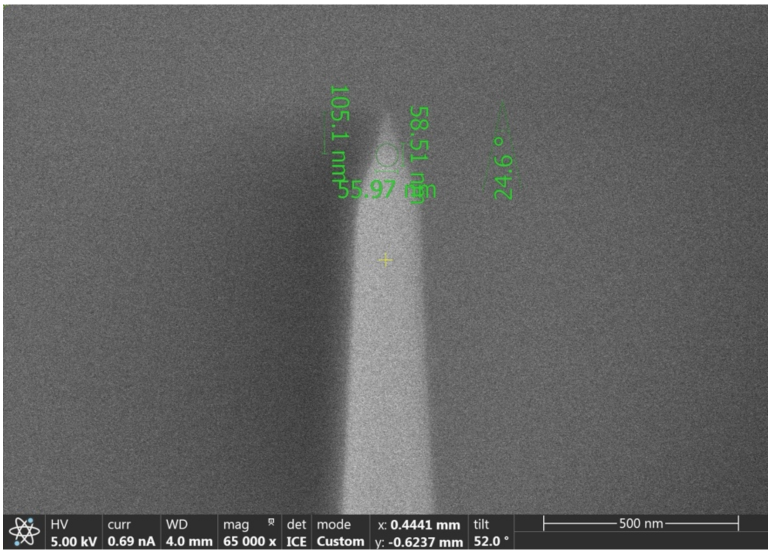


**Supplementary Figure 80.** Electron microscopy image of the specimen (*g*NaB$_{0.3}$ZIF-62) prepared for the APT measurement.

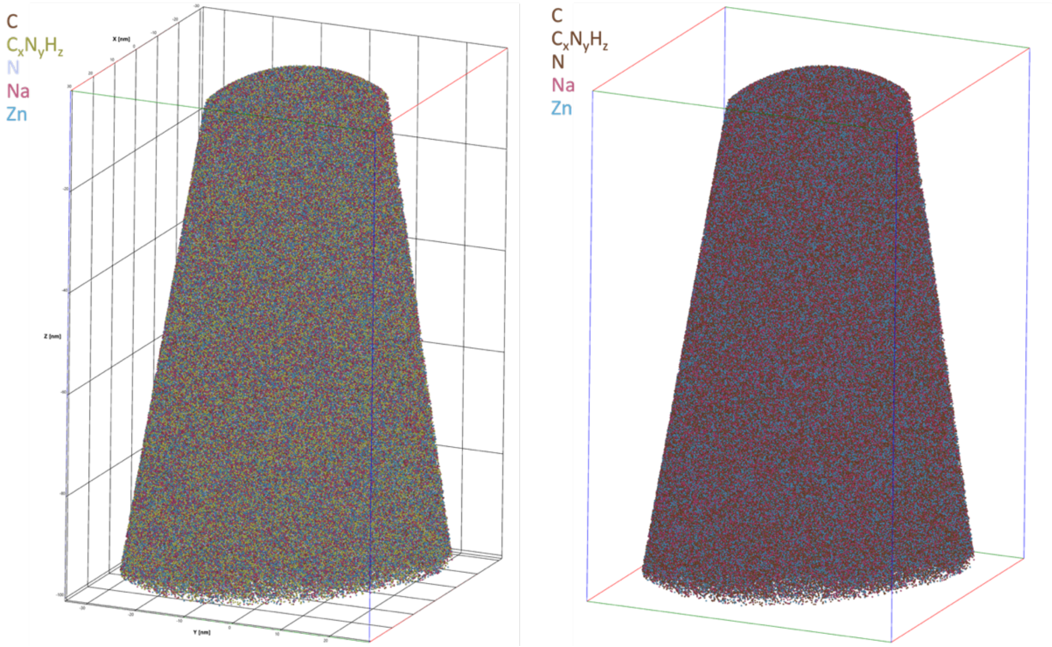


**Supplementary Figure 81.** Reconstructed atomic model of the specimen based on the ions detected in the APT measurement. Left: Full reconstruction depicting all Na- and Zn-containing (complex) ions represented as Na (red) and Zn (blue). All hydrogens and CNH-complex ions, which do not include Na and Zn, are represented by $C_xH_yN_z$ (green). Carbon and Nitrogen are represented by C (brown) and N (light blue), respectively. Right: Simplified view on the reconstruction showing all organic (complex) ions in brown. All Na and Zn-containing (complex) ions are represented as Na (red) and Zn (blue) respectively.

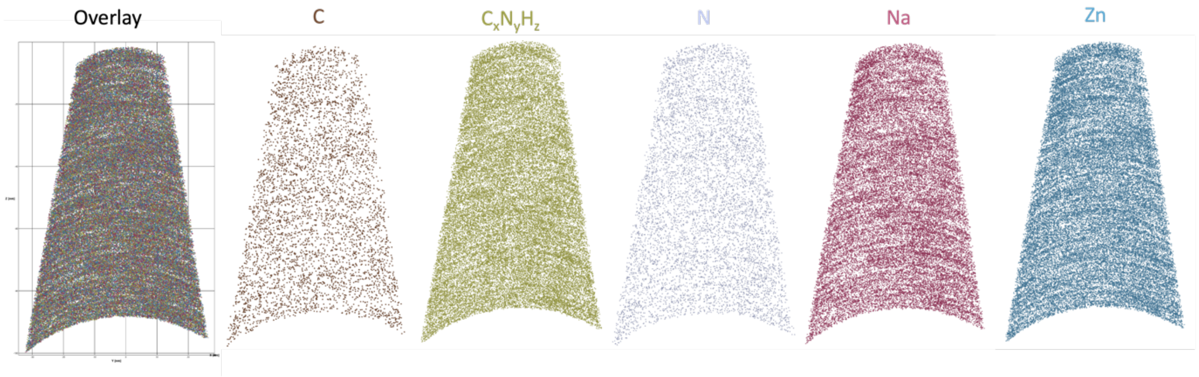


**Supplementary Figure 82.** Slice of the reconstructed atomic model as shown in Supplementary Figure 81 applying the same atomic representation scheme as in the full reconstruction (Supplementary Figure 81, left).

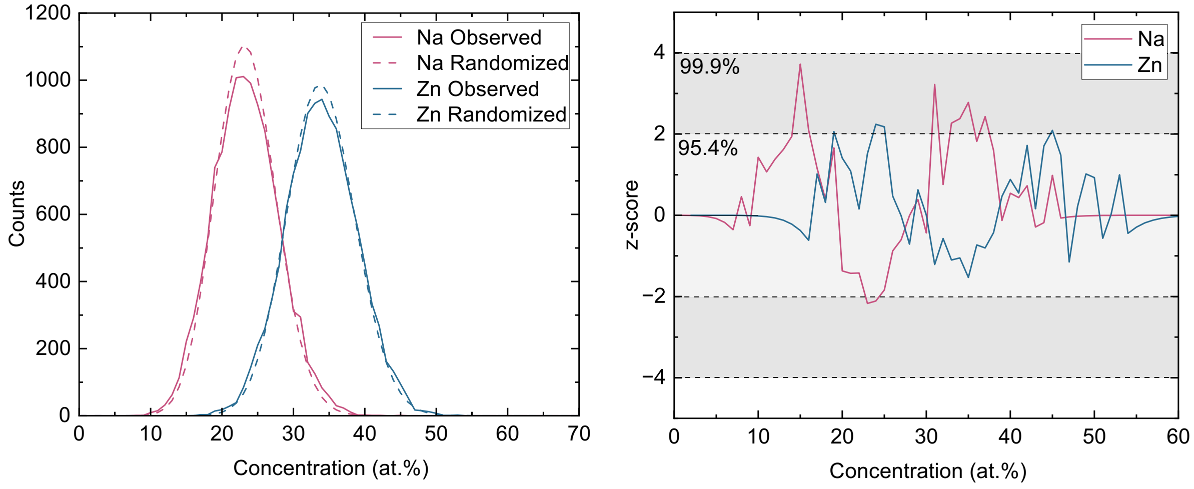


**Supplementary Figure 83.** Left: Frequency distribution curves of the respective Na and Zn sites in the reconstructed APT model together with computed randomized data as a reference for the distribution of an ideal random distribution. Right: z-Score for the frequency analysis shown left illustrating the deviation of the found ion concentration from the (random) binomial distribution with z-scores exceeding the 99.7 % confidence interval hinting towards the presence of Na ion clustering.

# S13. Electron Microscopy and Energy-Dispersive X-Ray Spectroscopy

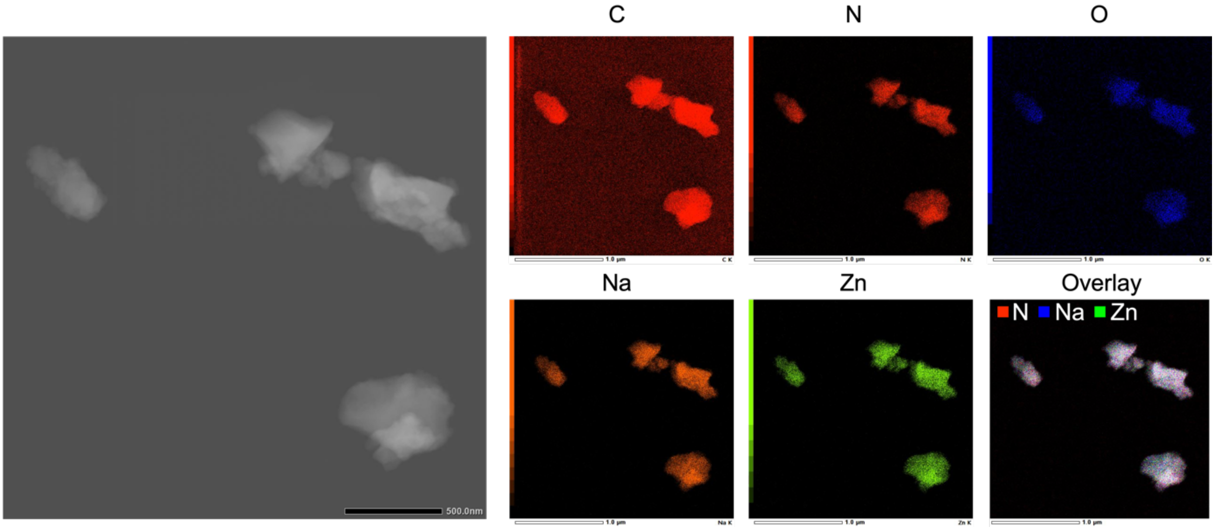


**Supplementary Figure 84.** STEM image of small shards of *g*NaB$_{0.3}$ZIF-62 (left) together with colour maps indicating the relative abundance of the respective nuclei obtained by EDX measurements.

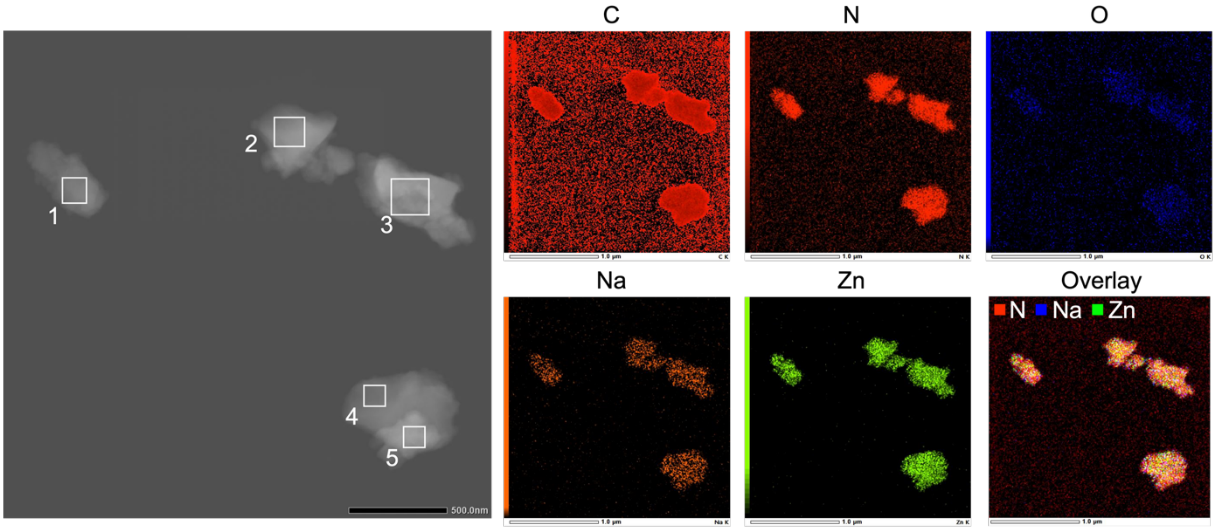


**Supplementary Figure 85.** STEM image of small shards of *g*NaB$_{0.3}$ZIF-62 (left) together with colour maps indicating the relative abundance of the respective nuclei obtained by quantitative EDX measurements. The results of the quantification approach is shown in Supplementary Table 12.

**Supplementary Table 12.** Results of the quantitative analysis of STEM-EDX measurements for *g*$NaB_{0.3}$ZIF-62. The areas correspond to the areas highlighted in Supplementary Figure 85. The expected Zn/Na ratio from synthesis and $^{1}$H NMR data is 3.3. The large deviation from the expected ratio in the STEM-EDX analysis can be explained by the complete overlap of the Zn Lα/Lβ and Na Kα emission lines, which severely compromises accuracy and reliability. The expected Zn/Na ratio from the synthesis procedure agrees with $^{1}$H NMR, IR, PXRD, PDF and DSC data.

| Area | C [at.%] | N [at.%] | O [at.%] | Na [at.%] | Zn [at.%] | S [at.%] | Cl [at.%] | Zn/Na ratio |
|---|---|---|---|---|---|---|---|---|
| 1 | 90.67 | 6.78 | 0.70 | 0.75 | 1.10 | - | - | 1.47 |
| 2 | 88.71 | 8.10 | 0.81 | 0.91 | 1.47 | - | - | 1.62 |
| 3 | 89.27 | 7.65 | 0.76 | 0.93 | 1.40 | - | - | 1.51 |
| 4 | 92.06 | 5.60 | 0.73 | 0.66 | 0.96 | - | - | 1.46 |
| 5 | 92.66 | 4.93 | 0.56 | 0.71 | 1.13 | - | - | 1.59 |

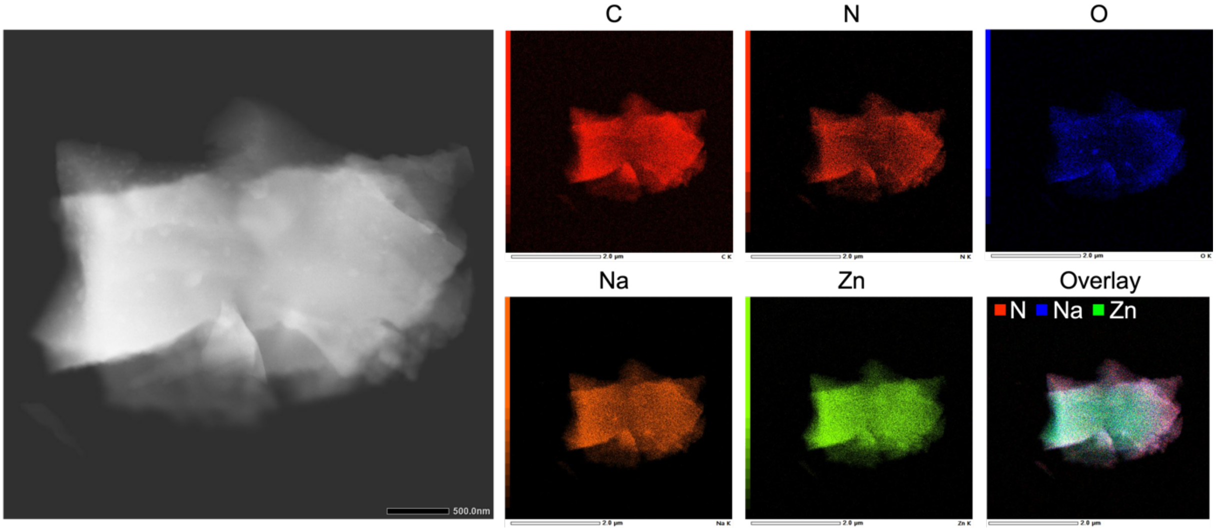


**Supplementary Figure 86.** STEM image of a shard of *g*$NaB_{1.0}$ZIF-62 (left) together with colour maps indicating the relative abundance of the respective nuclei obtained by EDX measurements.

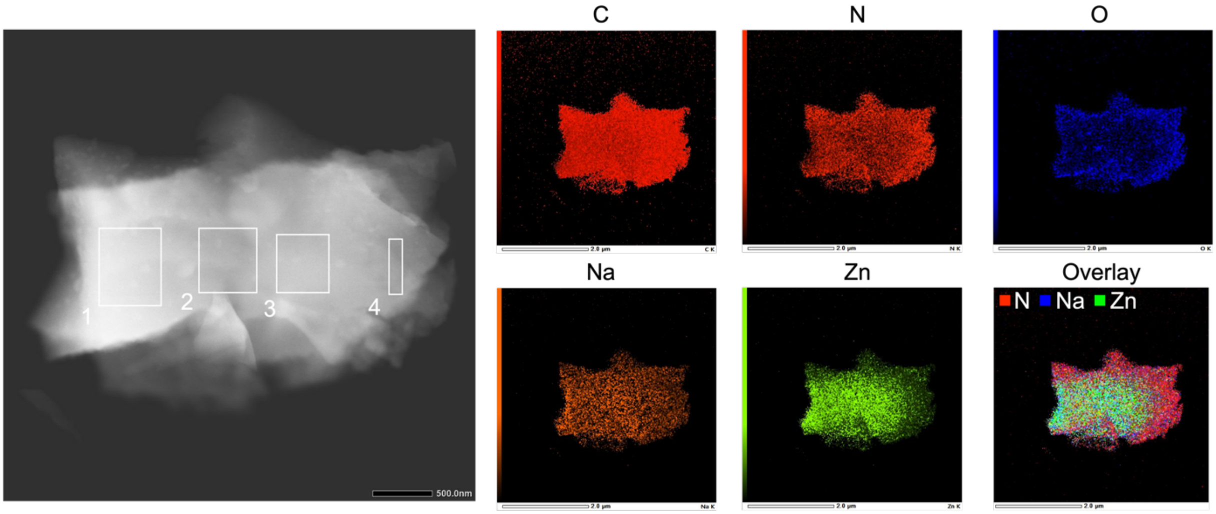


**Supplementary Figure 87.** STEM image of a shard of *g*NaB$_{1.0}$ZIF-62 (left) together with colour maps indicating the relative abundance of the respective nuclei obtained by quantitative EDX measurements. The results of the quantification approach is shown in Supplementary Table 13.

**Supplementary Table 13.** Results of the quantitative analysis of STEM-EDX measurements for *g*NaB$_{1.0}$ZIF-62. The areas correspond to the areas highlighted in Supplementary Figure 87. The expected Zn/Na ratio from synthesis and $^{1}$H NMR data is 1.0. The large deviation from the expected ratio in the STEM-EDX analysis can be explained by the complete overlap of the Zn Lα/Lβ and Na Kα emission lines, which severely compromises accuracy and reliability. The expected Zn/Na ratio from the synthesis procedure agrees with $^{1}$H NMR, IR, PXRD, PDF and DSC data.

| Area | C [at.%] | N [at.%] | O [at.%] | Na [at.%] | Zn [at.%] | S [at.%] | Cl [at.%] | Zn/Na ratio |
|---|---|---|---|---|---|---|---|---|
| 1 | 65.85 | 8.62 | 2.25 | 7.70 | 15.59 | - | - | 2.02 |
| 2 | 68.26 | 7.64 | 2.11 | 7.64 | 14.34 | - | - | 1.88 |
| 3 | 66.09 | 6.84 | 2.12 | 8.37 | 16.57 | - | - | 1.98 |
| 4 | 77.40 | 11.54 | 3.14 | 3.51 | 4.41 | - | - | 1.26 |

## S14. Density Functional Theory Calculations

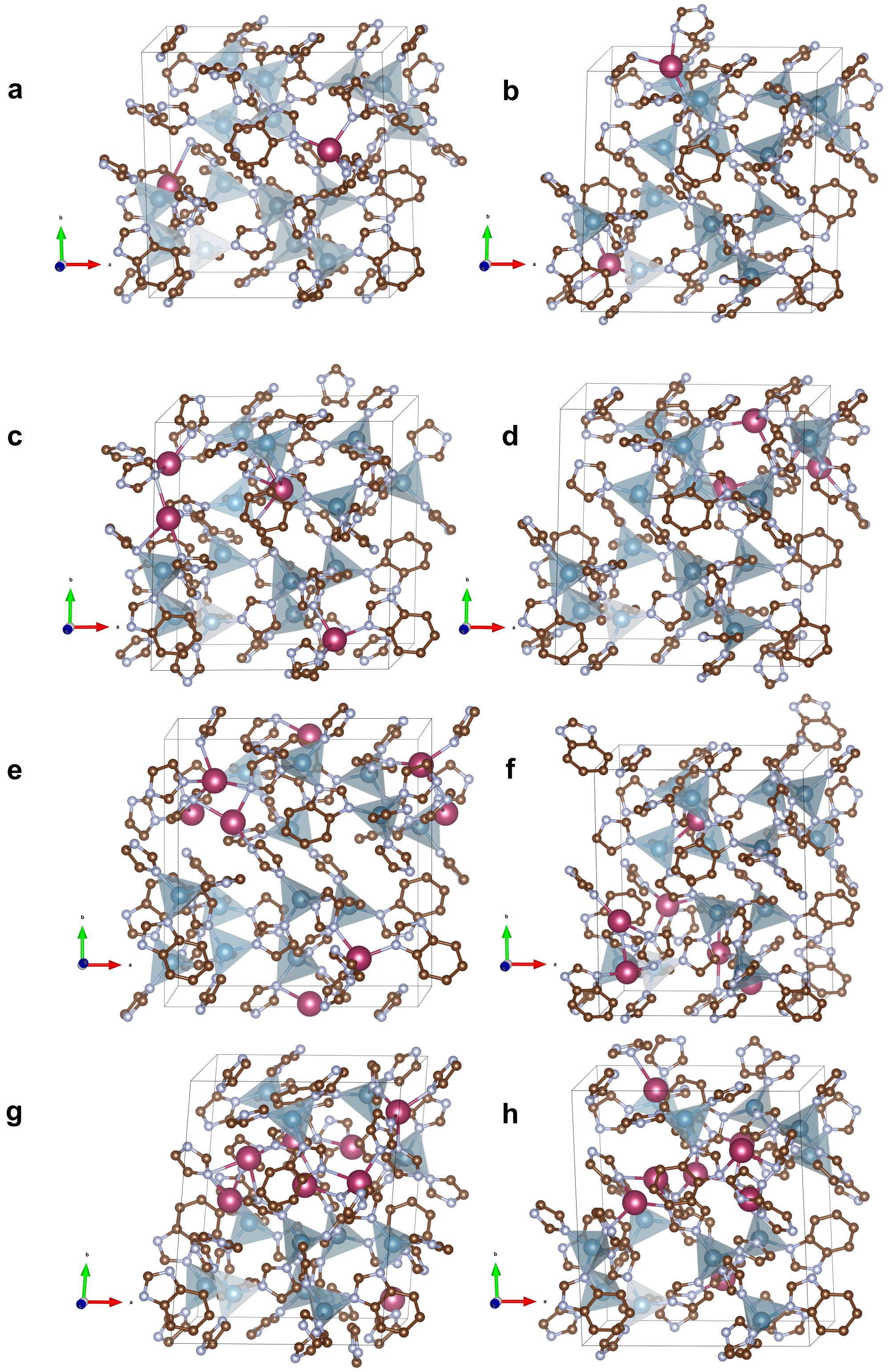

**Supplementary Figure 88.** Examples of geometry-optimised supercell structures of composition $(Na_{2y}Zn_{16-y})[(im)_{1.75}(bim)_{0.25}]_{16}$. Two examples are shown for each $y$ = 1 (a-b), 2 (c-d), 3 (e-f) and 4 (g-h). All supercells provided as .cell supplementary files. (Na, pink; Zn, dark blue; N, light blue; C, brown)

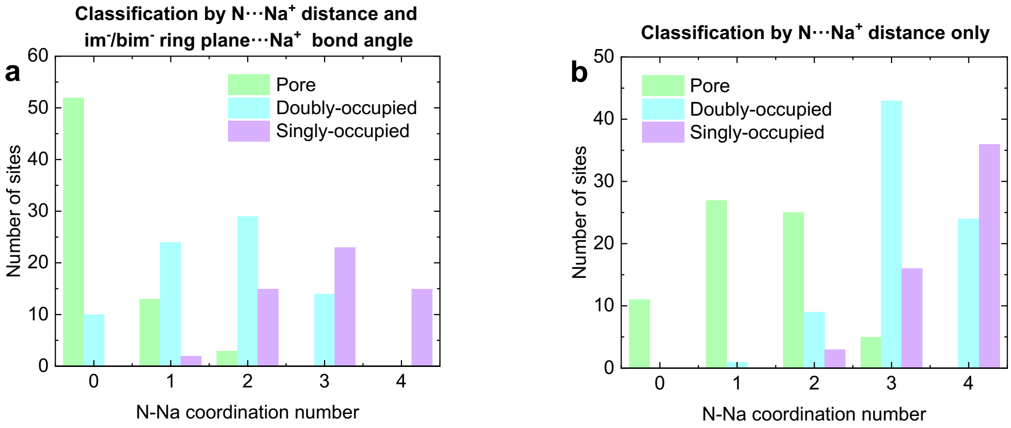


**Supplementary Figure 89**. Relationship between categorisation of geometry-optimised $Na^+$ as attached to the pore walls of *g*NaB$_x$ZIF-62, on doubly occupied or singly occupied node site and N–$Na^+$ coordination number, defined by N···$Na^+$ distance < 2.7 Å and the $im^-$/$bim^-$ ring plane···$Na^+$ angle < 30° (**a**) and N···$Na^+$ distance < 2.7 Å only (**b**). In the crystal structure of α-Na(bim) next-neighbour N···$Na^+$ distances range between 2.55 - 2.62 Å. [7]

**Supplementary Table 14.** Classification of DFT-simulated $Na^+$ coordination environments found in .cell files provided as supplementary files. N-$Na^+$ coordination number defined by distance only requires that N···$Na^+$ distance < 2.7 Å only for an N-$Na^+$ coordinate bond to be counted. N-$Na^+$ coordination number defined by distance and angle requires that both N···$Na^+$ distance < 2.7 Å and that a less than 30° angle between the plane of the $im^-$/$bim^-$ ring and $Na^+$ for a N-$Na^+$ coordinate bond to be counted. $Na^+$ site assignment: 1 = singly occupied node site, 2 = doubly occupied node site, 3 = pore wall site.

| Supercell | Na # (in .cell file) | N-$Na^+$ coordination (distance only) | N-$Na^+$ coordination (distance + angle) | $Na^+$ site assignment |
|---|---|---|---|---|
| ZIF-62_Na2_01 | 1 | 0 | 0 | 3 |
| ZIF-62_Na2_01 | 2 | 1 | 0 | 3 |
| ZIF-62_Na2_02 | 1 | 4 | 3 | 1 |
| ZIF-62_Na2_02 | 2 | 1 | 0 | 3 |
| ZIF-62_Na2_03 | 1 | 4 | 3 | 1 |
| ZIF-62_Na2_03 | 2 | 1 | 0 | 3 |
| ZIF-62_Na2_04 | 1 | 2 | 0 | 3 |
| ZIF-62_Na2_04 | 2 | 3 | 3 | 1 |
| ZIF-62_Na2_05 | 1 | 2 | 0 | 3 |
| ZIF-62_Na2_05 | 2 | 2 | 1 | 3 |
| ZIF-62_Na2_06 | 1 | 1 | 0 | 3 |
| ZIF-62_Na2_06 | 2 | 4 | 3 | 1 |
| ZIF-62_Na2_07 | 1 | 3 | 0 | 3 |
| ZIF-62_Na2_07 | 2 | 4 | 3 | 1 |
| ZIF-62_Na2_08 | 1 | 4 | 4 | 1 |
| ZIF-62_Na2_08 | 2 | 0 | 0 | 3 |
| ZIF-62_Na2_09 | 1 | 4 | 4 | 1 |
| ZIF-62_Na2_09 | 2 | 0 | 0 | 3 |
| ZIF-62_Na2_10 | 1 | 1 | 0 | 3 |
| ZIF-62_Na2_10 | 2 | 4 | 3 | 1 |
| ZIF-62_Na4_01 | 1 | 1 | 0 | 3 |
| ZIF-62_Na4_01 | 2 | 4 | 2 | 2 |
| ZIF-62_Na4_01 | 3 | 4 | 3 | 2 |
| ZIF-62_Na4_01 | 4 | 4 | 2 | 2 |
| ZIF-62_Na4_02 | 1 | 3 | 2 | 1 |
| ZIF-62_Na4_02 | 2 | 4 | 4 | 1 |
| ZIF-62_Na4_02 | 3 | 2 | 1 | 3 |
| ZIF-62_Na4_02 | 4 | 1 | 0 | 3 |
| ZIF-62_Na4_03 | 1 | 0 | 0 | 3 |
| ZIF-62_Na4_03 | 2 | 4 | 3 | 1 |
| ZIF-62_Na4_03 | 3 | 1 | 0 | 3 |
| ZIF-62_Na4_03 | 4 | 3 | 2 | 1 |
| ZIF-62_Na4_04 | 1 | 4 | 1 | 2 |
| ZIF-62_Na4_04 | 2 | 1 | 0 | 3 |
| ZIF-62_Na4_04 | 3 | 4 | 4 | 1 |
| ZIF-62_Na4_04 | 4 | 3 | 0 | 2 |
| ZIF-62_Na4_05 | 1 | 2 | 2 | 1 |
| ZIF-62_Na4_05 | 2 | 1 | 0 | 3 |
| ZIF-62_Na4_05 | 3 | 2 | 1 | 1 |
| ZIF-62_Na4_05 | 4 | 4 | 4 | 1 |
| ZIF-62_Na4_06 | 1 | 3 | 2 | 2 |
| ZIF-62_Na4_06 | 2 | 1 | 1 | 3 |
| ZIF-62_Na4_06 | 3 | 3 | 2 | 2 |
| ZIF-62_Na4_06 | 4 | 2 | 0 | 3 |
| ZIF-62_Na4_07 | 1 | 1 | 0 | 3 |
| ZIF-62_Na4_07 | 2 | 4 | 4 | 1 |
| ZIF-62_Na4_07 | 3 | 1 | 1 | 3 |
| ZIF-62_Na4_07 | 4 | 3 | 2 | 1 |
| ZIF-62_Na4_08 | 1 | 4 | 3 | 1 |
| ZIF-62_Na4_08 | 2 | 1 | 0 | 3 |
| ZIF-62_Na4_08 | 3 | 0 | 0 | 3 |
| ZIF-62_Na4_08 | 4 | 4 | 3 | 1 |
| ZIF-62_Na4_09 | 1 | 3 | 1 | 3 |
| ZIF-62_Na4_09 | 2 | 4 | 4 | 1 |
| ZIF-62_Na4_09 | 3 | 3 | 2 | 1 |
| ZIF-62_Na4_09 | 4 | 3 | 1 | 1 |
| ZIF-62_Na4_10 | 1 | 4 | 2 | 2 |
| ZIF-62_Na4_10 | 2 | 4 | 2 | 2 |
| ZIF-62_Na4_10 | 3 | 2 | 0 | 3 |
| ZIF-62_Na4_10 | 4 | 4 | 3 | 2 |
| ZIF-62_Na6_01 | 1 | 3 | 1 | 2 |
| ZIF-62_Na6_01 | 2 | 4 | 4 | 1 |
| ZIF-62_Na6_01 | 3 | 1 | 0 | 3 |
| ZIF-62_Na6_01 | 4 | 4 | 2 | 2 |
| ZIF-62_Na6_01 | 5 | 2 | 0 | 3 |
| ZIF-62_Na6_01 | 6 | 4 | 3 | 1 |
| ZIF-62_Na6_02 | 1 | 2 | 1 | 3 |

| | | | | |
|---|---|---|---|---|
| ZIF-62_Na6_02 | 2 | 4 | 3 | 2 |
| ZIF-62_Na6_02 | 3 | 4 | 4 | 1 |
| ZIF-62_Na6_02 | 4 | 1 | 0 | 3 |
| ZIF-62_Na6_02 | 5 | 3 | 2 | 1 |
| ZIF-62_Na6_02 | 6 | 3 | 3 | 2 |
| ZIF-62_Na6_03 | 1 | 3 | 1 | 2 |
| ZIF-62_Na6_03 | 2 | 4 | 3 | 2 |
| ZIF-62_Na6_03 | 3 | 2 | 1 | 3 |
| ZIF-62_Na6_03 | 4 | 3 | 2 | 2 |
| ZIF-62_Na6_03 | 5 | 4 | 2 | 2 |
| ZIF-62_Na6_03 | 6 | 2 | 0 | 3 |
| ZIF-62_Na6_04 | 1 | 4 | 3 | 2 |
| ZIF-62_Na6_04 | 2 | 2 | 2 | 3 |
| ZIF-62_Na6_04 | 3 | 4 | 2 | 2 |
| ZIF-62_Na6_04 | 4 | 2 | 1 | 3 |
| ZIF-62_Na6_04 | 5 | 2 | 1 | 2 |
| ZIF-62_Na6_04 | 6 | 3 | 1 | 2 |
| ZIF-62_Na6_05 | 1 | 4 | 3 | 1 |
| ZIF-62_Na6_05 | 2 | 3 | 3 | 1 |
| ZIF-62_Na6_05 | 3 | 4 | 2 | 1 |
| ZIF-62_Na6_05 | 4 | 1 | 0 | 3 |
| ZIF-62_Na6_05 | 5 | 3 | 3 | 2 |
| ZIF-62_Na6_05 | 6 | 2 | 1 | 2 |
| ZIF-62_Na6_06 | 1 | 4 | 3 | 2 |
| ZIF-62_Na6_06 | 2 | 1 | 0 | 3 |
| ZIF-62_Na6_06 | 3 | 2 | 1 | 2 |
| ZIF-62_Na6_06 | 4 | 3 | 2 | 2 |
| ZIF-62_Na6_06 | 5 | 3 | 2 | 2 |
| ZIF-62_Na6_06 | 6 | 3 | 1 | 2 |
| ZIF-62_Na6_07 | 1 | 4 | 0 | 2 |
| ZIF-62_Na6_07 | 2 | 2 | 1 | 2 |
| ZIF-62_Na6_07 | 3 | 4 | 3 | 1 |
| ZIF-62_Na6_07 | 4 | 3 | 3 | 1 |
| ZIF-62_Na6_07 | 5 | 1 | 0 | 3 |
| ZIF-62_Na6_07 | 6 | 1 | 0 | 3 |
| ZIF-62_Na6_08 | 1 | 4 | 3 | 1 |
| ZIF-62_Na6_08 | 2 | 4 | 4 | 1 |
| ZIF-62_Na6_08 | 3 | 4 | 2 | 1 |
| ZIF-62_Na6_08 | 4 | 1 | 0 | 3 |
| ZIF-62_Na6_08 | 5 | 2 | 0 | 3 |
| ZIF-62_Na6_08 | 6 | 1 | 0 | 3 |
| ZIF-62_Na6_09 | 1 | 3 | 2 | 2 |
| ZIF-62_Na6_09 | 2 | 3 | 0 | 2 |
| ZIF-62_Na6_09 | 3 | 3 | 0 | 3 |
| ZIF-62_Na6_09 | 4 | 2 | 1 | 2 |
| ZIF-62_Na6_09 | 5 | 2 | 2 | 1 |
| ZIF-62_Na6_09 | 6 | 3 | 2 | 2 |
| ZIF-62_Na6_10 | 1 | 4 | 1 | 2 |
| ZIF-62_Na6_10 | 2 | 3 | 2 | 2 |
| ZIF-62_Na6_10 | 3 | 0 | 0 | 3 |
| ZIF-62_Na6_10 | 4 | 4 | 3 | 1 |
| ZIF-62_Na6_10 | 5 | 3 | 3 | 1 |
| ZIF-62_Na6_10 | 6 | 1 | 0 | 3 |
| ZIF-62_Na8_01 | 1 | 3 | 1 | 2 |
| ZIF-62_Na8_01 | 2 | 2 | 1 | 3 |
| ZIF-62_Na8_01 | 3 | 4 | 1 | 2 |
| ZIF-62_Na8_01 | 4 | 3 | 2 | 2 |
| ZIF-62_Na8_01 | 5 | 0 | 0 | 3 |
| ZIF-62_Na8_01 | 6 | 3 | 2 | 2 |
| ZIF-62_Na8_01 | 7 | 4 | 4 | 1 |
| ZIF-62_Na8_01 | 8 | 3 | 3 | 1 |
| ZIF-62_Na8_02 | 1 | 4 | 3 | 1 |
| ZIF-62_Na8_02 | 2 | 3 | 1 | 2 |
| ZIF-62_Na8_02 | 3 | 2 | 0 | 3 |
| ZIF-62_Na8_02 | 4 | 4 | 4 | 1 |
| ZIF-62_Na8_02 | 5 | 2 | 0 | 3 |
| ZIF-62_Na8_02 | 6 | 2 | 0 | 3 |
| ZIF-62_Na8_02 | 7 | 4 | 2 | 2 |
| ZIF-62_Na8_02 | 8 | 3 | 2 | 2 |
| ZIF-62_Na8_03 | 1 | 2 | 1 | 2 |
| ZIF-62_Na8_03 | 2 | 3 | 3 | 2 |
| ZIF-62_Na8_03 | 3 | 3 | 2 | 2 |
| ZIF-62_Na8_03 | 4 | 1 | 0 | 3 |
| ZIF-62_Na8_03 | 5 | 4 | 1 | 2 |
| ZIF-62_Na8_03 | 6 | 4 | 3 | 2 |
| ZIF-62_Na8_03 | 7 | 3 | 2 | 2 |
| ZIF-62_Na8_03 | 8 | 3 | 2 | 2 |
| ZIF-62_Na8_04 | 1 | 3 | 0 | 2 |
| ZIF-62_Na8_04 | 2 | 3 | 1 | 3 |
| ZIF-62_Na8_04 | 3 | 3 | 2 | 2 |
| ZIF-62_Na8_04 | 4 | 3 | 2 | 2 |
| ZIF-62_Na8_04 | 5 | 3 | 2 | 1 |
| ZIF-62_Na8_04 | 6 | 4 | 2 | 1 |

| | | | | |
|---|---|---|---|---|
| ZIF-62_Na8_04 | 7 | 4 | 3 | 1 |
| ZIF-62_Na8_04 | 8 | 3 | 0 | 3 |
| ZIF-62_Na8_05 | 1 | 3 | 3 | 2 |
| ZIF-62_Na8_05 | 2 | 2 | 1 | 3 |
| ZIF-62_Na8_05 | 3 | 2 | 0 | 3 |
| ZIF-62_Na8_05 | 4 | 2 | 2 | 3 |
| ZIF-62_Na8_05 | 5 | 3 | 3 | 1 |
| ZIF-62_Na8_05 | 6 | 3 | 1 | 2 |
| ZIF-62_Na8_05 | 7 | 2 | 0 | 3 |
| ZIF-62_Na8_05 | 8 | 3 | 1 | 2 |
| ZIF-62_Na8_06 | 1 | 2 | 0 | 2 |
| ZIF-62_Na8_06 | 2 | 3 | 1 | 2 |
| ZIF-62_Na8_06 | 3 | 3 | 2 | 2 |
| ZIF-62_Na8_06 | 4 | 1 | 1 | 3 |
| ZIF-62_Na8_06 | 5 | 3 | 0 | 2 |
| ZIF-62_Na8_06 | 6 | 4 | 3 | 2 |
| ZIF-62_Na8_06 | 7 | 4 | 4 | 1 |
| ZIF-62_Na8_06 | 8 | 2 | 1 | 2 |
| ZIF-62_Na8_07 | 1 | 0 | 0 | 3 |
| ZIF-62_Na8_07 | 2 | 3 | 2 | 1 |
| ZIF-62_Na8_07 | 3 | 0 | 0 | 3 |
| ZIF-62_Na8_07 | 4 | 4 | 2 | 1 |
| ZIF-62_Na8_07 | 5 | 3 | 1 | 2 |
| ZIF-62_Na8_07 | 6 | 2 | 0 | 3 |
| ZIF-62_Na8_07 | 7 | 2 | 0 | 2 |
| ZIF-62_Na8_07 | 8 | 4 | 4 | 1 |
| ZIF-62_Na8_08 | 1 | 2 | 0 | 3 |
| ZIF-62_Na8_08 | 2 | 1 | 1 | 2 |
| ZIF-62_Na8_08 | 3 | 1 | 1 | 3 |
| ZIF-62_Na8_08 | 4 | 1 | 0 | 3 |
| ZIF-62_Na8_08 | 5 | 3 | 3 | 2 |
| ZIF-62_Na8_08 | 6 | 3 | 3 | 1 |
| ZIF-62_Na8_08 | 7 | 3 | 3 | 1 |
| ZIF-62_Na8_08 | 8 | 4 | 2 | 1 |
| ZIF-62_Na8_09 | 1 | 2 | 2 | 3 |
| ZIF-62_Na8_09 | 2 | 2 | 0 | 3 |
| ZIF-62_Na8_09 | 3 | 3 | 2 | 2 |
| ZIF-62_Na8_09 | 4 | 4 | 2 | 1 |
| ZIF-62_Na8_09 | 5 | 3 | 1 | 2 |
| ZIF-62_Na8_09 | 6 | 3 | 1 | 2 |
| ZIF-62_Na8_09 | 7 | 4 | 2 | 2 |
| ZIF-62_Na8_09 | 8 | 0 | 0 | 3 |
| ZIF-62_Na8_10 | 1 | 3 | 0 | 2 |
| ZIF-62_Na8_10 | 2 | 3 | 2 | 2 |
| ZIF-62_Na8_10 | 3 | 3 | 2 | 2 |
| ZIF-62_Na8_10 | 4 | 3 | 0 | 2 |
| ZIF-62_Na8_10 | 5 | 4 | 3 | 1 |
| ZIF-62_Na8_10 | 6 | 0 | 0 | 3 |
| ZIF-62_Na8_10 | 7 | 4 | 3 | 2 |
| ZIF-62_Na8_10 | 8 | 4 | 1 | 2 |

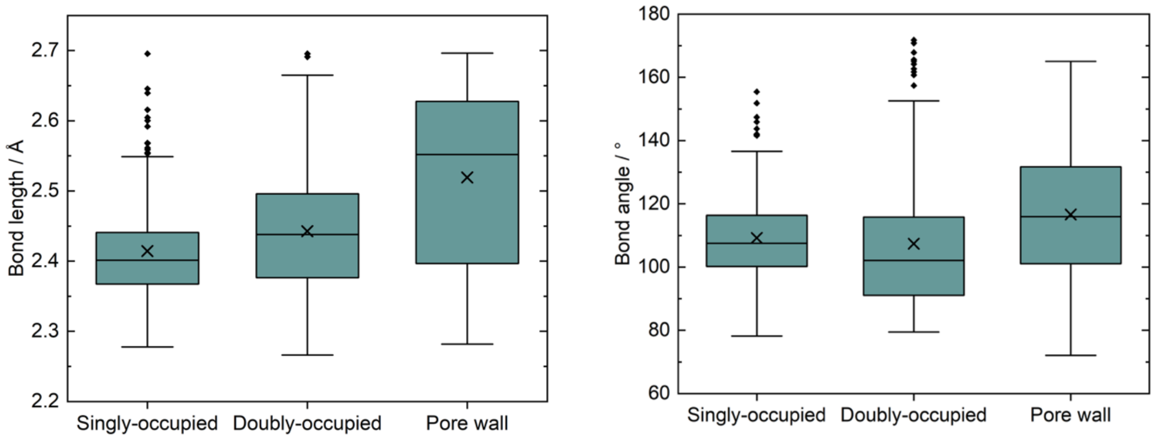


**Supplementary Figure 90.** Box plots illustrating the distribution of bond lengths and bond angles for the sodium ions categorised according to the identified three distinct Na sites (total number of counted sites: 55 singly-occupied, 77 doubly-occupied, 68 pore wall sites). The box represents the interquartile range (IQR) from the first (Q1) to the third quartile (Q3) and covers 50 % of the data points. The line inside indicates the median and the cross indicates the average value of the dataset. The whiskers extend to the smallest and largest values within 1.5 × IQR from the quartiles. Points beyond the whiskers are considered outliers. The broad distribution of bond angles for sodium ions in pore wall sites is a consequence of the large range of coordination numbers found for such sodium sites.

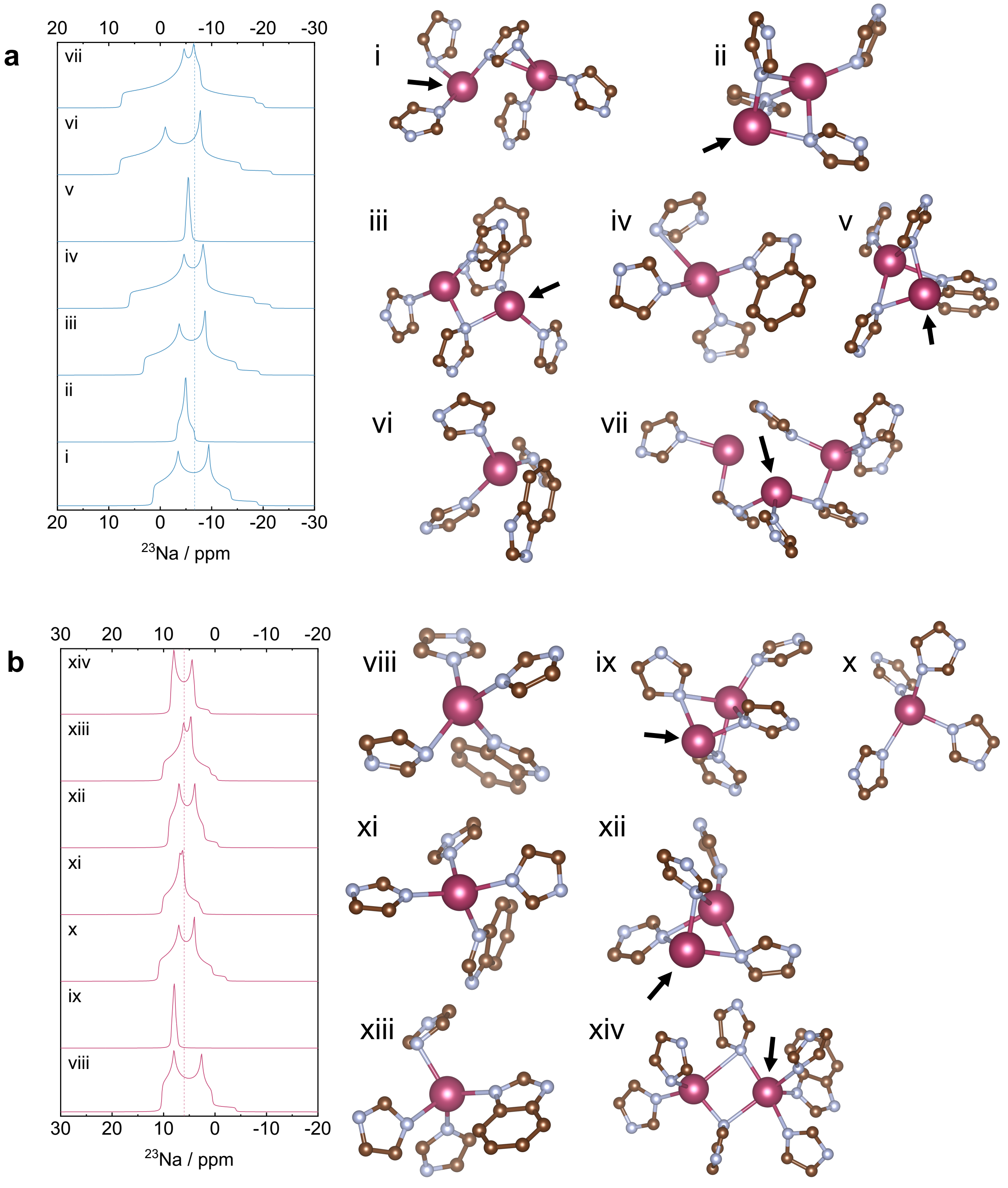


**Supplementary Figure 91**. DFT-simulated $Na^+$ coordination environments giving rise to predicted $^{23}$Na NMR spectra with maximum intensity in the regions around –6.3 ± 3 ppm (**a**) and 6.8 ± 3 ppm (**b**). Where multiple $Na^+$ are shown in a single environment, an arrow is used to show the $Na^+$ corresponding to the predicted $^{23}$Na spectrum of interest. (Na, pink; N, light blue; C, brown; hydrogens were omitted for clarity).

# S15. Solid-State Nuclear Magnetic Resonance Spectroscopy

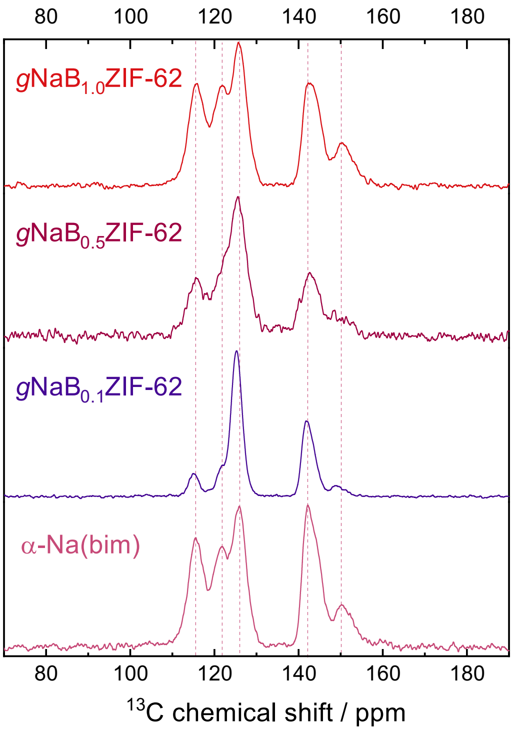


**Supplementary Figure 92.** $^{1}$H-decoupled $^{1}$H-$^{13}$C cross-polarisation MAS NMR (11.7 T, 12 kHz) of *g*NaB$_x$ZIF-62 and its organic salt precursors. Gradual increase in Na(bim) content in *g*NaB$_x$ZIF-62 is reflected in an increase in the intensity of $^{13}$C signals corresponding to bim$^{-}$. No other signals are observed, which might correspond to organic impurities in the modified MOF glass materials.

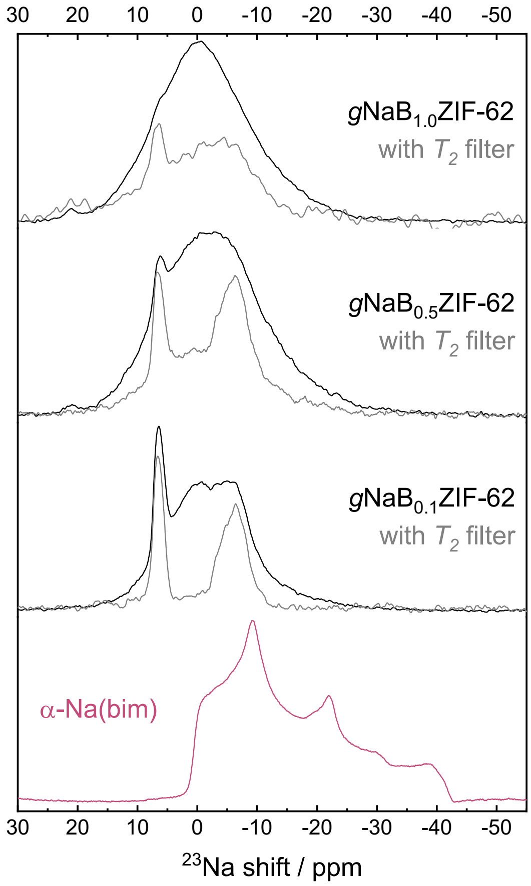


**Supplementary Figure 93.** $^{23}$Na MAS NMR spectra (20.0 T, 20 kHz) of α-Na(bim) and *g*NaB$_x$ZIF-62 with $x$ = 0.1, 0.5 and 1.0. Spectra of modified MOF glasses are shown with a short (0.05 ms) and long (12.8 ms) echo delay to filter spectral components with short spin-spin relaxation times ($T_2$) (see Supplementary Figure 78).

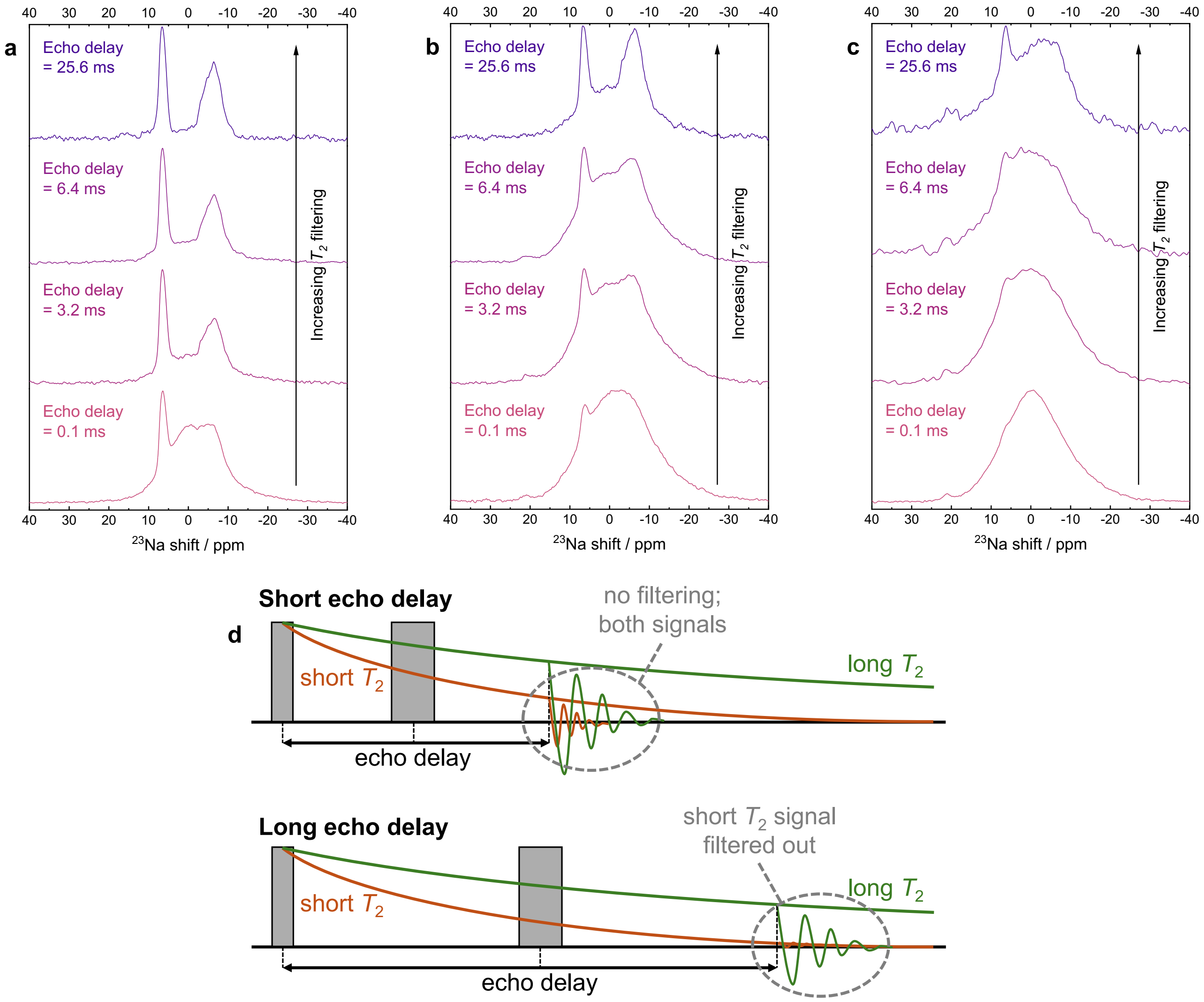


**Supplementary Figure 94.** Spin-spin relaxation time ($T_2$) filtering by increasing echo delay time of $^{23}$Na MAS NMR (20.0 T, 20 kHz) of *g*NaB$_x$ZIF-62 with $x$ = 0.1 (**a**), 0.5 (**b**) and 1.0 (**c**) acquired *via* Hahn echo pulse sequence. **d**, Schematic Supplementary Figure howing the effect of altering echo delay on the signal intensity of Na environments with short and long $T_2$ values.

Supercells with $2Na^{+}$ ($y$ = 1)

**a**

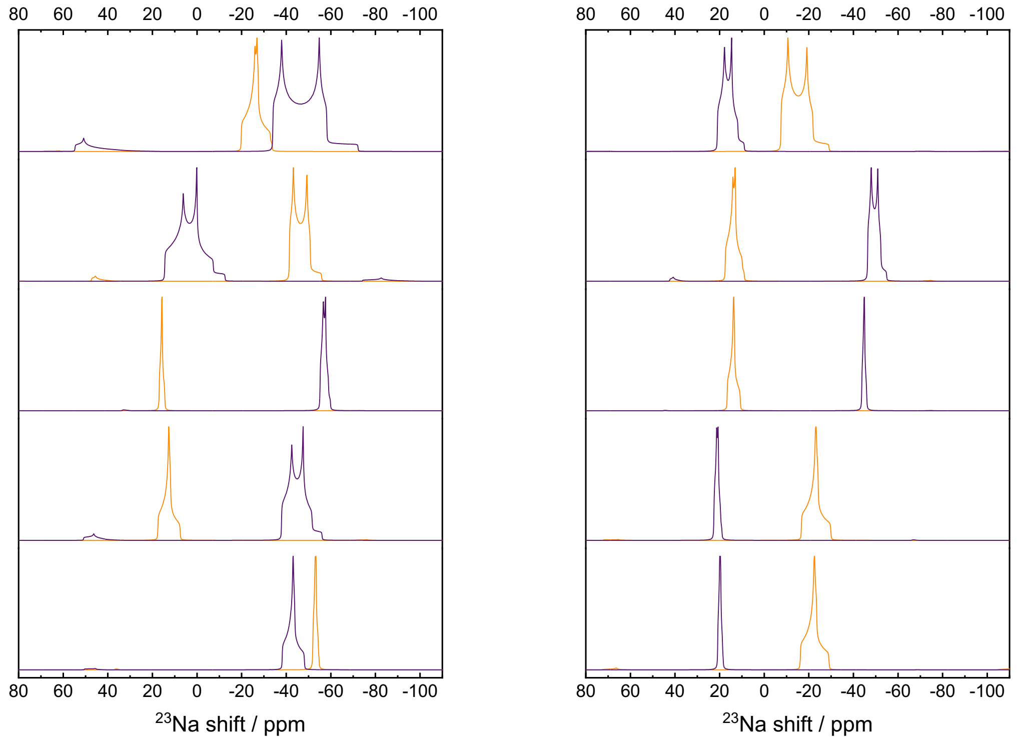


Supercells with $4Na^{+}$ ($y$ = 2)

**b**

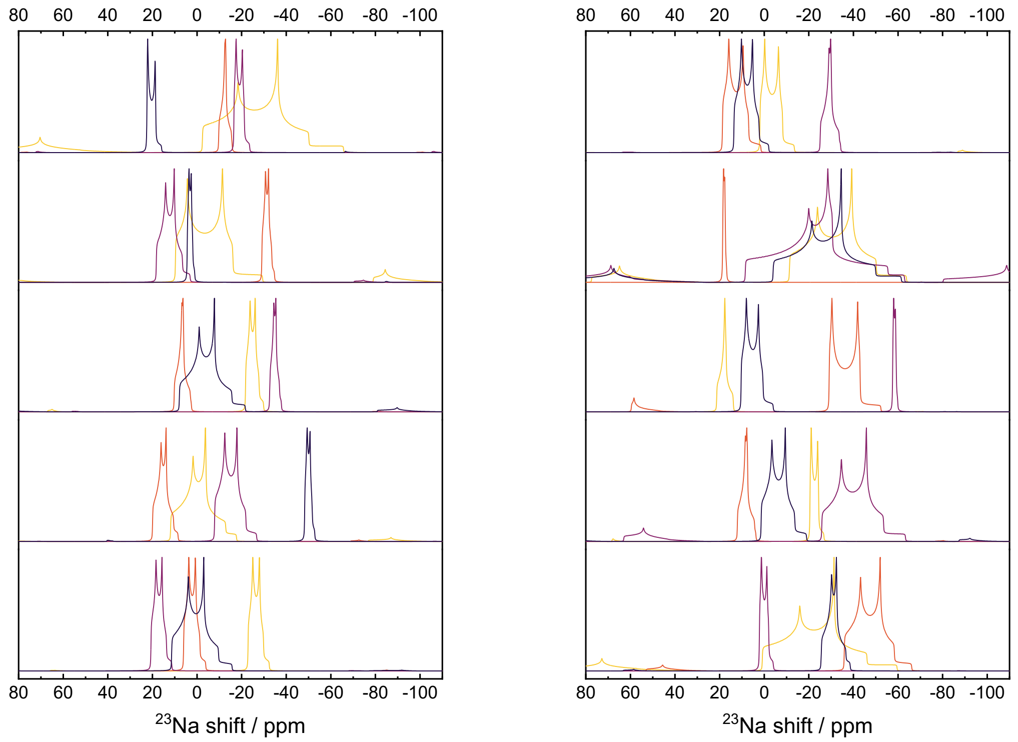

## Supercells with 6Na+ (y = 3)

**c**

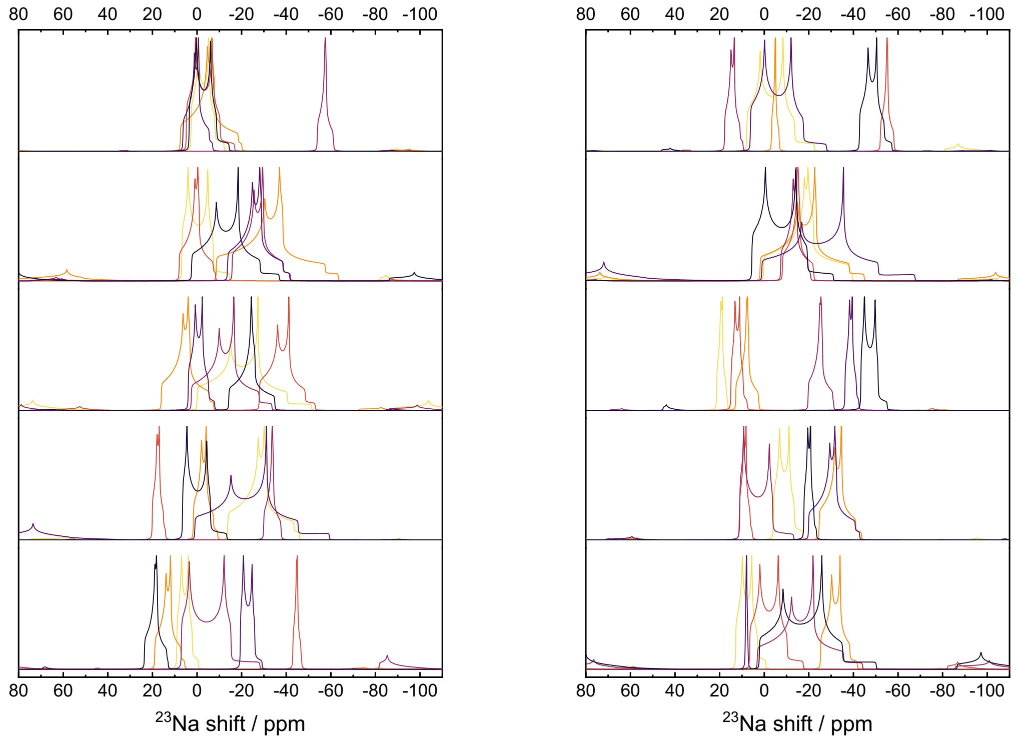


## Supercells with 8Na+ (y = 4)

**d**

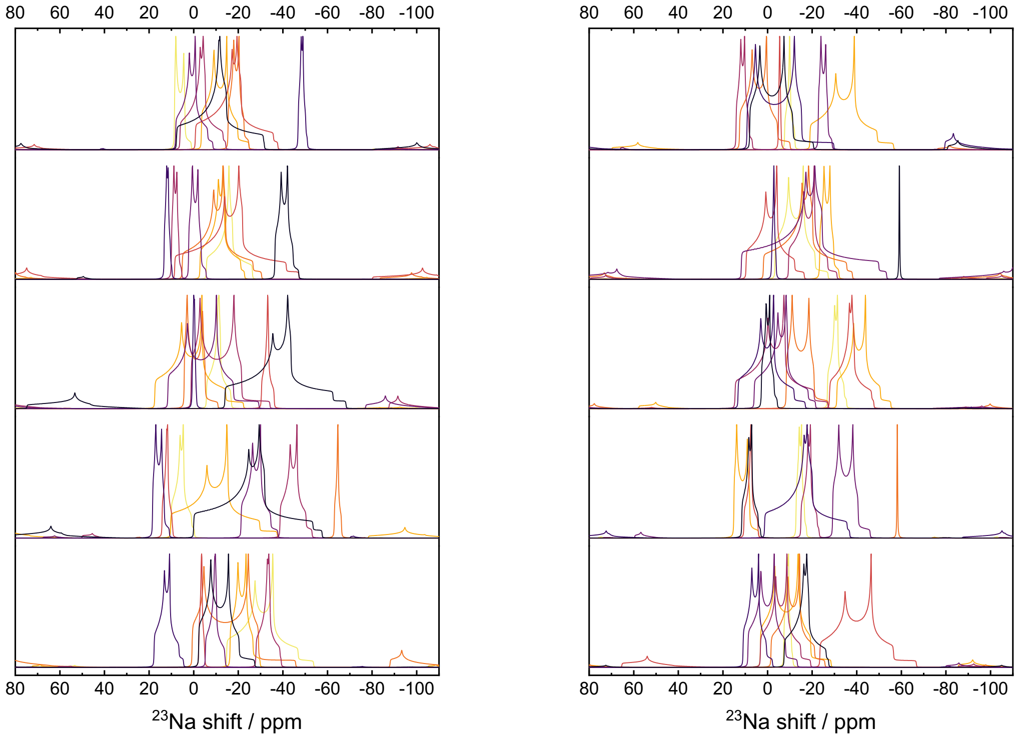


**Supplementary Figure 95.** Predicted $^{23}$Na spectra (20.0 T, MAS = 20 kHz) for all DFT-simulated $Na^+$ environments, simulated using calculated chemical shielding and electric field gradient (EFG) tensors. Spectra are grouped by supercell, each with composition $(Na_{2y}Zn_{16-y})[(im)_{1.75}(bim)_{0.25}]_{16}$, with $y$ = 1 (a), 2 (b), 3 (c) and 4 (d).

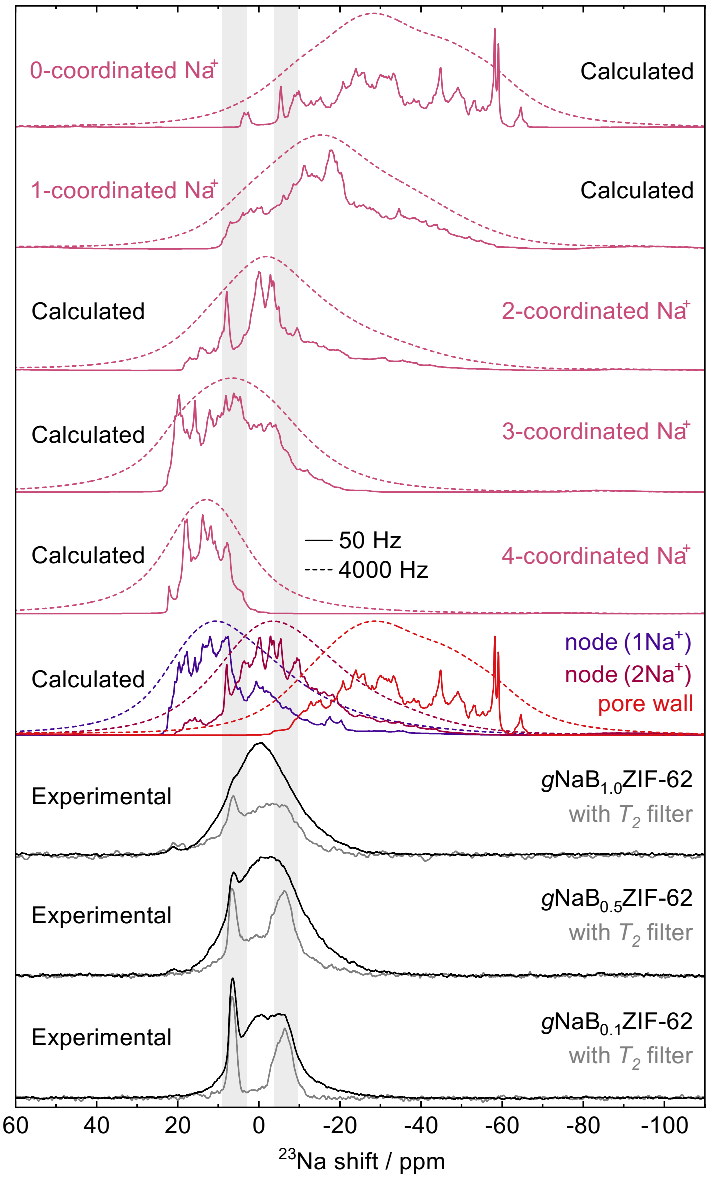


**Supplementary Figure 96**. This figure is identical to that found in the main text (**Figure 5a-i**), except that N-$Na^+$ coordination is defined by both N···$Na^+$ distance and the angle between the line between N and $Na^+$ and the plane of the $im^-$/$bim^-$ ring < 30°, as described below. Experimental $^{23}$Na NMR (20.0T, 20 kHz MAS) spectra of *g*NaB$_x$ZIF-62 with $x$ = 0.1, 0.5, 1.0 (**a-c**). Spectra acquired with both a short (0.1 ms, black) and long (25.6 ms, grey) echo delay are shown, with the latter effectively filtering components in the spectra with short spin-spin relaxation times ($T_2$). Predicted $^{23}$Na NMR spectra of $Na^+$ sites in DFT-simulated *g*NaB$_x$ZIF-62 structures, categorised by $Na^+$ occupation in the gNaBxZIF-62 structure (1$Na^+$ and 2$Na^+$ correspond to singly and doubly occupied node sites, respectively) (**d**) and N-$Na^+$ coordination number (**e-i**).

Doubly occupied $Na^+$ sites often resemble those observed in the α-Na(bim) structure (**Figure 5l**), with limited direct N–$Na^+$ coordination. As such, the bonding interactions present in many doubly occupied node sites may not be appropriately represented by

the definition of N–$Na^+$ coordination using the N···$Na^+$ distance only (**Figure 5e-i**). To investigate this, we introduced a second criterion of N–$Na^+$ coordination and recategorised all 200 simulated $Na^+$ sites. This criterion introduces a requirement for a degree of covalency (directionality) in N–$Na^+$ coordinate bonding by requiring that both (i) N···$Na^+$ distance < 2.7 Å, and (ii) the angle between the line between N and $Na^+$ and the plane of the $im^-$/$bim^-$ ring < 30°. In **Supplementary Figure 96** we show cumulative predicted $^{23}$Na spectra categorized using this new classification system and find that there is even less spectral overlap between the experimental spectra and the predicted $^{23}$Na region corresponding to 4-coordinate $Na^+$ sites. Instead, lower coordination numbers become experimentally relevant, further supporting our inference that $Na^+$ incorporation into *g*ZIF-62 distorts the MOF substantially, consistent with reduced network connectivity and partial pore collapse

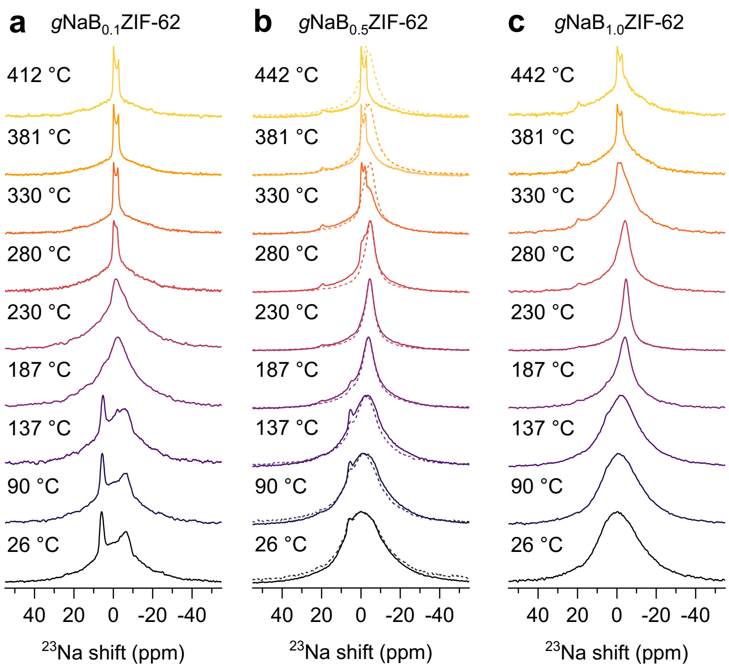


**Supplementary Figure 97**. Variable temperature solid-state $^{23}$Na MAS NMR (20.0T, 4 kHz magic angle spinning) spectra of *g*NaB$_x$ZIF-62 with $x$ = 0.1 (**a**), 0.5 (**b**) and 1.0 (**c**). Solid lines show spectra aquired *via* a Hahn echo pulse sequence (250 μs echo delay). Dashed lines show spectra acquired *via* a Bloch decay pulse sequence.

To further understand how the local structure of modified ZIF-62 glasses evolves under heating we conduct variable temperature $^{23}$Na MAS NMR (20.0 T, 4 kHz) on *g*NaB$_x$ZIF-62 with $x$ = 0.1, 0.5 and 1.0 (**Supplementary Figure 97**). For all three compositions, we observe three distinct temperature regimes. As temperature increases up to $T_g$,

$^{23}Na$ spectra narrow, indicating increasing flexibility in the MOF prior to reaching the glass transition. Above $T_g$, a single peak centred at –4.5 ppm emerges with a still narrower FWHM. We observe that the intensity of this peak in echo experiments progressively decreases as the temperature increases and, for the highest temperatures, is substantially lower compared to pulse-acquire (Bloch decay) experiments. This result indicates that the spin-spin relaxation time of the broad component decreases with increasing temperature, which we attribute to the onset of $Na^+$ diffusion in the material. At the highest temperatures (>230 °C), a new $^{23}Na$ signal is apparent, displaying a pronounced quadrupolar line shape. Although present for all compositions investigated, the intensity of this high-temperature peak relative to the broad component at –4.5 ppm is greatest for $x$ = 0.1. We attribute this peak to a distinct binding mode, or phase, which is present throughout but only becomes visible when the dominant broader component is suppressed due to $T_2$ shortening.

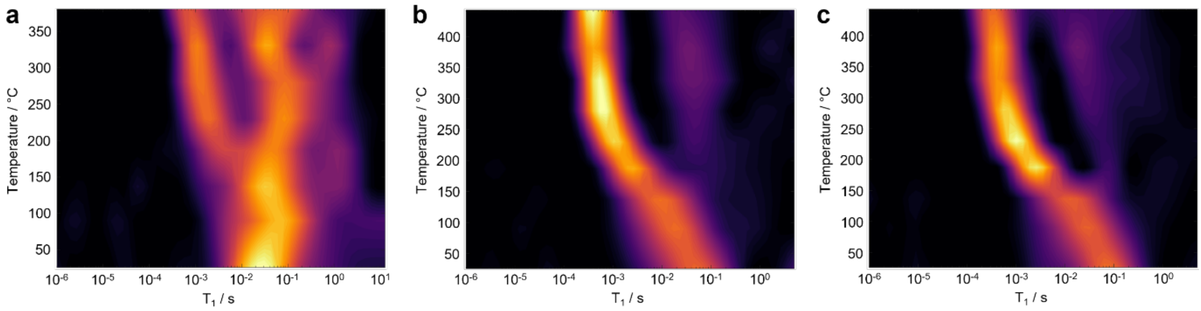


**Supplementary Figure 98**. Inverse Laplace transform fitting of $^{23}Na$ spin-lattice time ($T_1$) as a function of temperature for *g*NaB$_x$ZIF-62, $x$ = 0.1 (**a**), 0.5 (**b**), 1.0 (**c**). The colour scale from black to yellow represents intensity, with black indicating low intensity and yellow indicating high intensity. Below $T_g$, a single $^{23}Na$ $T_1$ is found for all three materials, while above $T_g$,a second component with notably longer $T_1$ is observed.

# S16. Water Leaching Experiments

## S16.1. Optical Microscopy Images

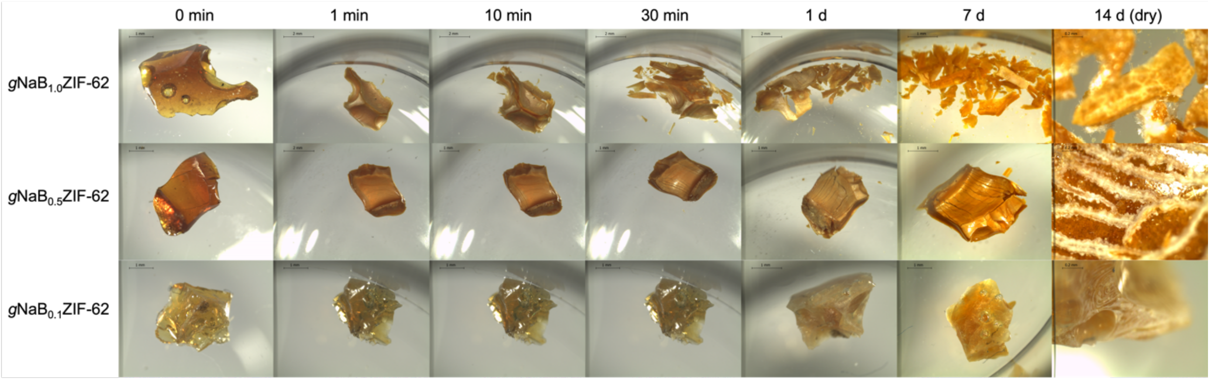


**Supplementary Figure 99.** Microscope images of *g*Na$B_x$ZIF-62 ($x$ = 1.0, 0.5, 0.1) displaying their reaction with water over a period of two weeks. The images on the right show a zoom into the reaction products after water evaporation (after 14 days). Phase separation into a water-soluble colourless phase (presumably hydrated Na(bim)/Na(im)) and a water-insoluble dark yellowish ZIF glass phase is evident. After 14 days, the remaining liquid was evaporated at room temperature (far-right image). The remaining solid material was washed with water and investigated by PXRD (see Supplementary Figure 101).

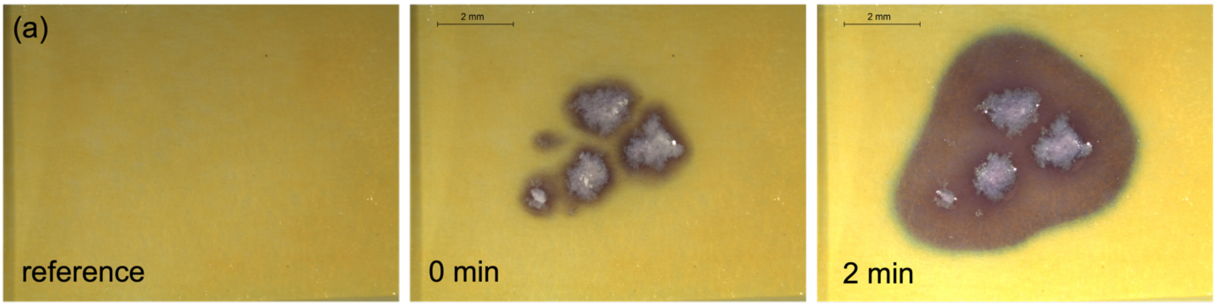


**Supplementary Figure 100.** Microscope images of the reaction of powdered *g*Na$B_{0.3}$ZIF-62 on wetted pH indicator paper over the course of 2 minutes. The pH indicator covers a pH range from pH 7 (yellow), pH 10 (green) up to pH 14 (dark brown).

## S16.2. X-Ray Diffraction Measurements

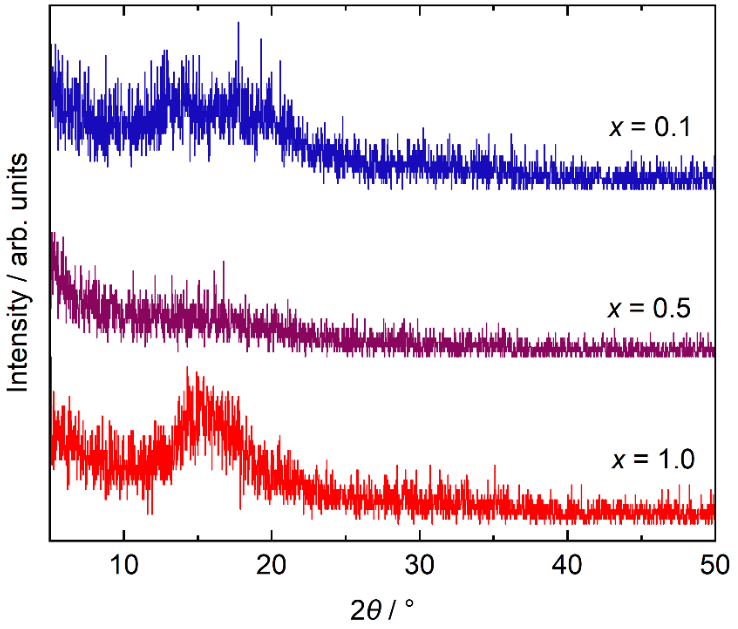


**Supplementary Figure 101.** PXRD patterns of *g*$NaB_xZIF$-62 materials with modifier contents *x* after two weeks of water treatment and subsequent washing with water (see Supplementary Figure 99). Diffraction patterns were recorded with $CuK_α$ radiation.

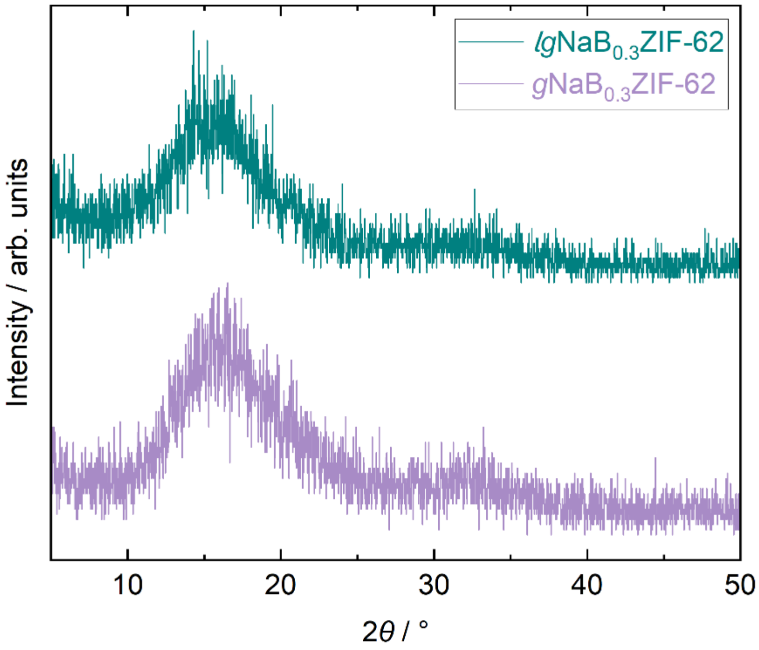


**Supplementary Figure 102**. PXRD pattern of *g*$NaB_{0.3}ZIF$-62 before and after (*lg*$NaB_{0.3}ZIF$-62) the water treatment. Diffraction patterns were recorded with $CuK_α$ radiation.

## S16.3. Solution $^{1}$H NMR Spectroscopy

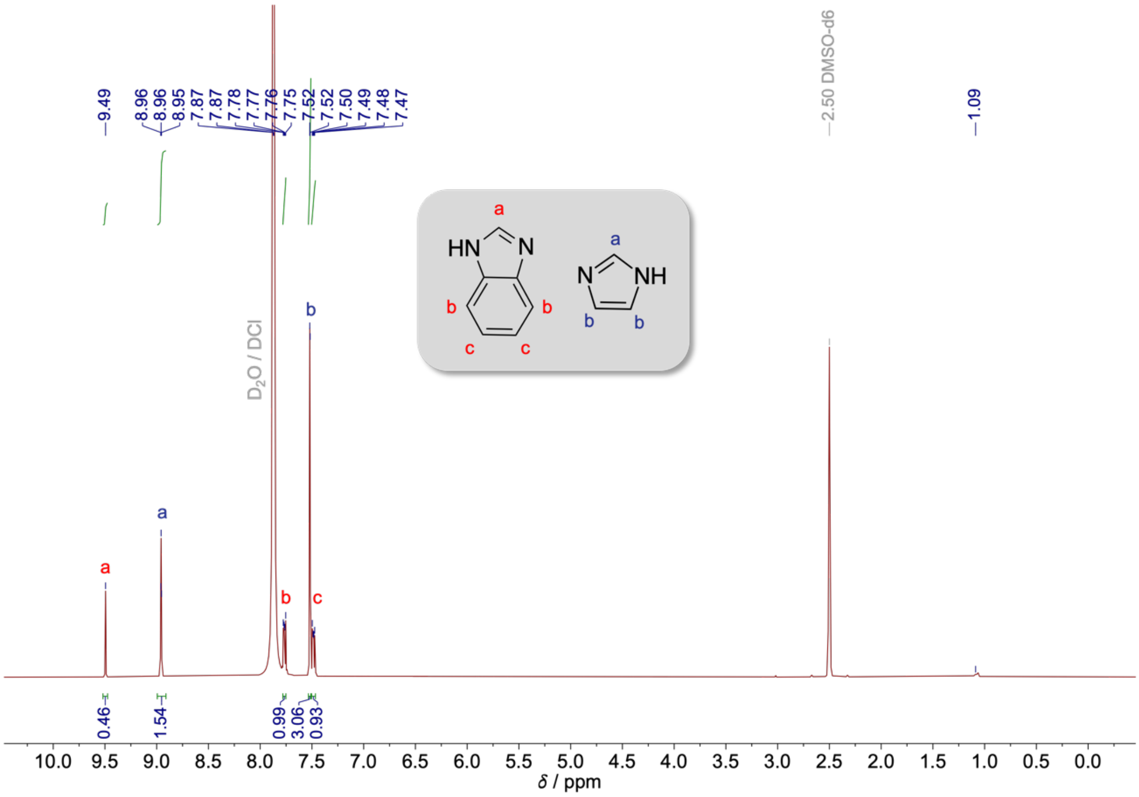


**Supplementary Figure 103.** $^{1}$H NMR spectrum of *ag*NaB$_{0.3}$ZIF-62 obtained via water-leaching of *g*NaB$_{0.3}$ZIF-62. The integrated signals suggest a chemical composition of Zn(im)$_{1.5}$(bim)$_{0.5}$ under the assumption of complete sodium ion removal. The material was dissolved in DMSO-$d_6$ and DCl/$D_2O$ (35 wt%, one drop, <0.1 mL).

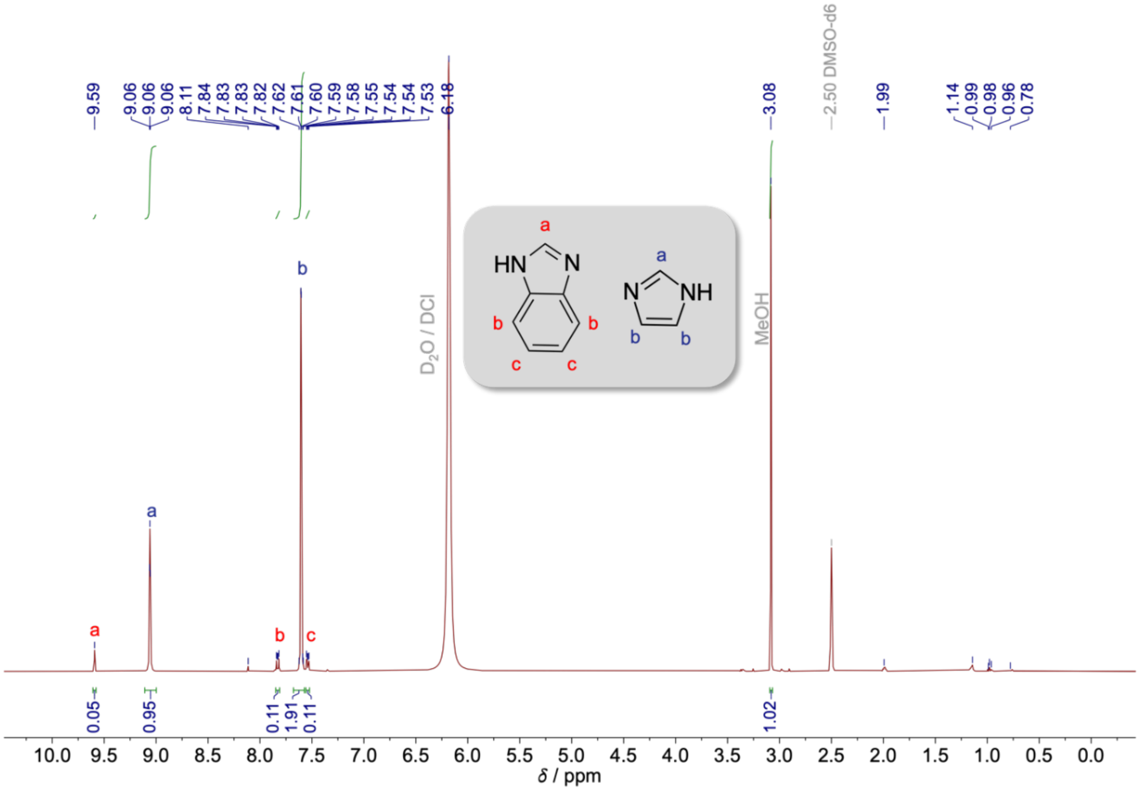


**Supplementary Figure 104.** $^{1}$H NMR spectrum of the leaching products obtained from the washing solution ($H_2O$, MeOH) as the result of the preparation of *lg*$NaB_{0.3}$ZIF-62 (prepared in ~250 mg scale. After solvent removal in a rotary evaporator at 60 °C the remaining solid material was dissolved in DMSO-$d_6$ and DCl/$D_2O$ (35 wt%, one drop, <0.1 mL).

## S16.4. SEM Imaging

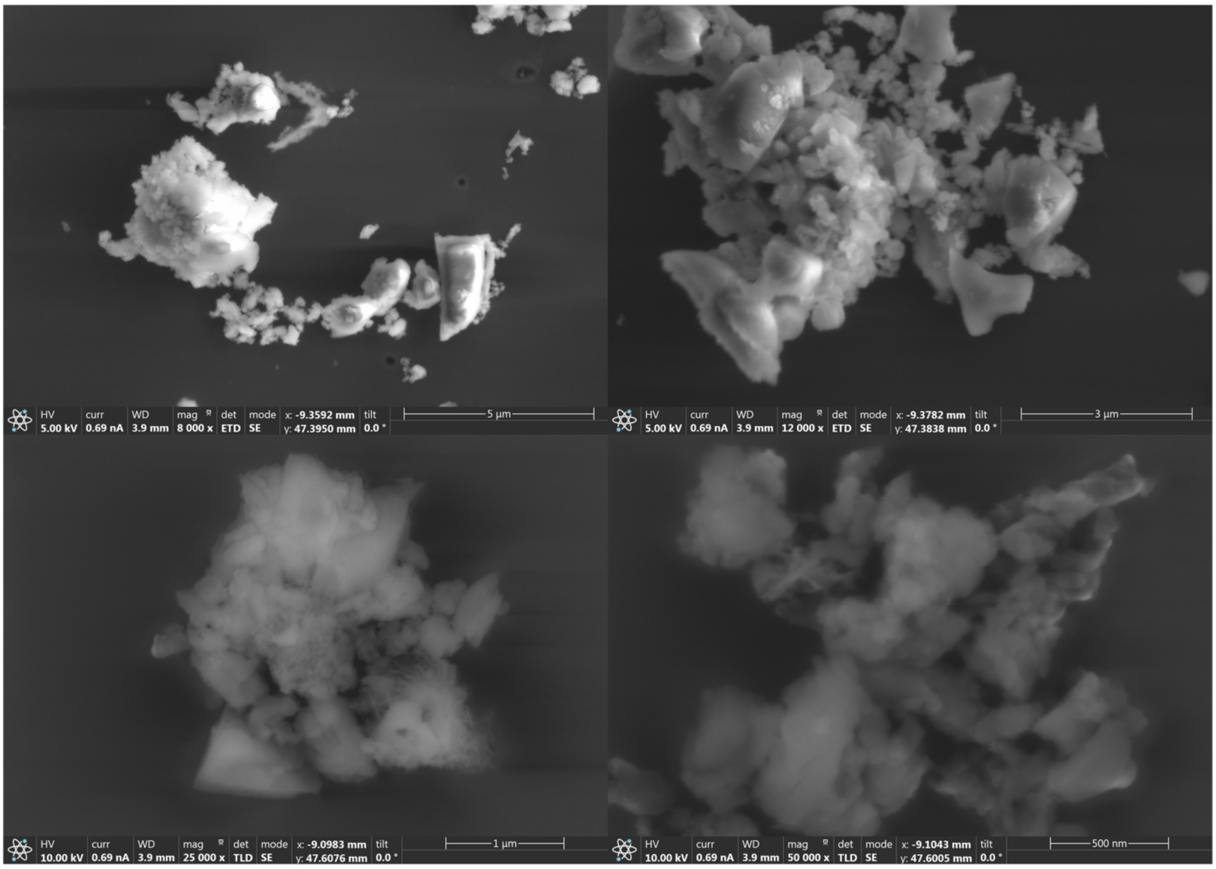


**Supplementary Figure 105.** SEM images of *lg*$NaB_{0.3}$ZIF-62 with different magnifications.

## S16.5. Infrared Spectroscopy

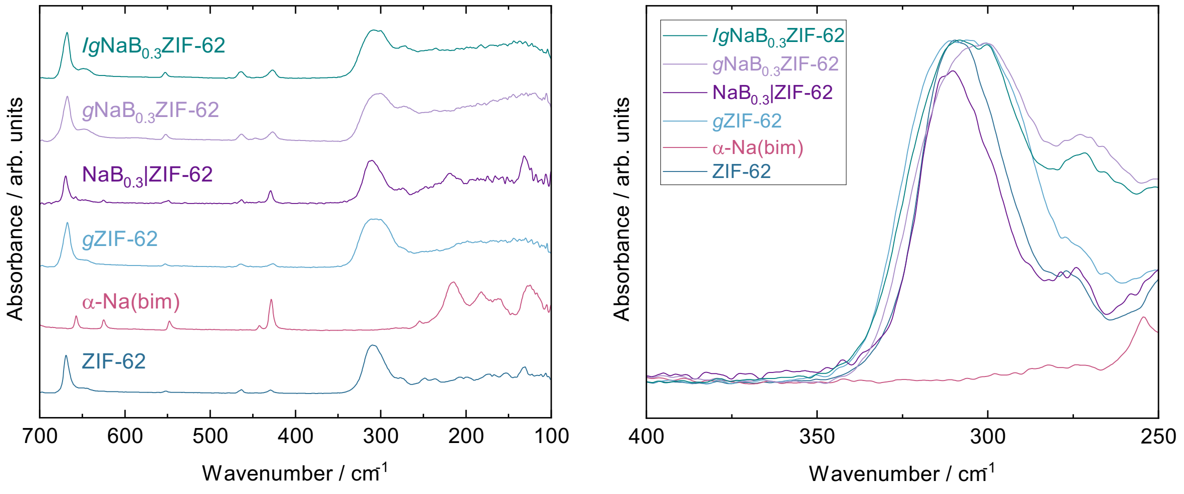


**Supplementary Figure 106.** Far-infrared spectra of ZIF-62, the corresponding glass *g*ZIF-62, the modifier α-Na(bim), the physical mixture of modifier and glass former $NaB_{0.3}$|ZIF-62, the corresponding glass of the physical mixture *g*$NaB_{0.3}$ZIF-62 and the glass after the water-leaching treatment *lg*$NaB_{0.3}$ZIF-62. The figure on the right shows a zoom into the wavenumber range of the [$ZnN_4$] tetrahedra vibration.

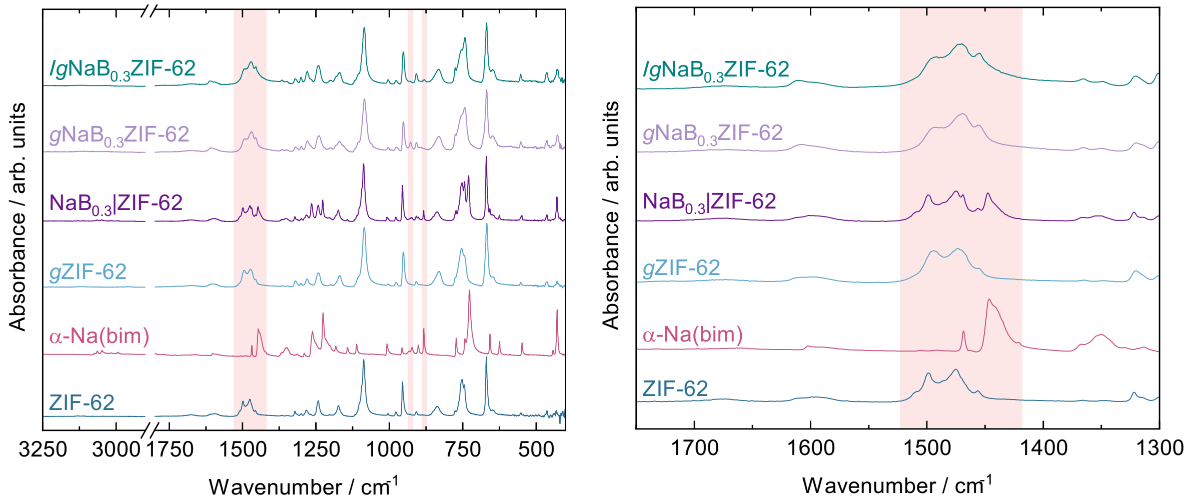


**Supplementary Figure 107**. Mid-infrared spectra of ZIF-62, the corresponding glass *g*ZIF-62, the modifier α-Na(bim), the physical mixture of modifier and glass former $NaB_{0.3}$|ZIF-62, the corresponding glass of the physical mixture *g*$NaB_{0.3}$ZIF-62 and the glass after the water-leaching treatment *lg*$NaB_{0.3}$ZIF-62. The figure on the right shows a zoom into the wavenumber range from 1800 to 1300 $cm^{-1}$.

## S16.6. X-ray Pair Distribution Function Analysis

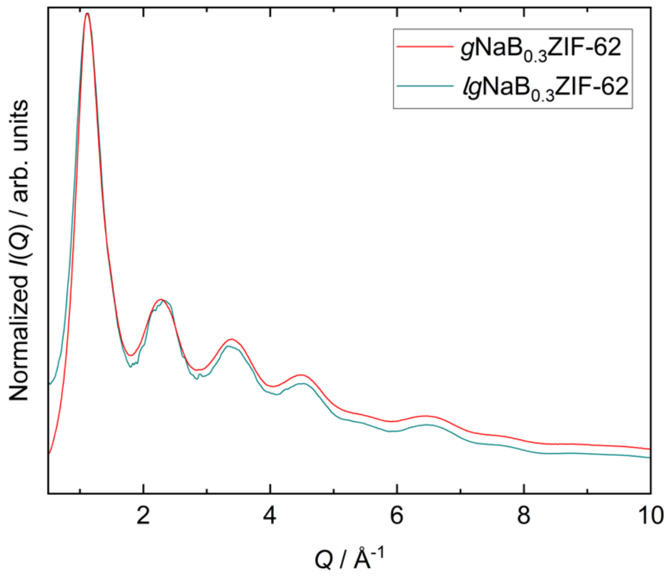


**Supplementary Figure 108.** Normalized, background-subtracted and scattering-corrected diffraction data collected for the PDF analysis of *g*NaB$_{0.3}$ZIF-62 and *lg*NaB$_{0.3}$ZIF-62. The data show no shift in the FSDP after water leaching.

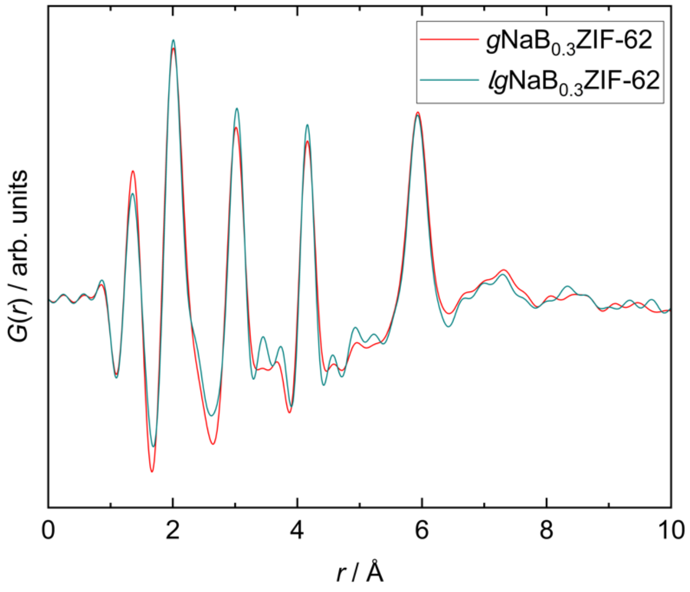


**Supplementary Figure 109.** X-ray pair distribution function in the form of *G*(*r*) for *g*NaB$_{0.3}$ZIF-62 and *lg*NaB$_{0.3}$ZIF-62 in an arbitrary scale. The extracted PDFs show a strong similarity between both materials as the water leaching primally removes Na(im) from the materials, which exhibits a low scattering cross-section.

## S16.7. Differential Scanning Calorimetry

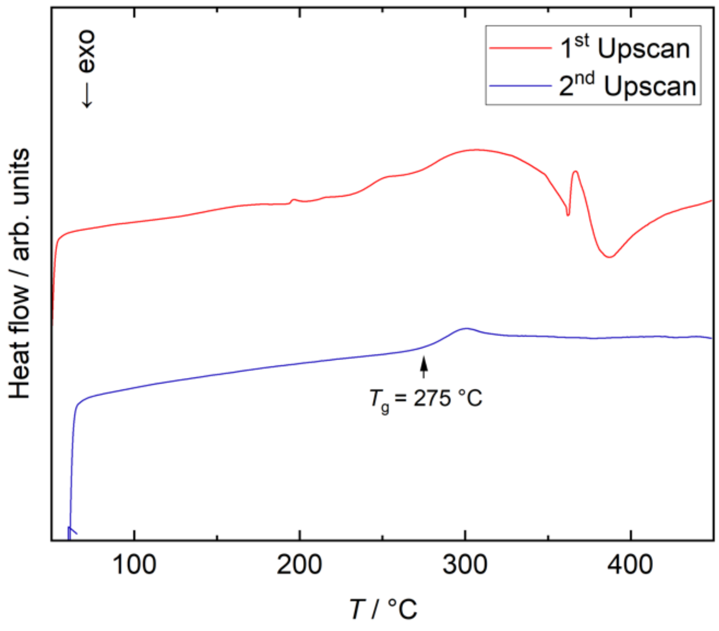


**Supplementary Figure 110**. Heat flow curves of consecutive thermal upscans of *ag*NaB$_{0.3}$ZIF-62. An isothermal segment of 30 mins was applied after the first upscan to improve homogenization. Heating and cooling were performed at a constant rate of ±10 K/min.

## S16.8. Quantification of $Na^+$ Content by $^{23}Na$ MAS NMR

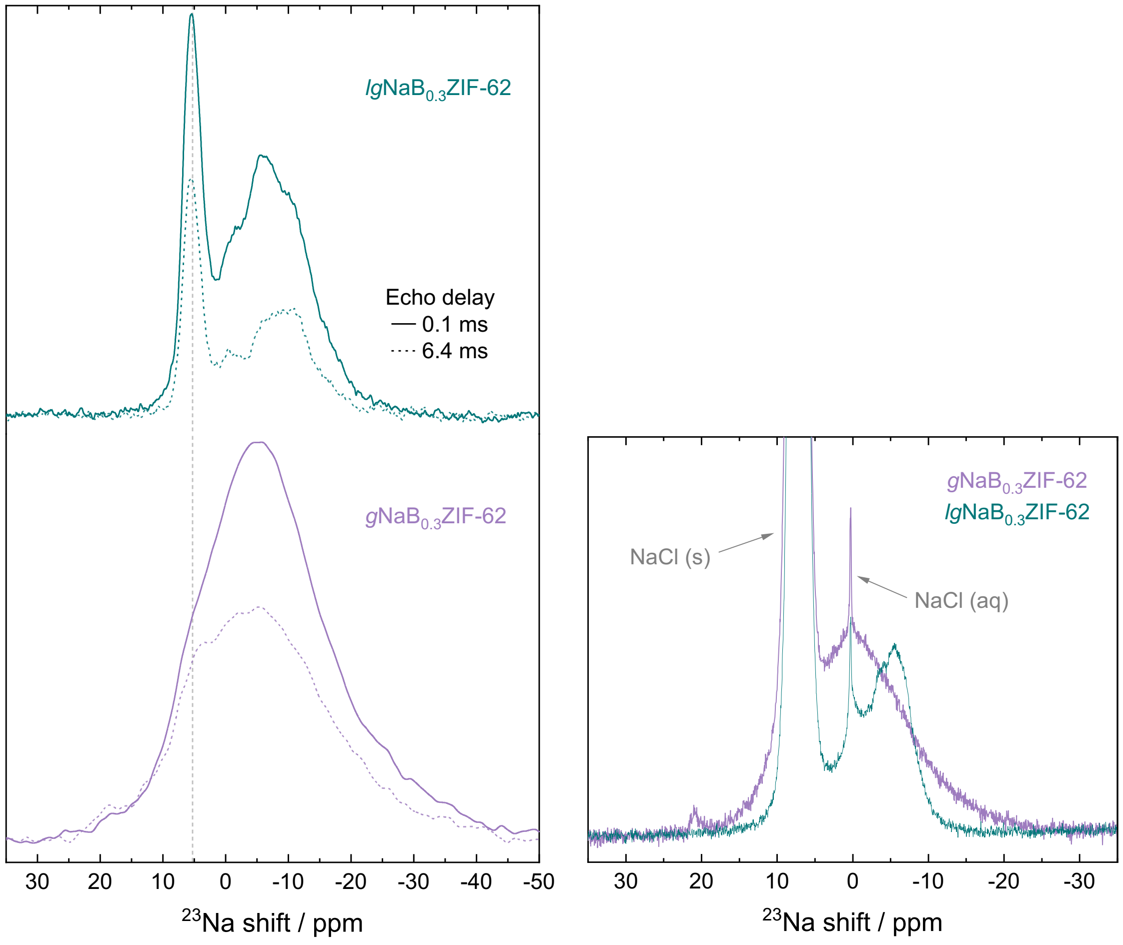


**Supplementary Figure 111**. $^{23}$Na MAS NMR of pristine *g*NaB$_{0.3}$ZIF-62 and leached *lg*NaB$_{0.3}$ZIF-62. Left shows the pristine and leached glass materials with and without a $T_2$ filter applied (described in Supplementary Figure 94). Spectra are recorded at 14.1 T with a 20 kHz MAS rate. Right shows the same materials with NaCl introduced, to enable quantification of $Na^+$ in the pristine and leached materials. Spectra are recorded at 23.5 T with a 20 kHz MAS rate.

In the absence of NaCl (left), it is apparent that leaching selectively removes $Na^+$ from the population giving rise to the broad component of the spectrum, leaving $Na^+$ in the populations contributing to underlying two narrow peaks. These narrow signals correspond to those discussed in the main text and observed at 6.8 and –6.3 ppm in $^{23}$Na MAS NMR spectra recorded at 20 T (**Figure 5 a-c**). This finding indicates that $Na^+$ in these environments are bound more strongly inside the metal-organic network than those contributing to the broad spectral component, consistent with our observation that these narrow signals correspond to sites of preferred but limited $Na^+$ occupancy.

By introducing a known quantity of NaCl to the sample during $^{23}$Na MAS NMR analysis (right) and using of a low tip angle pulse (0.5 μs) we determine the quantity of $Na^+$ in the pristine and leached glasses to be 0.81 and 0.54 mmol/g, respectively. The anticipated $Na^+$ concentration for *g*NaB$_{0.3}$ZIF-62, indirectly obtained from solution $^1$H NMR spectroscopy of 1.12 mmol/g is higher than the value obtained by spin-counting, which suggests a lower initial $Na^+$ content closer to $x$ = 0.2. This discrepancy, however, is supposedly caused by experimental imprecision and sample-to-sample variations and does not affect the relative change in $Na^+$ concentration for the samples before and after leaching. The results, therefore, indicate that approximately one-third of the $Na^+$ modifier has been removed by the leaching process.

## S16.9. Gas Sorption Experiments

Dual-site Langmuir isotherms were fitted to the desorption branches of the $CO_2$ sorption isotherms measured at 195 K.[21] This enables the extrapolation of the isotherms to higher relative pressures as accessible experimentally. In the dual-site Langmuir isotherm (Equation 7) the relationship between the uptake $N_{ads}$ and the pressure $p$ is expressed via two equilibrium constants $K_1$ and $K_2$ and the corresponding maximum adsorption capacities $N_1$ and $N_2$:

$$N_{\text{ads}} = \frac{K_1 \cdot p}{1 + \frac{K_1 \cdot p}{N_1}} + \frac{K_2 \cdot \text{p}}{1 + \frac{K_2 \cdot p}{N_2}} \tag{7}$$

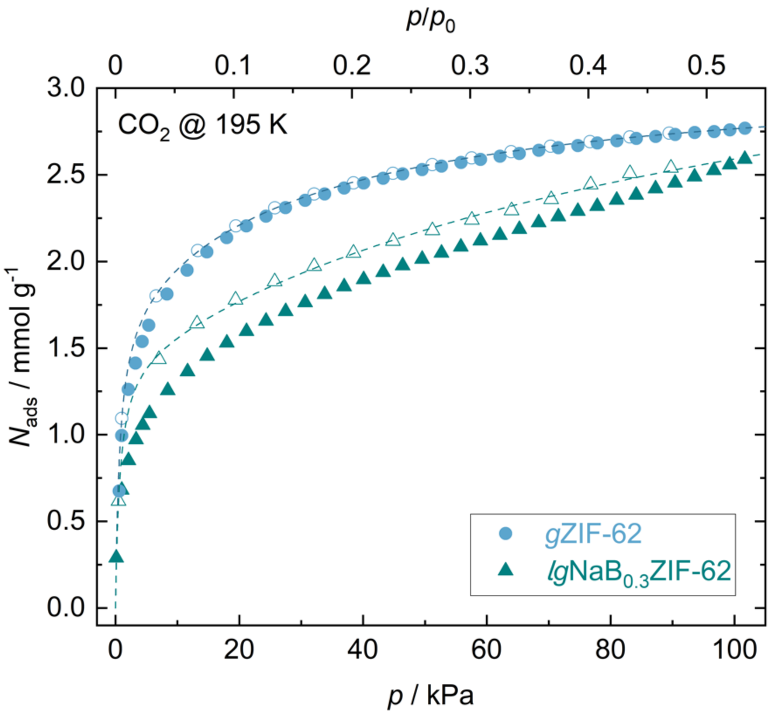


**Supplementary Figure 112.** $CO_2$ gas sorption isotherm of *g*ZIF-62, *g*$NaB_{0.3}$ZIF-62 and water-leached *lg*$NaB_{0.3}$ZIF-62 collected at a temperature of 195 K. The dashed lines correspond to a dual-site Langmuir model (7) fitted to the desorption branches of both gas sorption isotherms. The fitting parameters are shown in Supplementary Table 15.

**Supplementary Table 15.** Parameters obtained from the fitting of the dual-site Langmuir isotherms as shown in Equation 7 to the desorption branches of *g*ZIF-62 and *lg*NaB$_{0.3}$ZIF-62 in the gas sorption measurements performed with $CO_2$ at 195 K.

| Material | $K_1$ / mmol g$^{-1}$ kPa$^{-1}$ | $K_2$ / mmol g$^{-1}$ kPa$^{-1}$ | $N_1$ / mmol g$^{-1}$ | $N_2$ / mmol g$^{-1}$ |
|---|---|---|---|---|
| *g*ZIF-62 | 0.045 ± 0.002 | 2.64 ± 0.05 | 1.38 ± 0.01 | 1.72 ± 0.02 |
| *lg*NaB$_{0.3}$ZIF-62 | 0.021 ± 0.002 | 2.11 ± 0.13 | 2.47 ± 0.19 | 1.45 ± 0.04 |

At 195 K, $CO_2$ sorption is limited to a maximum $p/p_0$ of 0.53.[22–24] This means only micropores can be quantified under these conditions, while larger mesopores are not fully filled with the adsorbate. The virtual $CO_2$ capacity at saturation ($p/p_0$ = 1), $N_{sat,virt} = N_1 + N_2$, can be estimated from the saturation capacities $N_1$ and $N_2$ derived from the isotherm fits. $N_{sat,virt}$ is about 26% higher for *lg*NaB$_{0.3}$ZIF-62 ($N_{sat,virt}$ = 3.9 mmol g$^{-1}$) compared to *g*ZIF-62 ($N_{sat,virt}$ = 3.1 mmol g$^{-1}$). This reflects the substantially higher overall porosity of *lg*NaB$_{0.3}$ZIF-62 compared to *g*ZIF-62, which is also evident from *n*-butane sorption recorded at 273 K (see Figure 6 in the main manuscript). The experimental *n*-butane capacity close to saturation ($p/p_0$ = 0.95), $N_{sat,exp}$, similarly is 26% higher for *lg*NaB$_{0.3}$ZIF-62 ($N_{sat,exp}$ = 0.77 mmol g$^{-1}$) compared to *g*ZIF-62 ($N_{sat,exp}$ = 0.61 mmol g$^{-1}$)

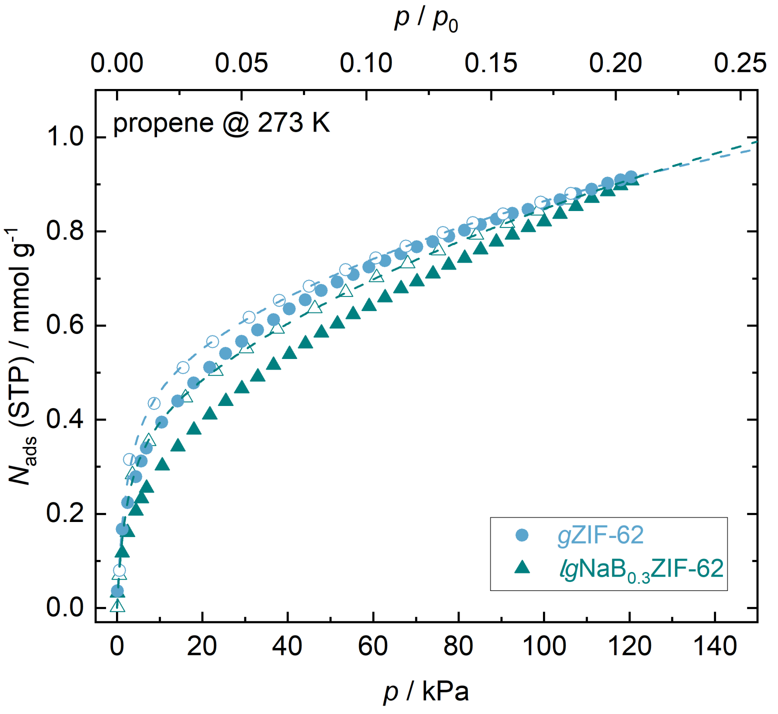


**Supplementary Figure 113.** Gas sorption isotherm of *g*ZIF-62 and *lg*NaB$_{0.3}$ZIF-62 collected with propene at a temperature of 273 K. The dashed lines correspond to a dual-site Langmuir model (7) fitted to the desorption branches of both gas sorption isotherms. The fitting parameters are shown in Supplementary Table 16.

**Supplementary Table 16.** Parameters obtained from the fitting of the dual-site Langmuir isotherms as shown in Equation 7 to the desorption branches of *g*ZIF-62 and *lg*NaB$_{0.3}$ZIF-62 in the gas sorption measurements performed with propene at 273 K.

| Material | $K_1$ / mmol g$^{-1}$ kPa$^{-1}$ | $K_2$ / mmol g$^{-1}$ kPa$^{-1}$ | $N_1$ / mmol g$^{-1}$ | $N_2$ / mmol g$^{-1}$ |
|---|---|---|---|---|
| *lg*NaB$_{0.3}$ZIF-62 | 0.006 ± 0.001 | 0.171 ± 0.013 | 1.578 ± 0.283 | 0.415 ± 0.020 |
| *g*ZIF-62 | 0.006 ± 0.001 | 0.211 ± 0.018 | 1.127 ± 0.247 | 0.502 ± 0.028 |

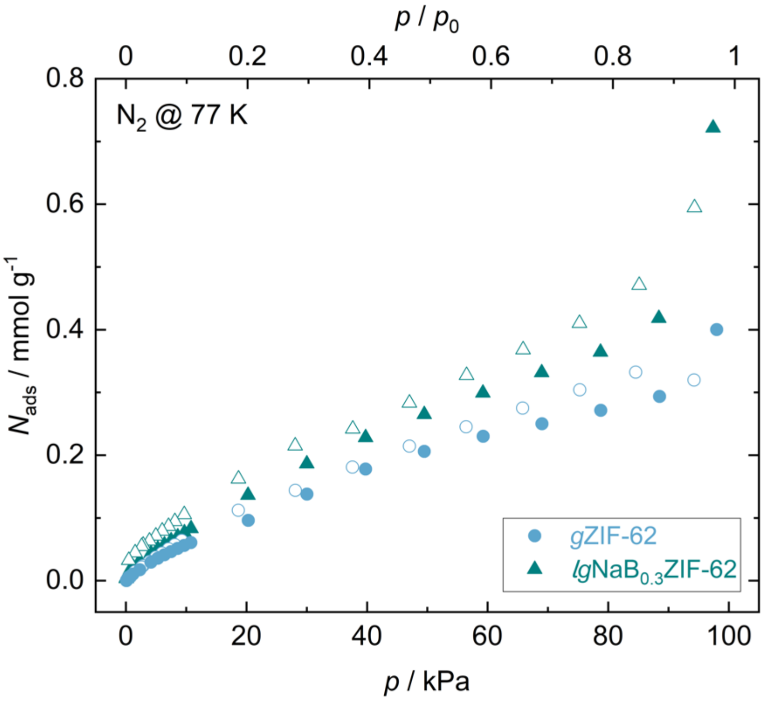


**Supplementary Figure 114.** $N_2$ gas sorption isotherm of *g*ZIF-62 and *lg*NaB$_{0.3}$ZIF-62 after the water leaching treatment collected at a temperature of 77K.

**Supplementary Table 17.** Maximum gas uptake $N_{ads,max}$ for *g*ZIF-62 and *g*NaB$_{0.3}$ZIF-62 prior to and after the water leaching (*lg*NaB$_{0.3}$ZIF-62) for different gases and at different temperatures. The given values correspond to the maximum uptake of $CO_2$ (at $p/p_0 \approx 0.53$), $N_2$ (at $p/p_0 \approx 0.90$), *n*-butane (at $p/p_0 \approx 0.95$) and propene (at $p/p_0 \approx 0.21$) in the corresponding gas sorption isotherm (see Supplementary Figure 112–114 and Figure 6 in the main manuscript).

| | $N_{ads,max}$ / mmol g$^{-1}$ | | | |
|---|---|---|---|---|
| Material | $CO_2$ (195 K) | $N_2$ (77 K) | $C_4H_{10}$ (273 K) | $C_3H_6$ (273 K) |
| *g*ZIF-62 | 2.77 | 0.29 | 0.61 | 0.92 |
| *lg*NaB$_{0.3}$ZIF-62 | 2.59 | 0.42 | 0.77 | 0.92 |
| *g*NaB$_{0.3}$ZIF-62 | 2.27 | – | – | – |

For determination of the BET area, the Brunauer-Emmett-Teller equation (8) was fitted to the adsorption branch of the gas sorption isotherms collected with $CO_2$ at 195 K in a pressure range from 0.05 – 0.1 ($p/p_0$) which corresponds to the range of micropore filling:[25]

$$\frac{1}{\mathrm{V}\,[(p/p_0)-1]} = \frac{c-1}{V_m\,c}\left(\frac{p}{p_0}\right) + \frac{1}{\mathrm{V_m}\,c} \qquad (8)$$

The parameters in Equation 8 correspond to the gas uptake ($V$), the gas volume adsorbed on the monolayer of the micropores ($V_m$) and the BET constant $c$. The BET area ($S_{BET}$) is calculated from $V_m$, the Avogadro constant $N_A$, the molar volume $V_{mol}$ and the molecular cross-sectional area $A_m$ (21 Å$^2$ for $CO_2$) according to (9):

$$S_{BET} = V_m\,\frac{N_A\,\cdot\,A_\mathrm{m}}{V_{mol}} \qquad (9)$$

The results are summarized in Supplementary Table 18. We would like to emphasize that the BET theory was originally developed for non-porous extended surfaces. Its application to ultramicroporous materials (such as those investigated here) is inherently problematic due to the breakdown of key assumptions, including the presence of a distinguishable multilayer regime and the absence of pore-filling effects. Consequently, the BET areas derived for the MOF glasses should be interpreted with caution and regarded as apparent surface areas rather than as meaningful representations of the physical surface accessible to adsorbates.

**Supplementary Table 18.** Results from the fit of the BET Equations (8) and (9) to the adsorption branch of the gas sorption isotherms of *g*ZIF-62, *g*NaB$_{0.3}$ZIF-62 and *lg*NaB$_{0.3}$ZIF-62 collected with $CO_2$ at 195 K.

| Material | $S_{BET}$ / m$^2$ g$^{-1}$ | $c$ | $V_m$ / cm$^3$ g$^{-1}$ |
|---|---|---|---|
| *g*ZIF-62 | 238.3 | 81.8 | 0.083 |
| *g*NaB$_{0.3}$ZIF-62 | 143.1 | 33.2 | 0.050 |
| *lg*NaB$_{0.3}$ZIF-62 | 171.3 | 63.1 | 0.060 |

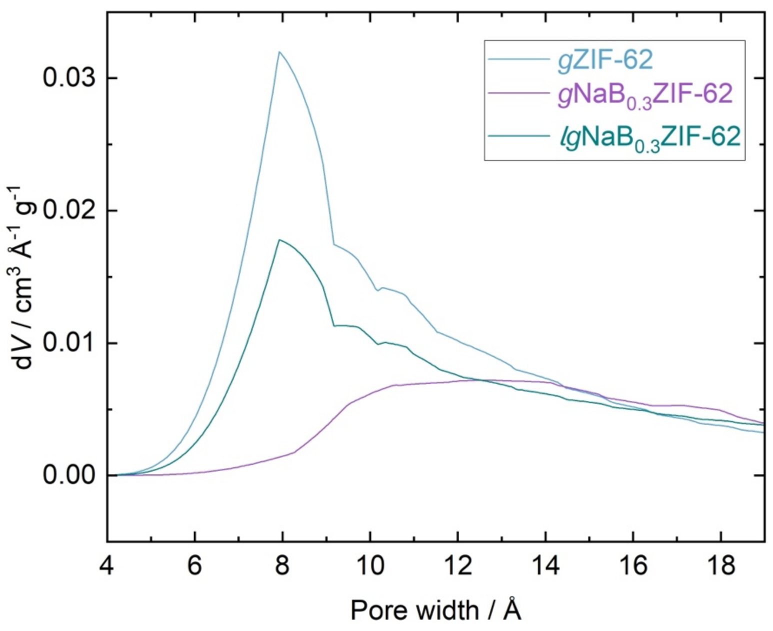


**Supplementary Figure 115.** Pore size distribution depicting the differential pore volume (d*V*) as a function of the pore width. The distribution is the result of calculations using the Horvath-Kawazoe (HK) model[26] based on $CO_2$ gas adsorption isotherms collected at 195 K. The plot demonstrates the loss of micropores upon modifier incorporation and the partial recovery of the micropore volume after water extraction.

# S17. Atmospheric Stability

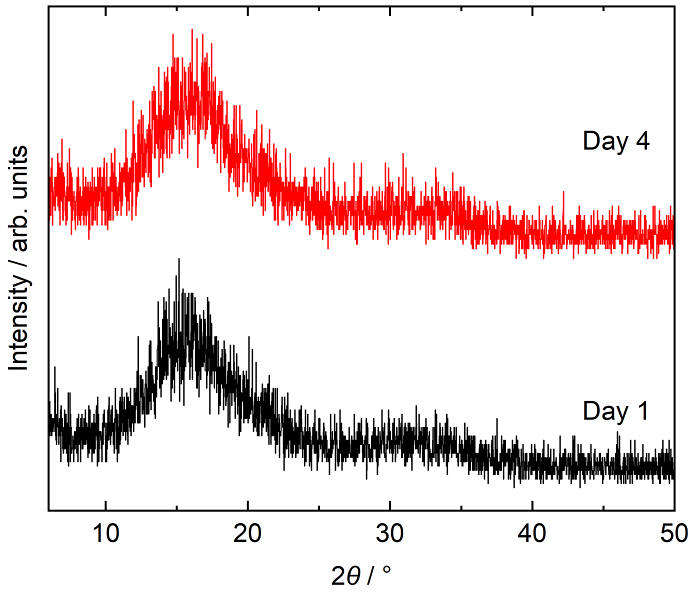


**Supplementary Figure 116**. PXRD pattern of *g*$NaB_{0.3}ZIF$-62 before and after exposure to the atmosphere for four days. Diffraction patterns were recorded with $CuK_\alpha$ radiation. The scattered intensity indicates no change in the amorphous state of the material due to the exposure to atmospheric water.

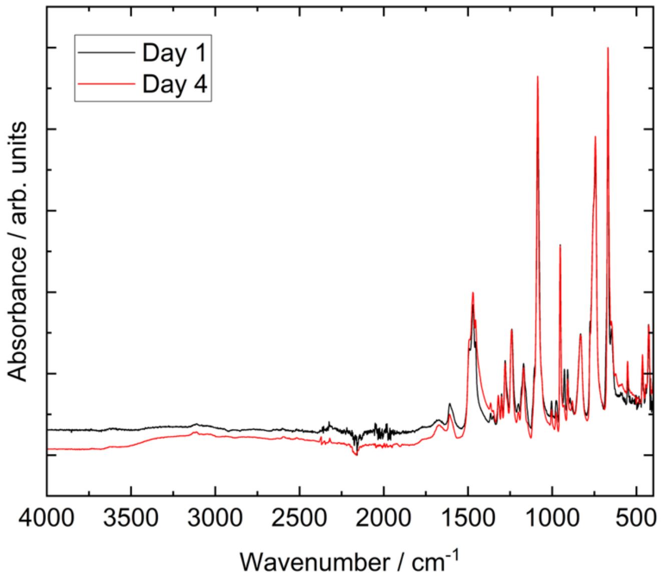


**Supplementary Figure 117.** Mid-infrared spectra of *g*$NaB_{0.3}ZIF$-62 before and after exposure to the atmosphere for four days. The increased intensity in the region between 2500 $cm^{-1}$ and 3500 $cm^{-1}$ is assigned to the OH stretch vibration of adsorbed atmospheric water. The sample mass increased by 3% over the course of 4 days due to the adsorption of water.

## S18. Lithium Benzimidazolate Modification

In an effort to further expand the chemical range of alkali-modified ZIF-62 glasses, Li(bim), LiB, was investigated as an alternative modifier.[7] Melt-quenching of physical mixtures $LiB_x$|ZIF-62 (with $x$ = 0.3, 0.6, 1.1) in sealed DSC pans from 425 °C produced clear, decomposition-free glasses with evidence of macroscopic flow, as confirmed by solution $^1H$ NMR measurements of acid-digested samples (Supplementary Figure 123–126) and microscopy images of the vitrified materials (Supplementary Figure 119). PXRD patterns of the resulting *g*$LiB_x$ZIF-62 glasses verified their amorphous nature (Extended Data Figure 1a). Additional PXRD measurements of the physical mixtures after the application of heating programs with maximum temperatures below 425 °C, together with VT-PXRD data, indicate a tendency for intermediate crystallisation of ZIF-zni or ZIF-7-III similar to the phase behavior observed for $NaB_x$|ZIF-62 materials (Supplementary Figure 121, 122, 130).

As in the case of *g*$NaB_x$ZIF-62 glasses, a modifier-dependent decrease in $T_g$ was observed as a consequence of glass network depolymerisation upon Li(bim) incorporation (Extended Data Figure 1b,c). To compare the relative drop in $T_g$ for various binary glass systems, the reduced glass transition temperature $T_R$ = $T_g$(modified glass) / $T_g$(glass former) is used. While $T_R$ follows a similar trend for Li(bim)- and Na(bim)-modified ZIF-62 as a function of alkali content, sodium silicate glasses were found to follow a much steeper decrease upon $Na_2O$ addition (Extended Data Figure 1d).[27] The shallower decrease in $T_R$ for the alkali-benzimidazolate-modified ZIF glasses, compared to $Na_2O$-modified silicate glasses, arises from the smaller charge contrast introduced into the network ($Na^+/Zn^{2+}$) relative to that in silicates ($Na^+/Si^{4+}$). The slightly higher $T_g$ of *g*$LiB_x$ZIF-62 compared to *g*$NaB_x$ZIF-62 at similar $x$ is rationalised by the smaller ionic radius of $Li^+$ compared to $Na^+$ and resulting shorter and stronger Li–N bonds,[7] providing a higher barrier for bond dissociation events upon approaching $T_g$.

Analogous to Na(bim)-modified glasses, the incorporation of Li(bim) is accompanied by a fusion and broadening of vibrational bands in the IR spectra, reflecting modifier-induced structural disorder, and by broadening of the PDF signal associated with a loss of Zn···Zn pair correlation density (Supplementary Figure 118, 131). The observed similarities in the thermal and structural analysis of Li(bim)- and Na(bim)-modified ZIF-

62 glasses underline the robustness and general applicability of the glass-modification concept to the field of MOF glasses and highlight how material properties can be controlled systematically by the choice of the modifiers‘ alkali cation.

## S18.1. Experimental Details

Lithium benzimidazolate (Li(bim)) was synthesized according to an earlier publication.[7] Benzimidazole (Hbim, 1.5 g, 12.7 mmol) was dissolved in 55 mL of anhydrous tetrahydrofuran (THF) in a 250 mL Schlenk flask under a continuous flow of argon. The solution was cooled to approximately −70 °C, and *n*-butyllithium (4.5 mL, 11.5 mmol, 2.5 M in *n*-hexane) was added dropwise via syringe. After addition, the cooling bath was removed, and the mixture was allowed to warm to ambient temperature (~25 °C) while stirring overnight. The following day, the supernatant liquid was decanted from the precipitate, and the solid was washed once with dry *n*-hexane (40 mL). The material was dried under dynamic vacuum at 100 °C for 2 h, followed by additional heating at 250 °C for 3 h under vacuum to remove residual Hbim. The resulting Li(bim) was obtained as a white powder (1.15 g, 81%) and stored under argon in a glovebox.

Further steps in the preparation of the physical mixtures and modified glasses via melt-quenching are analogous to the preparation of the Na(bim)-modified ZIF-62 glasses (see Methods Section in the main manuscript) with changes in the mass ratio of the starting compounds (Supplementary Table 19) and applied temperature program. The *g*LiB$_x$|ZIF-62 materials were prepared in a DSC crucible (10 mg scale) with a maximum temperature of 425 °C during the isothermal segment (30 min) in the corresponding temperature program. Heating and cooling were performed at a rate of ±10 °C/min. Manual handling of the materials was performed in a glovebox under an inert atmosphere to prevent the hygroscopic materials from reacting with atmospheric water.

**Supplementary Table 19.** Composition of LiB$_x$|ZIF-62 materials. The physical mixtures comprise various amounts of glass former ZIF-62 and glass modifier Li(bim).

| $x$ | $m_{ZIF\text{-}62}$ / mg | $n_{ZIF\text{-}62}$ / mmol | $m_{Li(bim)}$ / mg | $n_{Li(bim)}$ / mmol |
|---|---|---|---|---|
| 0 | 250.0 | 1.19 | 0.0 | 0.00 |
| 0.3 | 212.4 | 1.00 | 37.6 | 0.30 |
| 0.5 | 193.0 | 0.92 | 57.0 | 0.46 |
| 1.0 | 157.2 | 0.75 | 92.8 | 0.75 |

## S18.2. Infrared Spectroscopy

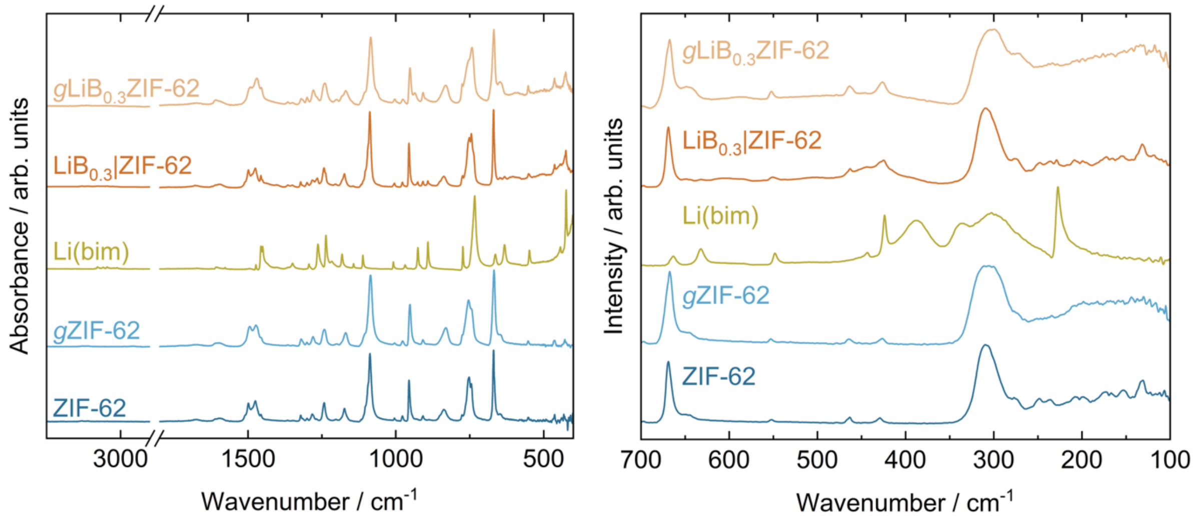


**Supplementary Figure 118.** Mid-infrared spectra (left) and far-infrared spectra (right) of ZIF-62, the corresponding glass *g*ZIF-62, the modifier Li(bim), the physical mixture of modifier and glass former LiB$_{0.3}$|ZIF-62 and the corresponding glass of the physical mixture *g*LiB$_{0.3}$ZIF-62

## S18.3. Microscopy Images

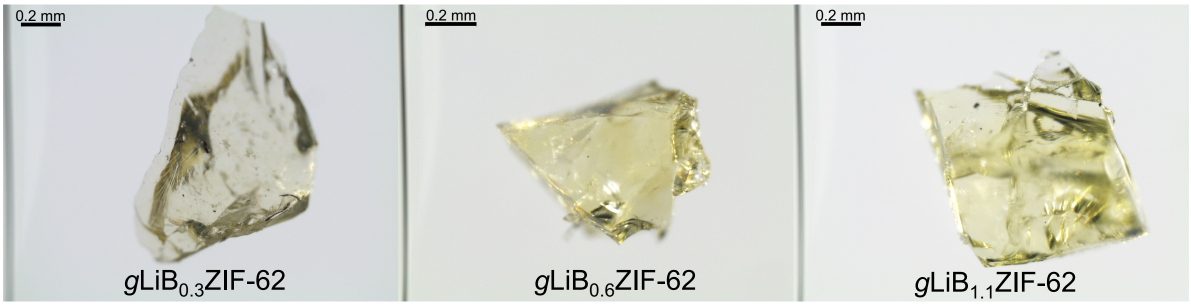


**Supplementary Figure 119.** Optical microscopy images of shards of *g*LiB$_x$ZIF-62 materials with different Li(bim) contents $x$. The glasses were prepared in a 10 mg scale in a DSC apparatus.

## S18.4. Powder X-ray Diffraction

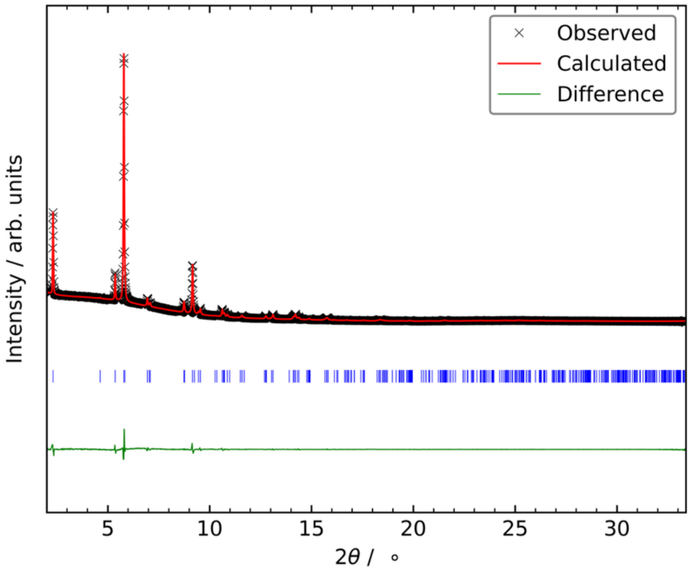


**Supplementary Figure 120.** Structureless profile fit (Pawley method[6]) performed on the PXRD measurement of Li(bim) recorded at room temperature with a wavelength of 0.4603 Å at DELTA. Blue tick marks indicate the position of allowed Bragg peaks. Refined unit cell parameters (space group *Cmma*) are $a$ = 5.7631(14), $b$ = 9.8426(7), $c$ = 11.4028(7), $\alpha = \beta = \gamma$ =90°, $V$ = Å$^3$ , $R_{wp}$ = 3.86%, $R_{exp}$ = 18.08%, $\chi$ = 0.2.

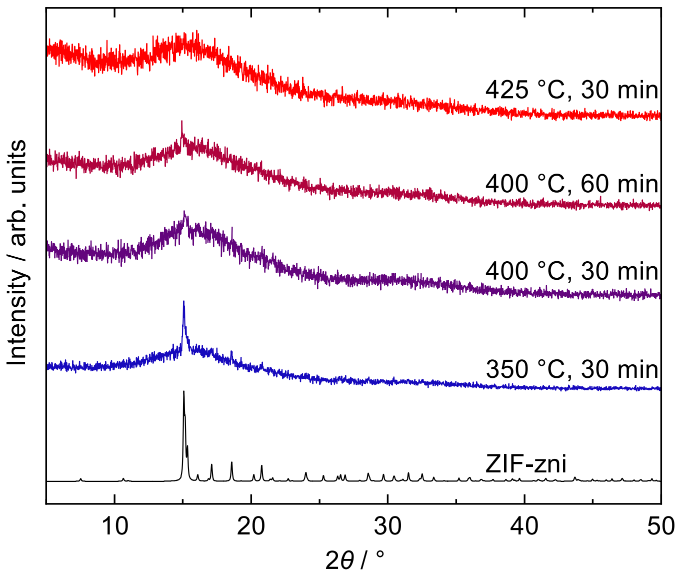


**Supplementary Figure 121.** PXRD patterns of $LiB_{0.3}$|ZIF-62 after different temperature treatments consisting of a heating segment from room temperature up to a maximum temperature, followed by an isothermal segment of several minutes and subsequent cooling back to room temperature. The heating and cooling were performed at a constant rate of 10 °C $min^{-1}$. The maximum temperature and time of the isothermal segment are given next to the corresponding PXRD patterns of the materials after the temperature treatment. The PXRD patterns were recorded with $CuK_{\alpha}$ radiation under air. The black pattern (bottom) corresponds to a simulated diffraction pattern of ZIF-zni (CCDC: IMIDZB).

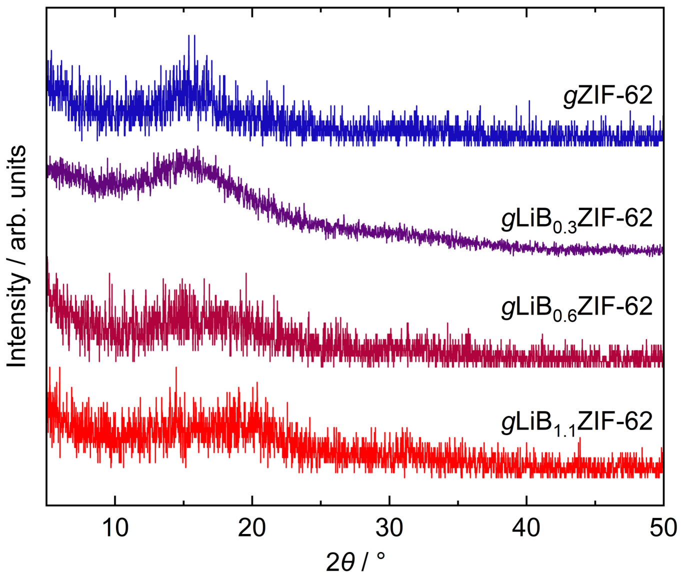


**Supplementary Figure 122.** PXRD patterns of *g*$LiB_x$ZIF-62 materials after the melt-quenching procedure performed in a DSC apparatus (~10 mg scale) consisting of a heating segment from room temperature up to 425 °C, followed by an isothermal segment of 30 mins and subsequent cooling back to room temperature. The heating and cooling were performed at a constant rate of 10 °C $min^{-1}$. Diffraction patterns were recorded with $CuK_{\alpha}$ radiation under air.

## S18.5. Solution $^1$H NMR Spectroscopy

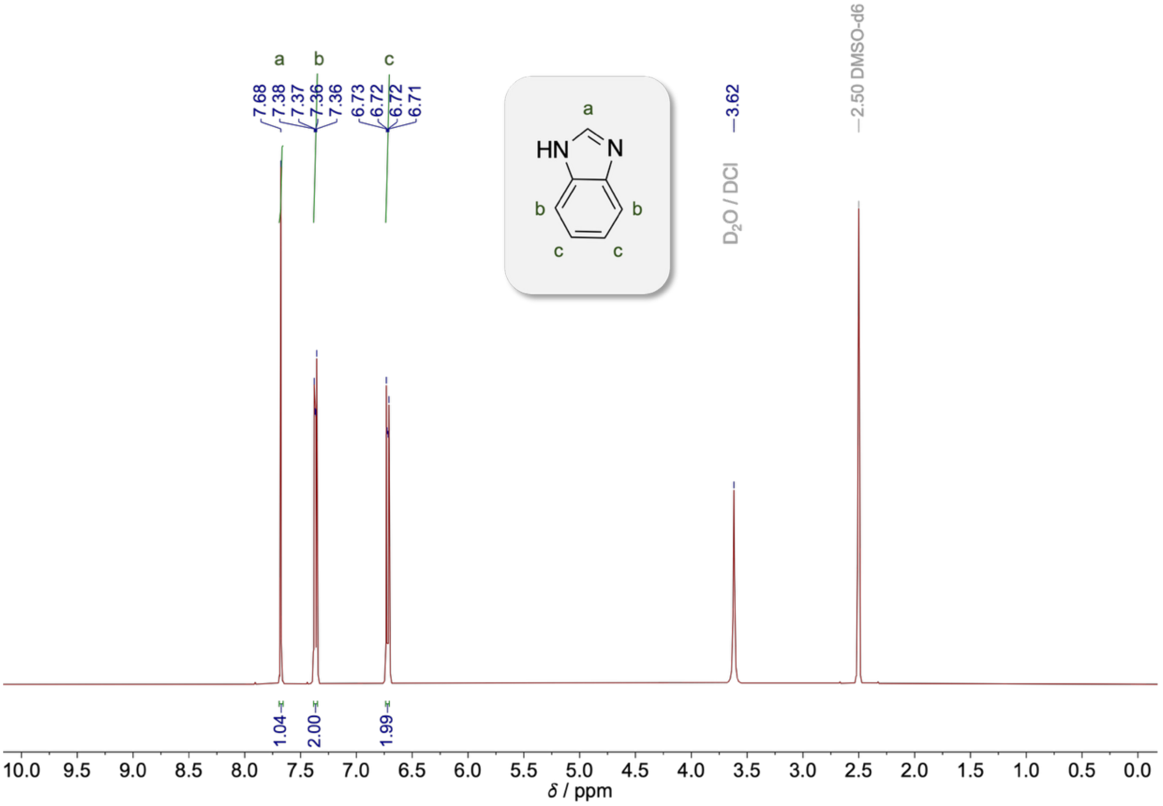


**Supplementary Figure 123.** $^1$H NMR spectrum of Li(bim) dissolved in DMSO-$d_6$ and DCl/$D_2O$ (35 wt%, one drop, <0.1 mL).

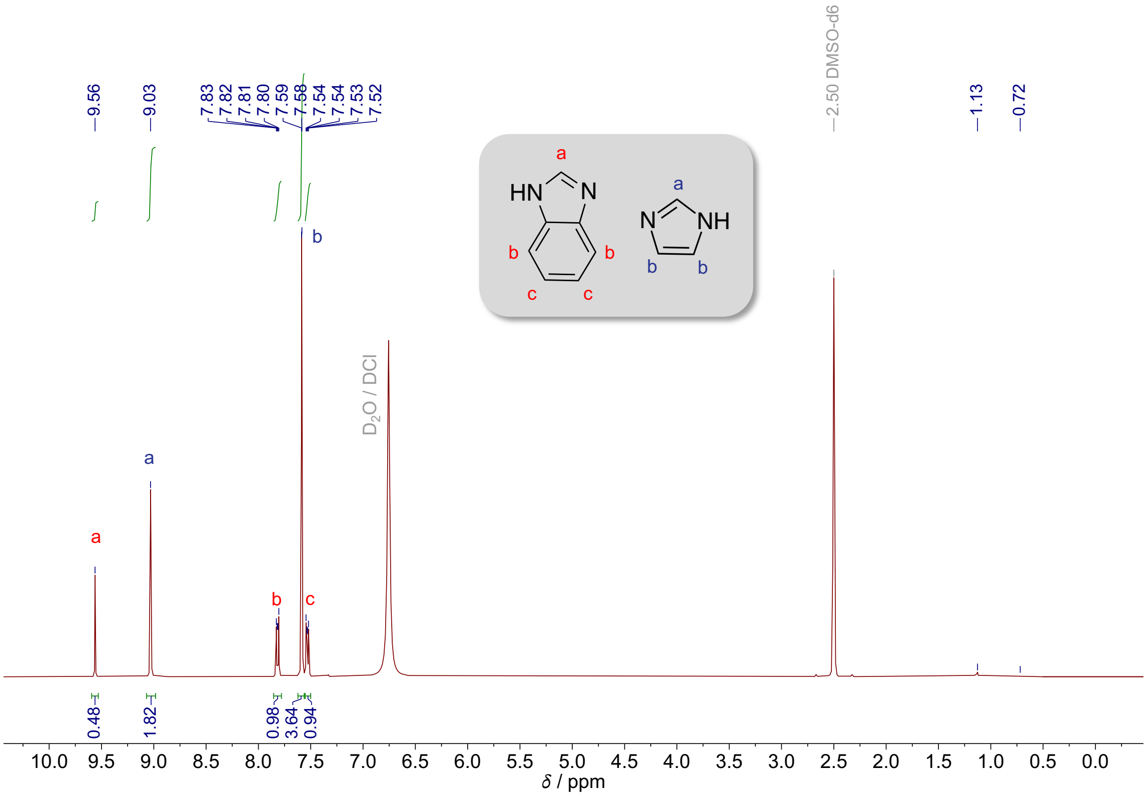


**Supplementary Figure 124.** $^{1}$H NMR spectrum of *g*LiB$_{0.3}$ZIF-62 (prepared in ~10 mg scale) dissolved in DMSO-*d*$_6$ and DCl/D$_2$O (35 wt%, one drop, <0.1 mL). Signals at 1.13 ppm are assigned to residues of *n*-hexane, which was added during the grinding of the obtained glass monolith.

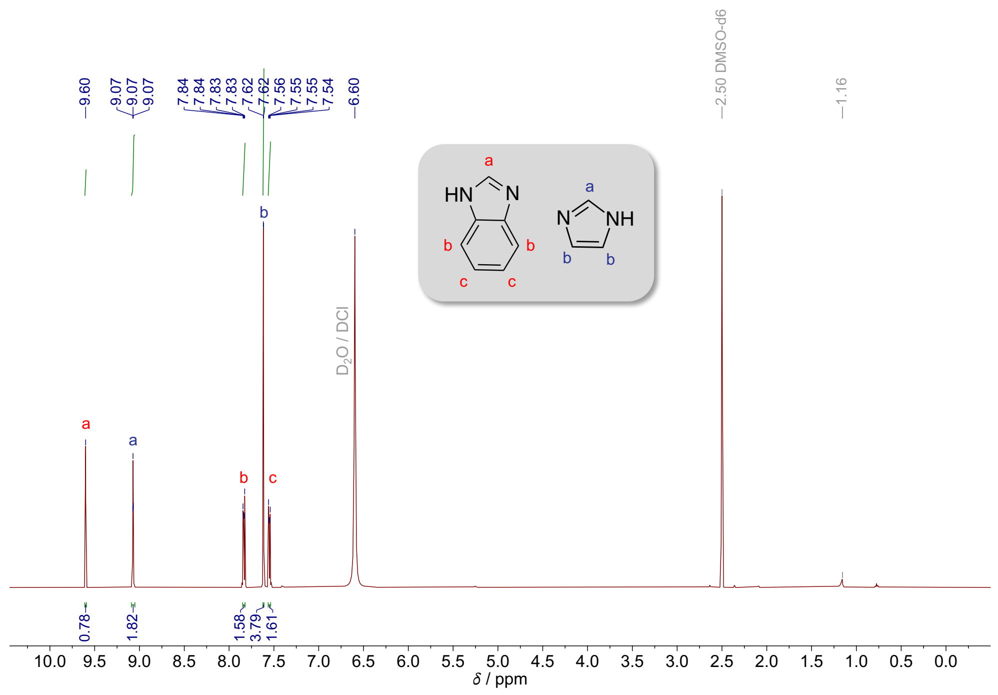

**Supplementary Figure 125.** $^{1}$H NMR spectrum of *g*LiB$_{0.6}$ZIF-62 (prepared in ~10 mg scale) dissolved in DMSO-$d_6$ and DCl/$D_2O$ (35 wt%, one drop, <0.1 mL). Signals at 1.13 ppm are assigned to residues of *n*-hexane, which was added during the grinding of the obtained glass monolith.

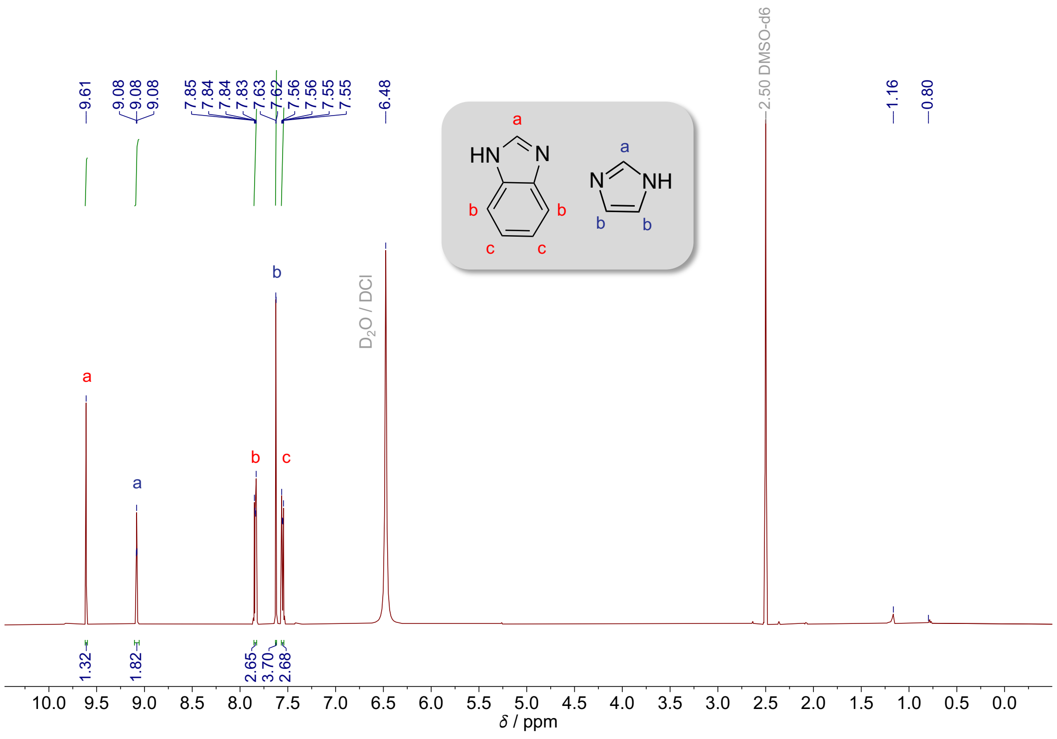

**Supplementary Figure 126.** $^{1}$H NMR spectrum of *g*LiB$_{1.1}$ZIF-62 (prepared in ~10 mg scale) dissolved in DMSO-$d_6$ and DCl/$D_2O$ (35 wt%, one drop, <0.1 mL). Signals at 1.13 ppm are assigned to residues of *n*-hexane, which was added during the grinding of the obtained glass monolith.

**Supplementary Table 20.** Name and chemical composition of prepared Li(bim)-modified ZIF-62 glasses *g*LiB$_x$ZIF-62 for different Li(bim) contents *x* and ZIF-62. The chemical composition was calculated according to the bim/im ratio obtained from solution $^1$H NMR spectroscopy measurements of the glass samples digested in DMSO-$d_6$ and DCl/$D_2O$ (35 wt%, one drop, <0.1 mL). The thus calculated Li(bim) content $x$($^1$H NMR) does not necessarily coincide with the *x* used in the nomenclature (*g*LiB$_x$ZIF-62) due to rounding. It is assumed that the *x* values in the nomenclature are in accordance with the solution $^1$H NMR results with regard to the error margin of the NMR measurement. The chemical composition of the corresponding physical mixtures LiB$_x$|ZIF-62 is expected to be identical to those of the respective glasses. Deviations between the intended chemical composition and the composition found in $^1$H NMR spectroscopy may be attributed to weighing errors during the preparation of the physical mixtures and measurement inaccuracies of $^1$H NMR spectroscopy.

| Approach | Name | bim$^-$ content | im$^-$ content | Chemical composition | $x$($^1$H NMR) |
|---|---|---|---|---|---|
| 10 mg scale | *g*LiB$_{0.3}$ZIF-62 | 0.48 | 1.82 | $Li_{0.30}Zn(im)_{1.82}(bim)_{0.48}$ | 0.30 |
| | *g*LiB$_{0.6}$ZIF-62 | 0.78 | | $Li_{0.60}Zn(im)_{1.82}(bim)_{0.78}$ | 0.60 |
| | *g*LiB$_{1.1}$ZIF-62 | 1.32 | | $Li_{1.14}Zn(im)_{1.82}(bim)_{1.32}$ | 1.14 |

## S18.6. Differential Scanning Calorimetry

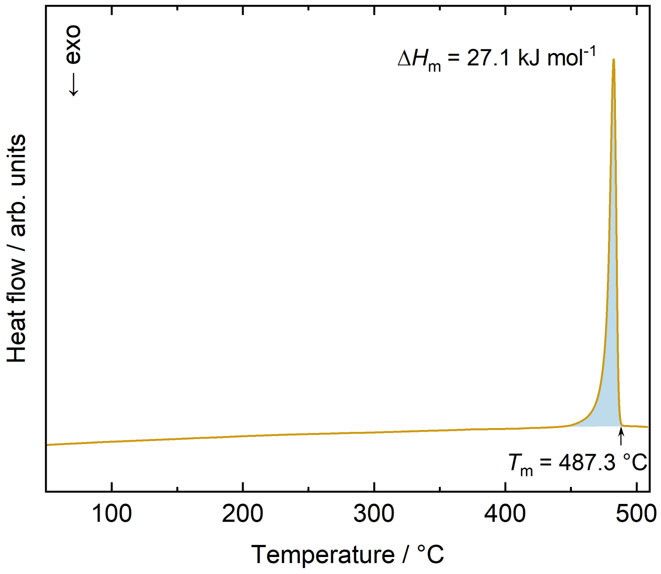


**Supplementary Figure 127.** Heat flow curves for the heating of Li(bim) at a constant rate of ±10 °C min$^{-1}$. The enthalpy of melting $\Delta H_m$ was calculated based on the mean molar mass of the compound. The arrow indicates the temperature of melting $T_m$,

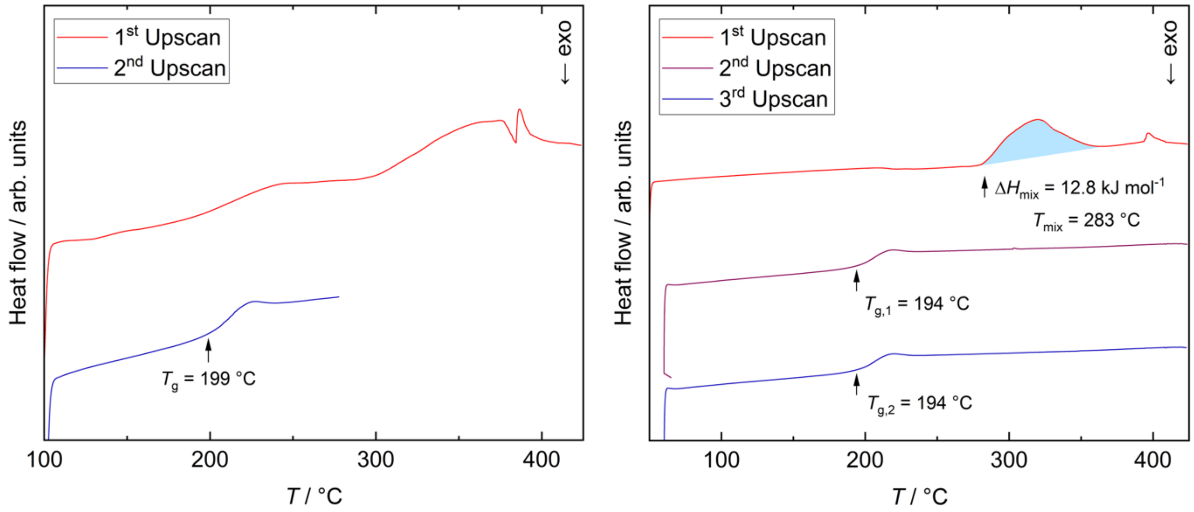


**Supplementary Figure 128.** Heat flow curves of consecutive thermal upscans of $LiB_x$|ZIF-62 for $x$ = 0.3 (left) and $x$ = 0.6 (right) to a maximum temperature of 425 °C in the first upscan. An isothermal segment of 30 mins was applied after the first upscan to improve homogenization. Heating and cooling were performed at a constant rate of ±10 °C min$^{-1}$. Enthalpies $\Delta H$ were calculated based on the mean molar mass of the corresponding physical mixture. Sharp features in the first upscan beyond 350 °C are considered artefacts resulting from crucible deformation under the autogenous pressure build-up. The arrows indicate the temperature of the glass transition $T_g$, and the temperature of the intermixing process $T_{mix}$ during the reaction of ZIF-62 with Li(bim).

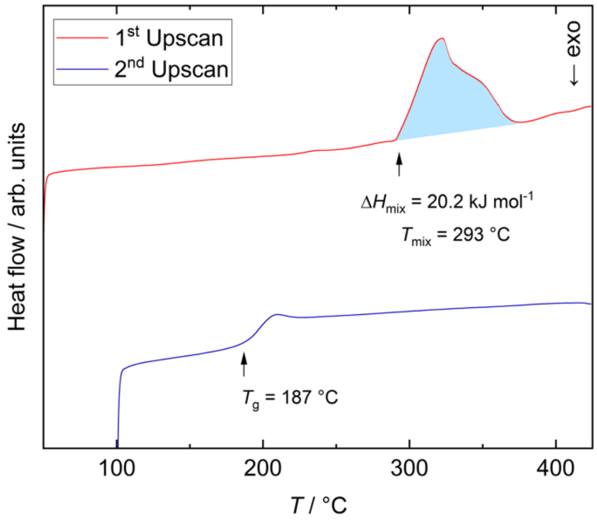


**Supplementary Figure 129.** Heat flow curves of consecutive thermal upscans of $LiB_x$|ZIF-62 for $x$ = 1.1 to a maximum temperature of 425 °C in the first upscan. An isothermal segment of 30 mins was applied after the first upscan to improve homogenization. Heating and cooling were performed at a constant rate of ±10 °C min$^{-1}$. Enthalpies $\Delta H$ were calculated based on the mean molar mass of the corresponding physical mixture. Sharp features in the first upscan beyond 350 °C are considered artefacts resulting from crucible deformation under the autogenous pressure build-up. The arrows indicate the temperature of the glass transition $T_g$, and the temperature of the intermixing process $T_{mix}$ during the reaction of ZIF-62 with Li(bim).

## S18.7. Variable Temperature Powder X-ray Diffraction

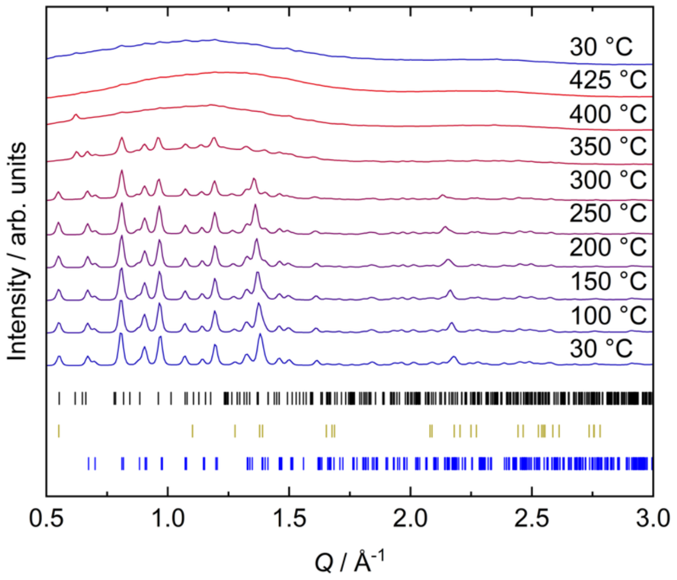


**Supplementary Figure 130.** VT-PXRD patterns of a heating-cooling-cycle of $LiB_{1.1}$|ZIF-62 recorded with λ = 0.1616 Å at DLS starting with the heating from 30 °C (blue, bottom) up to 425 °C (red) and subsequent cooling back to 30 °C (blue, top). The tick marks correspond to the allowed Bragg peak positions of ZIF-62 (blue) as well as Li(bim) (light green) and ZIF-7-III (black) (CCDC: GIZJOP, KOLYAM). ZIF-7-III crystallises at 350 °C and dissolves in the ZIF melt above 400 °C.

## S18.8. X-ray Pair Distribution Function Analysis

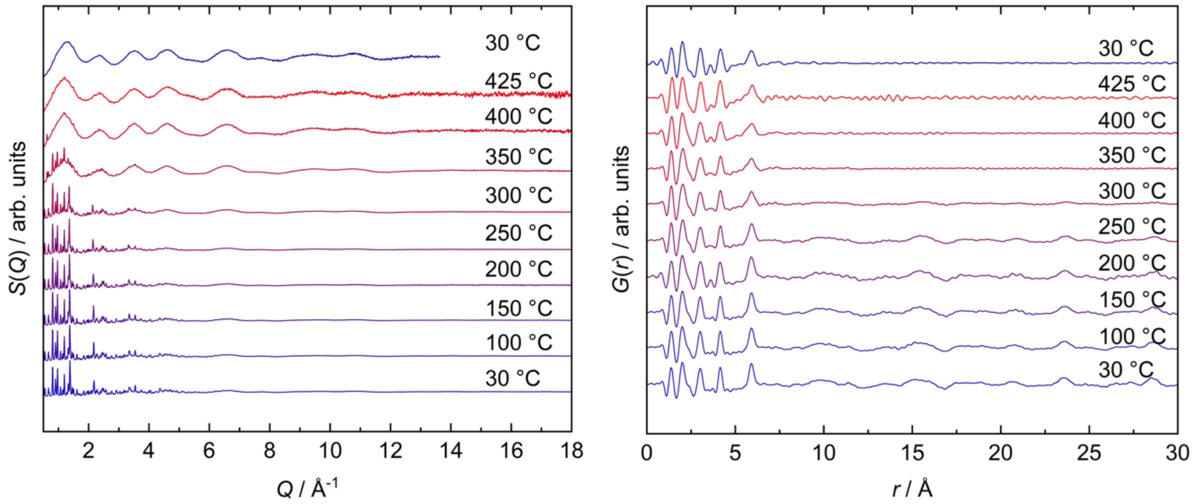


**Supplementary Figure 131.** Structure function $S(Q)$ extracted from X-ray total scattering data for the physical mixtures $LiB_{1.1}$|ZIF-62 (left) with increasing temperature and after temperature-quenching (top-pattern in blue) together with the corresponding PDFs in the form of $G(r)$. Data collection was performed at DLS with a wavelength of 0.1616 Å. The overall low intensity and high noise level in the pattern of $LiB_{1.1}$|ZIF-62 (left) collected at 425 °C is the result of the liquid material moving partially out of the beam path.

## S19. Bibliography


1. Kresse, G. & Furthmüller, J. Efficiency of ab-initio total energy calculations for metals and semiconductors using a plane-wave basis set. *Comput. Mater. Sci.* **6**, 15–50 (1996).
2. Kresse, G. & Furthmüller, J. Efficient iterative schemes for ab initio total-energy calculations using a plane-wave basis set. *Phys. Rev. B* **54**, 11169–11186 (1996).
3. Baroni, S., Gironcoli, S. de, Corso, A. D. & Giannozzi, P. Phonons and related crystal properties from density-functional perturbation theory. *Rev. Mod. Phys.* **73**, 515–562 (2001).
4. Giannozzi, P. & Baroni, S. Vibrational and dielectric properties of C60 from density-functional perturbation theory. *J. Chem. Phys.* **100**, 8537–8539 (1994).
5. Esfarjani, K., Hashi, Y., Onoe, J., Takeuchi, K. & Kawazoe, Y. Vibrational modes and IR analysis of neutral photopolymerized C60 dimers. *Phys. Rev. B* **57**, 223–229 (1998).
6. Pawley, G. S. Unit-cell refinement from powder diffraction scans. *J. Appl. Cryst.* **14**, 357–361 (1981).
7. Kolodzeiski, P. *et al.* Lithium and Sodium Benzimidazolate Coordination Networks: Syntheses, Structures, and Thermal Properties. *Cryst. Growth Des.* **24**, 7278–7286 (2024).
8. Zheng, Q., Zheng, J., Solvang, M., Yue, Y. & Mauro, J. C. Determining the liquidus viscosity of glass-forming liquids through differential scanning calorimetry. *J. Am. Ceram. Soc.* **103**, 6070–6074 (2020).
9. Juhás, P., Davis, T., Farrow, C. L. & Billinge, S. J. L. PDFgetX3: a rapid and highly automatable program for processing powder diffraction data into total scattering pair distribution functions. *J. Appl. Cryst.* **46**, 560–566 (2013).
10. Gallington, L. C., Wilke, S. K., Kohara, S. & Benmore, C. J. Review of Current Software for Analyzing Total X-ray Scattering Data from Liquids. *Quantum Beam Sci.* **7**, 20 (2023).
11. Crupi, C., Carini, G., Ruello, G. & D'Angelo, G. Intermediate range order in alkaline borate glasses. *Philos. Mag.* **96**, 788–799 (2016).

12. Liu, H. *et al.* A medium range order structural connection to the configurational heat capacity of borate–silicate mixed glasses. *Phys. Chem. Chem. Phys.* **18**, 10887–10895 (2016).
13. Frentzel-Beyme, L., Kolodzeiski, P., Weiß, J.-B., Schneemann, A. & Henke, S. Quantification of gas-accessible microporosity in metal-organic framework glasses. *Nat. Commun.* **13**, 7750 (2022).
14. Moneeb, T. M. S. The First Sharp Diffraction Peak in the Total Structure Function of Amorphous Chalcogenide Glasses: Anomalous Characteristics and Controversial Views. *New J. Glass Ceram.* **06**, 37–46 (2016).
15. Elliott, S. R. Extended-range order, interstitial voids and the first sharp diffraction peak of network glasses. *J. Non-Cryst. Solids* **182**, 40–48 (1995).
16. Misawa, M., Price, D. L. & Suzuki, K. The short-range structure of alkali disilicate glasses by pulsed neutron total scattering. *J. Non-Cryst. Solids* **37**, 85–97 (1980).
17. Chapman, K. W. & Chupas, P. J. *In-situ Characterization of Heterogeneous Catalysts, Chapter 5: Pair Distribution Function Analysis of High-Energy X-Ray Scattering Data*. (John Wiley & Sons, 2013). doi:10.1002/9781118355923.
18. Chapman, K. W., Lapidus, S. H. & Chupas, P. J. Applications of principal component analysis to pair distribution function data. *J. Appl. Cryst.* **48**, 1619–1626 (2015).
19. Takeshi, E. & Billinge, S. J. L. *Underneath the Bragg Peaks: Structural Analysis of Complex Materials*. vol. 7 (Pergamon, Oxford, 2003).
20. Ravel, B. & Newville, M. ATHENA, ARTEMIS, HEPHAESTUS: data analysis for X-ray absorption spectroscopy using IFEFFIT. *J. Synchrotron Radiat.* **12**, 537–541 (2005).
21. Thommes, M. *et al.* Physisorption of gases, with special reference to the evaluation of surface area and pore size distribution (IUPAC Technical Report). *Pure Appl. Chem.* **87**, 1051–1069 (2015).
22. Samios, S., Stubos, A. K., Papadopoulos, G. K., Kanellopoulos, N. K. & Rigas, F. The Structure of Adsorbed CO2 in Slitlike Micropores at Low and High Temperature and the Resulting Micropore Size Distribution Based on GCMC Simulations. *J. Colloid Interface Sci.* **224**, 272–290 (2000).

23. Branton, P. J., Hall, P. G., Treguer, M. & Sing, K. S. W. Adsorption of carbon dioxide, sulfur dioxide and water vapour by MCM-41, a model mesoporous adsorbent. *J. Chem. Soc. Faraday Trans.* **91**, 2041–2043 (1995).
24. Vishnyakov, A., Ravikovitch, P. I. & Neimark, A. V. Molecular Level Models for CO2 Sorption in Nanopores. *Langmuir* **15**, 8736–8742 (1999).
25. Brunauer, S., Emmett, P. H. & Teller, E. Adsorption of Gases in Multimolecular Layers. *J. Am. Chem. Soc.* **60**, 309–319 (1938).
26. Horváth, G. & Kawazoe, K. Method for the calculation of effective pore size distribution in molecular sieve carbon. *J. Chem. Eng. Jpn.* **16**, 470 (1983).
27. Deubener, J., Müller, R., Behrens, H. & Heide, G. Water and the glass transition temperature of silicate melts. *J. Non-Cryst. Solids* **330**, 268–273 (2003).
